# Glucosinolate diversity in seven field-collected Brassicaceae species

**DOI:** 10.1371/journal.pone.0336172

**Published:** 2025-11-13

**Authors:** Lisa Pormetter, Marina Pfalz, Mervic D. Kagho, Philipp Klahn, Heiko Vogel, Juergen Kroymann, Ute Wittstock

**Affiliations:** 1 Institute of Pharmaceutical Biology, Technische Universität Braunschweig, Braunschweig, Germany; 2 Ecologie Société Evolution, CNRS/Université Paris-Saclay/AgroParisTech, Gif-sur-Yvette, France; 3 Department of Chemistry and Molecular Biology, Division of Organic and Medicinal Chemistry, University of Gothenburg, Göteborg, Sweden; 4 Department of Entomology, Max Planck Institute for Chemical Ecology, Jena, Germany; Institute for Biological Research, University of Belgrade, SERBIA

## Abstract

The glucosinolate-myrosinase system is a well-known chemical defense in the Brassicales order, which has been extensively studied in *Arabidopsis thaliana*. Here, we assessed natural variation of leaf glucosinolate content and profiles in seven species of the Brassicaceae family, using over 300 cauline leaf samples collected from wild populations in Germany and France. Total glucosinolate content varied substantially among individuals, populations and species. Analysis of glucosinolate profiles identified two types of profiles each for *Cardamine amara* and *C. pratensis*, and three profile types for *C. impatiens*. One profile type for each *Cardamine* species showed glucosinolate compositions distinct from previously described profile types. In contrast, the glucosinolate profiles of the other four species – *Lepidium draba*, *Lunaria rediviva*, *Hesperis matronalis*, and *Descurainia sophia* – were less variable. The obtained dataset paves the way for more detailed analyses of the genetic basis of glucosinolate biosynthesis in these species. Our data indicate that, among plutellid species whose larvae feed exclusively on cruciferous host plants, the oligophagous *Eidophasia messingiella* and *Rhigognostis senilella* are exposed to a diverse array of glucosinolate structures. In contrast, *Plutella porrectella* primarily encounters only a limited set of unusual glucosinolates when feeding on its preferred host plant, *H. matronalis*. Future research is required to evaluate whether this has led to specialized adaptations in this Lepidopteran herbivore. Furthermore, our study indicates that the unpredictable variation in total glucosinolate content as detected in our field-collected samples might pose a substantial challenge even to adapted herbivores.

## Introduction

Structural diversity is a hallmark of plant specialized metabolism. In addition to the presence of distinct metabolite classes and structural groups in different plant orders and families, the profiles and quantities of specialized metabolites can vary between and within plant species. Chemodiversity has a genetic basis, may evolve rapidly, and is regulated by factors such as environmental conditions and ontogeny [1]. While the role of chemodiversity in ecological interactions is generally accepted, the functional significance of intraspecific chemodiversity remains poorly understood [2,3]. One possible explanation for the maintenance of intraspecific chemodiversity throughout evolution is that variability may increase the likelihood of phenotypes matching the requirements of changing environmental conditions [1,2]. However, structural diversification is constrained by the costs associated with biosynthesis pathways and storage mechanisms, which may affect a plant’s competitiveness [2].

**Fig 1:**
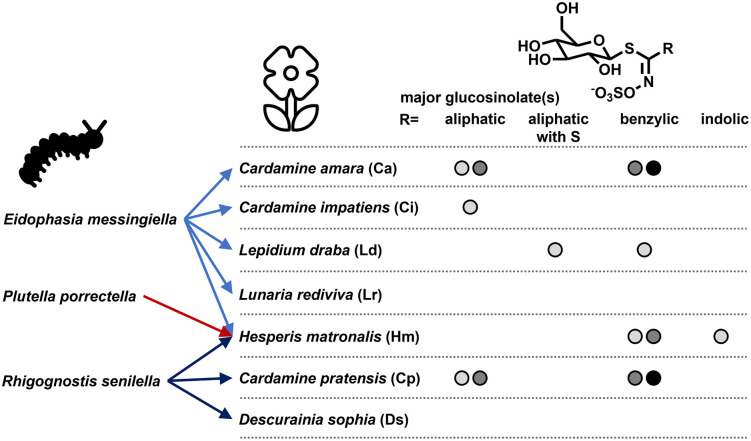
Oligophagous plutellid species and their host plants. Structural types of the major glucosinolates previously detected in leaves are indicated below the general glucosinolate structure. Different gray shades represent different glucosinolate profile types. See S1 Table for details and references. Leaf glucosinolate profiles of *L. rediviva* and *D. sophia* have not been reported previously.

Glucosinolates, amino acid-derived specialized metabolites found throughout the Brassicales order, are among the best studied chemical defenses in plants. The more than 100 glucosinolates described so far vary in the side chain attached to a common S-glucosyl thiohydroximate sulfate core structure [4,5] (Fig 1). The role of glucosinolates in direct defense against herbivores is attributed to isothiocyanates and other products, which are released upon tissue damage after glucosinolate hydrolysis by thioglucosidases known as myrosinases [6,7]. Since the complete genome sequence of *Arabidopsis thaliana* (L.) Heynh. (Brassicaceae) has become available as the first plant genome in 2000 [8], this species has been explored extensively to identify genes involved in glucosinolate biosynthesis, hydrolysis, transport, and regulation of these processes (e.g., [9–13]). In addition to the genome sequence, the enormous natural genetic variation of glucosinolate profiles among an increasing number of studied accessions has been instrumental in this endeavor [14,15]. The knowledge gained from *A. thaliana* has proven useful for the identification and manipulation of genes controlling glucosinolate metabolism in Brassicaceae crops, facilitating the development of new breeding strategies [16,17]. In contrast, relatively little is known about glucosinolate metabolism in wild members of the Brassicaceae family. Yet, this family comprises 338 genera and 3.700 species [18]. Moreover, some wild members are known to contain unusual glucosinolates, i.e., glucosinolates with restricted occurrence in few species and glucosinolates not commonly found in *A. thaliana* or Brassicaceae crops or not found in considerable amounts [5,19,20]. Assessing the natural variation of glucosinolates in wild species may lay the foundation for extending studies on glucosinolate biosynthesis beyond the compounds found in *A. thaliana*.

Among herbivores specialized on glucosinolate-containing plants, the larvae of the diamond back moth, *Plutella xylostella* (Lepidoptera: Plutellidae), process glucosinolates very quickly to desulfoglucosinolates by gut-expressed glucosinolate sulfatases [21]. In contrast to glucosinolates, the desulfated compounds are no longer substrates of myrosinases and are excreted with the frass. As long as the activity of gut glucosinolate sulfatases is sufficient to rapidly convert all glucosinolates and to outcompete the myrosinases ingested with the plant material, *P. xylostella* can feed on glucosinolate-containing plants with impunity. This highly efficient mechanism makes *P. xylostella* one of the most devastating pests on cruciferous crops such as oilseed rape and cabbages [22]. The three known glucosinolate sulfatases of *P. xylostella* differ in their substrate specificity and inducibility [23]. Together, they reflect the very broad host range of the insect [23].

Besides *P. xylostella*, the Plutellidae *sensu stricto* comprise five genera with more than 50 species [24,25]. The majority of plutellid species feeds on Brassicales [24]. It is reasonable to assume that these species are also protected from the adverse effects of the glucosinolate-myrosinase system by an efficient mechanism, likely glucosinolate sulfatases. However, unlike the polyphagous *P. xylostella*, other glucosinolate-feeding plutellids are oligo- or monophagous, i.e., they have few or even only a single reported host species. Natural variation in glucosinolate profiles or total content may pose a challenge to these species unless their (assumed) glucosinolate sulfatases act efficiently on a broad range of glucosinolates.

In the present study, we selected seven species of wild Brassicaceae, which are host plants to three oligo- to monophagous plutellid species, *Eidophasia messingiella*, *Rhigognostis senilella*, and *Plutella porrectella*, to study natural variation (Fig 1). Although glucosinolate profiles have been described for all seven host plant species (Fig 1, S1 Table), information on leaf glucosinolates is scarce and often derived from lab-grown plants. Our selection included three *Cardamine* species, each with several described glucosinolate profile types. Leaves of *C. amara* L. are rich in branched-chain aliphatic glucosinolates and/or benzyl glucosinolate, depending on the profile type [26–28]. *C. impatiens* L. leaves accumulate alkenyl glucosinolates [28,29]. Previous analyses of several populations of *C. pratensis* L. identified six glucosinolate profile types with various combinations of aliphatic glucosinolates containing hydroxymethyl groups, branched-chain aliphatic glucosinolates and/or 4-hydroxybenzyl glucosinolate as major compounds [28,30]. *Lepidium draba* L. leaves are known to contain the relatively rare 4-(methylsulfonyl)butyl glucosinolate alongside 4-(methylsulfinyl)butyl and 4-hydroxybenzyl glucosinolate, depending on the profile type [31,32]. Glucomatronalin (3,4-dihydroxybenzyl glucosinolate) provides the core structure of unusual apiosylated glucosinolates found in *Hesperis matronalis* L. [31,33]. Their absolute or relative content in leaves have not been reported so far. For *Descurainia sophia* (L.) Webb ex Prantl and *Lunaria rediviva* L. leaf glucosinolate content and profiles have not been described previously.

The primary goal of this study was to assess natural variation in leaf glucosinolate content and profiles of the selected plant species using material collected from various field sites in France and Germany. Additionally, we were interested in the question if content and profiles are uniform enough within and across populations to expect an adaptation of mono-/oligophagous plutellid species to certain structural types of glucosinolates and/or total glucosinolate levels.

## Results

**Fig 2:**
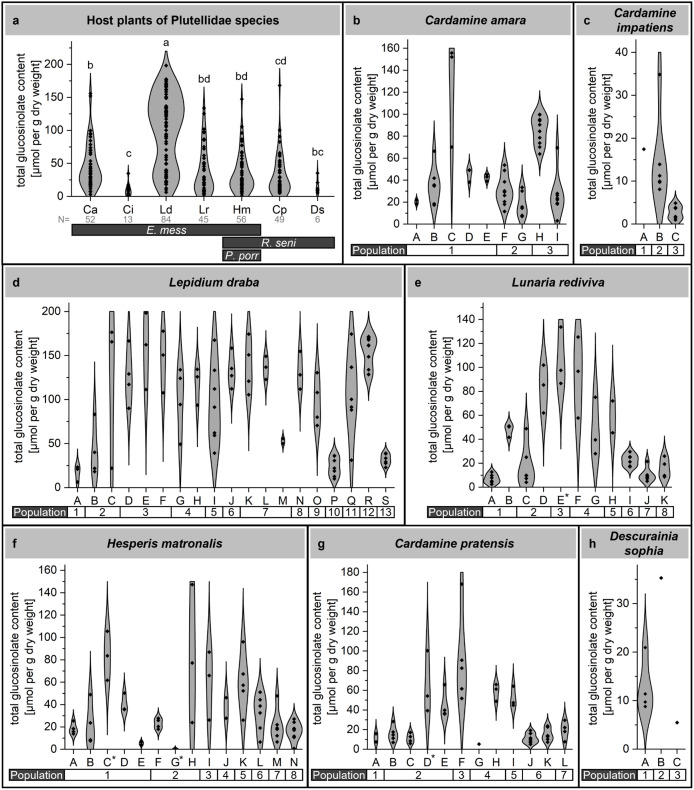
Total glucosinolate content in field-collected cauline leaves of plutellid host plants. a) Total content across all populations and samples of each host plant species represented as violin plots. Plant species abbreviations are as in Fig 1. Sample numbers (N) are given below species abbrevations. Horizontal bars indicate host plants of *E. messingiella* (*E. mess*), *R. senilella* (*R. seni*) and *P. porrectella* (*P. porr*). Different letters above the data indicate that species were significantly different (ANOVA with Post-hoc *t*-tests with Tukey using Johnson-transformed data). b-h) Total content in populations of each species represented as violin plots. Several populations (as indicated by numbers in the horizontal bar) were sampled repeatedly in 2021 and 2022. During each sampling event (as denoted by uppercase letters), cauline leaves of several individuals were harvested. An asterisk next to the uppercase letter (panels e-g) indicates that rosette leaves were harvested. Each data point (diamond shape) in panels a-h represents one individual plant.

To determine which glucosinolates the three plutellid species may encounter in their natural environment, we sampled their seven host plant species from a total of 45 populations (three to thirteen populations per plant species, S2 Table, Fig 2). Qualitative and quantitative analyses of glucosinolate profiles from the resulting 310 samples of (usually) cauline leaves revealed the presence of 35 glucosinolates, predominantly with aliphatic and benzylic side chains. Glucosinolate identification was initially based on the molecular masses (m/z values) of their desulfo-derivatives. For 22 known glucosinolates, we were able to combine this information with either the desulfoglucosinolate HPLC retention time (S3 Table) and UV spectrum or the molecular mass (m/z values) of the corresponding intact glucosinolate, or both (S4 Table). Glucosinolate identification was further supported by the MS^2^ spectra of their desulfo-derivatives, compared to those of standards where available (S4 and S5 Tables, S1 Appendix). For three glucosinolates with branched propyl or butyl side chains, isomer assignment was achieved through comparison of retention times with those of synthesized standards and spiking experiments (S4 Table). Six additional previously described glucosinolates were present in certain species based on their masses or MS^2^ spectra of the corresponding desulfo-derivatives, but their amounts were too low for quantification, and further structural confirmation was not undertaken (S4 Table). Two of the detected glucosinolates have not been identified before [33]. Their UV spectra, retention times, molecular masses, previous assumptions in the literature [33], and their presence in mixtures with similar compounds suggest their identities as 2-(hydroxymethyl)butyl and 4-apiosyloxybenzyl glucosinolate in *C. pratensis* and *H. matronalis*, respectively (S4 Table). As there are other possible isomers of 2-(hydroxymethyl)butyl glucosinolate, we refer to the glucosinolate as unidentified hydroxypentyl glucosinolate or isomer hereafter.

To get a first impression of quantitative variation, we compared total glucosinolate content among species as well as among populations and individuals of the same species (Fig 2; S6 and S7 Tables). This revealed high levels of variation both among and within species. *L. draba* frequently accumulated very high glucosinolate levels (>75 µmol/g, up to 200 µmol/g) while all other species typically had glucosinolate contents of <50 µmol/g (Fig 2a). The difference between *L. draba* and all other species was statistically significant (p < 0.0001) according to ANOVA with Johnson-transformed data (Fig 2a). The overall lowest glucosinolate contents were found in *C. impatiens* and *D. sophia*, but this may not be representative due to the low sample numbers (Fig 2a, c, h). Variances of total glucosinolate content differed significantly between species (S8 Table). A closer examination of the five species with sufficient sample numbers revealed substantial variation both across and within populations (Fig 2b, d–g). For example, within samples of population 1 of *C. amara*, total content varied between roughly 20 and 160 µmol/g. Although *L. draba* generally exhibited very high glucosinolate levels, samples from populations 1, 10, and 13 contained less than 50 µmol/g. Samples from population 3 were rather homogenous in their total glucosinolate content while samples from populations 2, 5, and 11 spanned almost the entire range of total glucosinolate content observed in this species.

**Fig 3:**
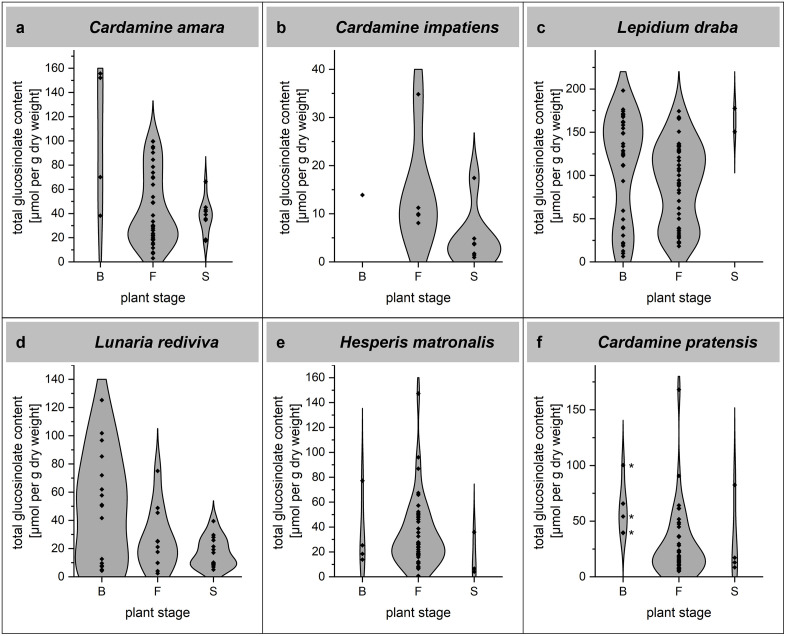
Total glucosinolate content in cauline leaves of plants at different developmental stages. Panels a-f represent species for which cauline leaves were collected at different stages: B (bolting), F (flowering), and S (siliques present). *D. sophia* samples were all from flowering plants and are therefore not included. Data are represented as violin plots, each data point represents one individual plant. An asterisk next to the data points (panel f) indicates that rosette leaves were harvested.

Variation in total glucosinolate content could not be related to any obvious sample characteristics such as field site location, population, year or month of sampling, presence of herbivores, or leaf damage for any of the analyzed plant species. We also tested whether total glucosinolate content might be related to the developmental stage of the plant at the time of sampling. Due to different sample numbers, correlations could not be determined with sufficient certainty. However, for the two best sampled species across the three stages analyzed, *L. rediviva* and *C. amara*, as well as for *C. impatiens*, we observed a trend towards lower glucosinolate content in cauline leaves from fruiting plants compared to those from bolting or flowering plants (Fig 3).

**Fig 4:**
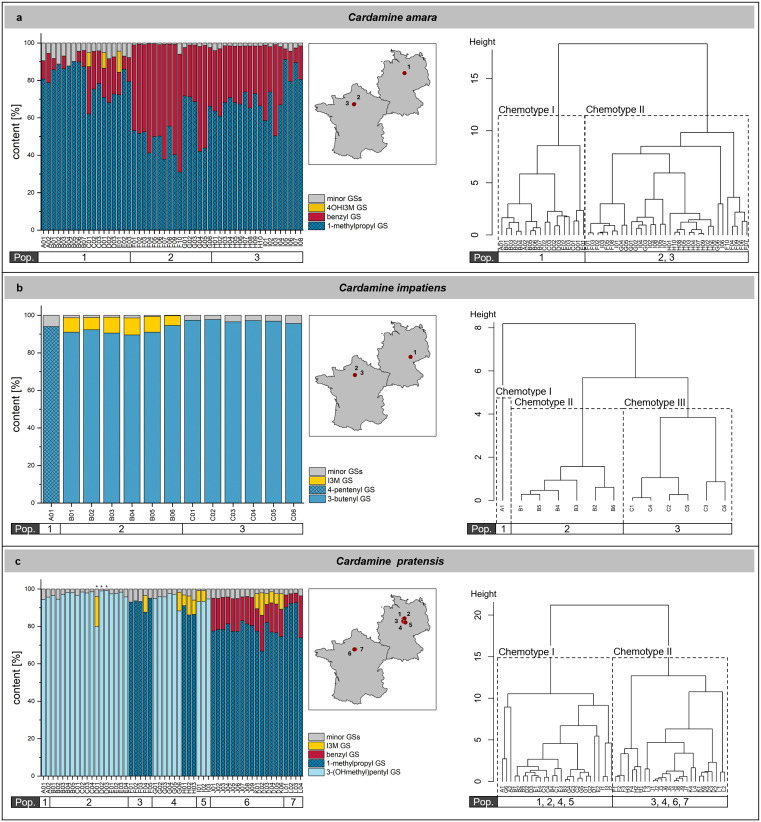
Variation of glucosinolate profiles of *C. amara*, *C. impatiens* and *C. pratensis.* Content of individual glucosinolates (determined as µmol/g dry weight) is represented as a percentage of the total glucosinolate content for each sample of *C. amara* (a), *C. impatiens* (b), and *C. pratensis* (c) together with a map of France and Germany showing the location of each population (red dots) and a dendrogram generated by cluster analysis for assignment of glucosinolate profile types. Populations are indicated in the horizontal bar below the bar graphs and dendrograms. Minor glucosinolates were defined as those present at <5% of the total glucosinolate content of a sample. (#) Tentatively identified glucosinolates. Please note that individual glucosinolates listed in the legend for some samples may also be present among minor glucosinolates in other samples (see S6 and S7 Tables for the complete data set). Asterisks above bars indicate samples of rosette leaves. Maps were created with RStudio using the ggplot2 package and the maps package based on the CIA World Data Bank [69–71].

Next, we analyzed glucosinolate profiles, i.e., the presence and quantities of individual glucosinolates, in samples of all species (S6 and S7 Tables). For the previously analyzed species (S1 Table), we detected the expected glucosinolates with few exceptions. For example, among major glucosinolates, none of our samples of *C. pratensis* contained 4-hydroxybenzyl glucosinolate and we did not find 4-hydroxyindol-3-ylmethyl glucosinolate in our *H. matronalis* samples (S6 and S7 Table). Despite the extensive variation in total glucosinolate content, the profiles were relatively homogenous. However, we observed considerable variation in the glucosinolate profiles of the three *Cardamine* species (Fig 4). The most striking differences between samples were the proportion of benzyl glucosinolate in *C. amara* (Fig 4a), the presence of either 4-pentenyl or 3-butenyl glucosinolate as a major compound in *C. impatiens* (Fig 4b), and the predominance of either 3-(hydroxymethyl)pentyl or 1-methylpropyl glucosinolate as well as the presence/absence of major proportions of benzyl glucosinolate in *C. pratensis* (Fig 4c).

All *C. amara* samples (N = 52) contained a high proportion of 1-methylpropyl glucosinolate (31–91%), while relative benzyl glucosinolate content varied between 2 and 63% (Fig 4a). Together, these glucosinolates accounted for >80% of the total glucosinolate content in all samples. Samples from the French populations tended to have a higher benzyl glucosinolate proportion than samples from the German population. To test for patterns of chemical diversity, we used cluster analysis on the standardized data set of relative contents to reveal differences also among profiles of minor glucosinolates. The samples formed two major branches supporting the presence of two types of profiles (Fig 4a). Inspection of the data showed that these profile types were differentiated based on minor glucosinolates. Samples from the German population of *C. amara* possessed profile I (higher proportions of 1-methylethyl and 2-methylpropyl glucosinolate), while samples from the two French populations had profile II (lower proportions of 1-methylethyl and 2-methylpropyl glucosinolate). Taken together, we identified 1-methylpropyl and benzyl glucosinolate as major glucosinolates in *C. amara*, consistent with findings by [26]. A previous study [27] reported 2-methylpropyl glucosinolate as a major glucosinolate in *C. amara*, but did not distinguish it from 1-methylpropyl glucosinolate. Most of our samples (33 of 52) contained small amounts of 2-methylpropyl glucosinolate, but in no case it was a major glucosinolate (S6 and S7 Tables).

The glucosinolate profiles of *C. impatiens* samples (N = 13) were dominated by either 4-pentenyl glucosinolate (German population, one sample) or 3-butenyl glucosinolate (French populations; Fig 4b). Samples formed three main clusters upon cluster analysis using standardized data. These clusters represent the three sampled populations. The first branch displaying profile I corresponds to the German sample (N = 1) with >90% 4-pentenyl glucosinolate. This profile is distinct from a previously described profile type with equal proportions of 4-pentenyl and 3-butenyl glucosinolate as major compounds [29] and might indicate a new glucosinolate profile type. The second branch with two clusters comprises all French samples which contain 3-butenyl glucosinolate at ≥90% of the total glucosinolate content (similar to [28]). The two clusters within this branch correspond to the two populations sampled in France and differ in their relative content of indol-3-ylmethyl glucosinolate at >5% (profile II; 7.5% on average) or <5% (profile III; 2.5% on average) of the total content (Fig 4b). This difference could be caused by different local environments of the two populations (e.g., induction by herbivores and pathogens) or it could be genetically fixed.

For *C. pratensis*, seven populations were included in our analysis (N = 54). In three German populations, we found 3-(hydroxymethyl)pentyl glucosinolate to be the major glucosinolate in all samples (>90% relative content). In three additional populations from Germany and France, 1-methylpropyl glucosinolate dominated the profile of all samples (>60% relative content), while samples from another German population contained either one or the other of these glucosinolates with >80%. Cluster analysis with standardized data revealed two major clusters suggesting the presence of two types of glucosinolate profiles. Profile I was dominated by 3-(hydroxymethyl)pentyl glucosinolate, similar to a previously described profile type [30] while profile II was characterized by a high proportion of 1-methylpropyl glucosinolate. Benzyl glucosinolate was absent in profile I, but present in eleven of 19 samples with profile II. None of our samples contained detectable amounts of 4-hydroxybenzyl glucosinolate, a compound previously reported as a major glucosinolate in *C. pratensis* [28,30]. Overall, our analyses demonstrated the presence of distinct profile types in the three *Cardamine* species, which, in part, differed from previously described glucosinolate profiles.

**Fig 5:**
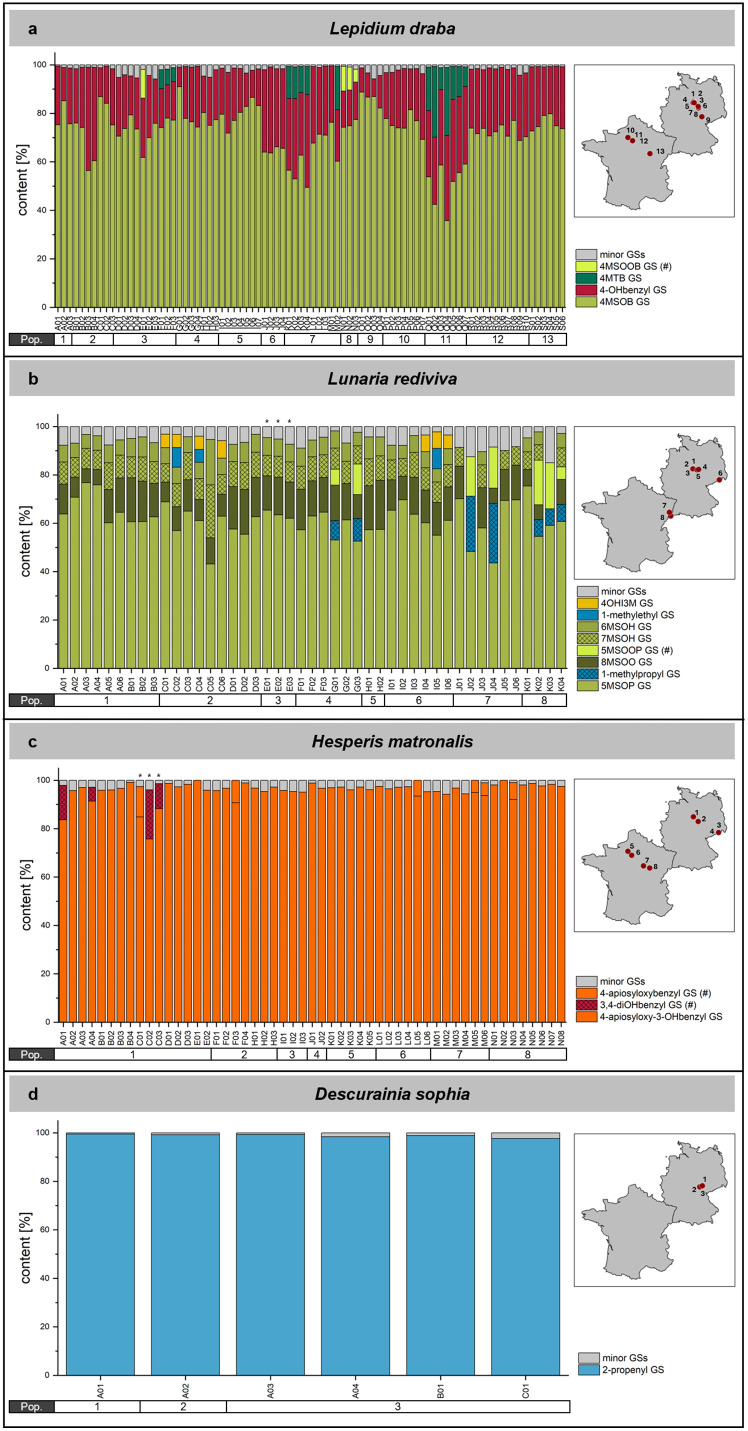
Glucosinolate profiles of *L. draba*, *L. rediviva*, *H. matronalis,* and *D. sophia* cauline leaves. Content of individual glucosinolates (determined as µmol/g dry weight) is represented as a percentage of the total glucosinolate content for each sample of *L. draba* (a), *L. rediviva* (b), *H. matronalis* (c)*,* and *D. sophia* (d) together with a map of France and Germany showing the location of each population (red dots). Populations are indicated in the horizontal bar below the bar graph. Minor glucosinolates were defined as those present at <5% of total glucosinolate content of a sample. (#) Tentatively identified glucosinolates. Please note that individual glucosinolates listed in the legend for some samples may also be present among minor glucosinolates in other samples (see S6 and S7 Tables for the complete dataset). Asterisks above bars in panels b and c indicate samples of rosette leaves. Maps were created with RStudio using the ggplot2 package and the maps package based on the CIA World Data Bank [69–71].

For the other four species included in our study, *L. draba*, *L. rediviva*, *H. matronalis,* and *D. sophia*, cluster analysis did not suggest the assignment of distinct profile types. Although there was some variation in the profiles, they were were relatively homogenous across all samples of each species (Fig 5).

The profiles of *L. draba* samples (N = 84) were dominated by 4-(methylsulfinyl)butyl glucosinolate and contained 10–40% 4-hydroxybenzyl glucosinolate, as well as 4-(methylthio)butyl and tentatively identified 4-(methylsulfonyl)butyl glucosinolate in varying proportions (Fig 5a). High proportions of 4-(methylthio)butyl glucosinolate were found mostly in three populations from both France and Germany. Although glucosinolates were largely the same as in previous studies, their proportions differed from earlier reports [31,32].

All *L. rediviva* samples (N = 45) contained mainly 5-(methylsulfinyl)pentyl glucosinolate (>50% in most samples; Fig 5b). In addition, most samples contained considerable levels of other ω-(methylsulfinyl)alkyl glucosinolates with longer alkyl chains (C6, C7, C8; together about 20–50%). In a few cases, more frequently in French populations but not restricted to them, 1-methylpropyl and tentatively identified 5-(methylsulfonyl)pentyl glucosinolate replaced these glucosinolates as the major compounds, alongside 5-(methylsulfinyl)pentyl glucosinolate (Fig 5b). 1-Methylethyl glucosinolate and several indolic glucosinolates were mostly present as minor glucosinolates (S6 and S7 Tables).

Apart from three samples of *H. matronalis* in which no glucosinolates were detected, all remaining *H. matronalis* samples (N = 53) were dominated by 4-apiosyloxy-3-hydroxybenzyl glucosinolate (>90% in most samples, as in [33]; Fig 5c). Only in four samples from population 1 (Germany), considerable proportions of the non-apiosylated precursor 3,4-dihydroxybenzyl glucosinolate (glucomatronalin) previously described in seeds and roots of *H. matronalis* [33–36] were also present (Fig 5c). Additionally, some samples contained a compound tentatively identified as 4-apiosyloxybenzyl glucosinolate (Fig 5c). The profiles of our samples were similar to the profile described in [33], but distinct from those found in another report, where 4-hydroxybenzyl- and an indolic glucosinolate appeared as major glucosinolates [31]. Our analysis of six samples from two populations of *D. sophia* showed a uniform profile with more than 95% 2-propenyl glucosinolate (Fig 5d). *D. sophia* cauline leaves also contained 3-butenyl, 1-methylethyl, benzyl and 4-methoxyindol-3-ylmethyl glucosinolate (S6 and S7 Tables).

Taken together, *L. draba* and *L. rediviva* were dominated by mixtures of several glucosinolates with high proportions of aliphatic glucosinolates with sulfur-containing side chains, while *H. matronalis* and *D. sophia* each contained a single major glucosinolate. Leaf glucosinolate profiles of *L. rediviva* and *D. sophia* are reported here for the first time.

## Discussion

Although more than 300 species of the Brassicaceae have been studied with respect to glucosinolate accumulation, our knowledge on within-species chemical diversity is limited to relatively few species (e.g., [27,30,37–41]). However, systematic studies of within-species chemodiversity could pave the way for elucidating the genetic basis of biosynthesis of glucosinolates that are not found in *A. thaliana*, for example dihydroxybenzyl, ω-(methylsulfonyl)alkyl and phenylhydroxyalkyl glucosinolates as well as glucosinolates with additional glycosylation of the core or side chain structure. The seven species investigated here were selected based on their role as known host plants of three plutellid species with different host plant spectra (Fig 1). At the same time, they represent tribes of the core Brassicaceae assigned to lineage I (Cardamineae, closely related to Camelineae with *Arabidopsis*; Descurainieae; Lepidieae), lineage III (Hesperideae), or not assigned to any lineage (Biscutelleae) [42]. Previous work on these seven species has established general glucosinolate profiles using different plant parts or entire plants, lab-grown plants or plants from natural sites, individual plants or pooled material from several individuals (Fig 1, S1 Table and references therein). In our present work, we analyzed material from individual plants collected at natural sites. As we are interested in the interaction of chewing herbivores of the Plutellidae with their various glucosinolate-containing host plants, we used the plant stage in which larvae are usually present, i.e., the generative phase. In this phase, cauline leaves make up most of the leaf area, therefore we analyzed primarily cauline leaves.

Our dataset revealed that both total glucosinolate content and the variances of total glucosinolate content differ significantly among species (Fig 2, S6 and S8 Tables). In general, total glucosinolate content varied greatly between individuals of the same species. In addition to seasonal effects, this intraspecific variation could result from different herbivore pressures and various other environmental factors. There was no obvious correlation between collection time point, observed herbivore damage, or presence of herbivores on the plants and total glucosinolate content. Future research should test under controlled conditions to which degree this variation is genetically determined. From a herbivore’s perspective, this variation might translate into a very low degree of predictability of total glucosinolate content within a given species, population or at a specific time point. In case of *L. rediviva*, *C. amara*, and *C. impatiens*, we observed a trend of decreasing glucosinolate content from bolting through flowering to seed set (Fig 3). This would be in agreement with a defensive role of the glucosinolate-myrosinase system, especially during the vulnerable plant stages before seed set. However, experiments conducted under controlled conditions are needed to test this rigorously with all investigated species.

For three of the species studied, all belonging to the genus *Cardamine*, we found distinct glucosinolate profile types, as one might expect based on previous work (S1 Table) and their close relatedness to *A. thaliana* with its extensively studied chemodiversity. The two French populations of *C. amara* had profiles of major glucosinolates (1-methylpropyl and benzyl glucosinolate) similar to those described for plants from a Danish population [26]. Plants from the German population possessed a different profile, due to the abundance of some minor glucosinolates, but were also characterized by a relatively low proportion of benzyl glucosinolate (Fig 4). Cluster analysis for our *C. impatiens* samples revealed three profile types, with each population representing one of them. All three profile types were dominated by a single glucosinolate, either 3-butenyl or 4-pentenyl glucosinolate, and were therefore distinct from the profile described for a North American population by [29]. Profile type II (detected for a French population) had the highest similarity with the glucosinolate profile described previously for plants from the Alps [28]. Samples from the German population, dominated by 4-pentenyl glucosinolate, might possibly represent a new glucosinolate profile type. The highest number of samples and populations among *Cardamine* species were available for *C. pratensis*. In this species, we identified plants of two distinct profile types within the same population (population 4, Fig 4). Profile type I, dominated by 3-(hydroxymethyl)pentyl glucosinolate, was present in four German populations, and profile type II, dominated by 1-methylpropyl glucosinolate, was present in two German and the two French populations. Previous studies on *C. pratensis* detected variation with respect to presence and abundance of 4-hydroxybenzyl glucosinolate, with samples from Danish populations having high levels of this glucosinolate [30]. Our samples differed from those of the Danish populations as we did not detect 4-hydroxybenzyl glucosinolate at all (S6 Table). A sample from North America lacking this glucosinolate had high levels of 3-(hydroxymethyl)pentyl glucosinolate instead [30], similar to profile type I described in the present study. Thus, profile type II found in our study might possibly represent a new glucosinolate profile type of *C. pratensis*.

While we were able to describe distinct profile types of *Cardamine* species, there was much less variation in the glucosinolate profiles of the other four species studied. *H. matronalis* and *D. sophia* samples were dominated by 4-apiosyloxy-3-hydroxybenzyl and 2-propenyl glucosinolate, respectively, with very low proportions of other glucosinolates. The absence of aliphatic glucosinolates in *H. matronalis* leaf samples is in agreement with a previous report [33] while another study detected considerable amounts of a glucosinolate with sulfur-containing side chain in leaves [31]. This might indicate the existance of distinct glucosinolate profile types of this species. Glucosinolate profiles of *L. draba* were similar to previous reports, with 4-(methylsulfinyl)butyl glucosinolate being the most abundant glucosinolate in most samples, followed by 4-hydroxybenzyl glucosinolate [31,32]. However, 4-(methylthio)butyl glucosinolate has not been reported to accumulate in *L. draba* in considerable amounts before. Leaf glucosinolate profiles of *L. rediviva* have not been reported before (previous work studied seed glucosinolate hydrolysis products, reviewed in [19]). Despite its position outside of Brassicaceae lineage I, the complex glucosinolate profile of *L. rediviva* cauline leaves showed the greatest similarity with the glucosinolate profile of *A. thaliana* among the studied species. Methylsulfinylalkyl glucosinolates of different side chain lengths dominated in all samples.

**Fig 6:**
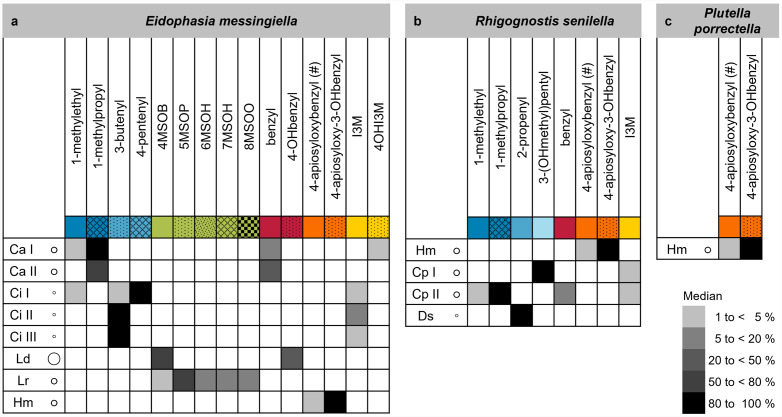
Glucosinolate profiles potentially encountered by plutellid species with different host plant ranges. Host plant species of *E. messingiella* (a), *R. senilella* (b), and *P. porrectella* (c) are abbreviated as in Fig 1 and are listed with their glucosinolate profiles (as indicated by side chain abbreviations as in S4 Table) found in our study. The gray scale in the heatmaps refers to relative content (median of all samples). Glucosinolates with a median relative content of <1% are not included. (#) Tentatively identified glucosinolates. The color code below the glucosinolate side chain names refers to structural groups and corresponds to that in Figs 4 and 5 (side chain color code: blue, branched-chain and alkenyl; green, sulfur-containing; red, benzenic/ non-apiosylated; orange, benzenic/ apiosylated; yellow, indolic). Total glucosinolate levels are indicated by circles next to the plant species name (corresponding to a median of >100 (large), > 15 (medium) and <15 (small) µmol/g dry weight).

Our study revealed large variation in total glucosinolate content and various degrees of glucosinolate profile variation among species, populations and individuals. A herbivore may perceive the qualitative and quantitative chemical diversity within and between species growing in a certain patch or even within an individual plant as a “phytochemical landscape” [3,43] or “chemical information space” [1]. Thus, chemodiversity may act as a central factor for feeding decisions or oviposition choices and, hence, determine level of damage to individual plants. While the present study is limited to the analysis of glucosinolates, plants accumulate a suite of diverse chemicals that are all part of the environment a herbivore is exposed to. To visualize the glucosinolate profiles an insect may encounter in the field, we compiled lists of host plants with glucosinolate profiles for each insect species of interest as heat maps (Fig 6). *E. messingiella* feeds on several species with quite different glucosinolate profiles. To circumvent the potentially detrimental effects of the glucosinolate-myrosinase system, it must deal with a broad range of structurally different glucosinolates, including branched-chain aliphatic, alkenyl-, methylthio-/methylsulfinylalkyl-, benzenic, apiosylated benzenic, and indolic glucosinolates. The occurrence of several profile types (as detected in this study and previous work on *Cardamine* species, respectively, S1 Table) adds another level of uncertainty for larvae, even if only one host plant species is considered. In contrast, *P. porrectella* with *H. matronalis* as the preferred host plant encounters mostly apiosylated benzenic glucosinolates. Therefore, one might expect larvae of this species to express a glucosinolate sulfatase specialized for these unusual compounds. Although *R. senilella* has only three known host plant species, it will likely have to deal with almost the entire range of structural groups of glucosinolates, similar to *E. messingiella*. Next to the glucosinolate profiles, the absolute glucosinolate content of plants encountered in the field might be an important aspect directing sulfatase evolution. However, our data suggest that glucosinolate content is rather unpredictable and varies largely between species, but also between populations and individuals within a population.

When weighing the relative importance of variation in total glucosinolate content versus differences in glucosinolate profiles, it is important to account for the possible variation in hydrolysis product formation and the toxicity of the breakdown products. For example, the accumulation of small amounts of an alkenyl glucosinolate (as observed in our *C. impatiens* and *D. sophia* samples) might be as effective as chemical defense as the accumulation of very high levels of 4-(methylsulfinyl)butyl glucosinolate (as seen in our *L. draba* samples) due to possible higher toxicity of allyl isothiocyanate compared to 4-(methylsulfinyl)butyl isothiocyanate [44,45]. However, the toxicity of isothiocyanates with various side chains may also depend on the herbivore species [46,47]. Furthermore, plants may produce other products besides isothiocyanates upon glucosinolate breakdown, e.g., nitriles, epithionitriles and oxazolidine-2-thiones, depending on the presence of specifier proteins and the glucosinolate side chain structure [48,49]. For instance, nitriles appear to be less toxic than isothiocyanates with the same side chain, but epithionitriles derived from the same glucosinolate might be toxic [50,51]. Thus, glucosinolate breakdown upon herbivore damage adds another layer of complexity to host plant chemistry. As toxic breakdown products are only formed upon damage to the plant, the ultimately perceived “phytochemical landscape” is even more unpredictable for the herbivore than just the glucosinolate content and profile, and herbivores are likely to use additional chemical or non-chemical cues to identify their suitable feeding sites. In support of a central role of the glucosinolate-myrosinase system in herbivore feeding decisions, a previous study found the feeding intensity of the weevil *Ceutorynchus cardariae* on a range of glucosinolate-containing plants to be associated with the glucosinolate profile rather than genetic similarity of the host plants [31].

As a model plant with a simple genome and numerous genetic tools, *A. thaliana* has been crucial for uncovering the genetic basis of glucosinolate biosynthesis. However, studying wild species, with their diverse genetic backgrounds and adaptations, offers valuable insights into the evolutionary and ecological factors shaping glucosinolate diversity and controlling their accumulation. The dataset generated in the present study provides a foundation for in-depth exploration of the biosynthetic pathways underlying glucosinolate diversity in wild species. Notably, the occurrence of 3-butenyl versus 4-pentenyl glucosinolate in *C. impatiens* indicates genotypic variation in methylthioalkylmalate synthase genes, which are known to determine the carbon chain length of methionine-derived glucosinolates [52]. Similarly, the observed quantitative differences in branched-chain amino acid-derived glucosinolates, such as 1-methylethyl and 2-methylpropyl glucosinolate, in *C. amara* suggest variation in cytochrome P450 monooxygenases of the CYP79 family, enzymes that mediate the initial steps of glucosinolate biosynthesis [53].

In summary, we found a high level of inter- and intraspecific variation in total glucosinolate content. For the three analyzed *Cardamine* species, this extended to variation of glucosinolate profiles. Among the identified profile types of *Cardamine* species, three were principally different to those of previously described ones. Future analyses should be aimed at dissecting genetically determined variation in more detail. Furthermore, natural variation at the level of glucosinolate hydrolysis should be assessed to better understand which glucosinolate-derived products herbivores have to avoid or overcome when feeding on their host plants. Studying wild Brassicaceae species at the genetic level will enrich our understanding of specialized metabolite evolution and may lead to new discoveries beyond what *A. thaliana* alone can provide.

## Materials and methods

### Plant material and sampling

Plant material was collected from field sites in Germany and France in 2021 and 2022 (S2 Table). Populations were located based on The Global Biodiversity Information Facility (GBIF; www.gbif.org). Species were identified in the field based on their morphological characters ([54]; supported by flora incognita, https://floraincognita.de/) and documented photographically and as a herbarium specimen whenever possible (S2 Appendix). Herbarium specimen have been deposited at Herbarium KIEL (www.herbarium.uni-kiel.de; inventory numbers KIEL0005124-KIEL0005136). For confirmation, samples were subjected to DNA barcoding (at least one individuum per population; S9 Table). Cauline leaves were removed from plants using a scalpel, placed on dry ice or ice immediately, shock frozen in liquid nitrogen on the same day and stored at −20°C. Samples were lyophilized to dryness, followed by grinding to a fine powder using an MM 400 Retsch swing mill (Retsch GmbH, Haan, Germany). In the rare cases where cauline leaves were not yet present, rosette leaves were harvested (as indicated in tables and figures; S2 Table). If insect larvae were present on a plant, the cauline leaves on which the larvae were found were collected, and the larvae were kept in DNA/RNA Shield (Zymo Research, Freiburg, Germany) at −20°C for identification by DNA barcoding (S10 Table).

Written consent for sampling of the relevant plants in protected areas was obtained from the responsible local authorities „Untere Naturschutzbehörde, Landratsamt Nordhausen“, „Untere Naturschutzbehörde, Landkreis Harz“, „Landesdirektion Sachsen”, and „Naturschutzbehörde des Landkreises Görlitz”. In addition, written consent for sampling of *L. rediviva* was obtained from the following authorities: „Untere Naturschutzbehörde, Landratsamt Nordhausen“, „Untere Naturschutzbehörde, Landkreis Harz“, „Niedersächsischer Landesbetrieb für Wasserwirtschaft, Küsten- und Naturschutz“, and „Landesdirektion Sachsen”. For field collection in France, written consent was obtained from Ministère de la transition écologique (France).

### DNA barcoding

Following DNA extraction [55], internal transcribed spacer sequences were amplified with ITS_S2F (5’-ATGCGATACTTGGTGTGAAT-3’) and ITS_S3R (5’-GACGCTTCTCCAGACTACAAT-3’) primers. PCR products were purified using the NucleoSpin Gel and PCR Clean-up kit (Macherey-Nagel SRS, Hoerdt, France) and sequenced. Sequences are provided in S9 Table. Nucleotide sequences were subjected to BLAST searches (Blastn) against GenBank or an inhouse database compiled from barcoding data of the target species and other species of the same genera, obtained from the Barcode of Life Data System (www.boldsystems.org; [56]).

### Glucosinolate standards and synthesis of glucosinolates

**Fig 7:**
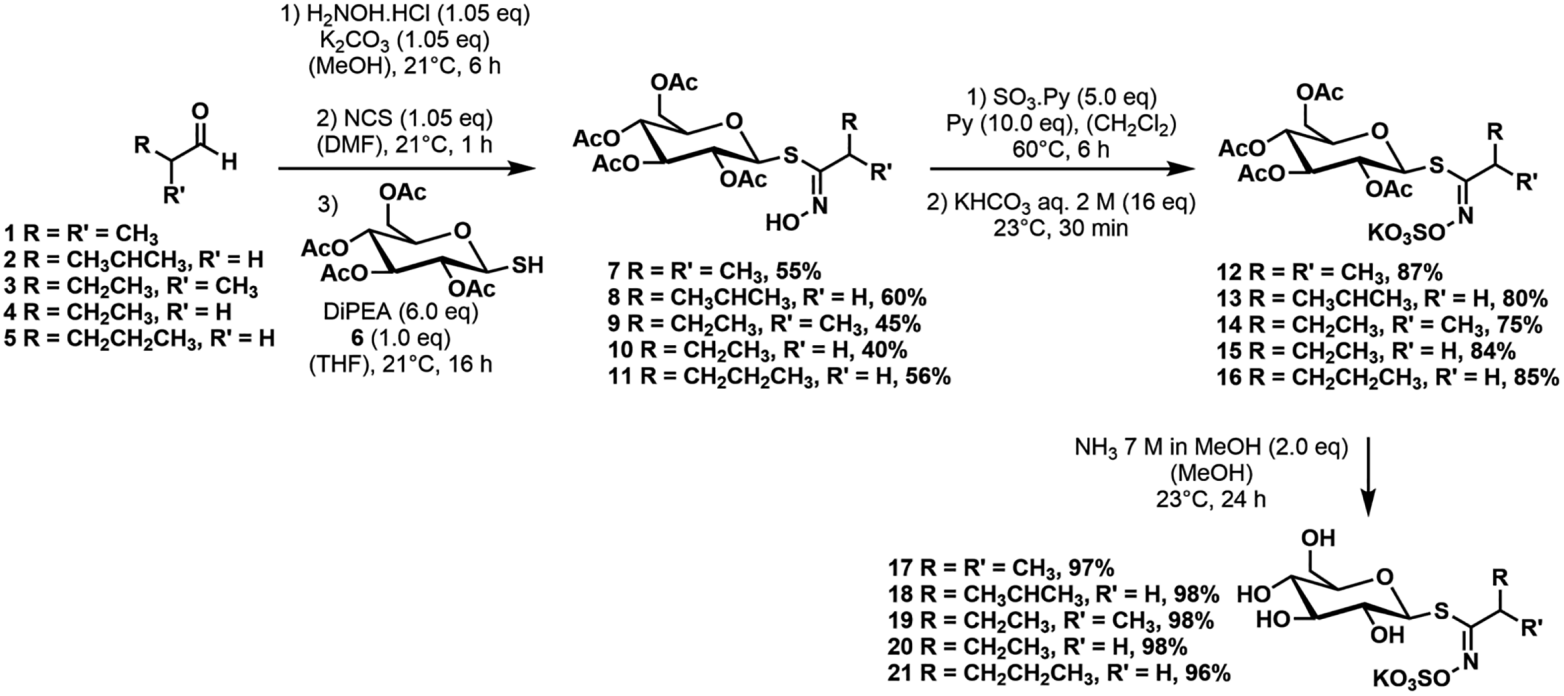
Scheme of glucosinolate synthesis.

Glucosinolates were purchased from Phytoplan (Heidelberg, Germany) with the following exceptions. 4-Hydroxybenzylglucosinolate (>99%) was purified from seeds of *Sinapis alba* L.. Authentic standards of desulfo-3-(hydroxymethyl)pentyl and desulfo-2-hydroxy-3-methylpentyl glucosinolate [29] were provided by Dr. Niels Agerbirk (University of Copenhagen, Denmark). A sample of authentic 4’-O-β-D-apiofuranosylglucomatronalin [33] was obtained from Prof. Sabine Montaut (Laurentian University, Sudbury, Ontario, Canada) and desulfated by Dr. Niels Agerbirk prior to providing it for the present analyses. Dr. Niels Agerbirk also provided a desulfoglucosinolate mix from *Cleome spinosa* Jacq. (Cleomaceae) seeds [57] as a tentative reference for desulfo-2-hydroxy-2-methylbutyl glucosinolate. An extract of *A. thaliana* Columbia-0 seeds treated as described below for generation of desulfoglucosinolates served as a reference for identification of desulfo-7-(methylsulfinyl)heptyl and desulfo-9-(methylsulfinyl)nonyl glucosinolate. Glucosinolates with n-butyl, 1-methylpropyl, 2-methylpropyl, n-propyl and 1-methylethyl side chains were synthesized starting from their respective commercially available aldehydes following a classical hydroxamic acid approach [58] as done earlier by us [59,60] (Fig 7, S1 Methods). First the aldehydes were converted into their corresponding oximes with hydroxylamine hydrochloride in the presence of potassium carbonate in methanol. Subsequent *in situ* formation of the chloro oximes in the presence of *N*-chlorosuccinimide in DMF under light exclusion, followed by coupling with β-D-thio-glucose tetraacetate in the presence of excess of di*iso*propylethylamine gave the corresponding glycosidic thiohydroximates in yields between 40–60%. The potassium *O*-sulfonates were formed by reaction with sulfur trioxide pyridine complex in the presence of excess of pyridine at 60°C and subsequent treatment with aqueous potassium hydrogen carbonate in yields between 75–87%. Finally, a global acetate deprotection in the presence of methanolic ammonia gave the desired glucosinolates containing n-butyl, 1-methylpropyl, 2-methylpropyl, n-propyl and 1-methylethyl side chains in overall yields of 33–46% over three steps. IR, NMR and MS data of the synthesized glucosinolates and reaction intermediates are given in S1 Methods.

### Preparation of desulfoglucosinolates

Desulfoglucosinolates were generated according to [61] based on procedures described by [62] and [63] (S2 Methods). Briefly, an extract of the freeze-dried plant material in 80% (v/v) methanol was loaded on DEAE-sephadex A25 columns and subjected to *Helix pomatia* sulfatase. Desulfoglucosinolates were eluted with 60% (v/v) methanol. Samples were brought to dryness and redissolved in 100 µl water. To generate an external standard, 50–100 µl of 1 mM 4-hydroxybenzyl glucosinolate were added to 1 ml 80% (v/v) methanol (without plant material) and subjected to the same steps.

### HPLC-DAD of desulfoglucosinolates for glucosinolate quantification

Separation of desulfoglucosinolates was achieved by HPLC on a 1200 Series instrument (Agilent, Waldbronn, Germany) equipped with a LiChrospher RP18 column (250 mm × 4.6 mm, 5 µm particle size; Wicom, Heppenheim, Germany) and coupled to a diode array detector. The gradients employed are provided in S11 Table, the injection volume was 5–15 µl. The eluent was monitored between 190 and 360 nm (2 nm interval) and quantification was based on peak areas at 229 nm relative to the peak area of the external standard using the following published response factors: 0.25 for indole glucosinolates [64], 0.50 for 4-hydroxybenzyl, 0.9 for benzyl and 1.0 for aliphatic glucosinolates [65]. For glucosinolates for which no response factor has been described in the literature, a response factor of 1.0 (aliphatic glucosinolates) or 0.50 (3,4-dihydroxybenzyl glucosinolate and apiosylated hydroxybenzyl glucosinolates) was assumed.

### Identification of glucosinolates

Glucosinolate identity was routinely deduced from desulfoglucosinolate identification based on comparison of HPLC retention times (S3 Table) and UV absorption spectra with those of known standards [66] (S4 Table). Identity of all desulfoglucosinolates under investigation was supported by their molecular masses determined by HPLC-MS as described below. With few exceptions, identity was confirmed for each source plant species at least once (S4 Table). For this purpose, the HPLC was run as above and coupled to a 3200 Qtrap mass spectrometer (Sciex, Framingham, MA, USA) which was run in negative mode with the enhanced MS scan type between 150 and 700 Da (see S2 Methods for details). In addition, MS^2^ spectra were recorded for all desulfoglucosinolates under investigation except desulfo-5-hexenyl glucosinolate (S4 Table) by HPLC-HRMS using a Vanquish Horizon UHPLC system coupled to an Exploris 120 HR-MS Orbitrap mass spectrometer. Compunds were separated on a Hypersil GOLD™ aQ column (150 mm x 2.1 mm, 3 µm particle size; Thermo Fisher Scientific, Darmstadt, Germany) at a flow rate of 0.25 ml/min. The gradient employed is provided in S12 Table. Detection was performed between 2.0 and 20.0 min in full scan mode between m/z 100 and 500 and with an Orbitrap resolution of 120.000. Data-dependent MS^2^ scan mode was applied from m/z 50–500 with an Orbitrap resolution of 15.000 to scan for specific m/z (S5 Table). The evaluation was performed using FreeStyle software 1.8 SP2 Version 1.8.63.0 (Thermo Fisher Scientific, Darmstadt, Germany). MS^2^ spectra of each compound were recorded once for each plant species (with few exceptions) in comparison to the respective standard (S1 Appendix).

Most compounds were also identified by the molecular mass of the intact glucosinolates, at least once for each source plant species with few exceptions (S4 Table). For this purpose, freeze-dried plant material was extracted with methanol at 94°C for 5 min followed by addition of an equal volume of water. Glass beads were added and samples were shaken vigorously in a paint shaker for 90 s followed by incubation in an ultrasonic bath for 4 min. Insoluble material was pelleted and washed once with 400 µl water. Supernatants were pooled and subjected to HPLC using a 1200 series instrument (Agilent, Waldbronn, Germany) equipped with a Hypersil GOLD™ aQ C18 column (150 mm x 2.1 mm, 3 µm particle size; Thermo Fisher Scientific, Darmstadt, Germany) coupled to a 3200 Qtrap mass spectrometer (Sciex, Framingham, MA, USA). The gradients employed are provided in S13 Table. The masses were detected in negative mode between 310 and 600 Da, with a precursor ion of 97 Da (see S2 Methods). To elucidate the identity of n-butyl, 1-methylpropyl, 2-methylpropyl, n-propyl and 1-methylethyl glucosinolates, the extracts were spiked with the synthesized isomers and analyzed as described above. For 1-(hydroxymethyl)propyl glucosinolate and 1-(hydroxymethyl)ethyl glucosinolate, the assignment as one of several possible isomers was based on their previous identification in the same species. If no standard compounds were available to prove identification in the studied species, we regarded these compounds as tentatively identified (label #; S4 Table).

Structure elucidation of two glucosinolates, namely 4-apiosyloxybenzyl glucosinolate from *H. matronalis* (‘unidentified’ according to [33]) and 2-(hydroxymethyl)butyl glucosinolate from *C. pratensis* has not been published previously. We identified these compounds tentatively in our samples based on their UV spectra, masses, MS^2^ spectra and similarity to known glucosinolates found in these species (S4 Table). As there are other possible isomers of 2-(hydroxymethyl)butyl glucosinolate, we refer to the glucosinolate as unidentified hydroxypentyl glucosinolate or isomer.

### Data analysis, statistics and graphical representation

To test for significant differences in total glucosinolate content among the studied species, data were Johnson-transformed using an online tool (https://statistikguru.de/rechner/johnson-transformation-berechnen.html) and analyzed by ANOVA with Post-hoc *t*-tests with Tukey correction using jamovi (www.jamovi.org). Variance homogeneity was tested using Levene’s test in jamovi. RStudio (64-bit) 2023.12.0 + 369 (Boston, MA, U.S.A.) was applied in R version 4.1.2. [67]. Relative abundances of glucosinolates (complete set of major and minor glucosinolates, S6 and S7 Tables) were subjected to hierarchical cluster analyses using the Ward method with Euclidean distances. Cluster analyses were performed on standardized data with RStudio using the cluster package (version 2.1.4) [68]. Violin plots and column charts were created with OriginPro, Version 2024 (OriginLab Corporation, Northampton, MA, U.S.A.). Maps with marked collection sites were created with RStudio using the ggplot2 package (version 3.4.4) and the maps package (version 3.4.0) based on the CIA World Data Bank [69–71] (https://cran.r-project.org/web/packages/mapdata/mapdata.pdf?utm_source=chatgpt.com; https://www.evl.uic.edu/pape/data/WDB/).

## Supporting information

S1 Table Profiles of major glucosinolates in leaves of plutellid host plants. Reports of a glucosinolate with the given side chain as a main glucosinolate in leaves is indicated by a letter. For each plant species, different letters stand for different profile types. Black letters refer to known profile types [26–33], while red letters represent profile types identified in the present study. Letters in parentheses indicate compounds present as major glucosinolates only in some samples. Reference [30] distinguishes between rosette and cauline leaves; only cauline leaf profile types are included in this table.
(DOCX)

S2 Table Sample table: Plant samples. (XLSX)

S3 Table Retention times of desulfoglucosinolates upon HPLC-DAD. *, only detectable by HPLC-HRMS. Gradients A, B, and C for HPLC-DAD are given in S11 Table.
(XLSX)

S4 Table Identification of glucosinolates. For the majority of glucosinolates, identification in each plant species was based on at least two of the criteria 1–5: 1, HPLC retention time and UV spectrum of the desulfoglucosinolate compared to those of known standards [66]; 2, molecular mass of the desulfoglucosinolate; 3, molecular mass of the intact glucosinolate; 4, retention time and molecular mass of the intact glucosinolate in spiking experiments with authentic standard; 5, MS^2^ spectrum in comparison with standard where available. Glucosinolate numbers refer to [5,19]. Symbol * indicates minute amounts (no quantification), symbol # indicates that authentic standards were not available (tentative identification based on criteria 2 and/or 3 and/or comparison with tentative standard). The isomer 2-hydroxy-2-methylbutyl glucosinolate (compound 29, [19]) of the unidentified hydroxypentyl glucosinolate was not present in *C. pratensis* samples based on retention time compared with a tentative standard of 2-hydroxy-2-methylbutyl glucosinolate [57]. n-Butyl glucosinolate was not detected in any sample. Glucosinolate side chain abbreviations are given as used in figures. Plant species abbreviations: Ca, *Cardamine amara*; Ci, *C. impatiens*; Ld, *Lepidium draba*; Lr, *Lunaria rediviva*; Hm, *Hesperis matronalis*; Cp, *C. pratensis*; Ds, *Descurainia sophia*.
(DOCX)

S5 Table Desulfoglucosinolate parent ions scanned for using an Exploris 120 HR-MS Orbitrap mass spectrometer. m/z of parent ions are listed for all studied desulfoglucosinolates. The complete MS2 spectra are shown in S1 Appendix. # indicates tentative identification.
(XLSX)

S6 Table Glucosinolate content (μmol per gram dry weight). (XLSX)

S7 Table Relative contents of individual glucosinolates (%). (XLSX)

S8 Table Variances of total glucosinolate content are not homogenous between species. Pairwise comparisons were done using Levene’s Homogeneity of Variances Test. P values indicating significantly different variances are printed in bold. Species abbreviations according to Fig 1.
(DOCX)

S9 Table Plant Barcode sequences. (XLSX)

S10 Table Sample table: Insect samples. (XLSX)

S11 Table Gradients for HPLC-DAD and HPLC-MS analysis of desulfoglucosinolates. (DOCX)

S12 Table Gradient for HPLC-HRMS analysis of desulfoglucosinolates. (DOCX)

S13 Table Gradient for HPLC-MS analysis of intact glucosinolates. (DOCX)

S1 Methods Syntheses. (DOCX)

S2 Methods Glucosinolate analysis. (DOCX)

S1 Appendix MS^2^ spectra of desulfoglucosinolates as detected for standards (framed in green) and in plant samples. Spectra were recorded with a collision energy of 30 V except those frame in yellow which were recorded with 10 V collision energy. For assignment of spectra to plant species, the reader is referred to the sample information line in each image. The first two letters indicate the plant species (Ca, *Cardamine amara*; Ci, *C. impatiens*; Ld, *Lepidium draba*; Lr, *Lunaria rediviva*; Hm, *Hesperis matronalis*; Cp, *C. pratensis*; Ds, *Descurainia sophia*). Primary, secondary, and tertiary alcohols could only be distinguished from one another with the aid of standards. Since theoretical differentiation options, such as dehydration peaks of varying intensities and C-C cleavage because of secondary or tertiary alcohols, did not apply to the standards, no conclusions were drawn about the structures without an existing standard. # indicates that standards were not available (tentative identification). ds, desulfo.
(DOCX)

S2 Appendix Photographical documentation of sampled populations, plants and herbarium specimen. Representative examples.
(PDF)
