# Supplementary material for: Glucosinolate diversity in seven field-collected Brassicaceae species
Source: PLoS One. 2025 Nov 13;20(11):e0336172. doi: 10.1371/journal.pone.0336172 (PMC12614607; doi:10.1371/journal.pone.0336172)
Supplement: S1 Table — Reports of a glucosinolate with the given side chain as a main glucosinolate in leaves is indicated by a letter. For each plant species, different letters stand for different profile types. Black letters refer to known profile types [26–33], while red letters represent profile types identified in the present study. Letters in parentheses indicate compounds present as major glucosinolates only in some samples. Reference [30] distinguishes between rosette and cauline leaves; only cauline leaf profile types are included in this table. (DOCX) [file pone.0336172.s001.docx]

**S1 Table: Profiles of major glucosinolates in leaves of plutellid host plants**. Reports of a glucosinolate with the given side chain as a main glucosinolate in leaves is indicated by a letter. For each plant species, different letters stand for different profile types. Black letters refer to known profile types [26-33], while red letters represent profile types identified in the present study. Letters in parentheses indicate compounds present as major glucosinolates only in some samples. Reference [30] distinguishes between rosette and cauline leaves; only cauline leaf profile types are included in this table.

|  | *C. amara* | *C. impatiens* | *L. draba* | *L. rediviva* | *H. matronalis* | *C. pratensis* | *D. sophia* |
| --- | --- | --- | --- | --- | --- | --- | --- |
| **References ⇒** | [26] a, [27] b, [28] c | [28] a, [29] b | [31] a, [32] b |  | [31] a, [33] b | [28] a,  [30] b c d e f |  |
| **⇓ Glucosinolate side chain** |  |  |  |  |  |  |  |
| 1-methylpropyl (sec-butyl) | a d |  |  | (a) |  | d f g |  |
| 2-methylpropyl (iso-butyl) | b |  |  |  |  |  |  |
| 1-(hydroxymethyl)ethyl |  |  |  |  |  | e |  |
| 1-(hydroxymethyl)propyl |  |  |  |  |  | c |  |
| 3-(hydroxymethyl)pentyl |  |  |  |  |  | b h |  |
| 2-propenyl |  |  |  |  |  |  | a |
| 3-butenyl |  | a b c |  |  |  |  |  |
| 4-pentenyl |  | b d |  |  |  |  |  |
| 4-(methylthio)butyl |  |  | (c) |  |  |  |  |
| 4-(methylsulfinyl)butyl |  |  | a b c |  |  |  |  |
| 4-(methylsulfonyl)butyl |  |  | a |  |  |  |  |
| 5-(methylsulfinyl)pentyl |  |  |  | a |  |  |  |
| ω-(methylsulfinyl)alkyl (C6-C8) |  |  |  | (a) |  |  |  |
| benzyl | a c (d) |  |  |  |  | e (g) |  |
| 4-hydroxybenzyl |  |  | a b (c) |  | a | a c d e f |  |
| 4-methoxybenzyl |  |  |  |  |  | c d |  |
| 4-apiosyloxy-3-hydroxybenzyl |  |  |  |  | b c |  |  |
| 4-hydroxyindol-3-ylmethyl |  |  |  |  | a |  |  |
