# Supplementary material for: Glucosinolate diversity in seven field-collected Brassicaceae species
Source: PLoS One. 2025 Nov 13;20(11):e0336172. doi: 10.1371/journal.pone.0336172 (PMC12614607; doi:10.1371/journal.pone.0336172)
Supplement: S4 Table — For the majority of glucosinolates, identification in each plant species was based on at least two of the criteria 1–5: 1, HPLC retention time and UV spectrum of the desulfoglucosinolate compared to those of known standards [66]; 2, molecular mass of the desulfoglucosinolate; 3, molecular mass of the intact glucosinolate; 4, retention time and molecular mass of the intact glucosinolate in spiking experiments with authentic standard; 5, MS2 spectrum in comparison with standard where available. Glucosinolate numbers refer to [5,19]. Symbol * indicates minute amounts (no quantification), symbol # indicates that authentic standards were not available (tentative identification based on criteria 2 and/or 3 and/or comparison with tentative standard). The isomer 2-hydroxy-2-methylbutyl glucosinolate (compound 29, [19]) of the unidentified hydroxypentyl glucosinolate was not present in C. pratensis samples based on retention time compared with a tentative standard of 2-hydroxy-2-methylbutyl glucosinolate [57]. n-Butyl glucosinolate was not detected in any sample. Glucosinolate side chain abbreviations are given as used in figures. Plant species abbreviations: Ca, Cardamine amara; Ci, C. impatiens; Ld, Lepidium draba; Lr, Lunaria rediviva; Hm, Hesperis matronalis; Cp, C. pratensis; Ds, Descurainia sophia. (DOCX) [file pone.0336172.s004.docx]

**S4 Table: Identification of glucosinolates.** For the majority of glucosinolates, identification in each plant species was based on at least two of the criteria 1-5: 1, HPLC retention time and UV spectrum of the desulfoglucosinolate compared to those of known standards [66]; 2, molecular mass of the desulfoglucosinolate; 3, molecular mass of the intact glucosinolate; 4, retention time and molecular mass of the intact glucosinolate in spiking experiments with authentic standard; 5, MS^2^ spectrum in comparison with standard where available. Glucosinolate numbers refer to [5] and [19]. Symbol * indicates minute amounts (no quantification), symbol # indicates that authentic standards were not available (tentative identification based on criteria 2 and/or 3 and/or comparison with tentative standard). The isomer 2-hydroxy-2-methylbutyl glucosinolate (compound 29, [19]) of tentatively identified 2-(hydroxymethyl)butyl glucosinolate was not present in *C. pratensis* samples based on retention time compared with a tentative standard of 2-hydroxy-2-methylbutyl glucosinolate [57]. n-Butyl glucosinolate was not detected in any sample. Glucosinolate side chain abbreviations are given as used in figures. Plant species abbreviations: Ca, *Cardamine amara*; Ci, *C. impatiens*; Ld, *Lepidium draba*; Lr, *Lunaria rediviva*; Hm, *Hesperis matronalis*; Cp, *C. pratensis*; Ds, *Descurainia sophia*.

| **Glucosinolate** | | **Plant species** | | | | | | |
| --- | --- | --- | --- | --- | --- | --- | --- | --- |
| **Glucosinolate side chain** | **No.** | **Ca** | **Ci** | **Ld** | **Lr** | **Hm** | **Cp** | **Ds** |
| n-propyl | **108** |  |  |  |  |  | 5* |  |
| 1-methylethyl (iso-propyl) | **56** | 12345 | 1235 |  | 125 |  | 1235 | 1345 |
| n-butyl | **13** |  |  |  |  |  |  |  |
| 2-methylpropyl (iso-butyl) | **62** | 2345 |  | 5* |  |  | 235 |  |
| 1-methylpropyl (sec-butyl) | **61** | 2345 |  | 5* | 125 |  | 2345 |  |
| 2-propenyl | **107** |  |  |  |  |  |  | 1235 |
| 3-butenyl | **12** |  | 1235 |  |  |  |  | 1235 |
| 4-pentenyl | **101** |  | 1235 |  |  |  |  |  |
| 5-hexenyl (#) | **19** |  | 3* |  |  |  |  |  |
| 1-(hydroxymethyl)ethyl (#) | **57** |  |  |  |  |  | 25 |  |
| 1-(hydroxymethyl)propyl (#) | **30** |  |  |  |  |  | 235 |  |
| unidentified hydroxypentyl or isomer (#) |  |  |  |  |  |  | 25 |  |
| 2-hydroxy-3-methylpentyl (#) | **149** |  |  |  |  |  | 15* |  |
| 3-(hydroxymethyl)pentyl | **141** |  |  |  |  |  | 1235 |  |
| 4-(methylthio)butyl (4MTB) | **84** |  |  | 1235 |  |  |  |  |
| 5-(methylthio)pentyl (5MTP) (#) | **94** |  |  | 5* | 35* |  |  |  |
| 6-(methylthio)hexyl (6MTH) (#) | **88** |  |  |  | 35* |  |  |  |
| 7-(methylthio)heptyl (7MTH) (#) | **87** |  |  |  | 35* |  |  |  |
| 8-(methylthio)octyl (8MTO) (#) | **92** |  |  |  | 35* |  |  |  |
| 4-(methylsulfinyl)butyl (4MSOB) | **64** |  |  | 1235 | 1235 |  |  |  |
| 5-(methylsulfinyl)pentyl (5MSOP) | **72** |  | 35* | 125 | 1235 |  |  |  |
| 6-(methylsulfinyl)hexyl (6MSOH) | **67** |  | 5* |  | 1235 | 3* |  |  |
| 7-(methylsulfinyl)heptyl (7MSOH) | **66** |  |  |  | 1235 |  |  |  |
| 8-(methylsulfinyl)octyl (8MSOO) | **69** |  |  |  | 1235 |  |  |  |
| 9-(methylsulfinyl)nonyl (9MSON) (#) | **68** |  |  |  | 125 |  |  |  |
| 4-(methylsulfonyl)butyl (4MSOOB) (#) | **76** |  |  | 25 |  |  |  |  |
| 5-(methylsulfonyl)pentyl (5MSOOP) (#) | **81** |  |  |  | 25 |  |  |  |
| benzyl | **11** | 1235 |  |  |  |  | 1235 | 135 |
| 4-hydroxybenzyl (4-OHbenzyl) | **23** | 125 |  | 1235 |  | 1235 |  |  |
| 3,4-dihydroxybenzyl (3,4-diOHbenzyl) (#) | **14** |  |  |  |  | 235 |  |  |
| tentative 4-apiosyloxybenzyl (#) |  |  |  |  |  | 235 |  |  |
| 4-apiosyloxy-3-hydroxybenzyl | **157** |  |  |  |  | 1235 |  |  |
| indol-3-ylmethyl (I3M) | **43** | 1235 | 1235 | 125 | 15 |  | 1235 | 5* |
| 4-hydroxy-I3M | **28** | 1235 |  |  | 1235 |  | 123 | 3* |
| 4-methoxy-I3M | **48** | 125 | 1 | 1235 | 1235 |  | 125 | 1235 |
| 1-methoxy-I3M | **47** |  |  |  |  |  | 25 |  |
