# Supplementary material for: Glucosinolate diversity in seven field-collected Brassicaceae species
Source: PLoS One. 2025 Nov 13;20(11):e0336172. doi: 10.1371/journal.pone.0336172 (PMC12614607; doi:10.1371/journal.pone.0336172)
Supplement: S8 Table — Pairwise comparisons were done using Levene’s Homogeneity of Variances Test. P values indicating significantly different variances are printed in bold. Species abbreviations according to Fig 1. (DOCX) [file pone.0336172.s008.docx]

**S8 Table: Variances of total glucosinolate content are not homogenous between species.** Pairwise comparisons were done using Levene's Homogeneity of Variances Test. P values indicating significantly different variances are printed in bold. Species abbreviations according to Fig 1.

|  |  | Ca | Ci | Cp | Ds | Hm | Ld |
| --- | --- | --- | --- | --- | --- | --- | --- |
| Ci | F | 10,5 |  |  |  |  |  |
|  | df1 | 1 |  |  |  |  |  |
|  | df2 | 62 |  |  |  |  |  |
|  | p | **<0.01** |  |  |  |  |  |
| Cp | F | 0.513 | 8.21 |  |  |  |  |
|  | df1 | 1 | 1 |  |  |  |  |
|  | df2 | 104 | 64 |  |  |  |  |
|  | p | 0.48 | **<0.01** |  |  |  |  |
| Ds | F | 4.09 | 0.594 | 3 |  |  |  |
|  | df1 | 1 | 1 | 1 |  |  |  |
|  | df2 | 56 | 16 | 58 |  |  |  |
|  | p | **<0.05** | 0.45 | 0.09 |  |  |  |
| Hm | F | 1.03 | 9.43 | 0.07 | 3.06 |  |  |
|  | df1 | 1 | 1 | 1 | 1 |  |  |
|  | df2 | 106 | 66 | 108 | 60 |  |  |
|  | p | 0.31 | **<0.01** | 0.79 | 0.09 |  |  |
| Ld | F | 23.2 | 28.8 | 31.4 | 12.8 | 36.7 |  |
|  | df1 | 1 | 1 | 1 | 1 | 1 |  |
|  | df2 | 134 | 94 | 136 | 88 | 138 |  |
|  | p | **<0.0001** | **<0.0001** | **<0.0001** | **<0.001** | **<0.0001** |  |
| Lr | F | 0.296 | 14.6 | 1.6 | 5.84 | 2.54 | 17 |
|  | df1 | 1 | 1 | 1 | 1 | 1 | 1 |
|  | df2 | 95 | 55 | 97 | 49 | 99 | 127 |
|  | p | 0.59 | **<0.001** | 0.21 | **<0.05** | 0.11 | **<0.0001** |
