# Supplementary material for: Glucosinolate diversity in seven field-collected Brassicaceae species
Source: PLoS One. 2025 Nov 13;20(11):e0336172. doi: 10.1371/journal.pone.0336172 (PMC12614607; doi:10.1371/journal.pone.0336172)
Supplement: S11 Table — (DOCX) [file pone.0336172.s011.docx]

**S11 Table: Gradients for HPLC-DAD and HPLC-MS analysis of desulfoglucosinolates**

| Gradient A | | | Gradient B | | | Gradient C | | |
| --- | --- | --- | --- | --- | --- | --- | --- | --- |
| for *C. impatiens*,  *C. pratensis*, *D. sophia, L. draba* und *L. rediviva*  extracts | | | for *H. matronalis* extracts | | | for *C. amara* extracts | | |
| time [min] | water [%] | acetonitrile[%] | time [min] | water [%] | acetonitrile[%] | time [min] | water [%] | acetonitrile[%] |
| 0 | 98.5 | 1.5 | 0 | 98.5 | 1.5 | 0 | 98.5 | 1.5 |
| 1 | 98.5 | 1.5 | 2 | 98.5 | 1.5 | 1 | 98.5 | 1.5 |
| 6 | 95.0 | 5.0 | 6 | 92.0 | 8.0 | 6 | 93.0 | 7.0 |
| 8 | 93.0 | 7.0 | 12 | 86.0 | 14.0 | 18 | 91.0 | 9.0 |
| 18 | 79.0 | 21.0 | 15 | 84.0 | 16.0 | 23 | 71.0 | 29.0 |
| 23 | 71.0 | 29.0 | 20 | 0.0 | 100.0 | 30 / 32 | 57.0 | 43.0 |
| 30 | 57.0 | 43.0 | 25 | 0.0 | 100.0 | 35 | 0.0 | 100.0 |
| 35 | 0.0 | 100.0 | 26 | 98.5 | 1.5 | 43 | 0.0 | 100.0 |
| 43 | 0.0 | 100.0 | 40 | 98.5 | 1.5 | 45 | 98.5 | 1.5 |
| 45 | 98.5 | 1.5 |  |  |  | 60 | 98.5 | 1.5 |
| 60 | 98.5 | 1.5 |  |  |  |  |  |  |
