# Supplementary material for: Glucosinolate diversity in seven field-collected Brassicaceae species
Source: PLoS One. 2025 Nov 13;20(11):e0336172. doi: 10.1371/journal.pone.0336172 (PMC12614607; doi:10.1371/journal.pone.0336172)
Supplement: S12 Table — (DOCX) [file pone.0336172.s012.docx]

**S12 Table: Gradient for HPLC-HRMS analysis of desulfoglucosinolates.** Solvent A: 0.1 % (v/v) formic acid, Solvent B: 0.1 % (v/v) formic acid in methanol.

| **Time [min]** | **% (v/v) Solvent A** | **% (v/v) Solvent B** |
| --- | --- | --- |
| 0 | 98.2 | 1.8 |
| 2 | 98.2 | 1.8 |
| 16 | 0 | 100.0 |
| 19 | 0 | 100.0 |
| 21 | 98.2 | 1.8 |
| 32 | 98.2 | 1.8 |
