# Supplementary material for: Glucosinolate diversity in seven field-collected Brassicaceae species
Source: PLoS One. 2025 Nov 13;20(11):e0336172. doi: 10.1371/journal.pone.0336172 (PMC12614607; doi:10.1371/journal.pone.0336172)
Supplement: S1 Methods — (DOCX) [file pone.0336172.s014.docx]

# S1 Methods: Syntheses

# 1. General methods

Unless otherwise noted, all reagents were purchased from commercial suppliers and used without further purification. (*N,N*-Dimethylformamid (DMF): *Acros Organics*, puriss., extra dry, over molecular sieve (water ≤ 0.005%), Ethanol (EtOH): *Acros Organics*, puriss., absolut, extra dry (water ≤ 0.005%), Pyridin (Pyr): *Acros Organics*, puriss., extra dry, over molecular sieve (water ≤ 0.005%), Dimethylsulfoxid (DMSO): *Acros Organics*, puriss., extra dry, over molecular sieve (water ≤ 0.005%), Methanol (MeOH): *Acros Organics*, puriss., extra dry (water ≤ 0.005%)).

Moisture-sensitive reactions were performed under argon atmosphere in dried glassware. Dry dichloromethane, diethyl ether, toluene, and tetrahydrofuran for moisture-sensitive reactions have been taken from a MB-SPS-800 (MBraun) solvent purifications system and stored under argon. All solvents used for workup and purification were of HPLC grade. Reactions were monitored by TLC, LCMS, or NMR.

Solutions of compounds in organic solvents were concentrated using rotary evaporators at a water bath temperature of max. 30°C. Solvent residues were removed in a high vacuum at a pressure of approximately 10^‑2^ mbar. Unless otherwise noted solvents were degassed either by a continuous argon flow over a minimum of 15 min or using the freeze-pump-thaw (FTP) technique (Branda, N. (2019) *JoVE Sci. Educ. Database. Org. Chem.*)

**Flash chromatography** (Still et al. (1978) *J. Org. Chem.* 43, 2923-2925) was done using appropriate glass columns filled with silica gel (Merck Millipore, Geduran^®^ Si60, 1.11567.9025, 40–63 µm) or using the Biotage Select® chromatography system with a DAD detector and cartridges packed with silica gel (Merck Millipore, Geduran^®^ Si60, 1.11567.9025, 40-63 µm) using a Cartridger® C-670 from the company Büchi.

**Thin-layer chromatography (TLC)** was performed on pre-coated glass plates (Merck TLC Silicagel 60 F_254_, 1.15341.0001, 2.5x7.5 cm) and components were visualized by observation under UV light (λ = 254 nm [UV^254^] or λ = 366 nm [UV^366^]) or visible light, treatment of developed plates in an iodine chamber or by treating the plates with TLC staining solutions (for preparation see list below) followed by heating. Eluent or eluent mixtures used are reported in parentheses.

***CAM staining solution* [CAM]:** 1 g Ce(IV)(SO_4_)_2_, 2.5 g (NH_4_)_6_Mo_4_O_7_ in 100 mL 10% H_2_SO_4_

***KMnO_4_ staining solution* [KMnO_4_]:** 1.5 g KMnO_4_, 10 g K_2_CO_3_, and 1.25 mL 10% NaOH in 200 mL H_2_O.

**Preparative thin-phase chromatography** was performed on pre-coated glass plates (Merck TLC Silica gel 60 F_254_, 1.05715.0001, 20x20 cm, max. 10-15 mg/plate and Analtech Uniplate Silica gel GF Z51305-9, 20x20 cm x 2 mm, max 100-150 mg/plate). Eluent or eluent mixtures used and the number of developments are reported in parentheses. Compounds were visualized by observation under UV light (λ = 254 or 366 nm). Compounds containing silica gel fractions were scratched from the plate with a scalpel, crushed into small pieces, and compounds were eluted with appropriate solvent mixtures.

**NMR** spectra were recorded on a Bruker Avance NEO 600 MHz spectrometer and an Oxford 800 magnet, Bruker Avance III HD spectrometer with cryoprobe systems at 293.15 K. ^1^H NMR spectra were recorded at 600 MHz and 800 MHz. ^13^C NMR spectra were recorded at 151 MHz and 201 MHz. Chemical shifts are referenced relative to residual solvent signals. Data are presented as follows: chemical shift, multiplicity (s = singlet, d = doublet, t = triplet, q = quartet, quint = quintet, sext = sextet, sept = septet, m = multiplet etc), and coupling constants in hertz (Hz), followed by the number of hydrogen atoms. The software MestReNova (Version 9.0.1-13254) from MestreLab Research S.L. was used to process the raw data.

**IR** data were recorded using a Bruker Invenio R instrument in transmission mode (with the ATR technique).

**Low-resolution mass spectrometry (LRMS)** data were recorded using a Waters™ LCMS system consisting of an Acquity Arc HPLC, an ACQ Arc Column Heater/Cooler (0-60°C), 2489 UV/Vis Detector (200-600 nm) and an Acquity QDa Mass Detector (ESI +/-, Quadropole, max.1250 Da, cone voltage: 20.0 V, probe temperature 600°C) equipped with a MS/UV Splitter (10:1) and a column switch with three analytical RP columns (1. Waters™ C18 RP Column XBridge BEH C18 130Å, 2.5 µm, 2.1 x 50 mm with Guard Column XBridge BEH C18 V-Gd Cart 2.5 μ, 2.1 x 5 mm, 2. Waters™ C8 RP Column XBridge PRM BEH 130Å C8 2.5 μm 2.1 x 50 mm with Guard Column XBridge BEH C18 V-Gd Cart 2.5 μm, 2.1 x 5 mm, 3. Waters™ C18 RP Column XBridge BEH C18 130Å, 3.5 µm, 2.1 x 150 mm with Guard Column XBridge BEH C18 V-Gd Cart 2.5 μ, 2.1 x 5 mm); Data were analyzed with the Software MassLynx 4.1.

**High-resolution mass spectrometry (HRMS)** was obtained from:

1. an Agilent 1290 Infinity LC system equipped with an autosampler in tandem with an Agilent 6520 Accurate Mass Q-TOF LC/MS.
2. a linear iontrap coupled with orbitrap mass analyzer LTQ-Orbitrap Velos from Thermo Fisher Scientific (Resolution: 100000 FWHM (at m/z = 400 amu), Scan: 130-2000 amu (resulting in acquisition times of 1.6 sec per cycle)). Electrospray measurements were performed in direct infusion mode using a custom microspray device mounted on a Proxeon nanospray ion source. The microspray device allows for the sample infusion through a stainless-steel capillary (90 μm I. D.). Accurate mass measurements in the orbitrap were performed using the lock mass option of the instrument control software using the cation of tetradecyltrimethylammonium bromide (256.29988 amu) as internal mass reference. (Sample concentration: approx. 50 μg/mL. Solvent: MeOH spiked with 0.1 mg/mL tetradecyltrimethylammonium bromide (unless otherwise stated). Flow: approx. 1 μL/min. Typical spray voltage pos. mode: 2.3 - 2.8 kV. Typical spray voltage neg. mode: 1.7 - 2.5 kV).

# 2. Syntheses and product characterization

## (2*R*,3*R*,4*S*,5*R*,6*S*)-2-(acetoxymethyl)-6-(((*Z*)-1-(hydroxyimino)-2-methylpropyl)thio)tetrahydro-2H-pyran-3,4,5-triyl triacetate (7)

To a solution of hydroxylamine hydrochloride (1.01 g, 14.56 mmol, 1.05 eq) and potassium carbonate (2.01 g, 14.56 mmol, 1.05 eq) in MeOH (8 mL) was added a solution of aldehyde **1** (1.27 mL, 1 g, 13.87 mmol, 1.0 eq) in MeOH (2 mL) dropwise. The reaction resulting mixture was stirred for 6 h at 21°C. After the completion of the reaction was indicated by TLC, the reaction mixture was filtrated and the white precipitate was washed with diethyl ether (4×20 mL). The filtrates were combined and evaporated under reduced pressure to afford the desired oxime (1.16 g, 13.31 mmol, 96%) as a 1:1-mixture of (*E*)- and (*Z*)-isomers and as a white amorphous solid which was used directly without further purification.

*N*-Chlorosuccinimide (161 mg, 1.21 mmol, 1.05 eq) was added portion wise to a solution of oxime (100 mg, 1.15 mmol, 1.0 eq) in dry DMF (1 mL) under argon atmosphere and the resulting mixture was stirred under light exclusion for 1 h at 23°C. The reaction mixture was thereafter diluted with H_2_O (30 mL) and extracted with CH_2_Cl_2_ (7×15 mL). The combined organic layers were washed with brine (3×20 mL), dried over anhydrous Na_2_SO_4_ and the solvent removed under reduced pressure to afford the desired chloro-oxime which was used directly without further purification.

DiPEA (1.2 mL, 880 mg, 6.89 mmol, 6.0 eq) was added to a solution of 1-Thio-*β*-D-glucose tetraacetate (**6**, 418 mg, 1.15 mmol, 1.0 eq) in dry THF (3.0 mL) and the resulting mixture was added dropwise to a solution of chloro-oxime (195 mg, 1.61 mol, 1.4 eq) in dry THF (3.0 mL) under argon atmosphere. The reaction mixture was stirred for 16 h at 23°C, before it was poured into ice cold H_2_O (20 mL) and extracted with CH_2_Cl_2_ (5×20 mL). The combined organic layers were washed with saturated solution of NH_4_Cl (3×20 mL), brine (3×20 mL), dried over anhydrous Na_2_SO_4_, filtered and the solvent was removed under reduced pressure. The resulting residue was purified by flash chromatography using the Biotage Select® chromatography system with a DAD detector and a cartridge packed with silica gel (CH_2_Cl_2_:MeOH/0→5%) to yield compound **7** (284 mg, 0.63 mmol, 55%) as a white amorphous solid.

**TLC** (CH_2_Cl_2_:MeOH/20:1), R*_f_* = 0.6 [UV^254^, KMnO_4_]. **IR** (ATR) [cm^-1^]: 3434, 2972, 2942, 2362, 2337, 1748, 1368, 1218, 1088, 1039, 946, 915, 600. **HRMS** (ESI) [m/z]: calculated for [C_18_H_27_NNaO_10_S]^+^, 472.1248; found 472.1304. **^1^H-NMR** (600 MHz, CD_3_OD) δ [ppm]: 5.35 (t, *J* = 9.3 Hz, 1H), 5.31 (d, *J* = 10.1 Hz, 1H), 5.03 (t, *J* = 9.8 Hz, 1H), 4.97 (dd, *J* = 10.1, 9.2 Hz, 1H), 4.21 (dd, *J* = 12.4, 5.6 Hz, 1H), 4.11 (dd, *J* = 12.4, 2.3 Hz, 1H), 3.96 (ddd, *J* = 10.1, 5.5, 2.3 Hz, 1H), 2.80 (hept, *J* = 6.8 Hz, 1H), 2.05 (s, 3H), 2.03 (s, 3H), 2.02 (s, 3H), 1.98 (s, 3H), 1.22 (d, *J* = 6.7 Hz, 3H), 1.21 (d, *J* = 6.8 Hz, 3H). **^13^C-NMR** (151 MHz, CD_3_OD) δ [ppm]: 172.23, 171.56, 171.22, 171.01, 155.60, 80.89, 76.67, 75.21, 71.90, 69.68, 63.42, 33.52, 22.09, 21.69, 20.65, 20.61, 20.53.

## (2*R*,3*R*,4*S*,5*R*,6*S*)-2-(acetoxymethyl)-6-(((*Z*)-1-(hydroxyimino)-3-methylbutyl)thio)tetrahydro-2H-pyran-3,4,5-triyl triacetate (8)

To a solution of hydroxylamine hydrochloride (847 mg, 12.19 mmol, 1.05 eq) and potassium carbonate (1.68 g, 12.19 mmol, 1.05 eq) in MeOH (8 mL) was added a solution of aldehyde **2** (1.25 mL, 1 g, 11.61 mmol, 1.0 eq) in MeOH (2 mL) dropwise and the reaction mixture was stirred for 6 h at 21°C. After the completion of the reaction was indicated by TLC, the reaction mixture was filtrated and the white precipitate was washed with diethyl ether (4×20 mL). The combined filtrates were evaporated under reduced pressure to afford the desired oxime (1.01 g, 9.99 mmol, 86%) as a 1:1-mixture of (*E*)- and (*Z*)-isomers and as a white amorphous solid which was used directly without further purification.

*N*-Chlorosuccinimide (139 mg, 1.04 mmol, 1.05 eq) was added portionwise to a solution of oxime (100 mg, 0.99 mmol, 1.0 eq) in dry DMF (1 mL) under argon atmosphere and the resulting mixture was stirred under light exclusion for 1 h at 23°C. The reaction mixture was diluted with H_2_O (30 mL) and extracted with CH_2_Cl_2_ (7×15 mL). The combined organic layers were washed with brine (3×20 mL), dried over anhydrous Na_2_SO_4_ and the solvent was removed under reduced pressure. The resulting chloro-oxime was used directly without further purification.

DiPEA (1.03 mL, 767 mg, 5.93 mmol, 6.0 eq) was added to a solution of 1-Thio-*β*-D-glucose tetraacetate (**6**, 360 mg, 0.99 mmol, 1.0 eq) in dry THF (3.0 mL) and the resulting mixture was added dropwise to a solution of chloro-oxime (188 mg, 1.38 mol, 1.4 eq) in dry THF (3.0 mL) under argon atmosphere. The reaction mixture was stirred for 16 h at 23°C, before it was poured into ice cold H_2_O (20 mL) and extracted with CH_2_Cl_2_ (5×20 mL). The combined organic layers were washed with saturated solution of NH_4_Cl (3×20 mL), brine (3×20 mL), dried over anhydrous Na_2_SO_4_, filtered and the solvent was removed under reduced pressure. The resulting residue was purified by flash chromatography using the Biotage Select® chromatography system with a DAD detector and a cartridge packed with silica gel (CH_2_Cl_2_:MeOH/0→5%) to yield compound **8** (276 mg, 0.60 mmol, 60%) as a white amorphous solid.

**TLC** (CH_2_Cl_2_:MeOH/20:1), R*_f_* = 0.57 [UV^254^, KMnO_4_]. **IR** (ATR) [cm^-1^]: 3421, 2959, 2872, 2362, 2339, 1750, 1369, 1223, 1085, 1041, 963, 915, 600. **HRMS** (ESI) [m/z]: calculated for [C_19_H_29_NNaO_10_S]^+^, 486.1404; found 486.1459. **^1^H-NMR** (600 MHz, CD_3_OD) δ [ppm]: 5.38 (t, *J* = 9.3 Hz, 1H), 5.25 (d, *J* = 10.1 Hz, 1H), 5.04 (t, *J* = 9.8 Hz, 1H), 5.02 – 4.95 (m, 1H), 4.22 (dd, *J* = 12.4, 5.5 Hz, 1H), 4.13 (dd, *J* = 12.4, 2.3 Hz, 1H), 3.97 (ddd, *J* = 10.1, 5.5, 2.3 Hz, 1H), 2.45 – 2.33 (m, 2H), 2.06 (s, 3H), 2.03 (s, 3H), 2.02 (s, 3H), 1.98 (s, 3H), 0.99 (d, *J* = 6.6 Hz, 3H), 0.96 (d, *J* = 6.6 Hz, 3H). **^13^C-NMR** (151 MHz, CD_3_OD) δ [ppm]: 172.20, 171.55, 171.22, 170.96, 151.40, 80.85, 76.78, 75.13, 71.78, 69.65, 63.47, 42.08, 27.84, 22.82, 22.51, 20.69, 20.59, 20.54.

## (2*R*,3*R*,4*S*,5*R*,6*S*)-2-(acetoxymethyl)-6-(((*Z*)-1-(hydroxyimino)-2-methylbutyl)thio)tetrahydro-2H-pyran-3,4,5-triyl triacetate (9)

To a solution of hydroxylamine hydrochloride (847 mg, 12.19 mmol, 1.05 eq) and potassium carbonate (1.68 g, 12.19 mmol, 1.05 eq) in MeOH (8 mL) was added dropwise a solution of aldehyde **3** (1.25 mL, 1 g, 11.61 mmol, 1.0 eq) in MeOH (2 mL). The reaction mixture was stirred for 6 h at 21°C. After the completion of the reaction was indicated by TLC, the reaction mixture was filtrated, and the white precipitate was washed with diethyl ether (4×20 mL). The filtrates were combined and evaporated under reduced pressure to afford the desired oxime (999.1 mg, 9.88 mmol, 85%) as a 1:1-mixture of (*E*)- and (*Z*)-isomers and as a white amorphous solid which was used directly without further purification.

*N*-Chlorosuccinimide (139 mg, 1.04 mmol, 1.05 eq) was added portionwise to a solution of oxime (100 mg, 0.99 mmol, 1.0 eq) in dry DMF (1 mL) under argon atmosphere. The reaction mixture was stirred under light exclusion for 1 h at 23°C. The mixture was diluted with H_2_O (30 mL) and extracted with CH_2_Cl_2_ (7×15 mL). The combined organic layers were washed with brine (3×20 mL), dried over Na_2_SO_4_ and the solvent removed under reduced pressure. The resulting chloro-oxime was used directly without further purification.

DiPEA (1.03 mL, 767 mg, 5.93 mmol, 6.0 eq) was added to a solution of 1-Thio-*β*-D-glucose tetraacetate (**6**, 360 mg, 0.99 mmol, 1.0 eq) in dry THF (3.0 mL) and the resulting mixture was added dropwise to a solution of chloro-oxime (188 mg, 1.38 mol, 1.4 eq) in dry THF (3.0 mL) under argon atmosphere. The reaction mixture was stirred for 16 h at 23°C, before it was poured into ice cold H_2_O (20 mL) and extracted with CH_2_Cl_2_ (5×20 mL). The combined organic layers were washed with saturated solution of NH_4_Cl (3×20 mL), brine (3×20 mL), dried over anhydrous Na_2_SO_4_, filtered and the solvent was removed under reduced pressure. The resulting residue was purified by flash chromatography using the Biotage Select® chromatography system with a DAD detector and a cartridge packed with silica gel (CH_2_Cl_2_:MeOH/0→5%) to yield compound **9** (206 mg, 0.44 mmol, 45%) as a white amorphous solid.

**TLC** (CH_2_Cl_2_:MeOH/20:1), R*_f_* = 0.56 [UV^254^, KMnO_4_]. **IR** (ATR) [cm^-1^]: 3447, 2966, 2941, 2878, 2362, 2337, 1751, 1369, 1223, 1088, 1041, 914, 600. **HRMS** (ESI) [m/z]: calculated for [C_19_H_29_NNaO_10_S]^+^, 486.1404; found 486.1542. **^1^H-NMR** (600 MHz, CDCl_3_) δ [ppm]: 5.28 – 5.23 (m, 2H), 5.19 (t, *J* = 9.5 Hz, 1H), 5.11 – 5.04 (m, 2H), 4.22 (dd, *J* = 12.6, 2.4 Hz, 1H), 4.13 (dd, *J* = 12.5, 2.3 Hz, 1H), 2.07 (s, 3H), 2.07 (s, 3H), 2.02 (s, 3H), 2.01 (s, 3H), 1.24 – 1.18 (m, 2H), 1.16 – 1.11 (m, 1H), 0.99 – 0.83 (m, 6H). **^13^C-NMR** (151 MHz, CDCl_3_) δ [ppm]: 170.72, 170.13, 169.35, 169.30, 169.18, 87.23, 76.15, 73.85, 69.70, 67.83, 61.54, 40.71, 28.10, 26.85, 20.56, 18.44, 11.69.

## (2*R*,3*R*,4*S*,5*R*,6*S*)-2-(acetoxymethyl)-6-(((*Z*)-1-(hydroxyimino)butyl)thio)-tetrahydro-2H-pyran-3,4,5-triyl triacetate (10)

To a solution of hydroxylamine hydrochloride (1.01 g, 14.56 mmol, 1.05 eq) and potassium carbonate (2.01 g, 14.56 mmol, 1.05 eq) in MeOH (8 mL) was added dropwise a solution of aldehyde **4** (1.27 mL, 1 g, 13.87 mmol, 1.0 eq) in MeOH (2 mL) and the reaction mixture was stirred for 6 h at 21°C. After the completion of the reaction was indicated by TLC, the reaction mixture was filtrated and the white precipitate was washed with diethyl ether (5×20 mL). The combined filtrates were evaporated under reduced pressure to afford the desired oxime (1 g, 11.48 mmol, 83%) as a 1:1-mixture of (*E*)- and (*Z*)-isomers and as a white amorphous solid which was used directly without further purification.

*N*-Chlorosuccinimide (161 mg, 1.21 mmol, 1.05 eq) was added portionwise to a solution of oxime (100 mg, 1.15 mmol, 1.0 eq) in dry DMF (1 mL) under argon atmosphere. The reaction mixture was stirred under light exclusion for 1 h at 23°C and thereafter diluted with H_2_O (50 mL) and extracted with CH_2_Cl_2_ (7×15 mL). The combined organic layers were washed with brine (3×20 mL), dried over anhydrous Na_2_SO_4_ and the solvent was removed under reduced pressure. The resulting chloro-oxime was used directly without further purification.

DiPEA (1.2 mL, 880 mg, 6.8871 mmol, 6.0 eq) was added to solution of 1-Thio-*β*-D-glucose tetraacetate (**6**, 418 mg, 1.15 mmol, 1.0 eq) in dry THF (3.0 mL) and the mixture was added dropwise to a solution of chloro-oxime (195 mg, 1.61 mol, 1.4 eq) in dry THF (3.0 mL) under argon atmosphere. The reaction mixture was stirred for 16 h at 23°C, before it was poured into ice-cold H_2_O (20 mL) and extracted with CH_2_Cl_2_ (3×20 mL). The combined organic layers were washed with a saturated solution of NH_4_Cl (3×20 mL), brine (3×20 mL), dried over anhydrous Na_2_SO_4_, filtered and the solvent was removed under reduced pressure. The resulting residue was purified by flash chromatography using the Biotage Select® chromatography system with a DAD detector and a cartridge packed with silica gel (CH_2_Cl_2_:MeOH/0→5%) to yield compound **10** (206 mg, 0.46 mmol, 40%) as a white amorphous solid.

**TLC** (CH_2_Cl_2_:MeOH/20:1), R*_f_* = 0.58 [UV^254^, KMnO_4_]. **IR** (ATR) [cm^-1^]: 3473, 2962, 2942, 2877, 2362, 2338, 1743, 1368, 1210, 1036, 912, 822, 599. **HRMS** (ESI) [m/z]: calculated for [C_18_H_27_NNaO_10_S]^+^, 472.1248; found 472.1334. **^1^H-NMR** (600 MHz, CD_3_OD) δ [ppm]: 5.38 (t, *J* = 9.3 Hz, 1H), 5.26 (d, *J* = 10.1 Hz, 1H), 5.04 (t, *J* = 9.8 Hz, 1H), 4.98 (t, *J* = 9.7 Hz, 1H), 4.22 (dd, *J* = 12.4, 5.6 Hz, 1H), 4.13 (dd, *J* = 12.4, 2.3 Hz, 1H), 3.99 (ddd, *J* = 10.2, 5.5, 2.3 Hz, 1H), 2.55 – 2.50 (m, 2H), 2.06 (s, 3H), 2.03 (s, 3H), 2.02 (s, 3H), 1.98 (s, 3H), 1.73 – 1.65 (m, 2H), 0.99 (t, *J* = 7.4 Hz, 3H). **^13^C-NMR** (151 MHz, CD_3_OD) δ [ppm]: 172.22, 171.56, 171.23, 170.98, 152.22, 80.67, 76.69, 75.15, 71.70, 69.67, 63.45, 35.07, 21.86, 20.64, 20.58, 20.53, 13.92.

## (2*R*,3*R*,4*S*,5*R*,6*S*)-2-(acetoxymethyl)-6-(((*Z)*-1-(hydroxyimino)pentyl)thio)-tetrahydro-2H-pyran-3,4,5-triyl triacetate (11)

To a solution of hydroxylamine hydrochloride (847 mg, 12.19 mmol, 1.05 eq) and potassium carbonate (1.68 g, 12.19 mmol, 1.05 eq) in MeOH (8 mL) was added dropwise a solution of aldehyde **5** (1.25 mL, 1 g, 11.61 mmol, 1.0 eq) in MeOH (2 mL) and the reaction mixture was stirred for 6 h at 21°C. After the completion of the reaction was indicated by TLC, the resulting crude product was filtrated and the white precipitate was washed with diethyl ether (4×20 mL). The combined filtrates were evaporated under reduced pressure to afford the desired oxime (993.1 mg, 9.82 mmol, 85%) as a 1:1-mixture of (*E*)- and (*Z*)-isomers and as a white amorphous solid which was used directly without further purification.

*N*-Chlorosuccinimide (139 mg, 1.04 mmol, 1.05 eq) was added portionwise to a solution of oxime (100 mg, 0.99 mmol, 1.0 eq) in dry DMF (1 mL) under argon atmosphere. The reaction mixture was stirred under light exclusion for 1 h at 23°C before it was diluted with H_2_O (30 mL) and extracted with CH_2_Cl_2_ (7×15 mL). The combined organic layers were washed with brine (3×20 mL), dried over anhydrous Na_2_SO_4_ and the solvent removed under reduced pressure. The resulting chloro-oxime was used directly without further purification.

DiPEA (1.03 mL, 767 mg, 5.93 mmol, 6.0 eq) was added to a solution of 1-Thio-*β*-D-glucose tetraacetate (**6**, 360 mg, 0.99 mmol, 1.0 eq) in dry THF (3.0 mL) and the mixture was added dropwise to a solution of chloro-oxime (188 mg, 1.38 mol, 1.4 eq) in dry THF (3.0 mL) under argon atmosphere. The reaction mixture was stirred for 16 h at 23°C, before it was poured into ice-cold H_2_O (20 mL) and extracted with CH_2_Cl_2_ (5×20 mL). The combined organic layers were washed with a saturated solution of NH_4_Cl (3×20 mL), brine (3×20 mL), dried over anhydrous Na_2_SO_4_, filtered and the solvent was removed under reduced pressure. The resulting residue was purified by flash chromatography using the Biotage Select® chromatography system with a DAD detector and a cartridge packed with silica gel (CH_2_Cl_2_:MeOH/0→5%) to yield compound **11** (256 mg, 0.55 mmol, 56%) as a white amorphous solid.

**TLC** (CH_2_Cl_2_:MeOH/20:1), R*_f_* = 0.55 [UV^254^, KMnO_4_]. **IR** (ATR) [cm^-1^]: 3367, 2960, 2939, 2875, 2362, 2338, 2256, 1746, 1367, 1211, 1088, 1034, 910, 729, 598. **HRMS** (ESI) [m/z]: calculated for [C_19_H_29_NNaO_10_S]^+^, 486.1404; found 486.1465. **^1^H-NMR** (600 MHz, CD_3_OD) δ [ppm]: 5.38 (t, *J* = 9.3 Hz, 1H), 5.26 (d, *J* = 10.2 Hz, 1H), 5.05 (dd, *J* = 10.1, 9.4 Hz, 1H), 4.98 (dd, *J* = 10.1, 9.2 Hz, 1H), 4.23 (dd, *J* = 12.4, 5.4 Hz, 1H), 4.13 (dd, *J* = 12.4, 2.3 Hz, 1H), 3.98 (ddd, *J* = 10.1, 5.4, 2.3 Hz, 1H), 2.58 – 2.50 (m, 2H), 2.05 (s, 3H), 2.03 (s, 3H), 2.02 (s, 3H), 1.98 (s, 3H), 1.67 – 1.61 (m, 2H), 1.41 (h, *J* = 7.4 Hz, 2H), 0.96 (t, *J* = 7.4 Hz, 3H). **^13^C-NMR** (151 MHz, CD_3_OD) δ [ppm]: 172.20, 171.55, 171.22, 170.96, 152.34, 80.67, 76.69, 75.13, 71.71, 69.64, 63.43, 32.90, 30.70, 23.21, 20.66, 20.59, 20.54, 14.23.

## Potassium ((*Z*)-2-methyl-1-(((2*S*,3*R*,4S,5*R*,6*R*)-3,4,5-triacetoxy-6-(acetoxy-methyl)tetrahydro-2H-pyran-2-yl)thio)propylidene)amino sulfate (12)

Sulfur trioxide pyridinium complex (283 mg, 1.78 mmol, 5.0 eq) and pyridine (0.29 mL, 282 mg, 3.56 mmol, 10.0 eq) were added subsequently to a solution of compound **7** (160 mg, 0.36 mmol, 1.0 eq) in CH_2_Cl_2_ (1.0 mL) at 23°C under an argon atmosphere and the mixture was stirred at 60°C for 6 h. Afterward, the reaction mixture was cooled down to 23°C and an aqueous solution of KHCO_3_ (2.85 mL, 5.70 mmol, 16.0 eq, 2 M) was added slowly under stirring. The mixture was stirred vigorously for 30 min, before it was concentrated under reduced pressure. The resulting residue was purified by flash chromatography through silica gel (dry load, CH_2_Cl_2_:MeOH 10/:0→5:1) yielding GSL **12** as a white amorphous solid (176 mg, 0.31 mmol, 87%).

**TLC** (CH_2_Cl_2_:MeOH/10:1), R*_f_* = 0.27 [UV^254^, KMnO_4_]. **IR** (ATR) [cm^-1^]: 2973, 2941, 2362, 2338, 1746, 1710, 1436, 1367, 1213, 1038, 894, 871, 777, 696, 626, 599, 531, 489. **HRMS** (ESI) [m/z]: calculated for [C_18_H_26_NO_13_S_2_]^-^, 528.0851; found 528.0876. **^1^H-NMR** (600 MHz, CD_3_OD) δ [ppm]: 5.41 (d, *J* = 10.2 Hz, 1H), 5.37 (t, *J* = 9.3 Hz, 1H), 5.07 – 5.02 (m, 1H), 4.98 (dd, *J* = 10.1, 9.2 Hz, 1H), 4.22 (dd, *J* = 12.5, 5.3 Hz, 1H), 4.15 (dd, *J* = 12.4, 2.3 Hz, 1H), 4.03 (ddd, *J* = 10.2, 5.3, 2.3 Hz, 1H), 2.94 (hept, *J* = 6.8 Hz, 1H), 2.06 (s, 3H), 2.04 (s, 3H), 2.03 (s, 3H), 1.98 (s, 3H), 1.29 (t, *J* = 7.0 Hz, 6H). **^13^C-NMR** (151 MHz, CD_3_OD) δ [ppm]: 172.25, 171.43, 171.23, 170.94, 162.90, 81.03, 76.58, 74.99, 71.52, 69.39, 63.27, 34.20, 21.92, 21.46, 20.68, 20.57, 20.53.

## Potassium ((*Z*)-3-methyl-1-(((2*S*,3*R*,4*S*,5*R*,6*R*)-3,4,5-triacetoxy-6-(acetoxy-methyl)tetrahydro-2H-pyran-2-yl)thio)butylidene)amino sulfate (13)

Sulfur trioxide pyridinium complex (172 mg, 1.08 mmol, 5.0 eq) and pyridine (0.17 mL, 171 mg, 2.16 mmol, 10.0 eq) were added subsequently to a solution of compound **8** (100 mg, 0.22 mmol, 1.0 eq) in CH_2_Cl_2_ (1.0 mL) at 23°C under an argon atmosphere and the mixture was stirred at 60°C for 4 h. Afterwards, the reaction mixture was cooled down to 23°C and an aqueous solution of KHCO_3_ (1.73 mL, 3.45 mmol, 16.0 eq, 2 M) was added slowly under stirring. The resulting mixture was stirred vigorously for 30 min, before it was concentrated under reduced pressure. The resulting residue was purified by flash chromatography through silica gel (dry load, CH_2_Cl_2_:MeOH 10/:0→5:1) yielding GSL **13** as a white amorphous solid (100 mg, 0.17 mmol, 80%).

**TLC** (CH_2_Cl_2_:MeOH/10:1), R*_f_* = 0.23 [UV^254^, KMnO_4_]. **IR** (ATR) [cm^-1^]: 2961, 2874, 2362, 2337, 1751, 1373, 1229, 1060, 911, 797, 645, 577. **HRMS** (ESI) [m/z]: calculated for [C_19_H_28_NO_13_S_2_]^-^, 542.1008; found 542.1027. **^1^H-NMR** (600 MHz, CD_3_OD) δ [ppm]: 5.39 (t, *J* = 9.3 Hz, 1H), 5.35 (d, *J* = 10.2 Hz, 1H), 5.05 (t, *J* = 9.8 Hz, 1H), 4.97 (dd, *J* = 10.2, 9.2 Hz, 1H), 4.22 (dd, *J* = 12.5, 5.4 Hz, 1H), 4.14 (dd, *J* = 12.5, 2.2 Hz, 1H), 4.00 (ddd, *J* = 10.1, 5.4, 2.2 Hz, 1H), 2.50 (dd, *J* = 7.1, 2.1 Hz, 2H), 2.16 (dt, *J* = 13.5, 6.8 Hz, 1H), 2.06 (s, 3H), 2.02 (d, *J* = 3.6 Hz, 6H), 1.97 (s, 3H), 1.03 (d, *J* = 6.7 Hz, 3H), 1.01 (d, *J* = 6.6 Hz, 3H). **^13^C-NMR** (151 MHz, CD_3_OD) δ [ppm]: 172.22, 171.52, 171.21, 170.90, 158.32, 81.12, 76.86, 75.08, 71.53, 69.54, 63.40, 42.36, 27.89, 22.78, 22.58, 20.69, 20.52.

## Potassium ((*Z*)-2-methyl-1-(((2*S*,3*R*,4*S*,5*R*,6*R*)-3,4,5-triacetoxy-6-(acetoxy-methyl)tetrahydro-2H-pyran-2-yl)thio)butylidene)amino sulfate (14)

Sulfur trioxide pyridinium complex (266 mg, 1.67 mmol, 5.0 eq) and pyridine (0.27 mL, 265 mg, 3.34 mmol, 10.0 eq) were added subsequently to a solution of compound **9** (155 mg, 0.33 mmol, 1.0 eq) in CH_2_Cl_2_ (1.0 mL) at 23°C under an argon atmosphere and the mixture was stirred at 60°C for 4 h. Afterwards, the reaction mixture was cooled down to 23°C and an aqueous solution of KHCO_3_ (2.68 mL, 5.35 mmol, 16.0 eq, 2 M) was added slowly under stirring. The mixture was stirred vigorously for 30 min, before it was concentrated under reduced pressure. The resulting residue was purified by flash chromatography through silica gel (dry load, CH_2_Cl_2_:MeOH 10/:0→5:1) yielding GSL **14** as a white amorphous solid (146 mg, 0.25 mmol, 75%).

**TLC** (CH_2_Cl_2_:MeOH/10:1), R*_f_* = 0.25 [UV^254^, KMnO_4_]. **IR** (ATR) [cm^-1^]: 2967, 2938, 2878, 2362, 2339, 1748, 1368, 1215, 1037, 942, 913, 823, 600. **HRMS** (ESI) [m/z]: calculated for [C_19_H_28_NO_13_S_2_]^-^, 542.1008; found 542.1025. **^1^H-NMR** (600 MHz, CDCl_3_) δ [ppm]: 5.44 – 5.38 (m, 1H), 5.30 – 5.22 (m, 2H), 5.10 – 5.06 (m, 2H), 4.25 – 4.17 (m, 2H), 2.07 (s, 6H), 2.03 (s, 3H), 2.01 (s, 3H), 1.28 – 1.22 (m, 2H), 1.20 – 1.16 (m, 1H), 0.99 – 0.89 (m, 6H). **^13^C-NMR** (151 MHz, CDCl_3_) δ [ppm]: 170.72, 170.48, 170.06, 169.43, 169.31, 81.30, 80.49, 74.05, 73.46, 67.79, 61.89, 40.70, 28.21, 20.55, 18.39, 11.57.

## Potassium ((*Z*)-1-(((2*S*,3*R*,4*S*,5*R*,6*R*)-3,4,5-triacetoxy-6-(acetoxymethyl)-tetrahydro-2H-pyran-2-yl)thio)butylidene)amino sulfate (15)

Sulfur trioxide pyridinium complex (266 mg, 1.67 mmol, 5.0 eq) and pyridine (0.27 mL, 265 mg, 3.34 mmol, 10.0 eq) were added subsequently to a solution of compound **10** (155 mg, 0.33 mmol, 1.0 eq) in CH_2_Cl_2_ (1.0 mL) at 23°C under an argon atmosphere and the mixture was stirred at 60°C for 4 h. Afterwards, the reaction mixture was cooled down to 23°C and an aqueous solution of KHCO_3_ (2.68 mL, 5.35 mmol, 16.0 eq, 2 M) was added slowly under stirring. The mixture was stirred vigorously for 30 min, before it was concentrated under reduced pressure. The resulting residue was purified by flash chromatography through silica gel (dry load, CH_2_Cl_2_:MeOH 10/:0→5:1) yielding GSL **15** as a white amorphous solid (146 mg, 0.25 mmol, 75%).

**TLC** (CH_2_Cl_2_:MeOH/10:1), R*_f_* = 0.25 [UV^254^, KMnO_4_]. **IR** (ATR) [cm^-1^]: 2965, 2941, 2877, 1750, 1370, 1226, 1058, 911, 800, 642, 626, 577. **HRMS** (ESI) [m/z]: calculated [C_18_H_26_NO_13_S_2_]^-^, 528.0851; found 528.0845. **^1^H-NMR** (600 MHz, CD_3_OD) δ [ppm]: 5.39 (t, *J* = 9.3 Hz, 1H), 5.35 (d, *J* = 10.1 Hz, 1H), 5.04 (dd, *J* = 10.1, 9.3 Hz, 1H), 4.98 (dd, *J* = 10.2, 9.2 Hz, 1H), 4.24 – 4.21 (m, 1H), 4.21 (s, 1H), 4.14 (dd, *J* = 12.4, 2.4 Hz, 1H), 4.02 (ddd, *J* = 10.2, 5.5, 2.3 Hz, 1H), 2.63 (td, *J* = 7.2, 1.4 Hz, 2H), 2.06 (s, 3H), 2.03 (s, 3H), 2.02 (s, 3H), 1.98 (s, 3H), 1.76 (h, *J* = 7.4 Hz, 2H), 1.03 (t, *J* = 7.4 Hz, 3H). **^13^C-NMR** (151 MHz, CD_3_OD) δ [ppm]: 172.22, 171.50, 171.21, 170.91, 159.20, 80.88, 76.75, 75.05, 71.42, 69.55, 63.40, 53.18, 35.49, 21.72, 20.52, 13.92.

## Potassium ((*Z*)-1-(((2*S*,3*R*,4*S*,5*R*,6*R*)-3,4,5-triacetoxy-6-(acetoxymethyl)-tetrahydro-2H-pyran-2-yl)thio)pentylidene)amino sulfate (16)

Sulfur trioxide pyridinium complex (172 mg, 1.08 mmol, 5.0 eq) and pyridine (0.17 mL, 171 mg, 2.16 mmol, 10.0 eq) were added subsequently to a solution of compound **11** (100 mg, 0.33 mmol, 1.0 eq) in CH_2_Cl_2_ (1.0 mL) at 23°C under an argon atmosphere and the mixture was stirred to 60°C for 6 h. Afterward, the reaction mixture was cooled down to 23°C, and an aqueous solution of KHCO_3_ (1.73 mL, 3.45 mmol, 16.0 eq, 2 M) was added slowly under stirring. The mixture was stirred vigorously for 30 min, before it was concentrated under reduced pressure. The resulting residue was purified by flash chromatography through silica gel (dry load, CH_2_Cl_2_:MeOH 10/:0→5:1) yielding GSL **16** as a white amorphous solid (107 mg, 0.18 mmol, 85%).

**TLC** (CH_2_Cl_2_:MeOH/10:1), R*_f_* = 0.28 [UV^254^, KMnO_4_]. **IR** (ATR) [cm^-1^]: 2954, 2870, 2362, 2338, 1749, 1718, 1368, 1246, 1230, 1061, 1048, 777, 623. **HRMS** (ESI) [m/z]: calculated for [C_19_H_28_NO_13_S_2_]^-^, 542.1008; found 542.1001. **^1^H-NMR** (600 MHz, CD_3_OD) δ [ppm]: 5.39 (t, *J* = 9.3 Hz, 1H), 5.36 (d, *J* = 10.2 Hz, 1H), 5.05 (t, *J* = 9.8 Hz, 1H), 5.01 – 4.96 (m, 1H), 4.22 (dd, *J* = 12.5, 5.4 Hz, 1H), 4.14 (dd, *J* = 12.4, 2.3 Hz, 1H), 4.03 – 3.99 (m, 1H), 2.68 – 2.64 (m, 2H), 2.06 (s, 3H), 2.03 (s, 3H), 2.03 (s, 3H), 1.98 (s, 3H), 1.71 (dt, *J* = 15.3, 7.4 Hz, 2H), 1.45 (h, *J* = 7.4 Hz, 2H), 0.97 (t, *J* = 7.4 Hz, 3H). **^13^C-NMR** (151 MHz, CD_3_OD) δ [ppm]: 172.20, 171.49, 171.22, 170.91, 159.60, 80.87, 76.74, 75.03, 71.42, 69.52, 63.38, 33.38, 30.49, 23.22, 20.65, 20.53, 14.19.

## Potassium ((*Z*)-2-methyl-1-(((2*S*,3*R*,4*S*,5*S*,6*R*)-3,4,5-trihydroxy-6-(hydroxy-methyl)tetrahydro-2H-pyran-2-yl)thio)propylidene)amino sulfate (17)

A methanolic solution of ammonia (50.3 µL, 0.35 mmol, 2.0 eq, 7 M in MeOH) was added to a solution of compound **12** (100 mg, 0.18 mmol, 1.0 eq) in dry MeOH (3 mL) under an argon atmosphere and the reaction mixture was stirred for 24 h at 23°C. The mixture was filtered through a pipette packed with glass wool and anhydrous Na_2_SO_4_ and the filtrate was evaporated under reduced pressure to yield the GSL **17** (68.3 mg, 0.17 mmol, 97%) as a white amorphous solid.

**TLC** (CH_2_Cl_2_:MeOH/5:1), R*_f_* = 0.25 [UV^254^, KMnO_4_]. **IR** (ATR) [cm^-1^]: 3421, 2975, 2935, 2879, 2524, 2362, 2337, 1271, 1241, 1053, 867, 789, 630, 604, 5081. **HRMS** (ESI) [m/z]: calculated for [C_10_H_18_NO_9_S_2_]^-^, 360.0428; found 360.0493. **^1^H-NMR** (800 MHz, CD_3_OD) δ [ppm]: 4.87 (d, *J* = 9.7 Hz, 1H), 3.83 (dd, *J* = 12.2, 2.0 Hz, 1H), 3.64 (dd, *J* = 12.2, 5.3 Hz, 1H), 3.40 (t, *J* = 8.8 Hz, 1H), 3.34 – 3.33 (m, 1H), 3.25 (dd, *J* = 9.8, 8.6 Hz, 1H), 2.98 (p, *J* = 6.8 Hz, 1H), 1.31 (d, *J* = 3.1 Hz, 3H), 1.30 (d, *J* = 3.1 Hz, 3H). **^13^C-NMR** (201 MHz, CD_3_OD) δ [ppm]: 165.22, 83.94, 82.04, 79.59, 74.42, 71.17, 62.63, 33.94, 22.40, 21.21.

## Potassium ((*Z*)-3-methyl-1-(((2*S*,3*R*,4*S*,5*S*,6*R*)-3,4,5-trihydroxy-6-(hydroxymethyl)tetrahydro-2H-pyran-2-yl)thio)butylidene)amino sulfate (18)

A methanolic solution of ammonia (49.1 µL, 0.34 mmol, 2.0 eq, 7 M in MeOH) was added to a solution of compound **13** (100 mg, 0.17 mmol, 1.0 eq) in dry MeOH (3 mL) under an argon atmosphere and the reaction mixture was stirred for 24 h at 23°C. The mixture was filtered through a pipette packed with glass wool and anhydrous Na_2_SO_4_ and the filtrate was evaporated under reduced pressure to yield the GSL **18** (69.7 mg, 0.17 mmol, 98%) as a white amorphous solid.

**TLC** (CH_2_Cl_2_:MeOH/5:1), R*_f_* = 0.22 [UV^254^, KMnO_4_]. **IR** (ATR) [cm^-1^]: 3401, 2960, 2930, 2873, 2362, 2337, 1271, 1239, 1059, 909, 867, 801, 647, 576. **HRMS** (ESI) [m/z]: calculated for [C_11_H_20_NO_9_S_2_]^-^, 374.0585; found 374.0651. **^1^H-NMR** (600 MHz, CD_3_OD) δ [ppm]: 4.81 (d, *J* = 9.7 Hz, 1H), 4.59 (s, 1H), 3.85 (d, *J* = 12.4 Hz, 1H), 3.64 (dd, *J* = 12.3, 5.4 Hz, 1H), 3.39 (t, *J* = 8.7 Hz, 1H), 3.32 (d, *J* = 6.4 Hz, 1H), 3.25 (t, *J* = 9.2 Hz, 1H), 2.60 (dd, *J* = 14.7, 5.9 Hz, 1H), 2.47 (dd, *J* = 14.7, 8.3 Hz, 1H), 2.16 (dp, *J* = 13.0, 6.6 Hz, 1H), 1.03 (d, *J* = 6.6 Hz, 3H), 1.00 (d, *J* = 6.6 Hz, 3H). **^13^C-NMR** (151 MHz, CD_3_OD) δ [ppm]: 161.13, 83.90, 82.40, 79.56, 74.22, 71.14, 62.64, 42.30, 28.02, 23.11, 22.36.

## Potassium ((*Z*)-2-methyl-1-(((2*S*,3*R*,4*S*,5*S*,6*R*)-3,4,5-trihydroxy-6-(hydroxymethyl)tetrahydro-2H-pyran-2-yl)thio)butylidene)amino sulfate (19)

A methanolic solution of ammonia (34.4 µL, 0.24 mmol, 2.0 eq, 7 M in MeOH) was added to a solution of compound **14** (70 mg, 0.12 mmol, 1.0 eq) in dry MeOH (3 mL) under an argon atmosphere and the reaction mixture was stirred for 24 h at 23°C. The mixture was filtered through a pipette packed with glass wool and anhydrous Na_2_SO_4_ and the filtrate was evaporated under reduced pressure to yield the GSL **19** (48.8 mg, 0.12 mmol, 98%) as a white amorphous solid.

**TLC** (CH_2_Cl_2_:MeOH/5:1), R*_f_* = 0.23 [UV^254^, KMnO_4_]. **IR** (ATR) [cm^-1^]: 3393, 2968, 2934, 2877, 2362, 2337, 1240, 1058, 880, 846, 792, 781, 636, 582. **HRMS** (ESI) [m/z]: calculated for [C_11_H_20_NO_9_S_2_]^-^, 374.0585; found 374.0581. **^1^H-NMR** (800 MHz, CD_3_OD) δ [ppm]: 4.88 (dd, *J* = 17.9, 9.8 Hz, 1H), 3.83 (dt, *J* = 12.0, 2.1 Hz, 1H), 3.66 (td, *J* = 11.8, 5.1 Hz, 1H), 3.39 (tt, *J* = 9.5, 4.7 Hz, 1H), 3.37 – 3.35 (m, 1H), 3.34 – 3.32 (m, 1H), 3.25 (td, *J* = 9.1, 6.0 Hz, 1H), 2.79 – 2.69 (m, 1H), 2.00 – 1.81 (m, 1H), 1.55 (dtd, *J* = 13.9, 7.3, 4.5 Hz, 1H), 1.28 (t, *J* = 6.8 Hz, 3H), 0.99 (dt, *J* = 12.4, 7.3 Hz, 3H). **^13^C-NMR** (201 MHz, CD_3_OD) δ [ppm]: 164.24, 84.11, 83.99, 82.08, 81.95, 79.61, 79.58, 74.41, 71.17, 71.11, 62.61, 40.84, 40.68, 29.84, 28.93, 20.01, 18.82, 12.34, 12.01.

## Potassium ((*Z*)-1-(((2*S*,3*R*,4*S*,5*S*,6*R*)-3,4,5-trihydroxy-6-(hydroxymethyl)-tetrahydro-2H-pyran-2-yl)thio)butylidene)amino sulfate (20)

A methanolic solution of ammonia (12.4 µL, 0.09 mmol, 2.0 eq, 7 M in MeOH) was added to a solution of compound **15** (24.6 mg, 0.04 mmol, 1.0 eq) in dry MeOH (1 mL) under an argon atmosphere and the reaction mixture was stirred for 24 h at 23°C. The mixture was filtered through a pipette packed with glass wool and anhydrous Na_2_SO_4_ and the filtrate was evaporated under reduced pressure to yield the GSL **20** (17 mg, 0.04 mmol, 98%) as white amorphous solid.

**TLC** (CH_2_Cl_2_:MeOH/5:1), R*_f_* = 0.24 [UV^254^, KMnO_4_]. **IR** (ATR) [cm^-1^]: 3384, 2964, 2933, 2876, 2362, 2337, 1272, 1242, 1105, 1059, 885, 803, 644, 629, 578. **HRMS** (ESI) [m/z]: calculated for [C_10_H_18_NO_9_S_2_]^-^, 360.0428; found 360.0426. **^1^H-NMR** (600 MHz, CD_3_OD) δ [ppm]: 4.81 (d, *J* = 9.8 Hz, 1H), 3.84 (dd, *J* = 12.2, 1.8 Hz, 1H), 3.66 – 3.61 (m, 1H), 3.39 (t, *J* = 8.8 Hz, 1H), 3.32 (d, *J* = 2.1 Hz, 2H), 3.25 (dd, *J* = 9.8, 8.6 Hz, 1H), 2.66 (td, *J* = 7.5, 2.0 Hz, 2H), 1.77 (h, *J* = 7.2 Hz, 2H), 1.01 (t, *J* = 7.4 Hz, 3H). **^13^C-NMR** (151 MHz, CD_3_OD) δ [ppm]: 161.73, 83.76, 82.33, 79.59, 74.21, 71.20, 62.72, 35.52, 21.97, 14.03.

## Potassium ((*Z*)-1-(((2*S*,3*R*,4*S*,5*S*,6*R*)-3,4,5-trihydroxy-6-(hydroxymethyl)-tetrahydro-2H-pyran-2-yl)thio)pentylidene)amino sulfate (21)

A methanolic solution of ammonia (13 µL, 0.09 mmol, 2.0 eq, 7 M in MeOH) was added to a solution of compound **16** (26.5 mg, 0.05 mmol, 1.0 eq) in dry MeOH (1 mL) under an argon atmosphere and the reaction mixture was stirred for 24 h at 23°C. The mixture was filtered through a pipette packed with glass wool and anhydrous Na_2_SO_4_ and the filtrate was evaporated under reduced pressure to result in the GSL **21** (18.1 mg, 0.04 mmol, 96%) as a white amorphous solid.

**TLC** (CH_2_Cl_2_:MeOH/5:1), R*_f_* = 0.21 [UV^254^, KMnO_4_]. **IR** (ATR) [cm^-1^]: 3393, 2959, 2932, 2874, 2362, 2337, 1272, 1245, 1061, 874, 801, 643, 579. **HRMS** (ESI) [m/z]: calculated for C_11_H_20_NO_9_S_2_]^-^, 374.0585; found 374.0584. **^1^H-NMR** (800 MHz, CD_3_OD) δ [ppm]: 4.82 (d, *J* = 9.8 Hz, 1H), 3.84 (dd, *J* = 12.2, 1.8 Hz, 1H), 3.64 (ddd, *J* = 12.2, 4.0, 1.5 Hz, 1H), 3.39 (ddd, *J* = 8.8, 5.8, 3.1 Hz, 1H), 3.32 (dd, *J* = 3.8, 2.0 Hz, 2H), 3.25 (dd, *J* = 9.8, 8.7 Hz, 1H), 2.72 – 2.64 (m, 2H), 1.73 (p, *J* = 7.0 Hz, 2H), 1.44 (dt, *J* = 14.4, 7.3 Hz, 2H), 0.96 (t, *J* = 7.4 Hz, 3H). **^13^C-NMR** (201 MHz, CD_3_OD) δ [ppm]: 160.55, 82.35, 80.93, 78.19, 72.80, 69.78, 61.30, 31.95, 29.38, 21.98, 12.75.

# 3. ^1^H, ^13^C NMR, HRMS and IR Spectra of the Compounds

(2*R*,3*R*,4*S*,5*R*,6*S*)-2-(acetoxymethyl)-6-(((*Z*)-1-(hydroxyimino)-2-methylpropyl)thio)tetra-hydro-2H-pyran-3,4,5-triyl triacetate (**7**)

(2*R*,3*R*,4*S*,5*R*,6*S*)-2-(acetoxymethyl)-6-(((*Z*)-1-(hydroxyimino)-3-methylbutyl)thio)tetrahydro-2H-pyran-3,4,5-triyl triacetate (**8**)

2*R*,3*R*,4*S*,5*R*,6*S*)-2-(acetoxymethyl)-6-(((*Z*)-1-(hydroxyimino)-2-methylbutyl)thio)tetrahydro-2H-pyran-3,4,5-triyl triacetate (**9**)

(2*R*,3*R*,4*S*,5*R*,6*S*)-2-(acetoxymethyl)-6-(((*Z*)-1-(hydroxyimino)butyl)thio)tetrahydro-2H-pyran-3,4,5-triyl triacetate (**10**)

**(**2*R*,3*R*,4*S*,5*R*,6*S*)-2-(acetoxymethyl)-6-(((*Z)*-1-(hydroxyimino)pentyl)thio)tetrahydro-2H-pyran-3,4,5-triyl triacetate (**11**)

Potassium ((*Z*)-2-methyl-1-(((2*S*,3*R*,4S,5*R*,6*R*)-3,4,5-triacetoxy-6-(acetoxymethyl)tetrahydro-2H-pyran-2-yl)thio)propylidene)amino sulfate (**12**)

Potassium ((*Z*)-3-methyl-1-(((2*S*,3*R*,4*S*,5*R*,6*R*)-3,4,5-triacetoxy-6-(acetoxymethyl)tetrahydro-2H-pyran-2-yl)thio)butylidene)amino sulfate (**13**)

Potassium ((*Z*)-2-methyl-1-(((2*S*,3*R*,4*S*,5*R*,6*R*)-3,4,5-triacetoxy-6-(acetoxymethyl)tetrahydro-2H-pyran-2-yl)thio)butylidene)amino sulfate (14)

Potassium ((*Z*)-1-(((2*S*,3*R*,4*S*,5*R*,6*R*)-3,4,5-triacetoxy-6-(acetoxymethyl)tetrahydro-2H-pyran-2-yl)thio)butylidene)amino sulfate (**15**)

Potassium ((*Z*)-1-(((2*S*,3*R*,4*S*,5*R*,6*R*)-3,4,5-triacetoxy-6-(acetoxymethyl)tetrahydro-2H-pyran-2-yl)thio)pentylidene)amino sulfate (**16**)

Potassium ((*Z*)-2-methyl-1-(((2*S*,3*R*,4*S*,5*S*,6*R*)-3,4,5-trihydroxy-6-(hydroxymethyl)tetrahydro-2H-pyran-2-yl)thio)propylidene)amino sulfate (**17**)

Potassium ((*Z*)-3-methyl-1-(((2*S*,3*R*,4*S*,5*S*,6*R*)-3,4,5-trihydroxy-6-(hydroxymethyl)tetrahydro-2H-pyran-2-yl)thio)butylidene)amino sulfate (**18**)

Potassium ((*Z*)-2-methyl-1-(((2*S*,3*R*,4*S*,5*S*,6*R*)-3,4,5-trihydroxy-6-(hydroxymethyl)tetrahydro-2H-pyran-2-yl)thio)butylidene)amino sulfate (**19**)

Potassium ((*Z*)-1-(((2*S*,3*R*,4*S*,5*S*,6*R*)-3,4,5-trihydroxy-6-(hydroxymethyl)tetrahydro-2H-pyran-2-yl)thio)butylidene)amino sulfate (**20**)

Potassium ((*Z*)-1-(((2*S*,3*R*,4*S*,5*S*,6*R*)-3,4,5-trihydroxy-6-(hydroxymethyl)tetrahydro-2H-pyran-2-yl)thio)pentylidene)amino sulfate (**21**)
