# Supplementary material for: Glucosinolate diversity in seven field-collected Brassicaceae species
Source: PLoS One. 2025 Nov 13;20(11):e0336172. doi: 10.1371/journal.pone.0336172 (PMC12614607; doi:10.1371/journal.pone.0336172)
Supplement: S2 Methods — (DOCX) [file pone.0336172.s015.docx]

**S2 Methods: Glucosinolate analysis**

**1. Preparation of desulfoglucosinolates**

Freeze-dried and grinded plant material (10-15 mg) was extracted in 1 ml 80% (v/v) methanol for 15 min. After centrifugation at 2500 rpm for 10 min, the supernatant (400-800 µl, depending on the species) was loaded on DEAE-sephadex A25 (Sigma-Aldrich, Darmstadt, Germany; 45 µl dry matrix preincubated with 800 µl water per sample) which was mounted in a 96 well filter plate placed in a NucloVac 96 vacuum manifold (Macherey-Nagel, Düren, Germany) and had been washed with 1 ml water and 1 ml 80% (v/v) methanol per well. After sample application, the material was washed with 1 ml 80% (v/v) methanol, 2x 1 ml deionized water, and 1 ml 0.02 M sodium acetate buffer (pH 5.0), 50 µl sulfatase from *Helix pomatia* (Type H-1, Sigma-Aldrich, Darmstadt, Germany, dissolved in 0.02 M sodium acetate buffer, pH 5.0, to a final concentration of approximately 14 mg/ml after ethanol precipitation) were added and samples were incubated overnight. Elution of desulfoglucosinolates was achieved by addition of 60% (v/v) methanol (2x 0.5 ml). The solvent was evaporated under an air stream, and samples were redissolved in 100 µl water. To generate an external standard, 50-100 µl of 1 mM 4-hydroxybenzylglucosinolate (purified from seeds of *Sinapis alba* L., 99%) were added to 1 ml 80% (v/v) methanol (without plant material) and subjected to the same steps

**2. Qtrap 3200 mass spectrometer settings for desulfoglucosinolate analysis**

Enhanced MS scan type (negative mode) was performed from 150 Da to 589 Da in 0.44 s and from 583 Da to 700 Da in 0.12 s using the following settings: curtain gas: 10 psi, ionizaion voltage (IS): -4500 V, ion source temperature (TEM): 550°C, ion source gas 1 (GS1): 40 psi, ion source gas 2 (GS2): 40 psi, collision activated dissociation (CAD): high, declustering potential (DP): -40 V, entrance potential (EP): -10 V und collision energy (CE): ‑10 eV.

**3. Qtrap 3200 mass spectrometer settings for analysis of intact glucosinolate**

The mass spectrometer was run in the precursor ion scan type (negative mode). The masses that generate a mass of 97 Da after fragmentation were detected by scanning from 310 Da to 600 Da in 0.5 s using the following settings: curtain gas: 45 psi, ionization voltage (IS): -4500 V, ion source temperature (TEM): 400°C, ion source gas 1 (GS1): 35 psi, ion source gas 2 (GS2): 50 psi, collision activated dissociation (CAD): medium, declustering potential (DP): _-_55 V, entrance potential (EP): -10 V, collision energy (CE): -40 eV and colision cell exit potential (CXP): -3 V.

**4. Orbitrap mass spectrometer settings for desulfoglucosinolate analysis**

The Orbitrap mass spectrometer was used with the following settings: H-ESI static ionization voltage (positive): 3400 V, HCD collision energy: 30 V, static gas mode, sheath gas pressure: 3 Arb, auxiliary gas pressure: 2 Arb, sweep gas pressure: 0 Arb, ion transfer tube temperature: 320 °C, vaporizer temperature: 0 °C, RF lens: 70%.
