# Supplementary material for: Glucosinolate diversity in seven field-collected Brassicaceae species
Source: PLoS One. 2025 Nov 13;20(11):e0336172. doi: 10.1371/journal.pone.0336172 (PMC12614607; doi:10.1371/journal.pone.0336172)

**S1 Appendix: MS^2^ spectra of desulfoglucosinolates as detected for standards (framed in green) and in plant samples.** Spectra were recorded with a collision energy of 30 V except those frame in yellow which were recorded with 10 V collision energy. For assignment of spectra to plant species, the reader is referred to the sample information line in each image. The first two letters indicate the plant species (Ca, *Cardamine amara*; Ci, *C. impatiens*; Ld, *Lepidium draba*; Lr, *Lunaria rediviva*; Hm, *Hesperis matronalis*; Cp, *C. pratensis*; Ds, *Descurainia sophia*). Primary, secondary, and tertiary alcohols could only be distinguished from one another with the aid of standards. Since theoretical differentiation options, such as dehydration peaks of varying intensities and C-C cleavage because of secondary or tertiary alcohols, did not apply to the standards, no conclusions were drawn about the structures without an existing standard. # indicates that standards were not available (tentative identification). ds, desulfo.

List of compounds with exact mass

[ds 2-propenyl 280.085 3](#_Toc210295992)

[ds n-propyl 282.1006 3](#_Toc210295993)

[ds 1-methylethyl 282.1006 4](#_Toc210295994)

[ds 3-butenyl 294.1006 6](#_Toc210295995)

[ds n-butyl 296.1163 7](#_Toc210295996)

[ds 1-methylpropyl 296.1163 7](#_Toc210295997)

[ds 2-methylpropyl 296.1163 8](#_Toc210295998)

[ds 1-(hydroxymethyl)ethyl (#) 298.0955 10](#_Toc210295999)

[ds 4-pentenyl 308.1163 10](#_Toc210296000)

[ds 1-(hydroxymethyl)propyl (#) 312.1112 11](#_Toc210296001)

[ds 2-hydroxy-2-methylbutyl 326.1268 11](#_Toc210296002)

[unidentified ds hydroxypentyl or isomer (#) 326.1268 11](#_Toc210296003)

[ds benzyl 330.1006 12](#_Toc210296004)

[ds 3-(hydroxymethyl)pentyl 340.1425 13](#_Toc210296005)

[ds 2-hydroxy-3-methylpentyl 340.1425 13](#_Toc210296006)

[ds 4-(methylthio)butyl 342.104 14](#_Toc210296007)

[ds 4-hydroxybenzyl 346.0955 15](#_Toc210296008)

[ds 5-(methylthio)pentyl (#) 356.1197 16](#_Toc210296009)

[ds 4-(methylsulfinyl)butyl 358.0989 16](#_Toc210296010)

[ds 3,4-dihydroxybenzyl (#) 362.0905 17](#_Toc210296011)

[ds indol-3-ylmethyl 369.1115 18](#_Toc210296012)

[ds 6-(methylthio)hexyl (#) 370.1353 19](#_Toc210296013)

[ds 5-(methylsulfinyl)pentyl 372.1145 20](#_Toc210296014)

[ds 4-(methylsulfonyl)butyl (#) 374.0938 21](#_Toc210296015)

[ds 7-(methylthio)heptyl 384.151 21](#_Toc210296016)

[ds 4-hydroxyindol-3-ylmethyl 385.1064 21](#_Toc210296017)

[ds 6-(methylsulfinyl)hexyl 386.1302 22](#_Toc210296018)

[ds 5-(methylsulfonyl)pentyl (#) 388.1095 23](#_Toc210296019)

[ds 8-(methylthio)octyl (#) 398.1666 23](#_Toc210296020)

[ds 4-methoxyindol-3-ylmethyl 399.1221 23](#_Toc210296021)

[ds 1-methoxyindol-3-ylmethyl 399.1221 25](#_Toc210296022)

[ds 7-(methylsulfinyl)heptyl (#) 400.1459 26](#_Toc210296023)

[ds 8-(methylsulfinyl)octyl 414.1615 27](#_Toc210296024)

[ds 9-(methylsulfinyl)nonyl (#) 428.1772 27](#_Toc210296025)

[tentative ds 4-apiosyloxybenzyl (#) 478.1378 28](#_Toc210296026)

[ds 4-apiosyloxy-3-hydroxybenzyl 494.1327 28](#_Toc210296027)

# ds 2-propenyl 280.085


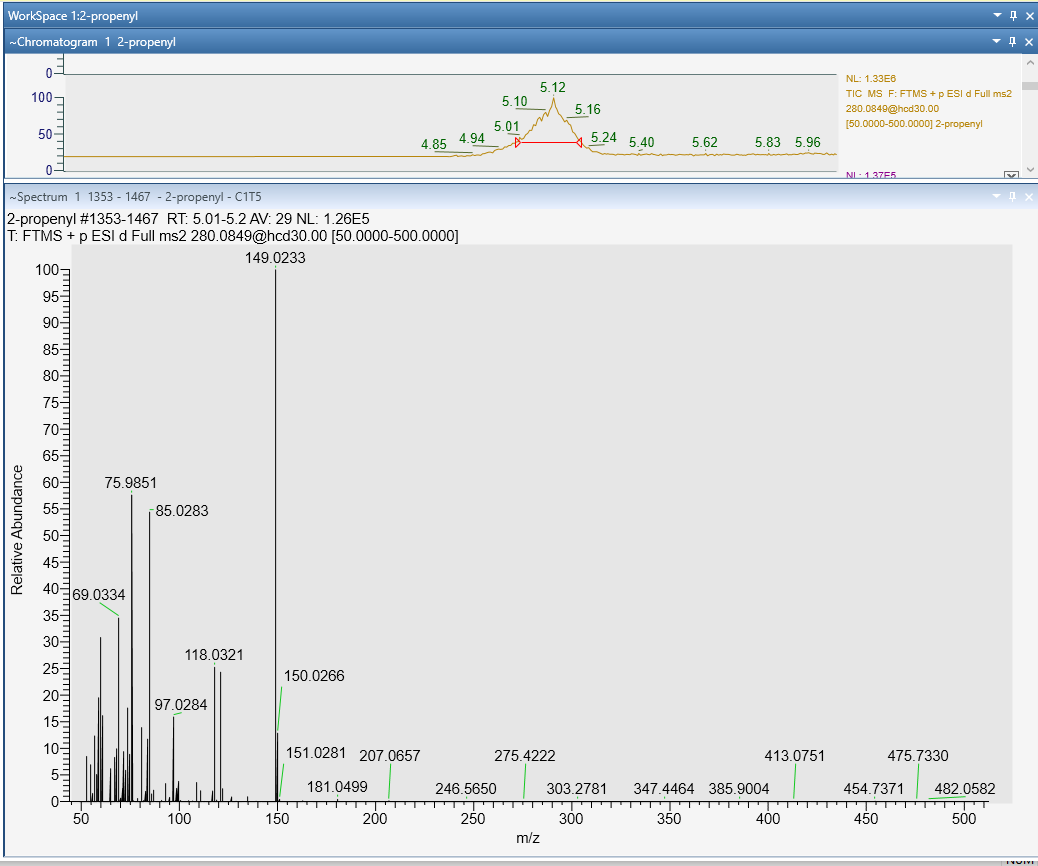


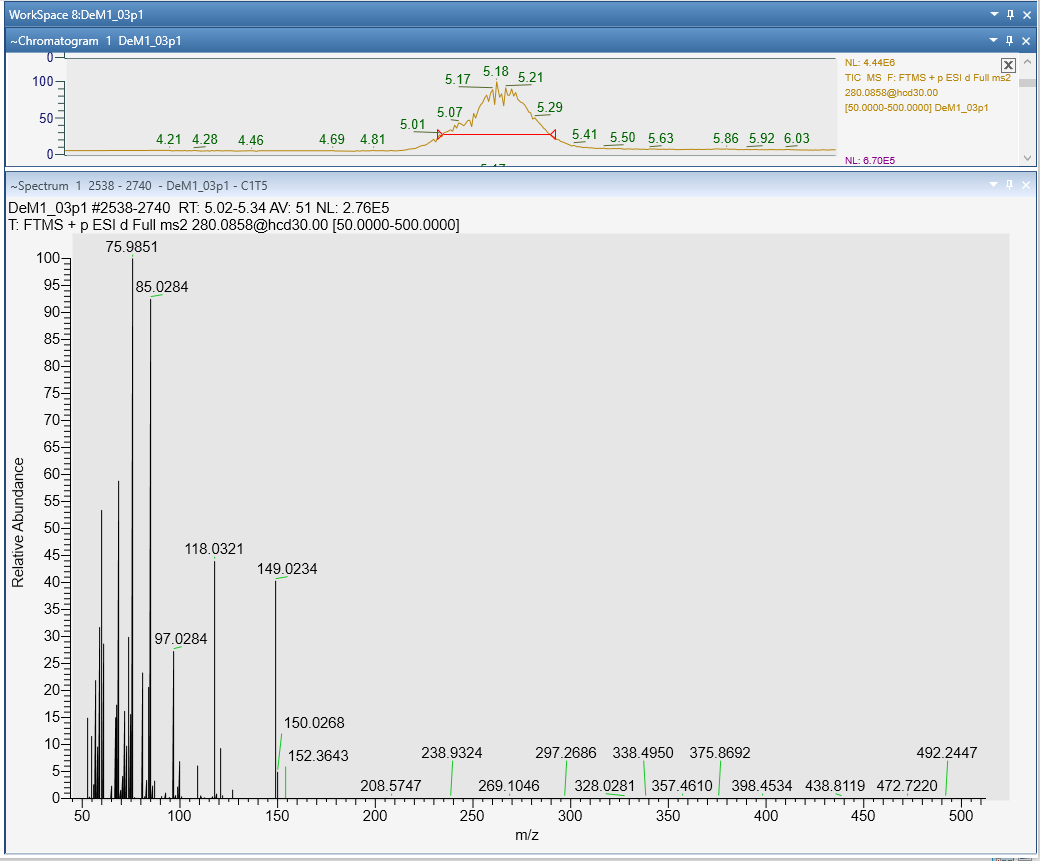


# ds n-propyl 282.1006


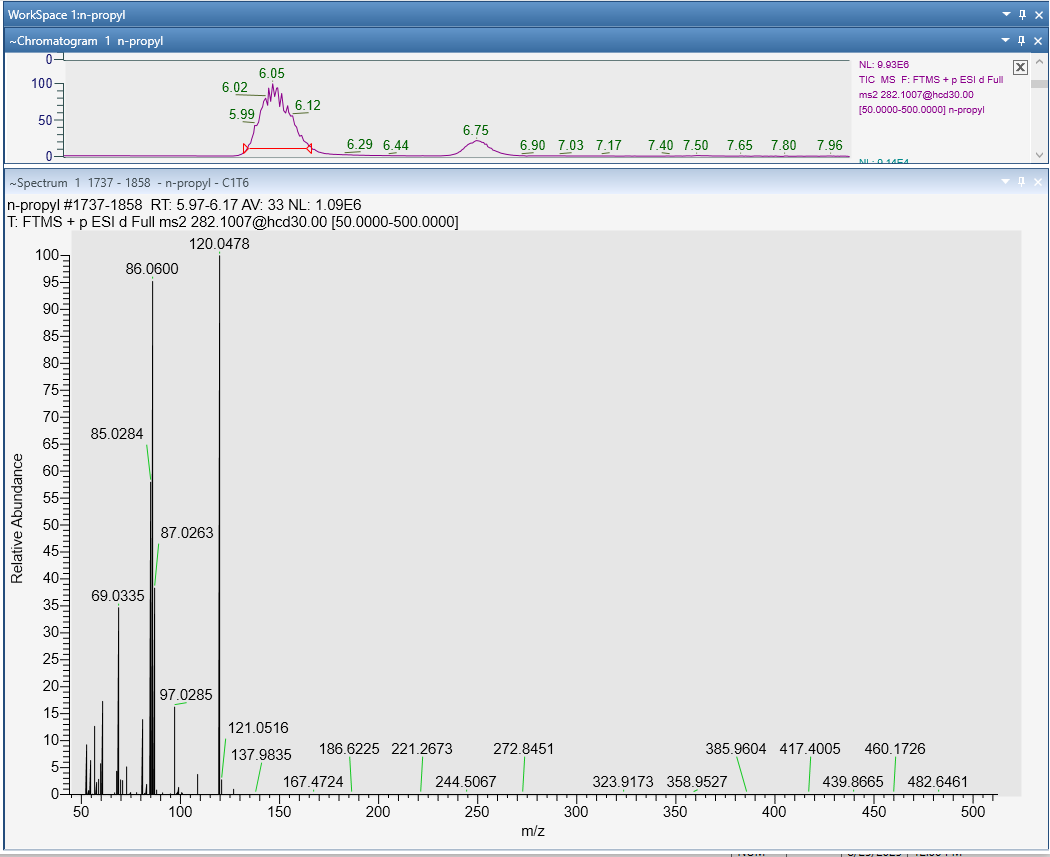


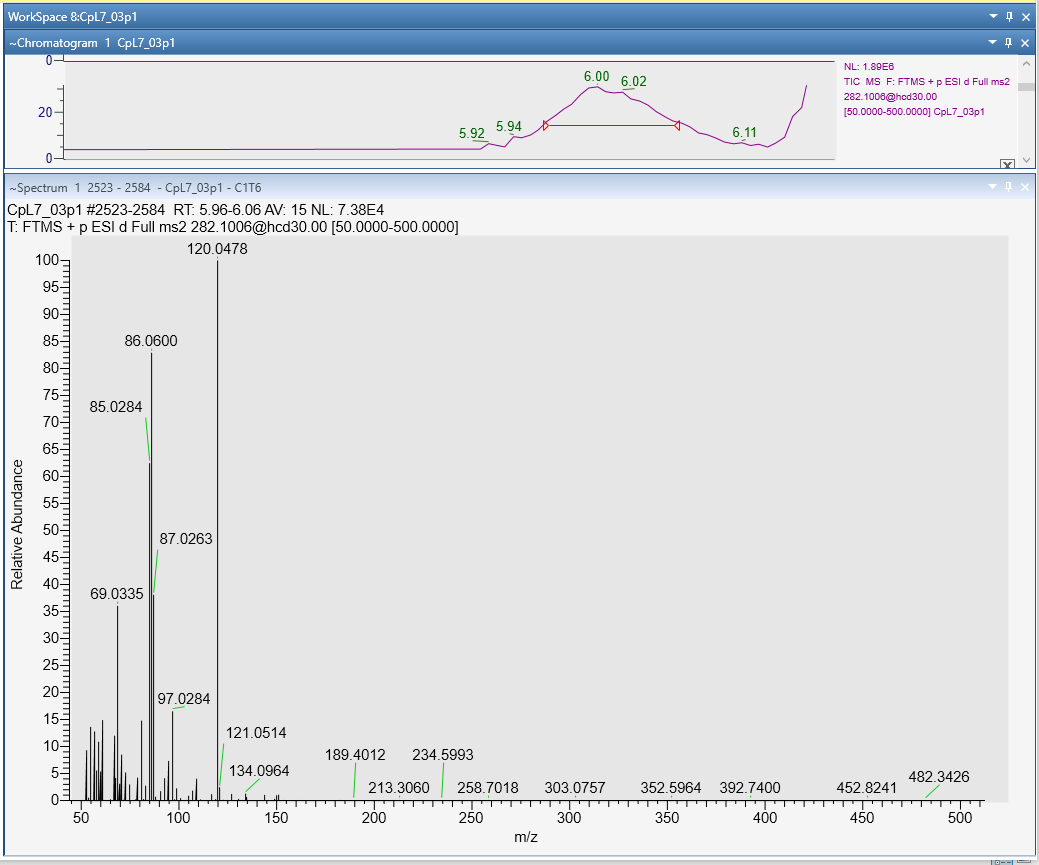


# ds 1-methylethyl 282.1006


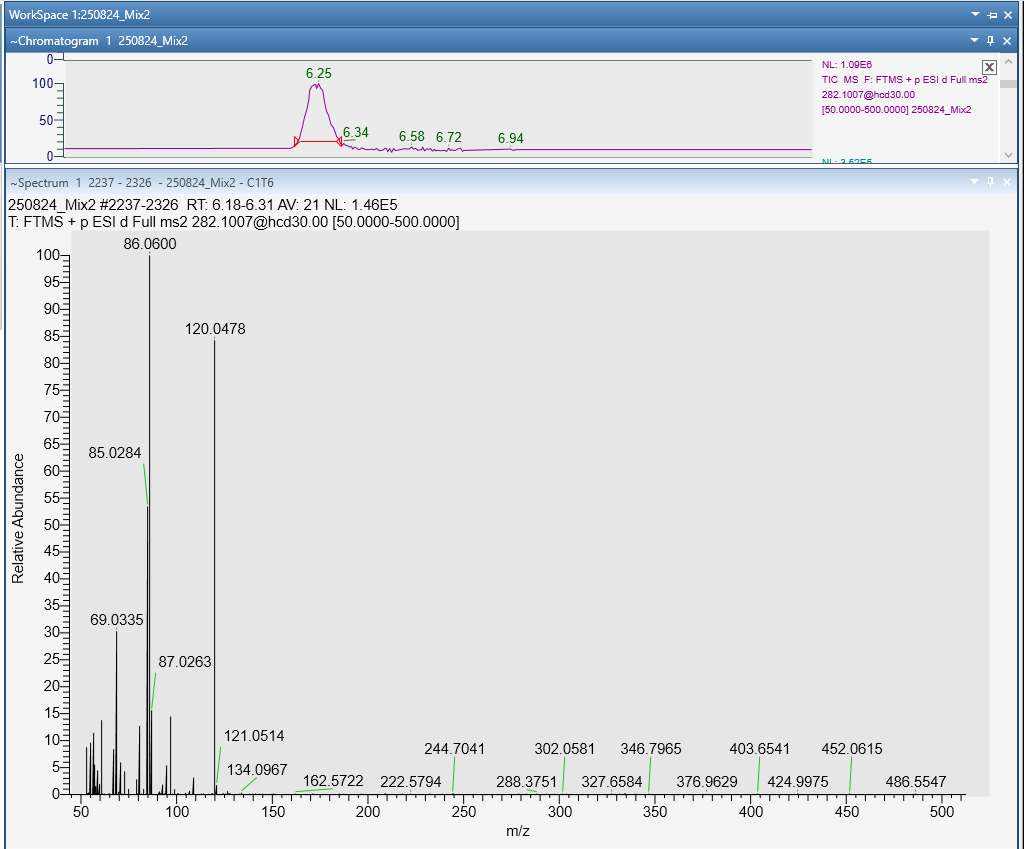


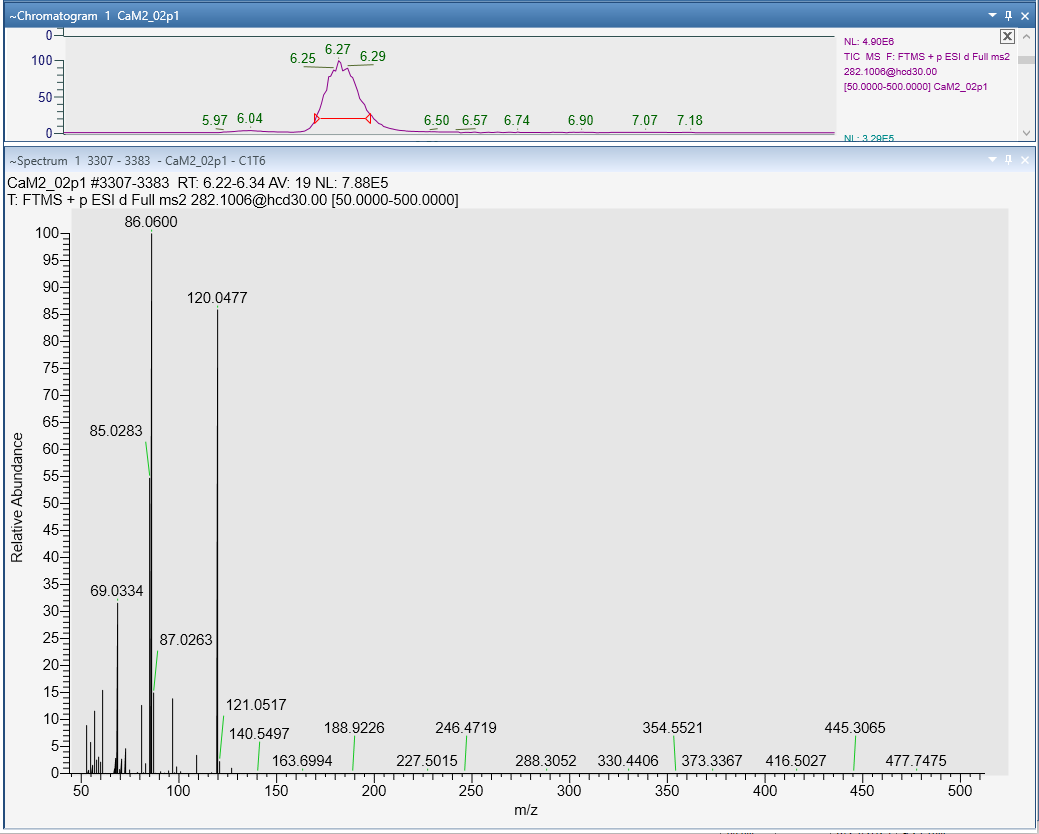


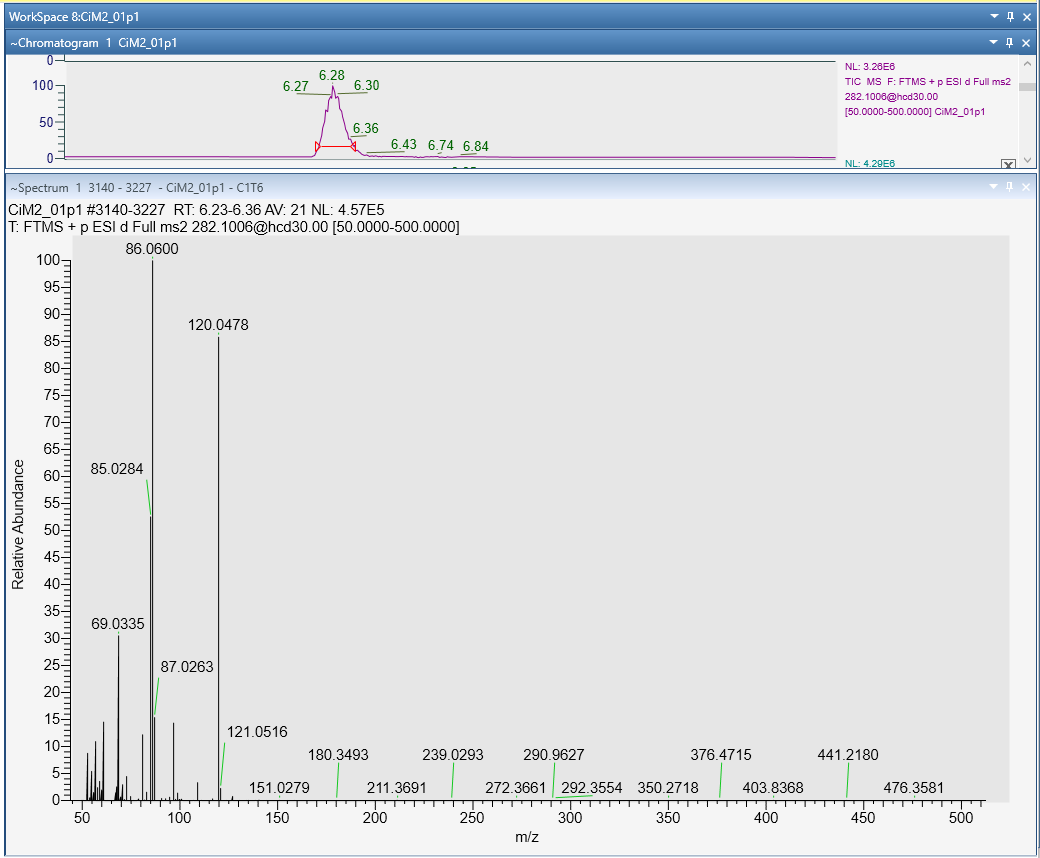


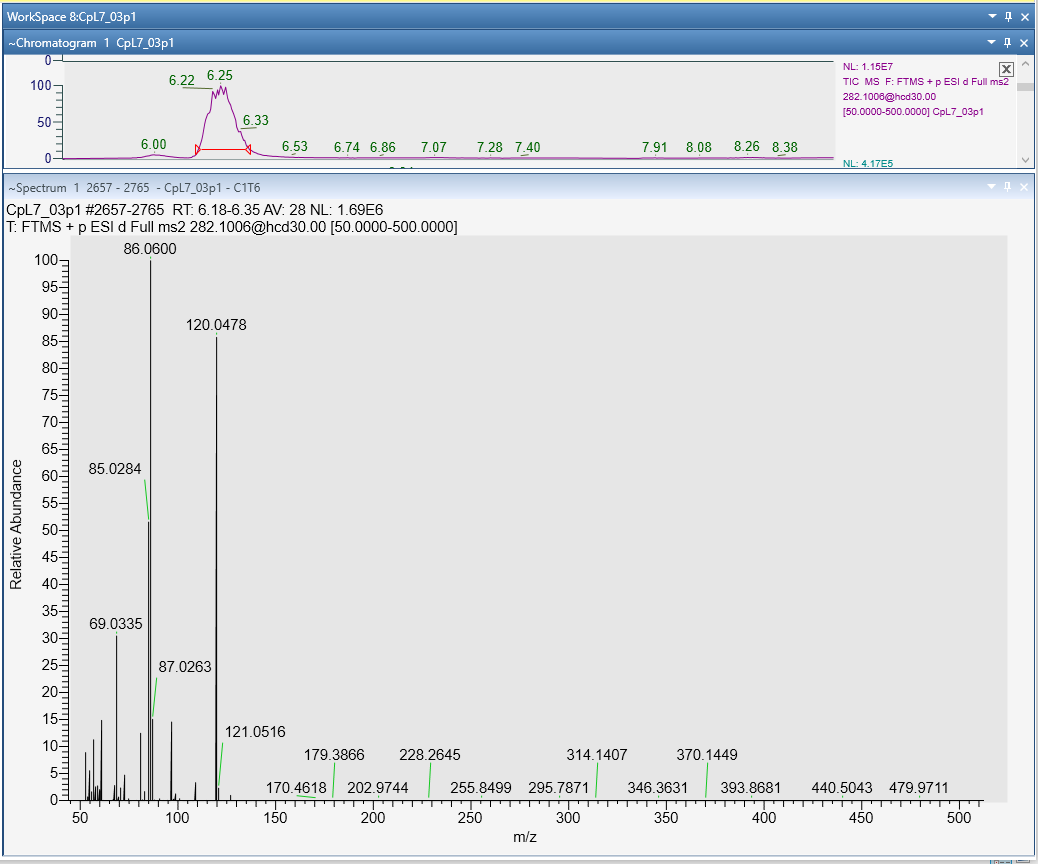


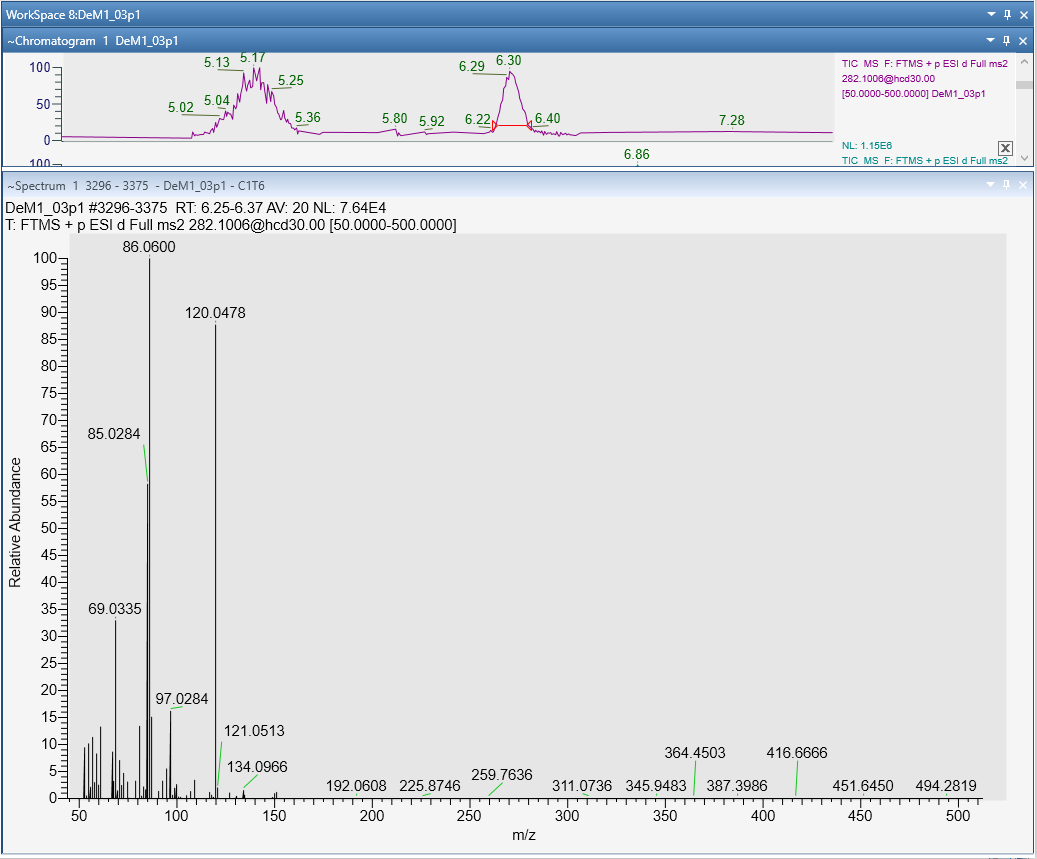


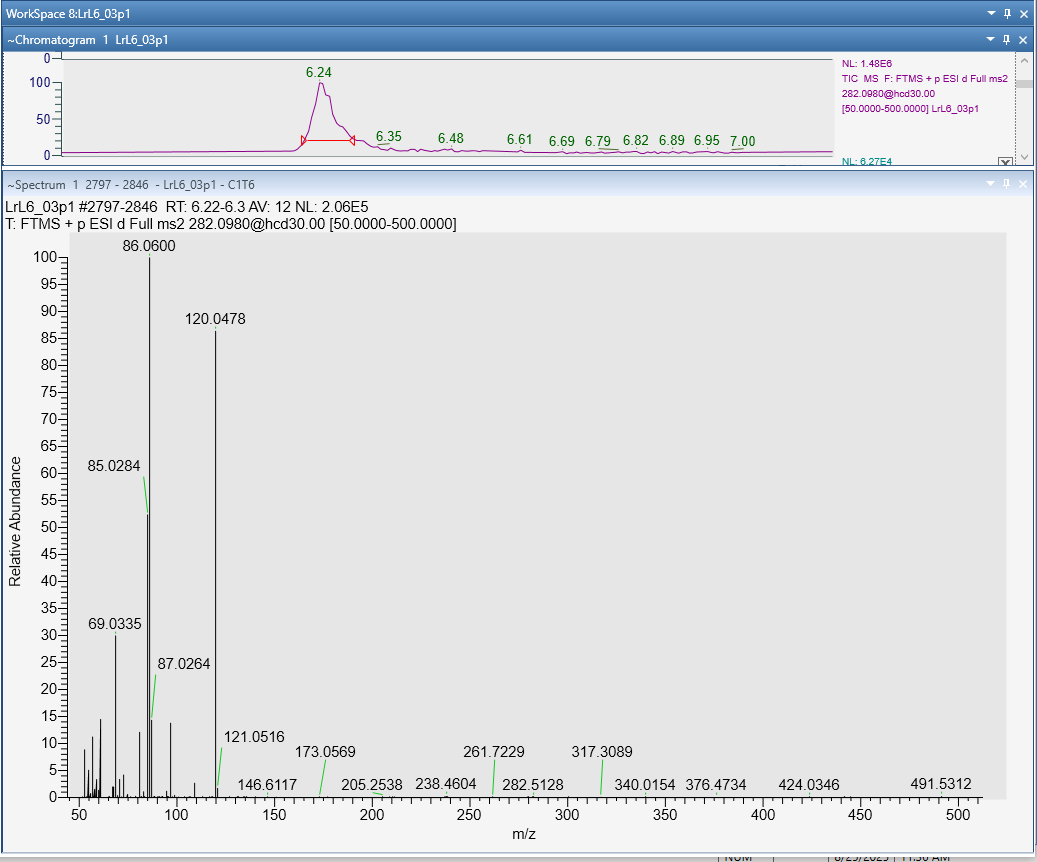


# ds 3-butenyl 294.1006


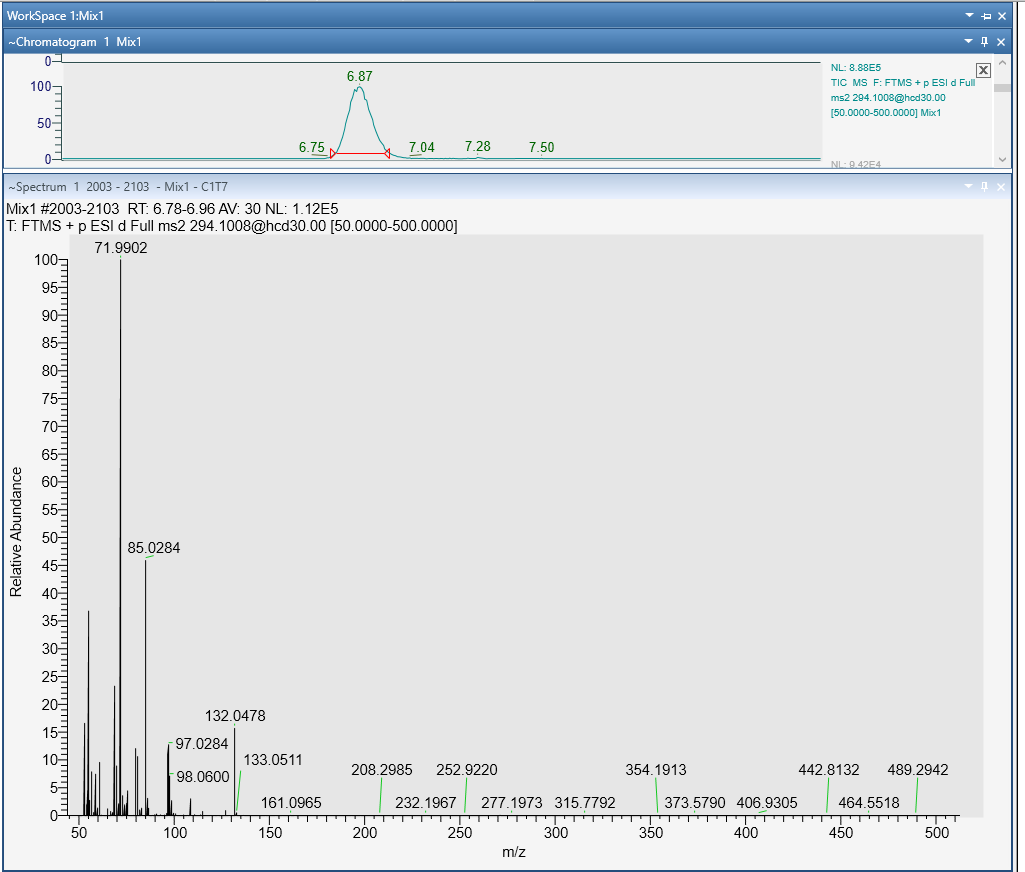


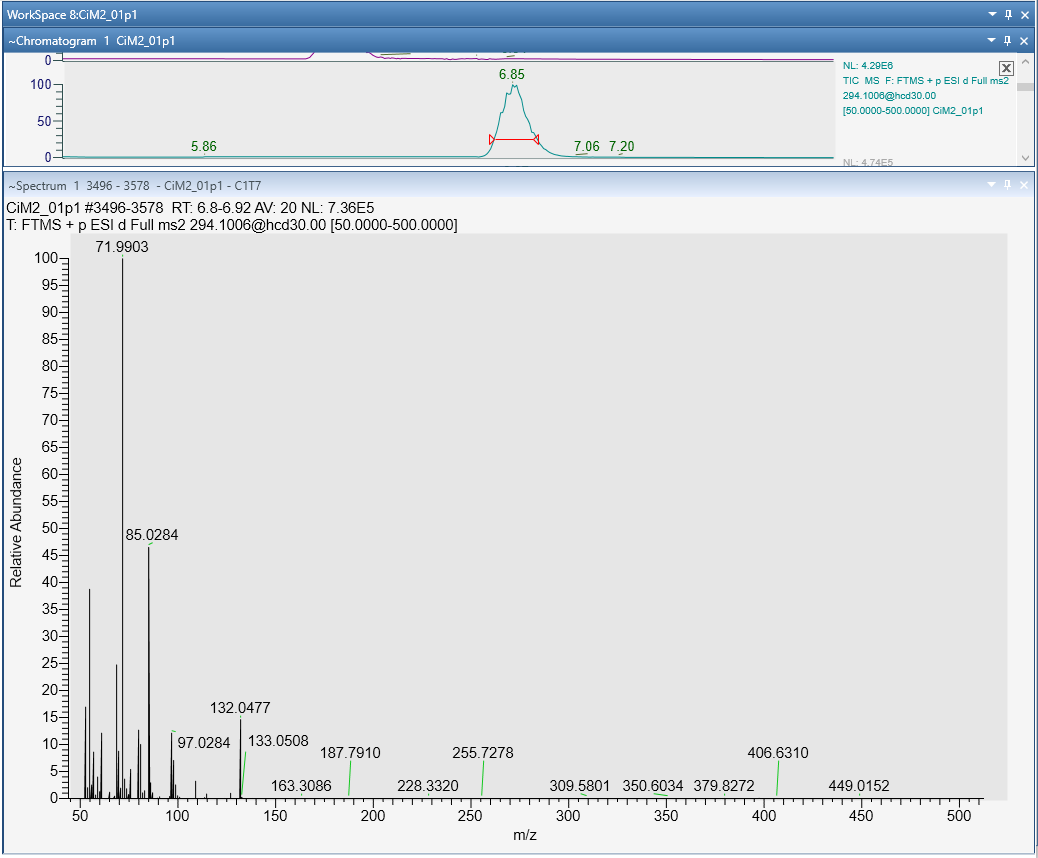


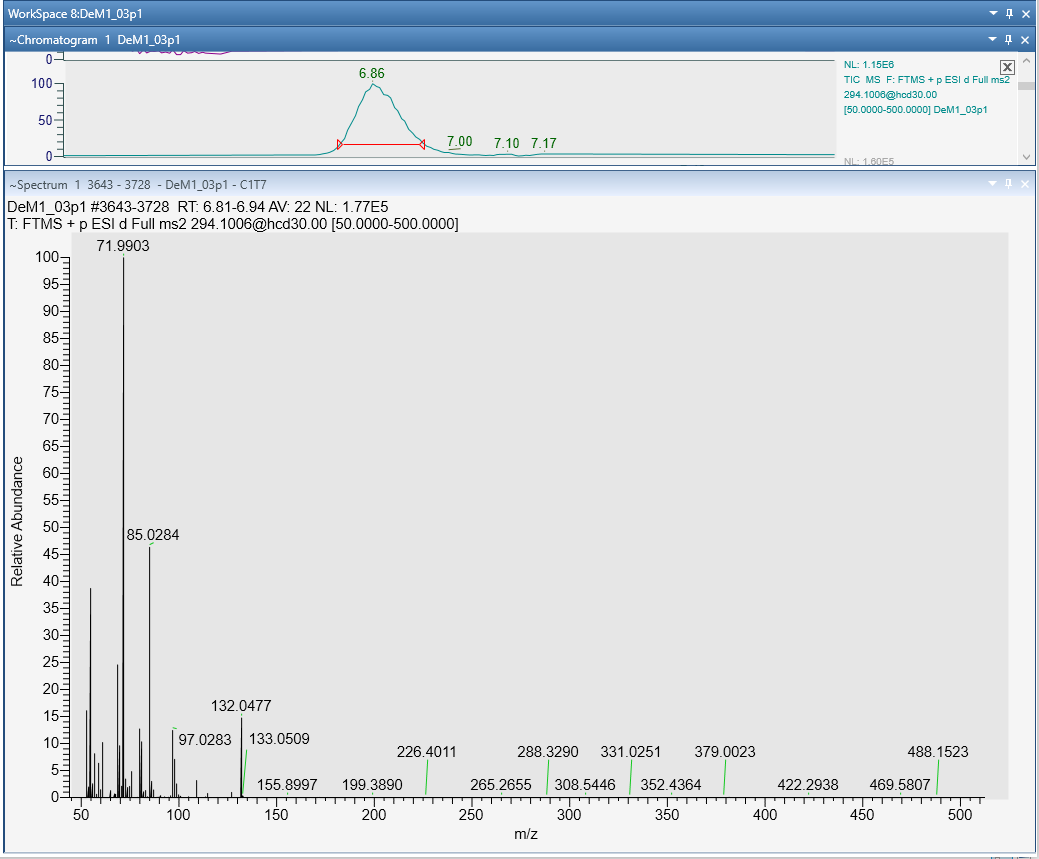


# ds n-butyl 296.1163


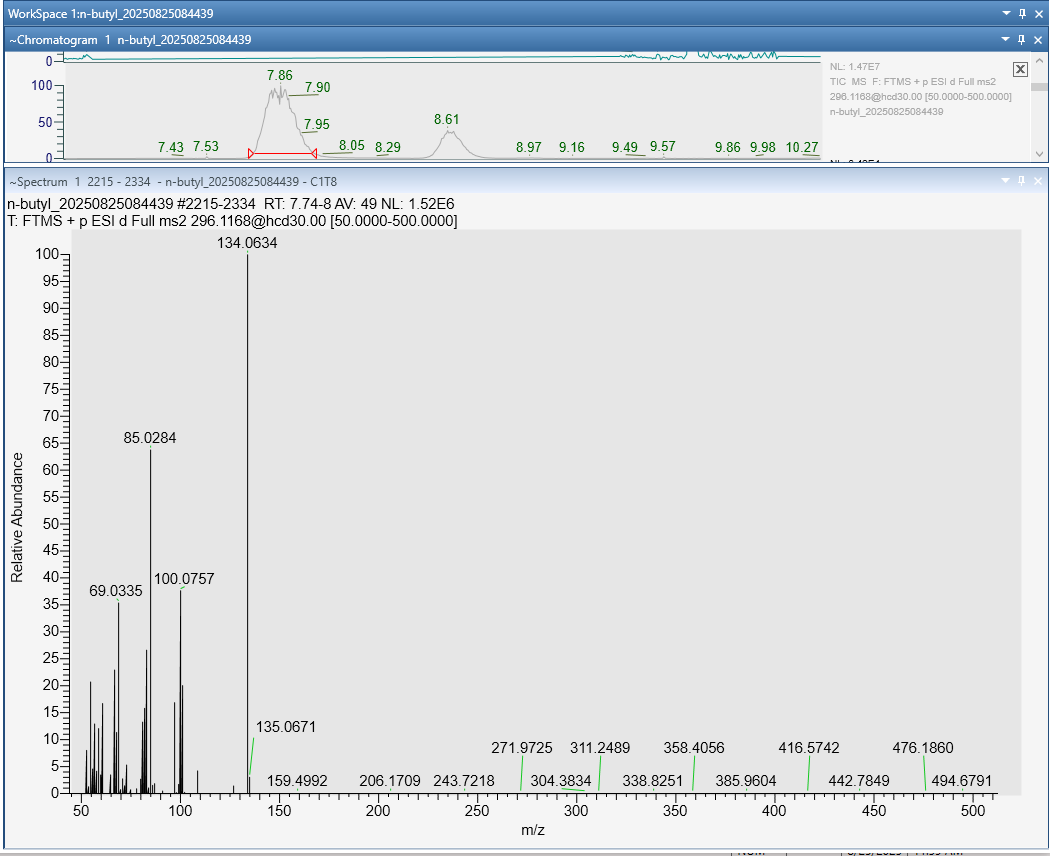


# ds 1-methylpropyl 296.1163


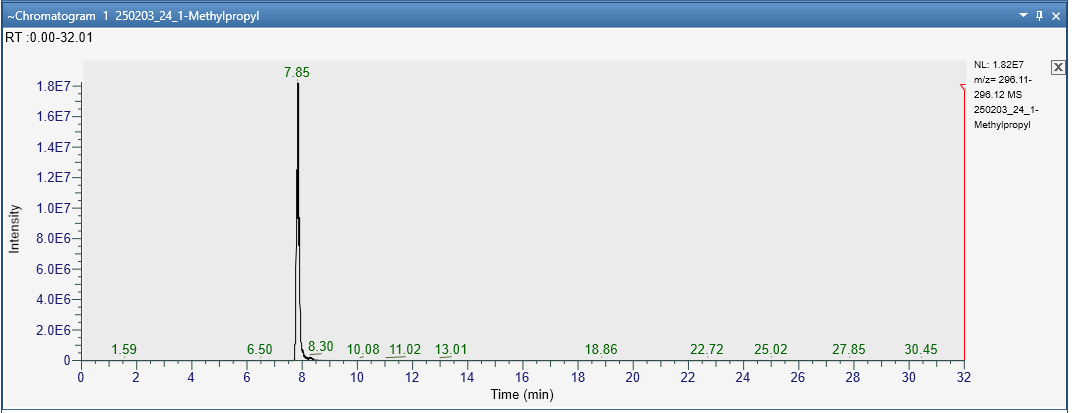


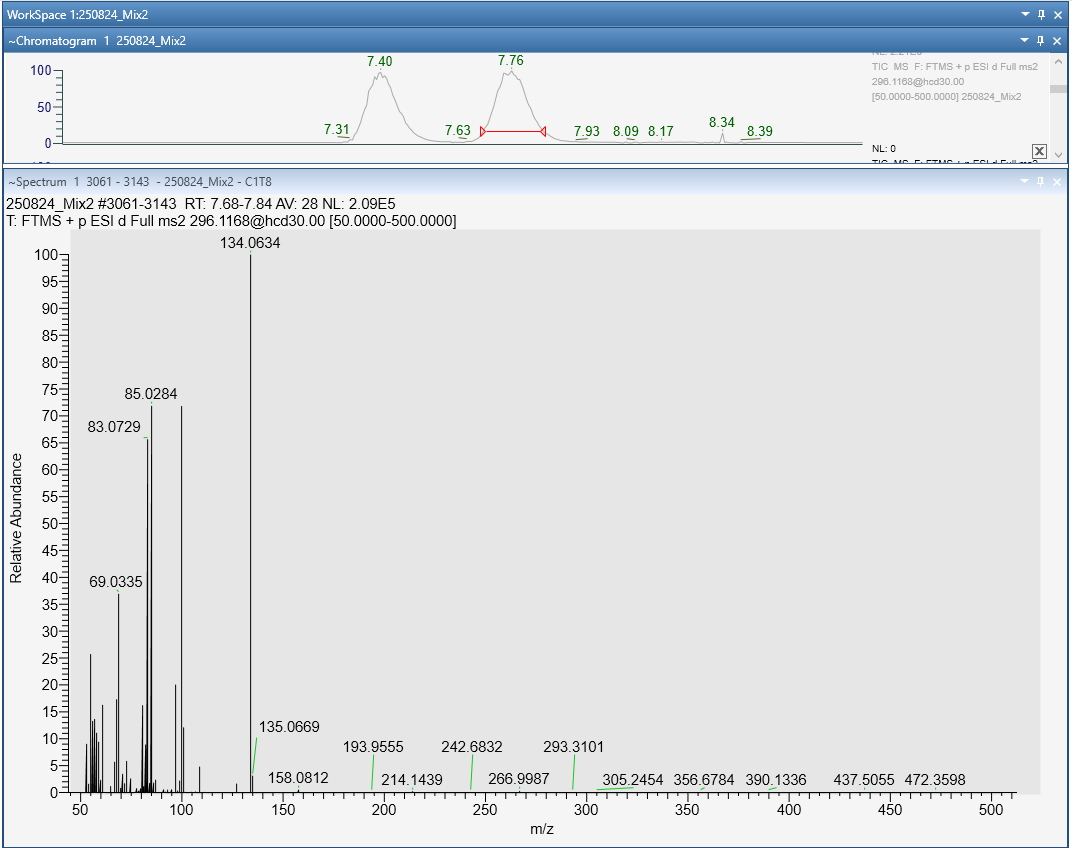


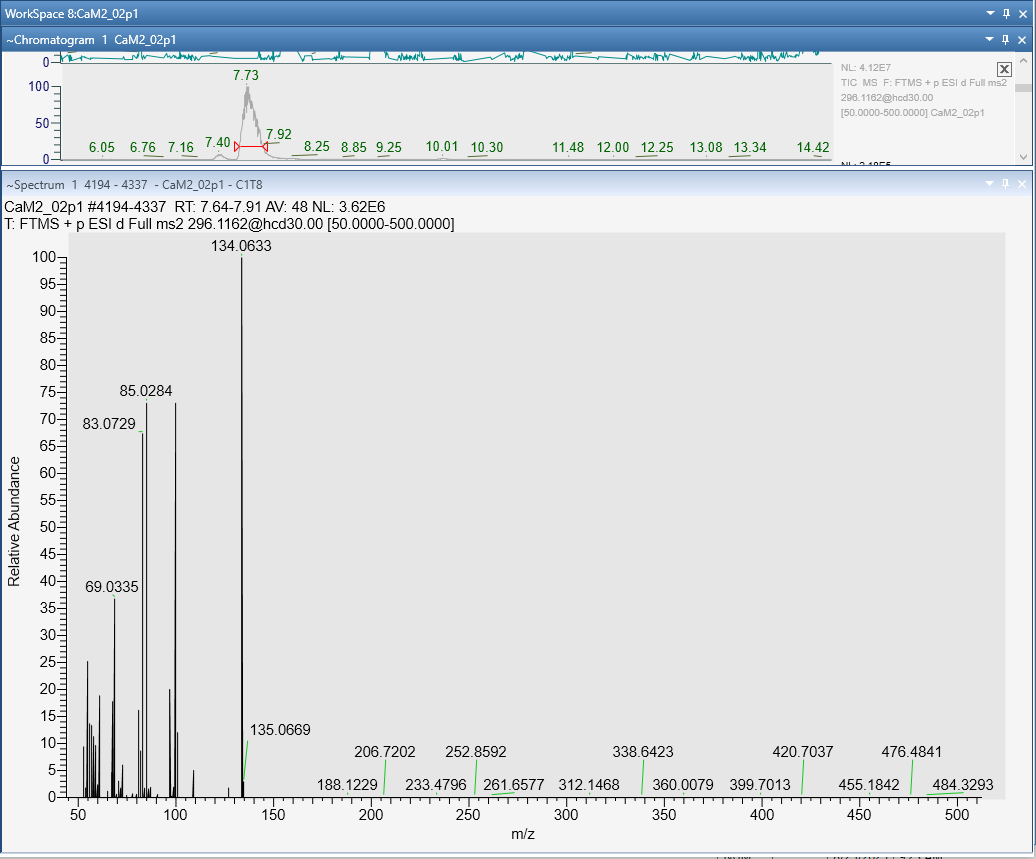


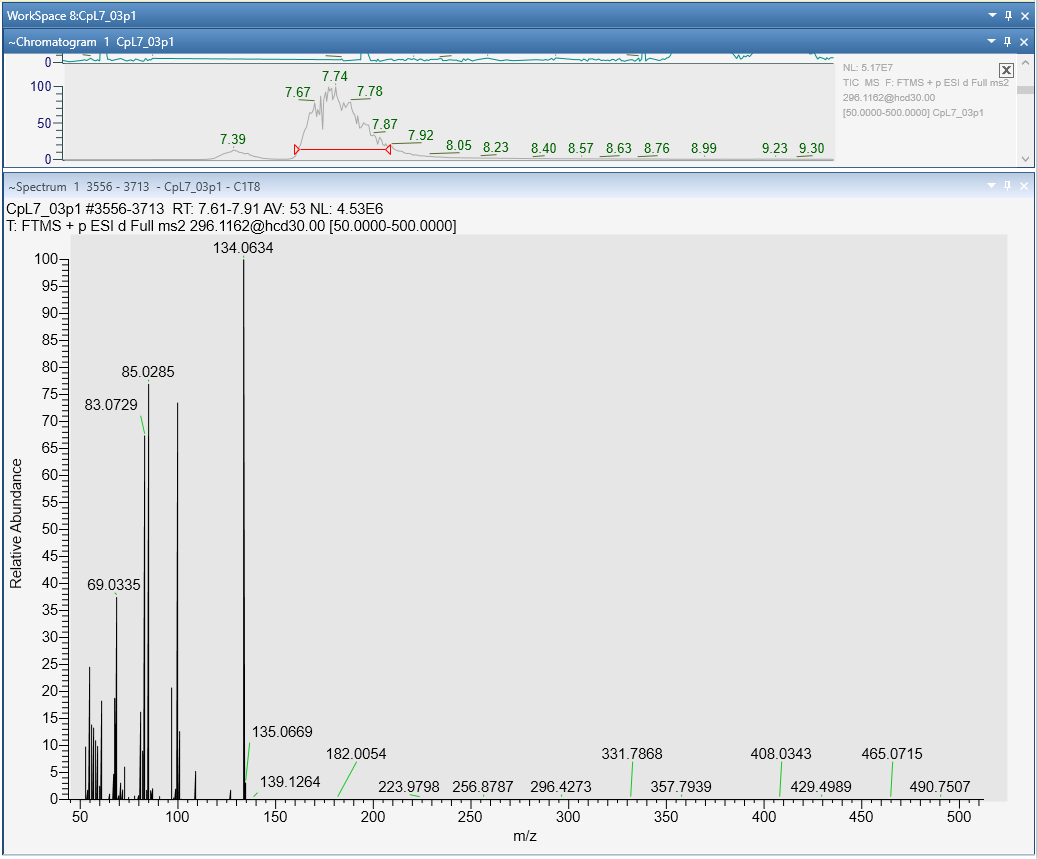


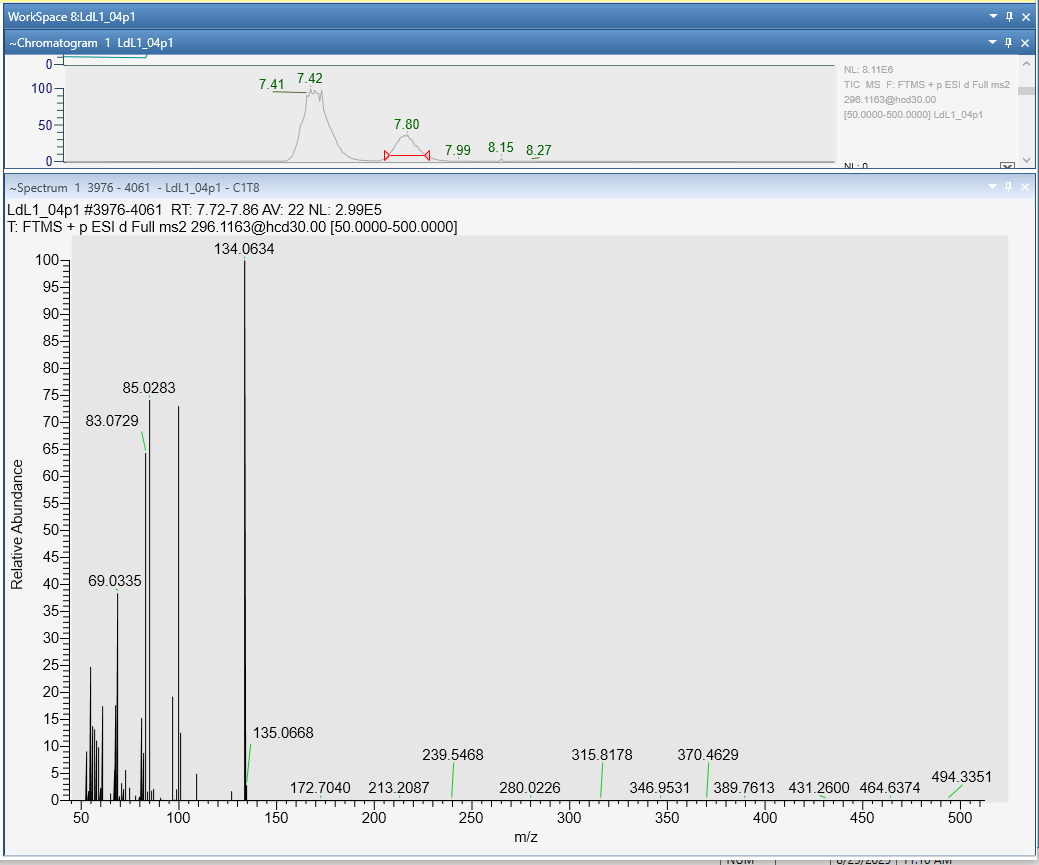


# ds 2-methylpropyl 296.1163


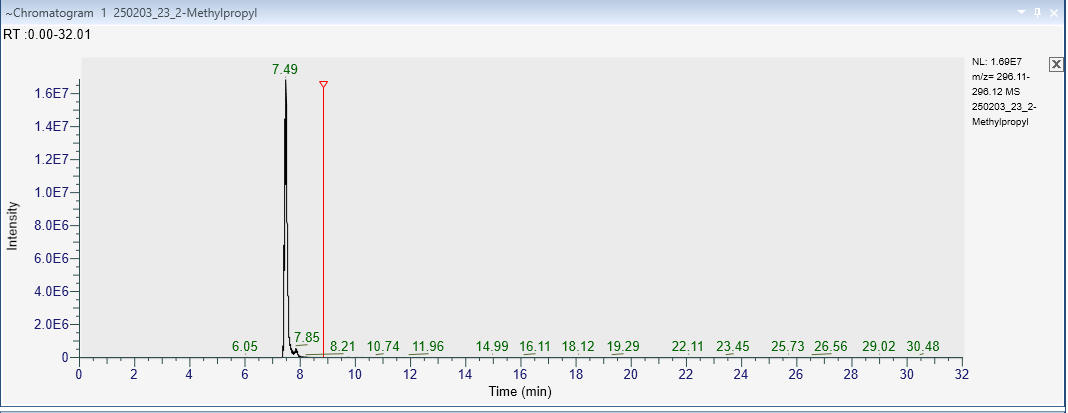


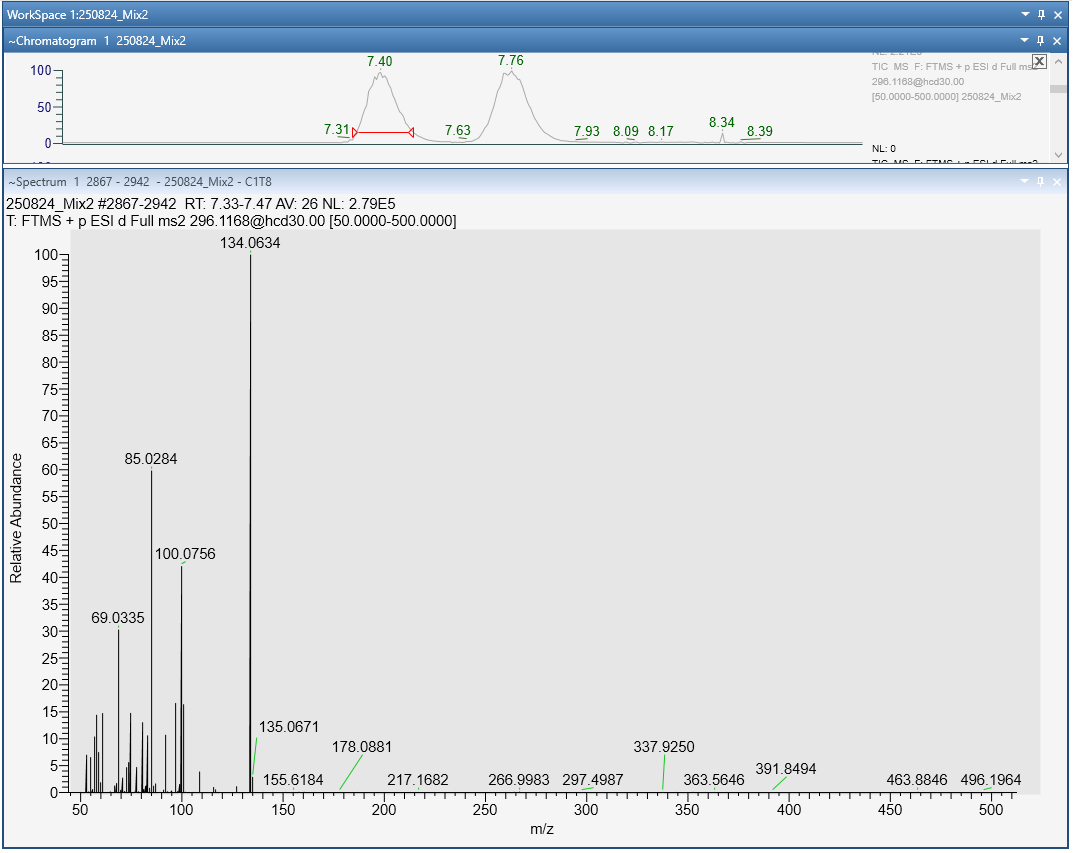


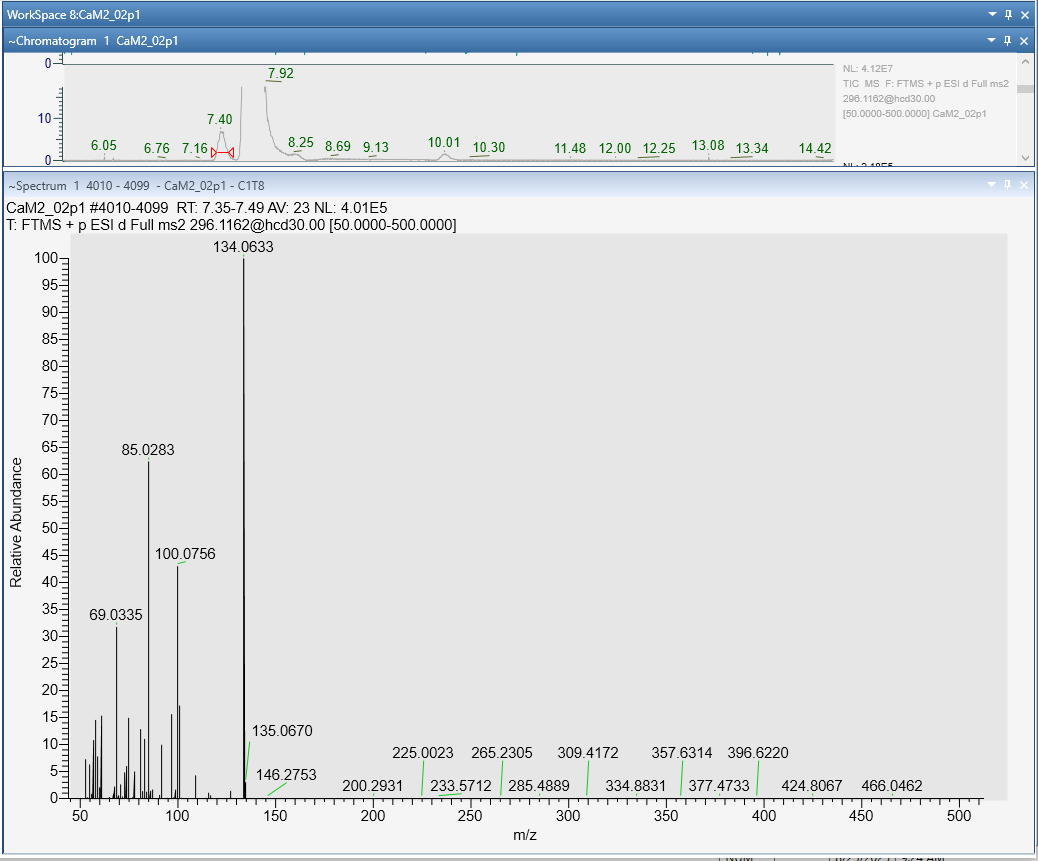


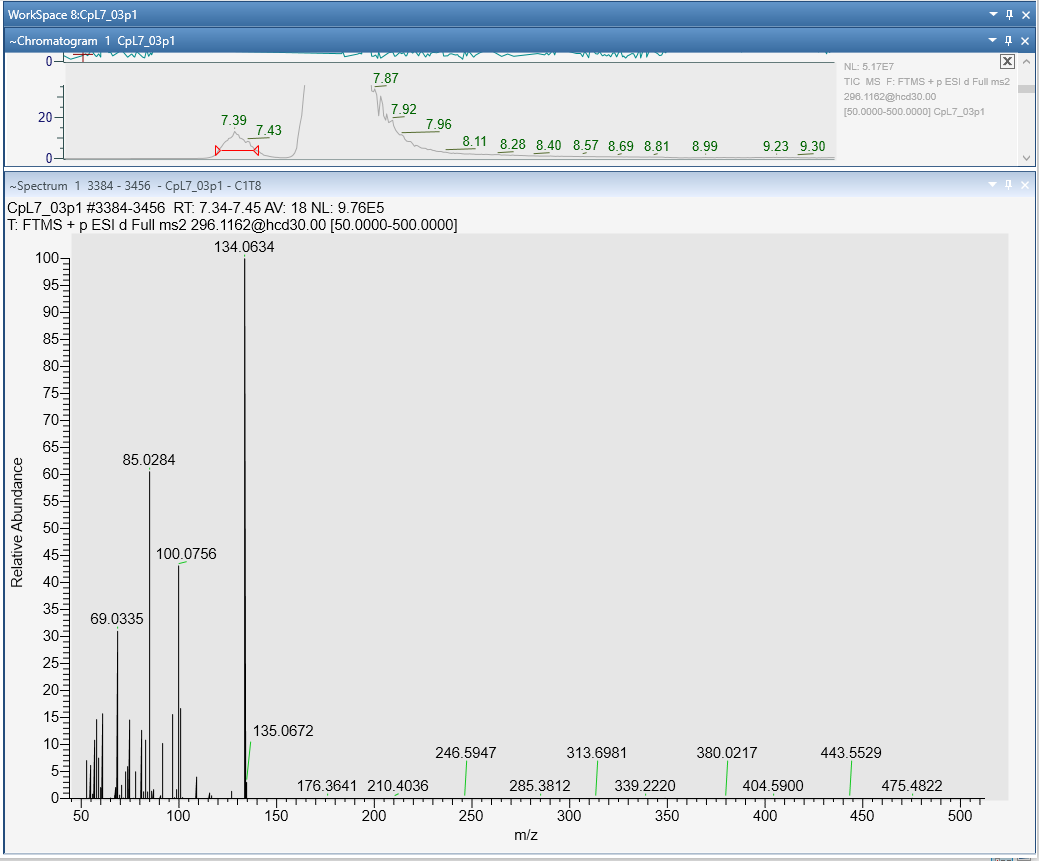


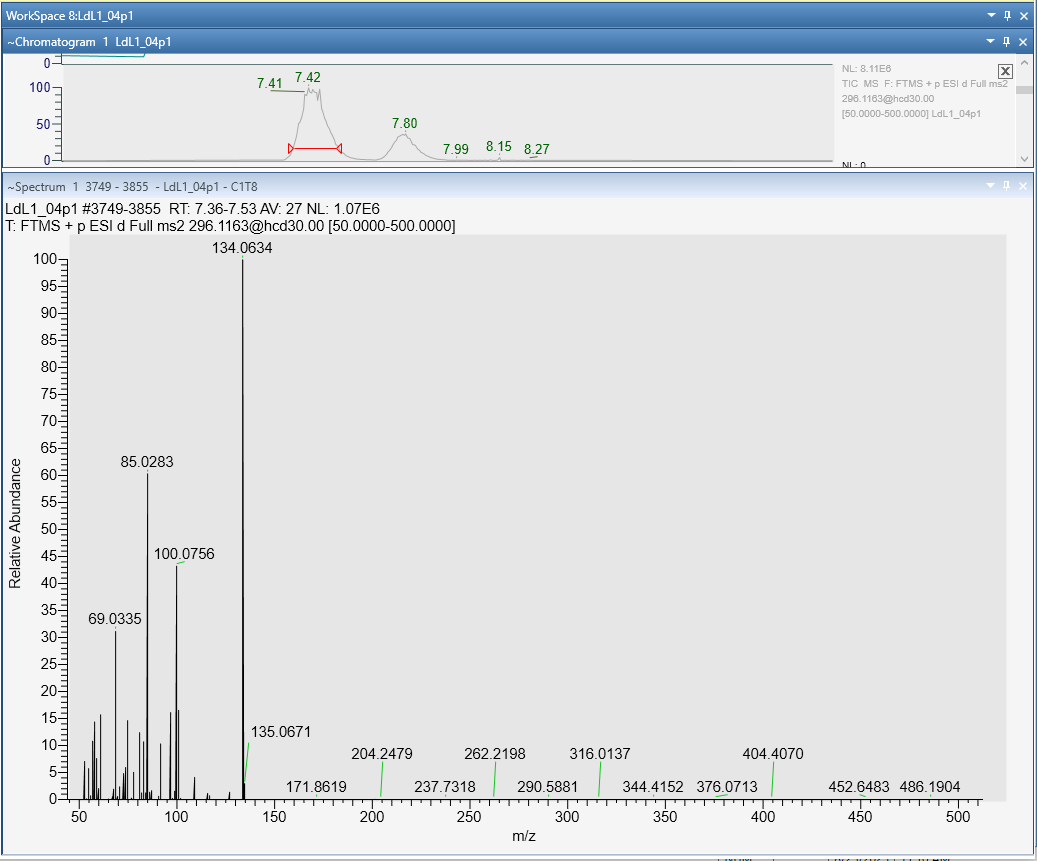


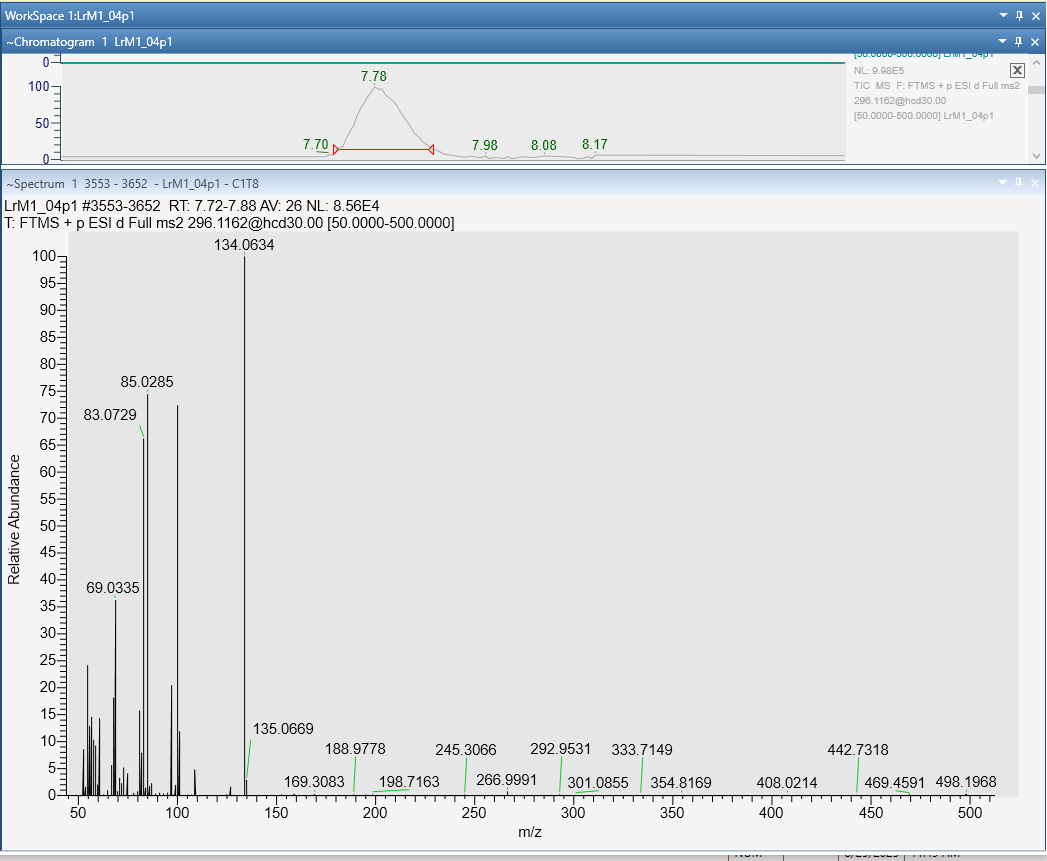


# ds 1-(hydroxymethyl)ethyl (#) 298.0955


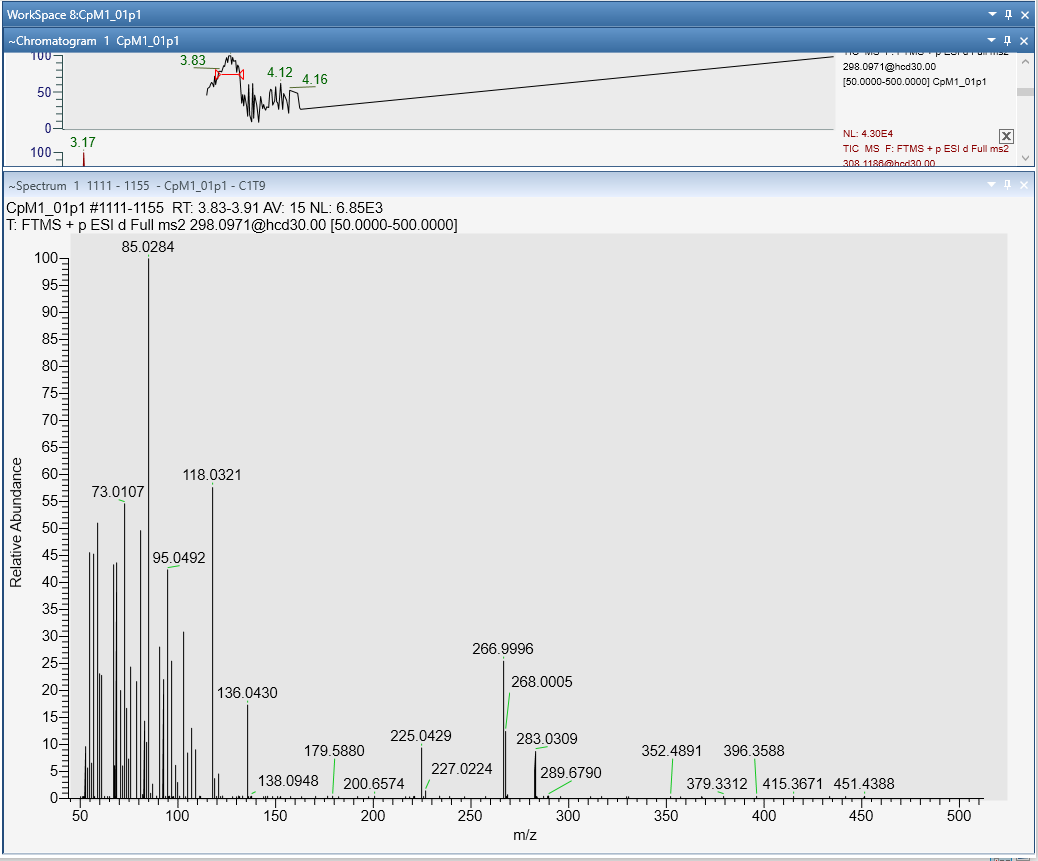


# ds 4-pentenyl 308.1163


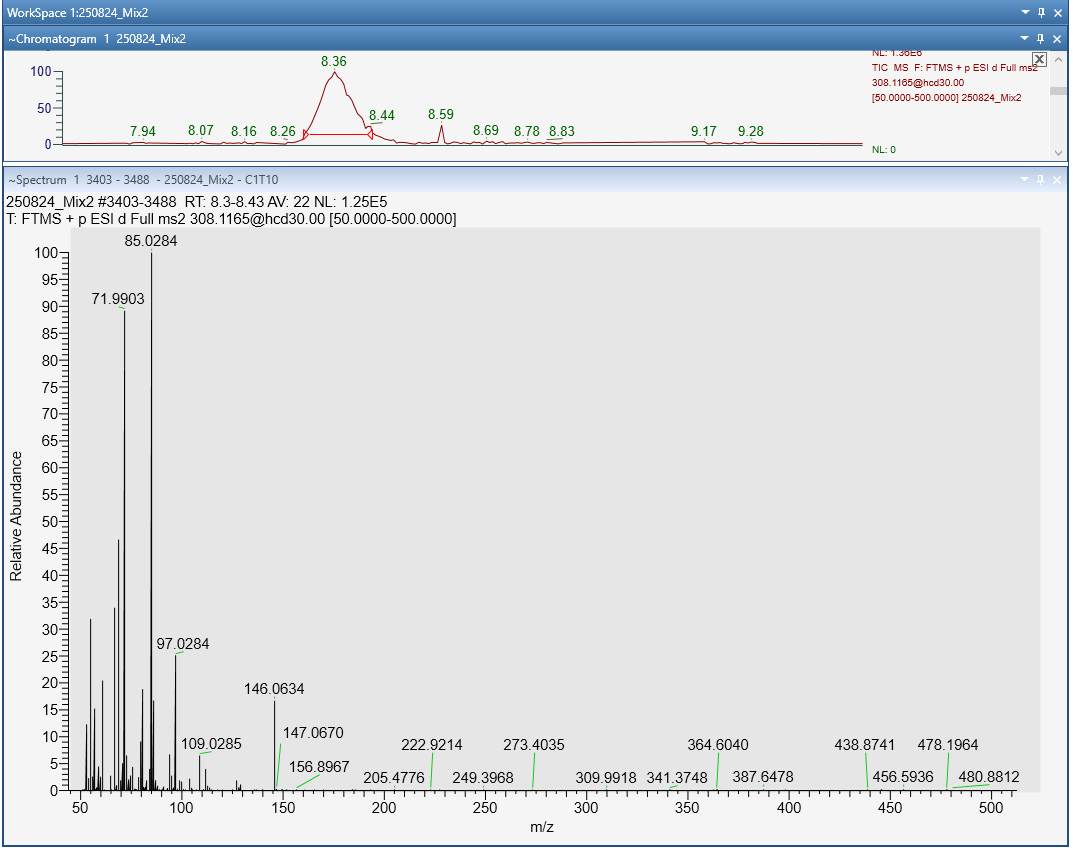


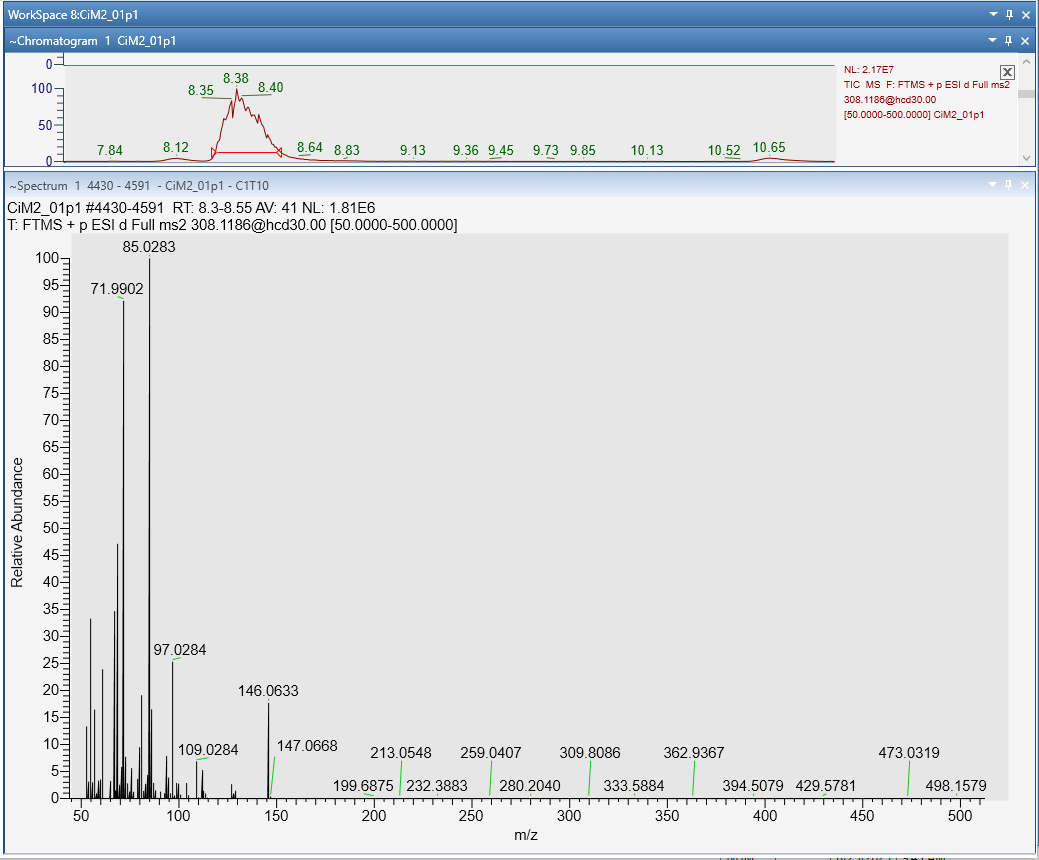


# ds 1-(hydroxymethyl)propyl (#) 312.1112


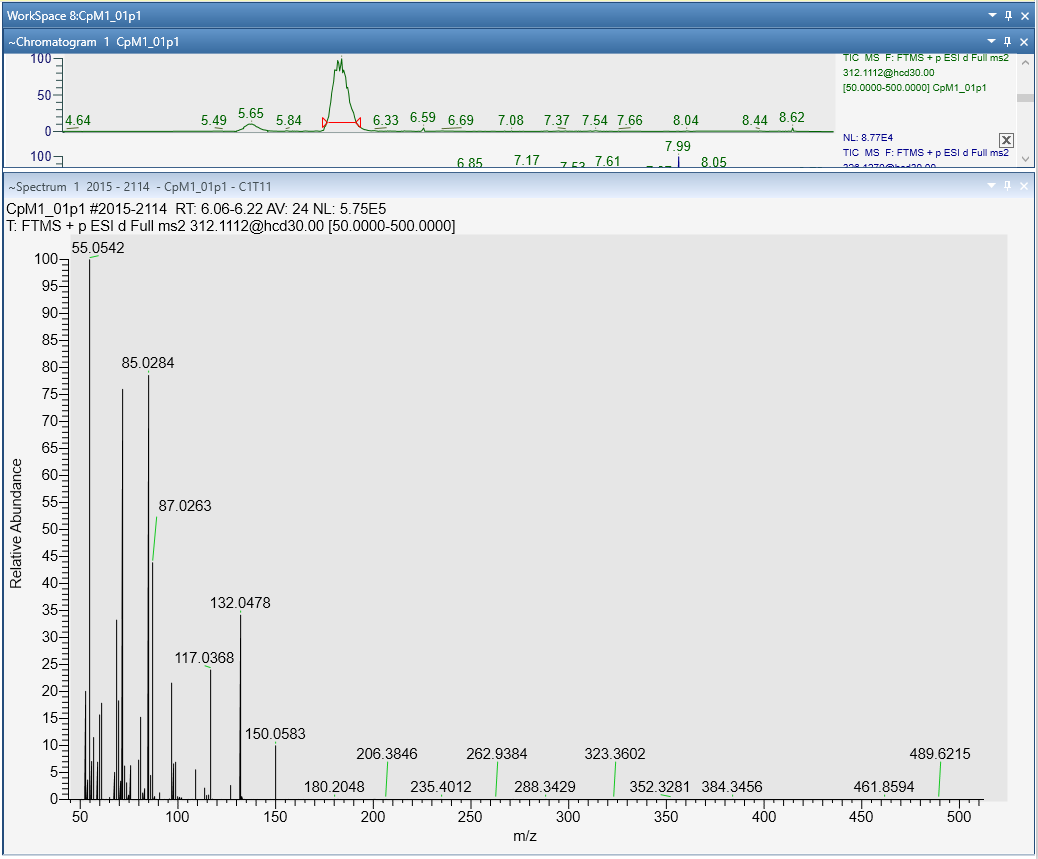


# ds 2-hydroxy-2-methylbutyl 326.1268


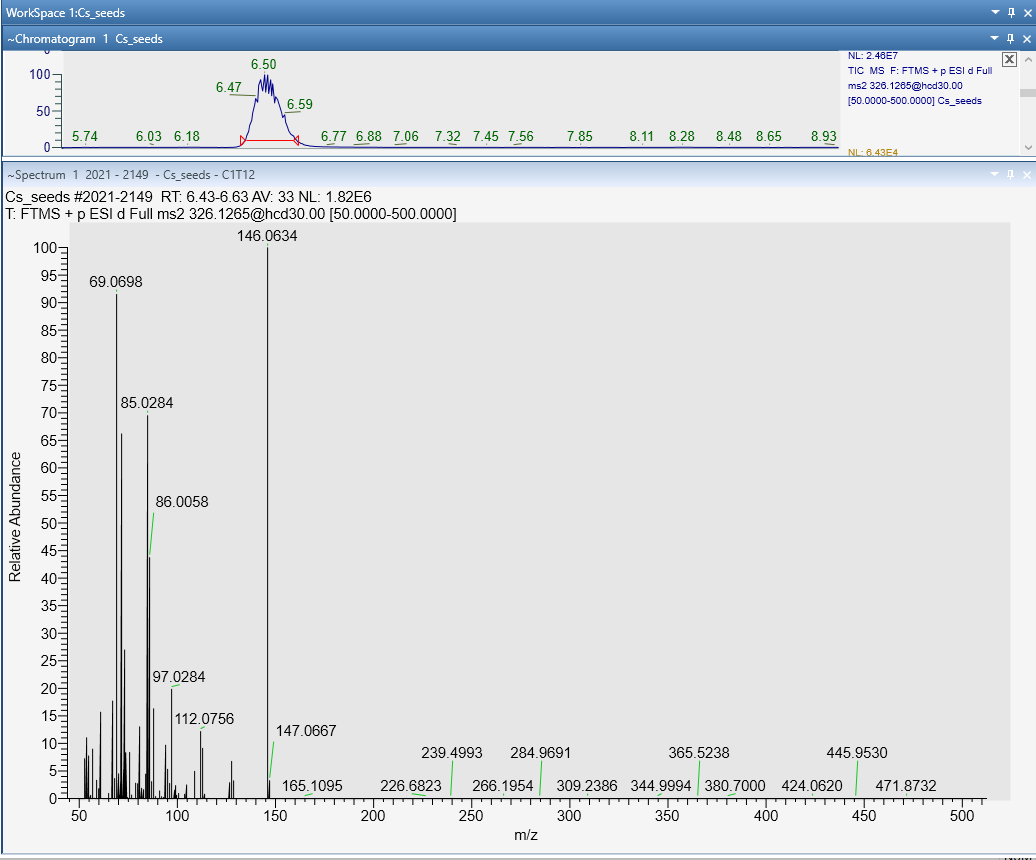


# unidentified ds hydroxypentyl or isomer (#) 326.1268


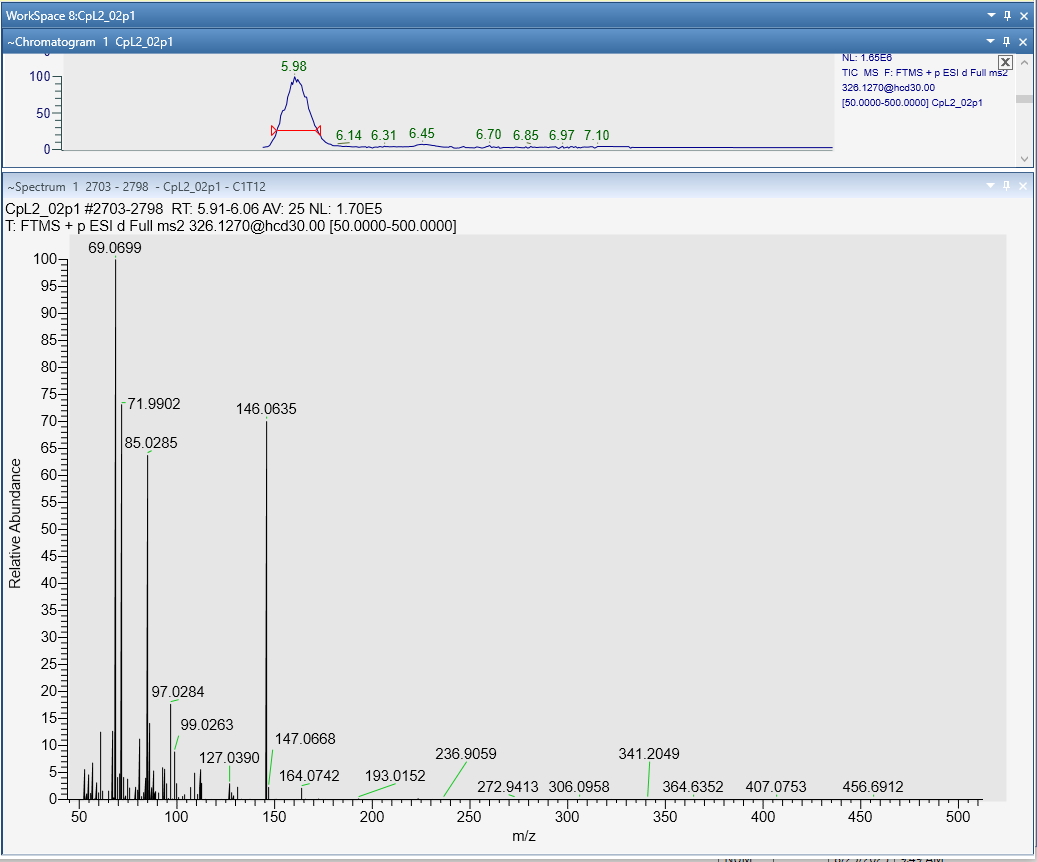

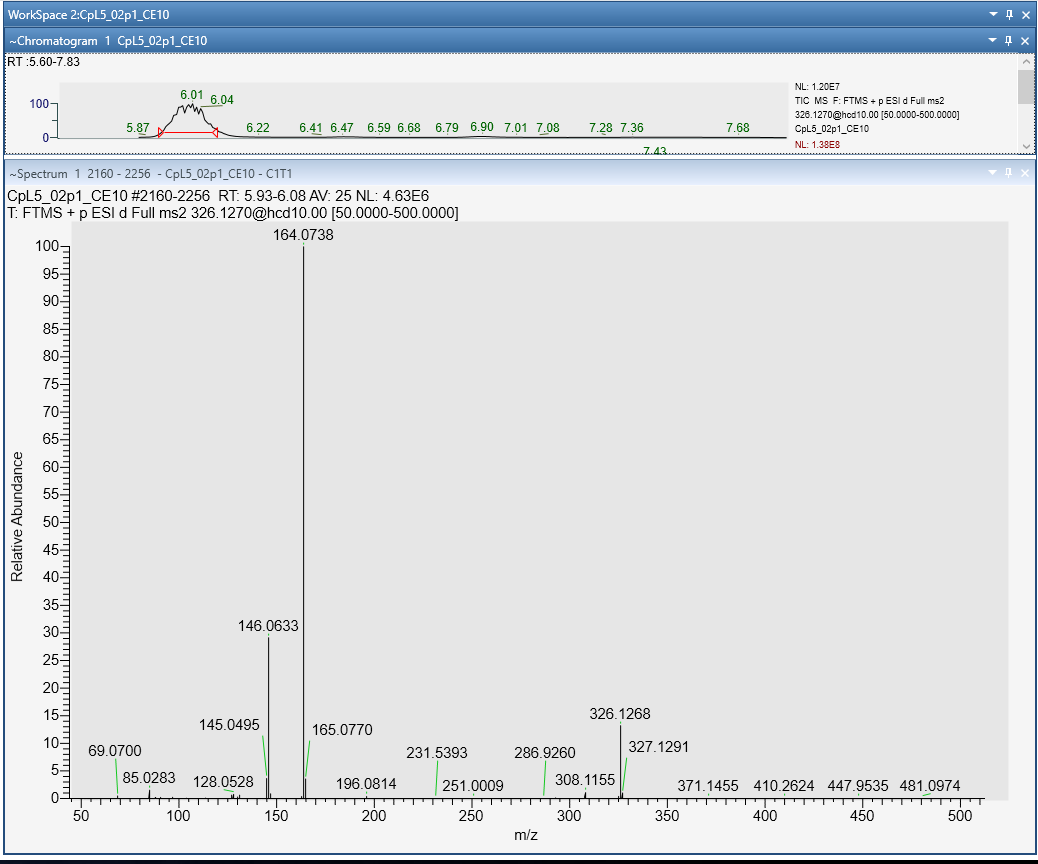


# ds benzyl 330.1006


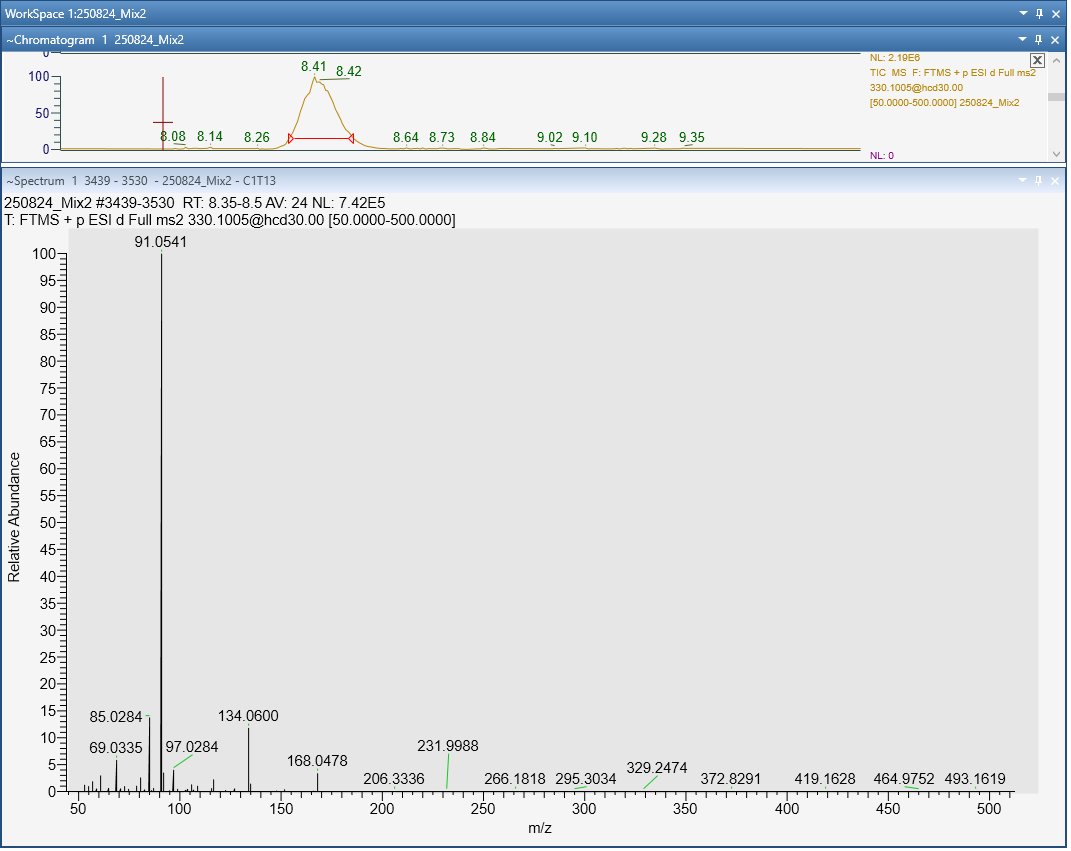


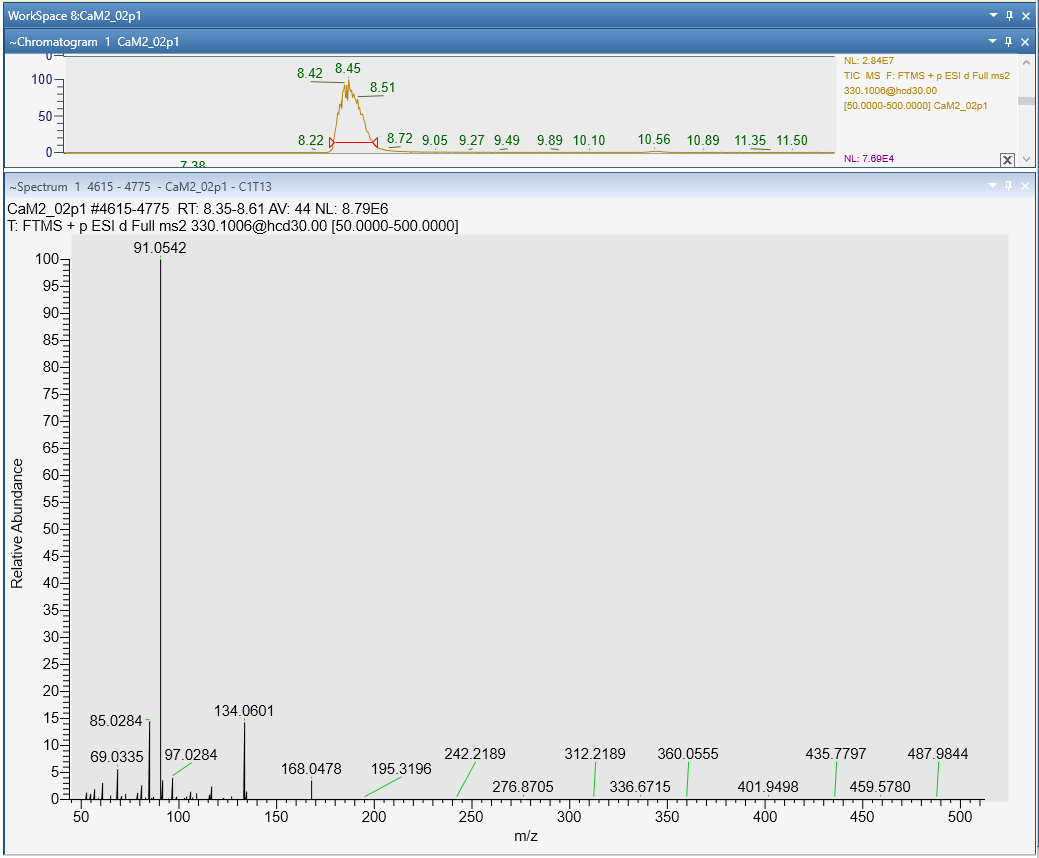


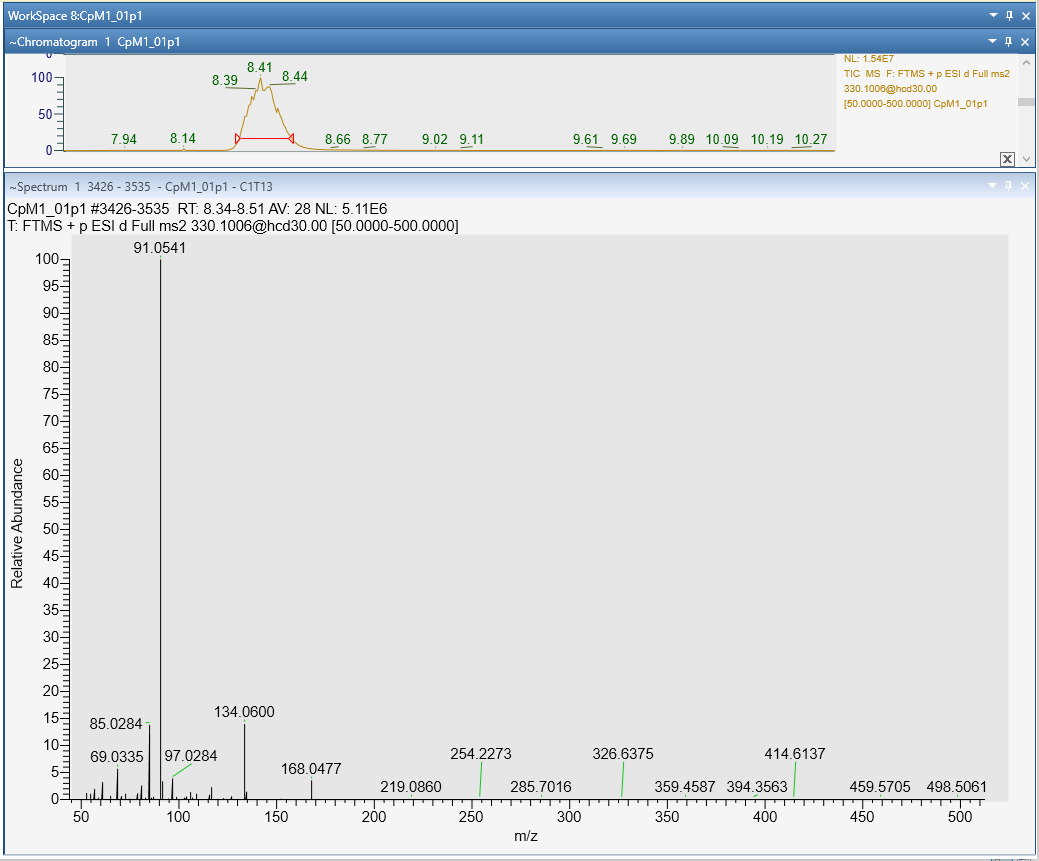


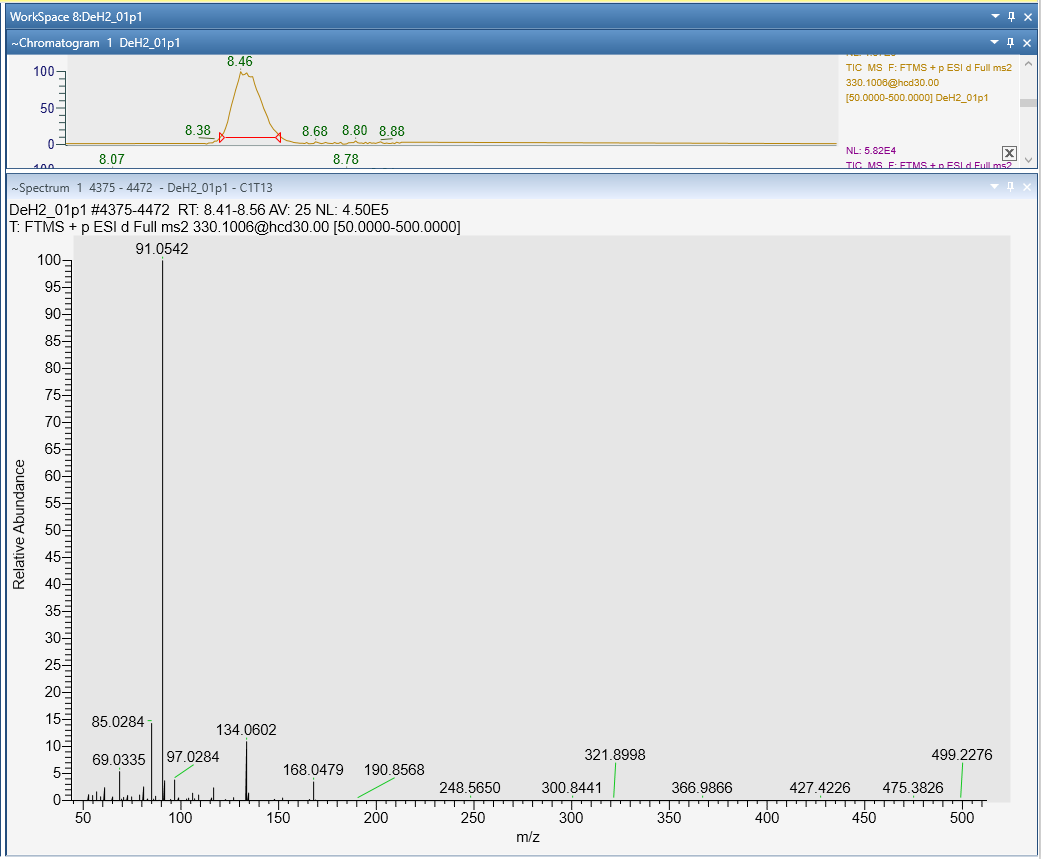


# ds 3-(hydroxymethyl)pentyl 340.1425


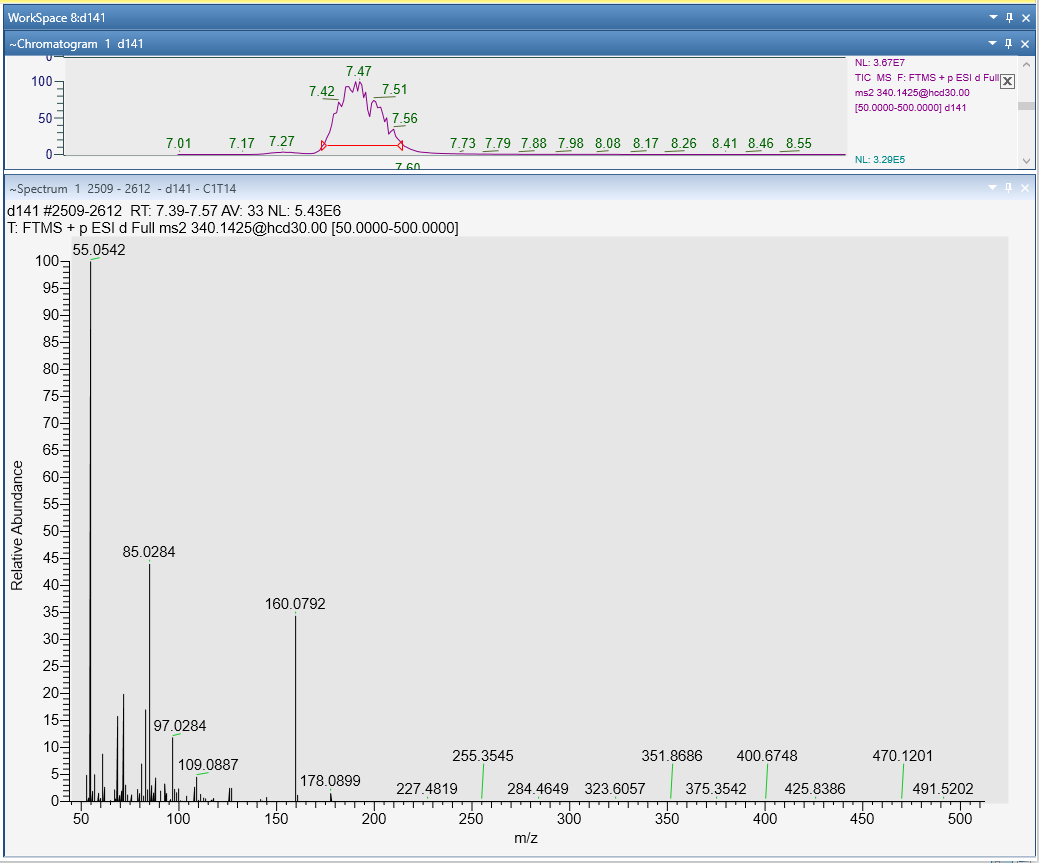


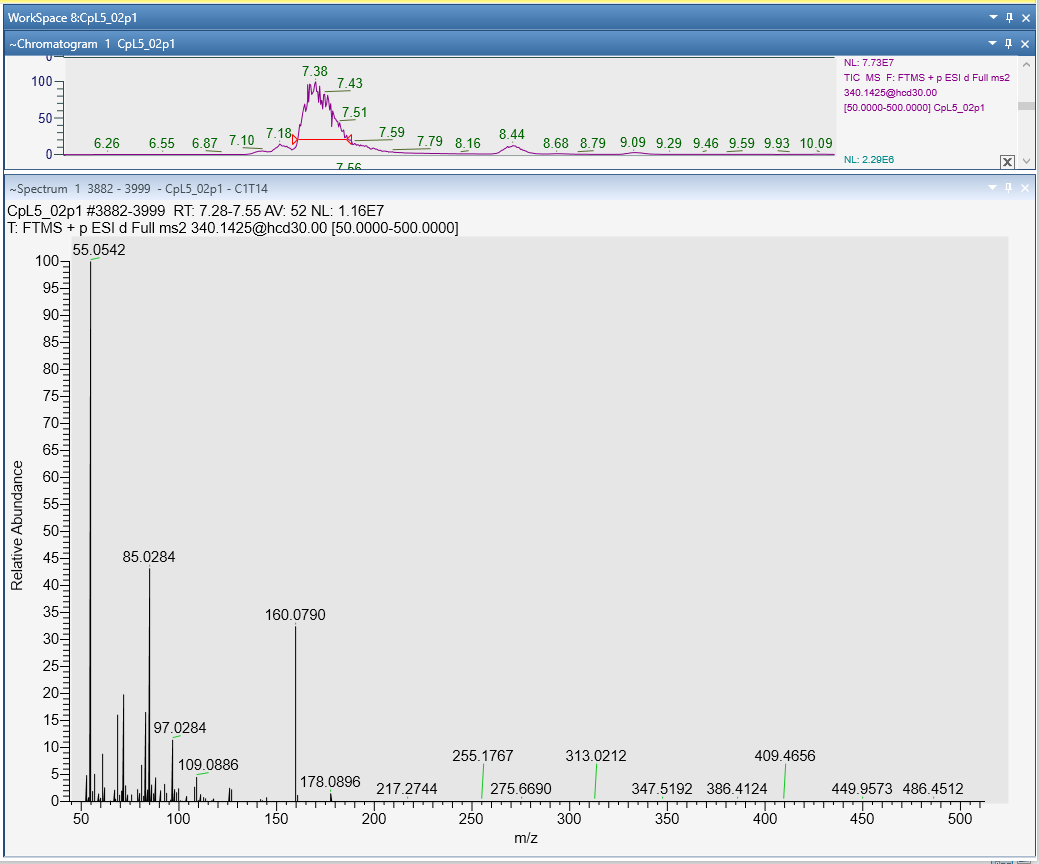

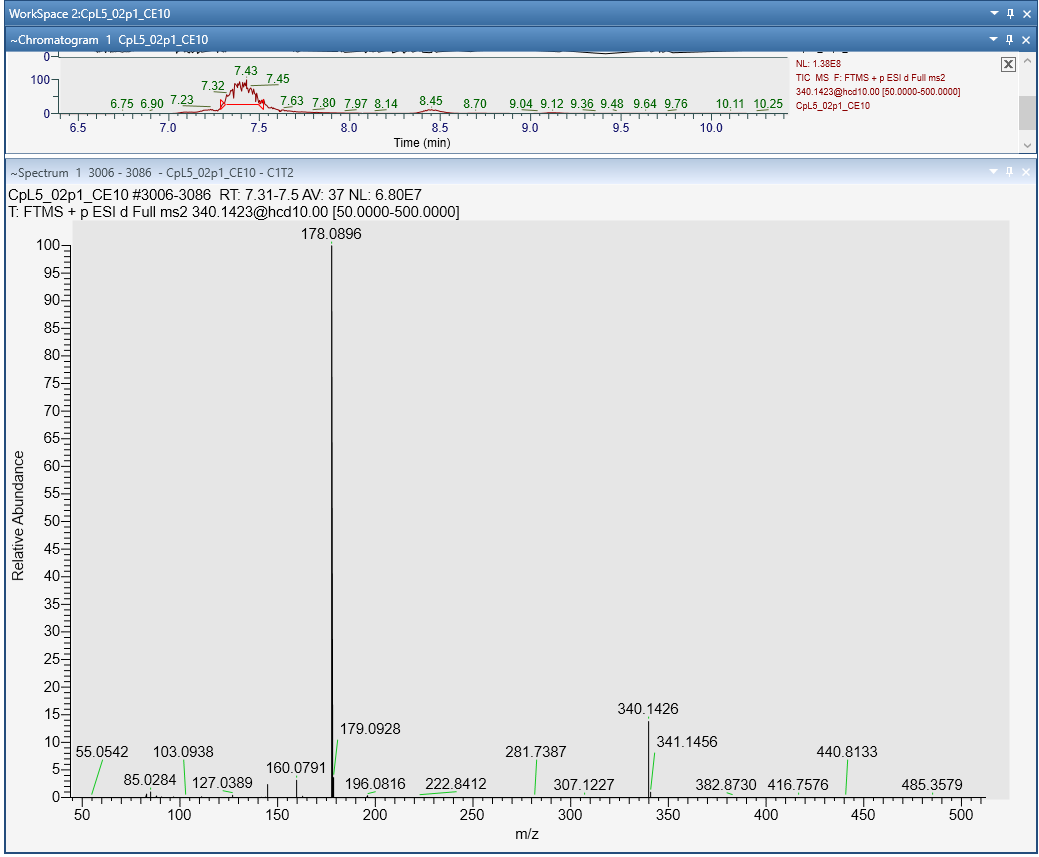


# ds 2-hydroxy-3-methylpentyl 340.1425


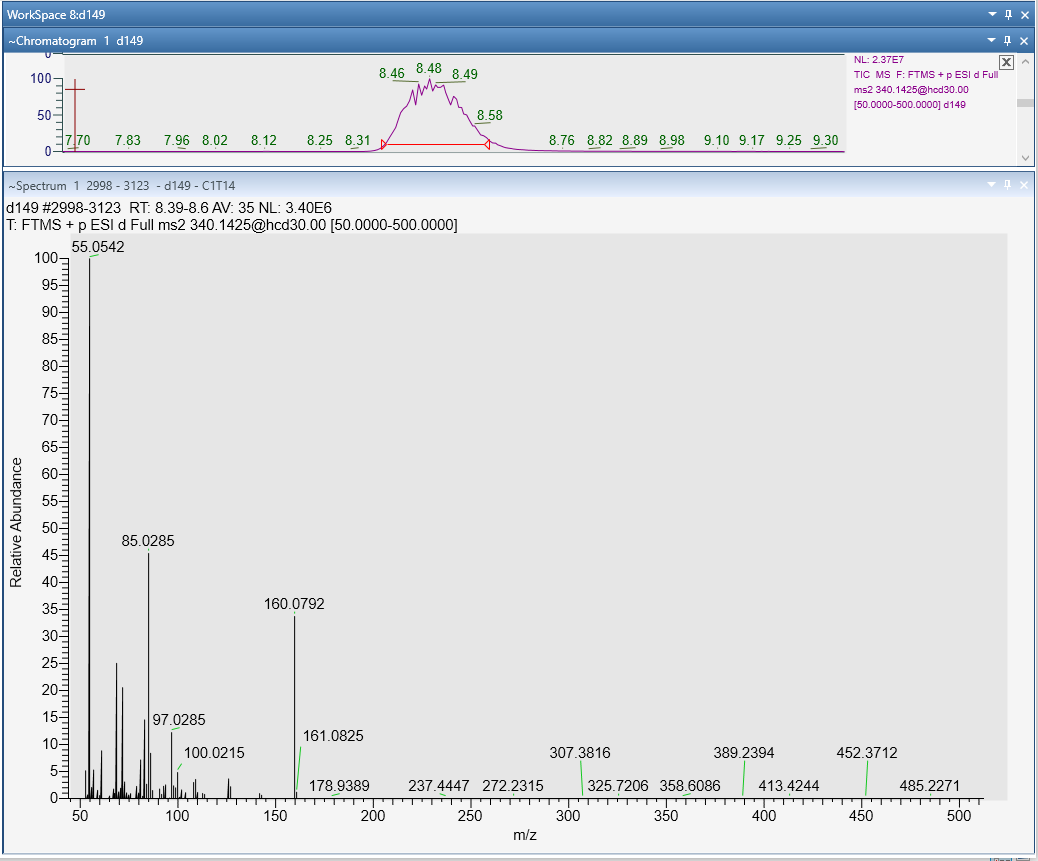


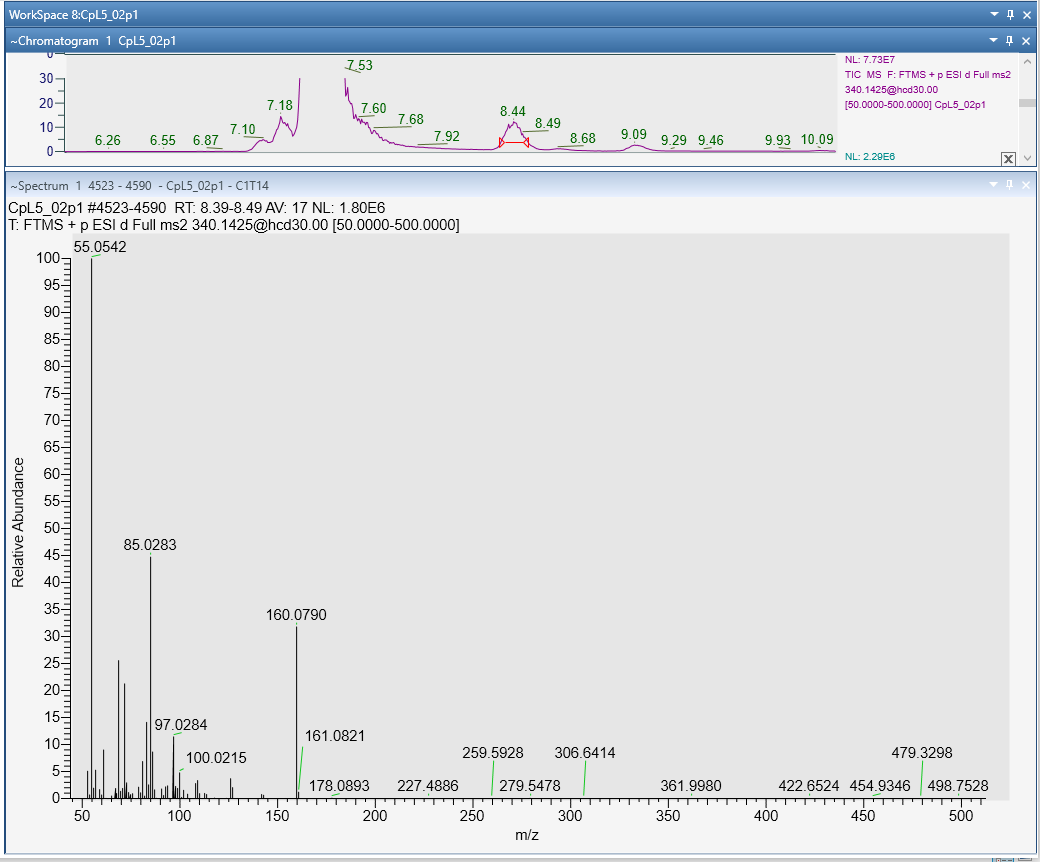

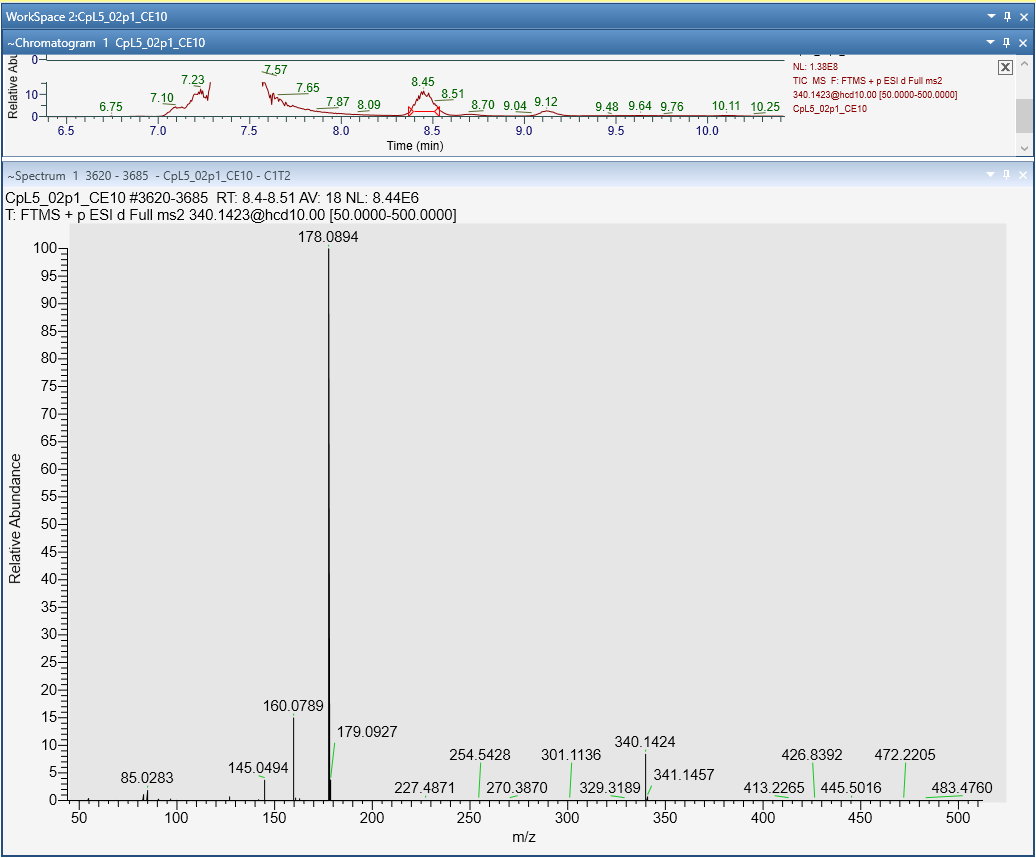


# ds 4-(methylthio)butyl 342.104


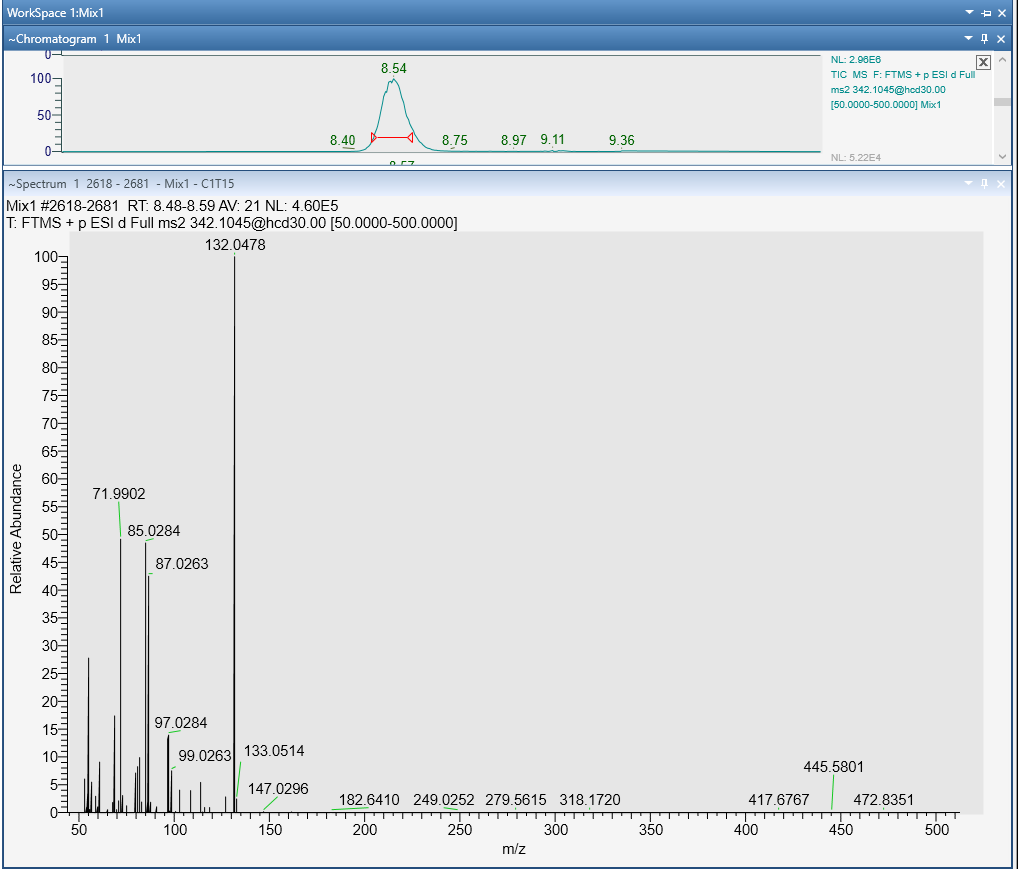


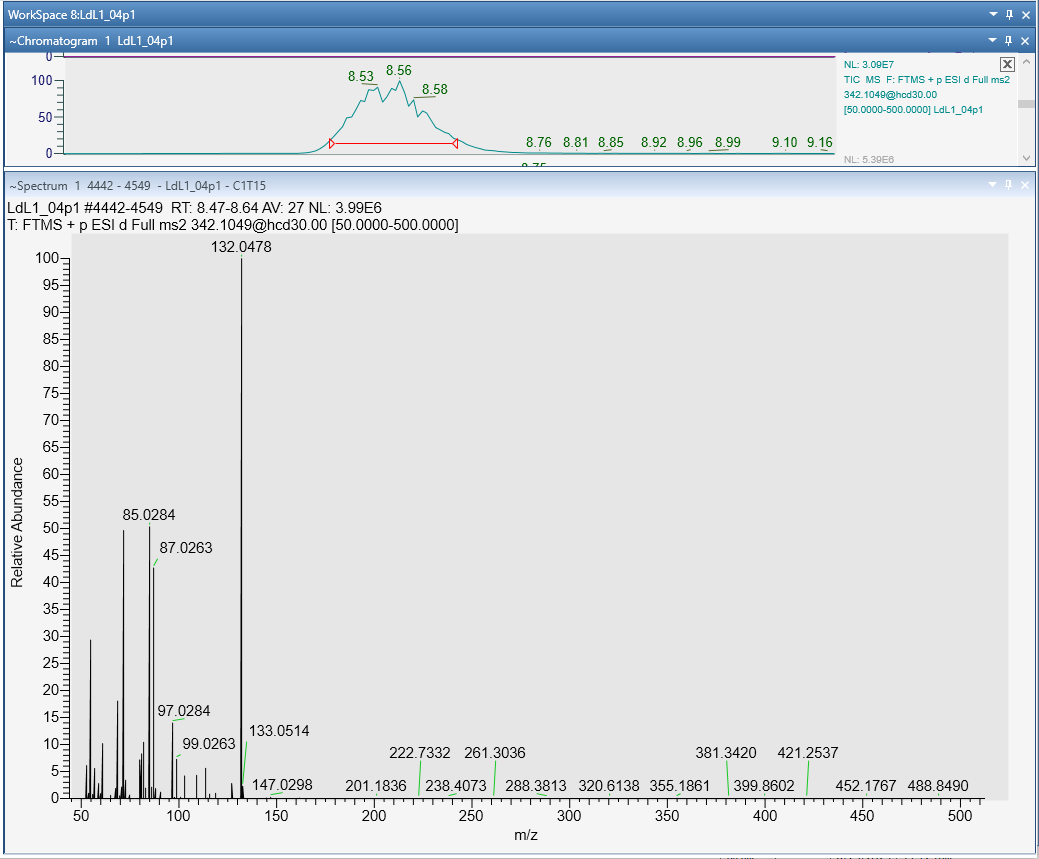


# ds 4-hydroxybenzyl 346.0955


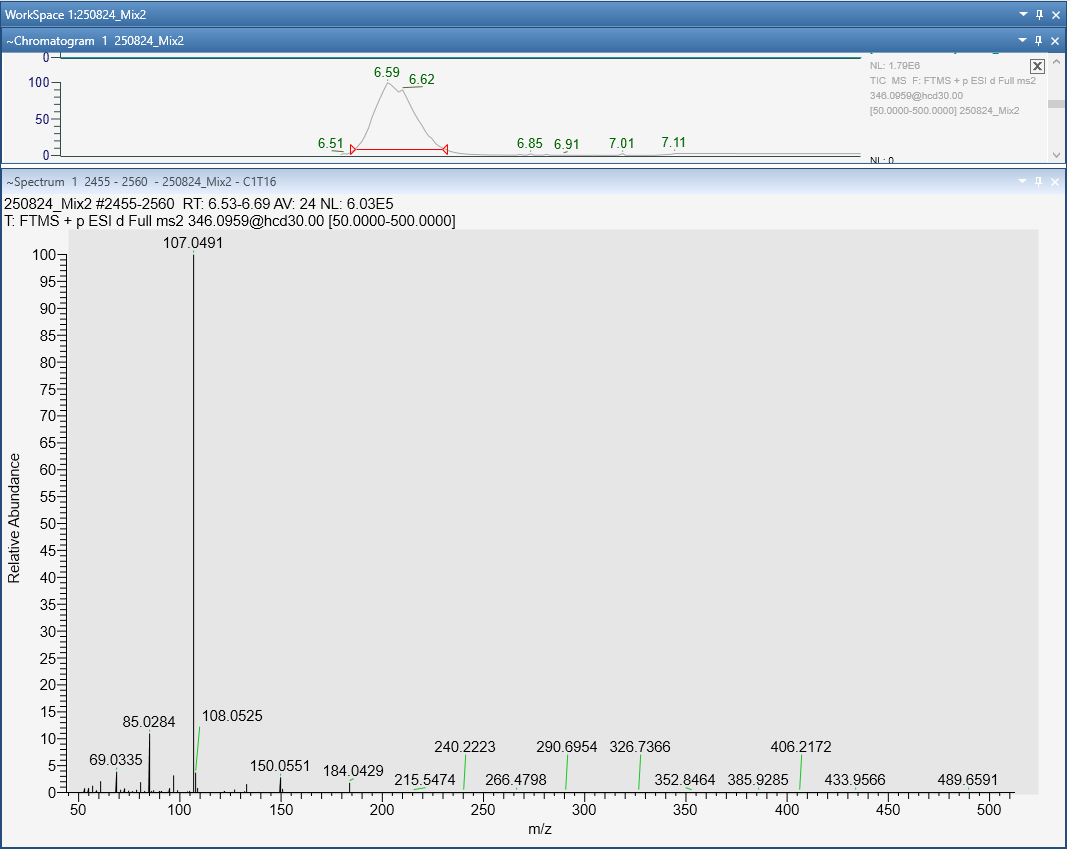


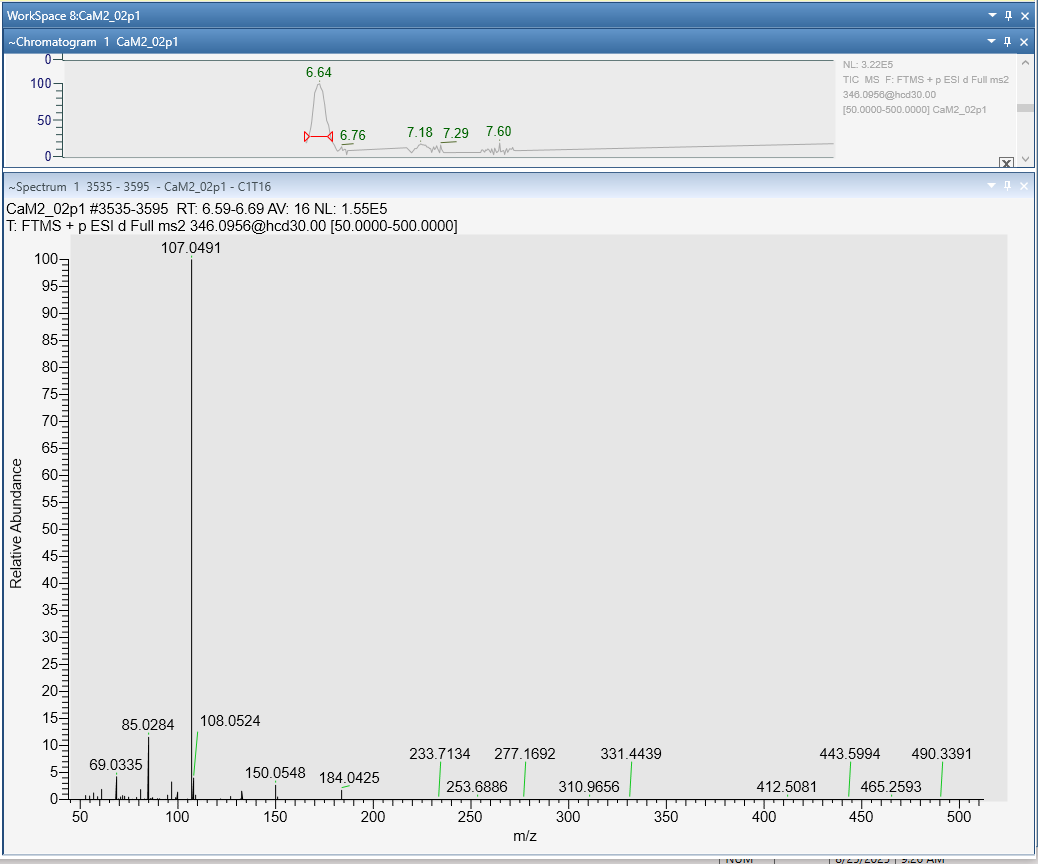


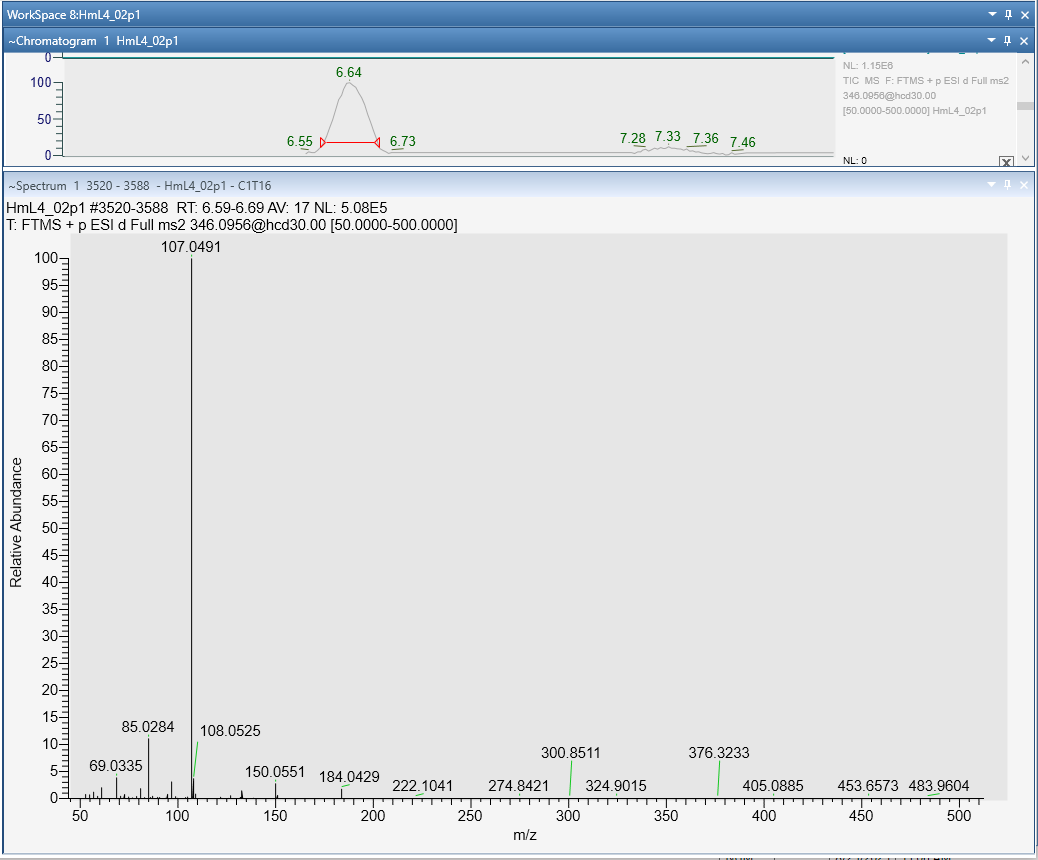


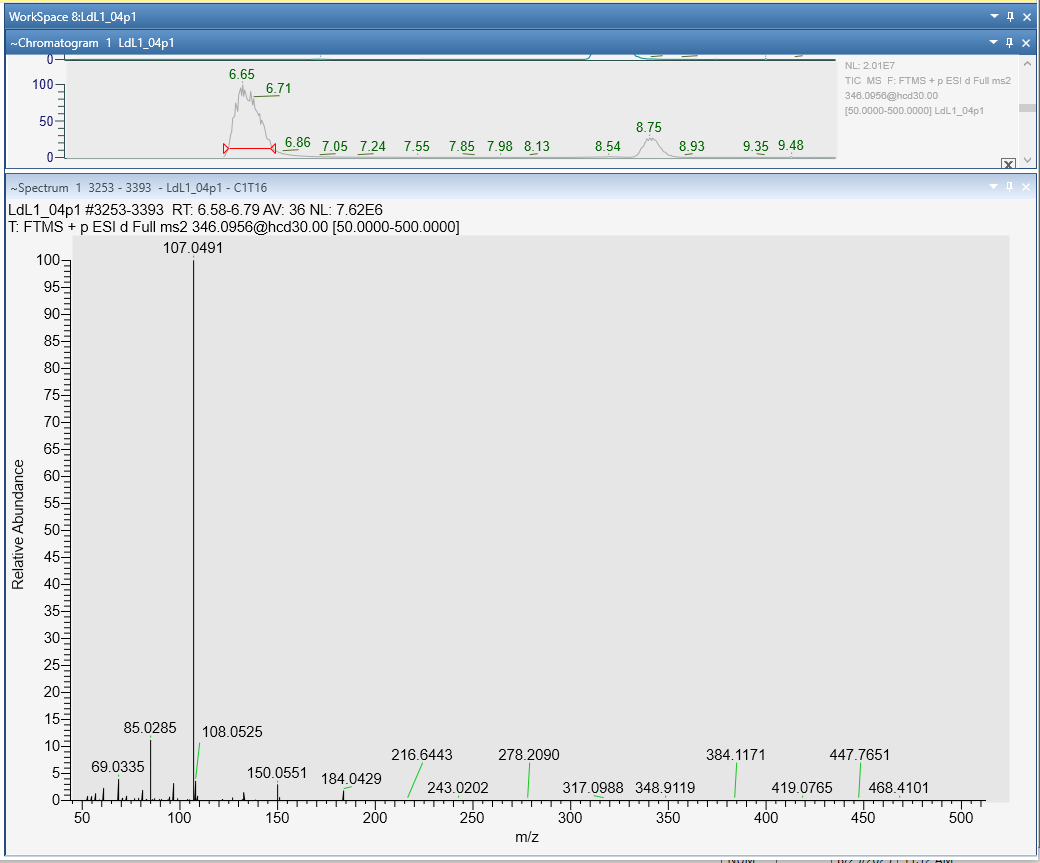


# ds 5-(methylthio)pentyl (#) 356.1197


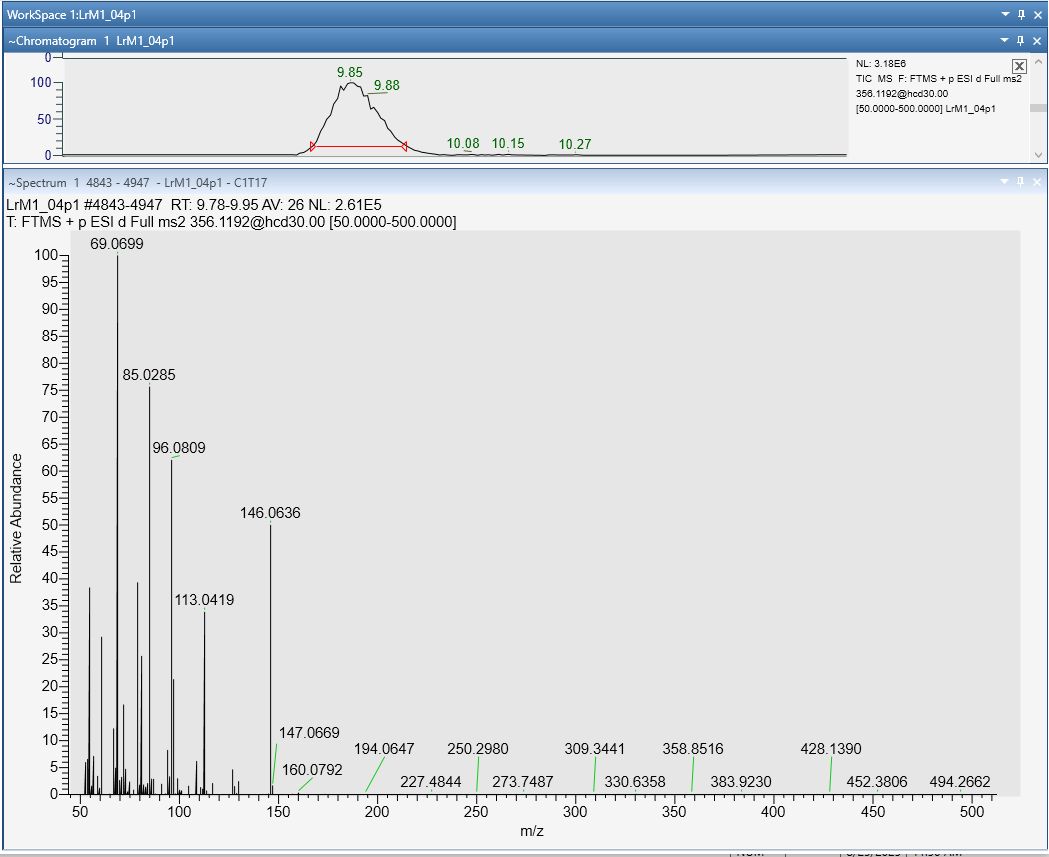


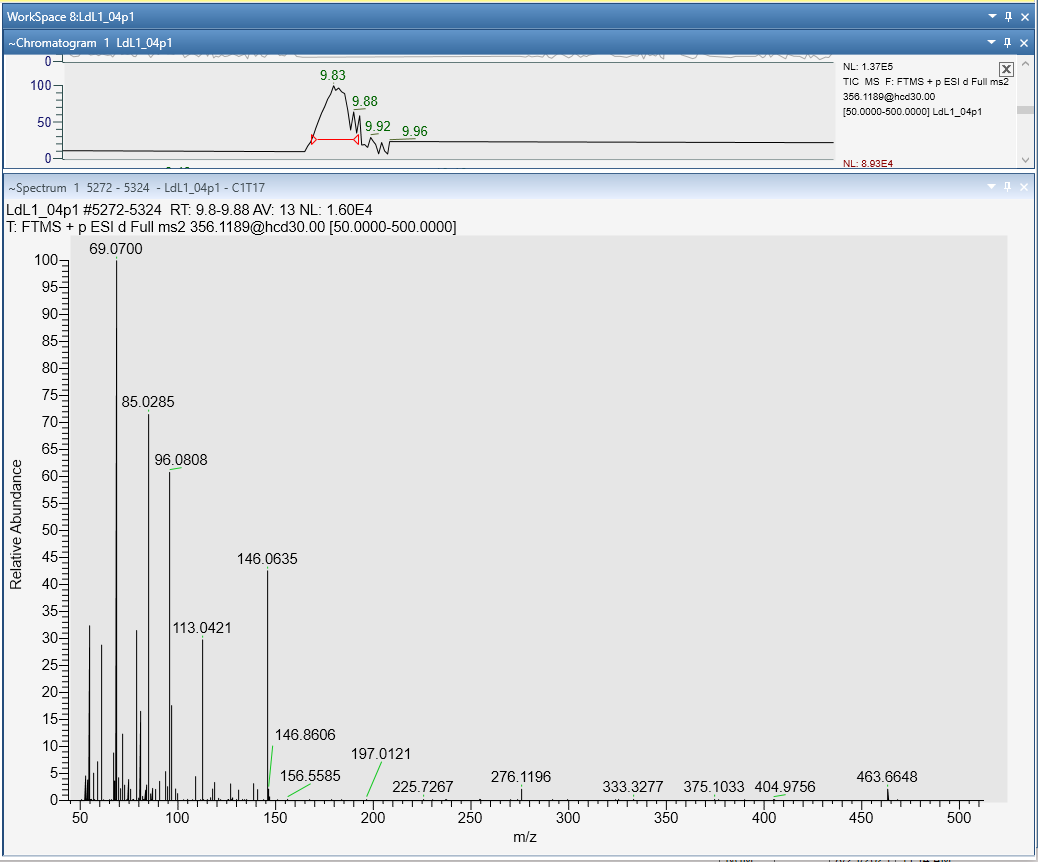


# ds 4-(methylsulfinyl)butyl 358.0989


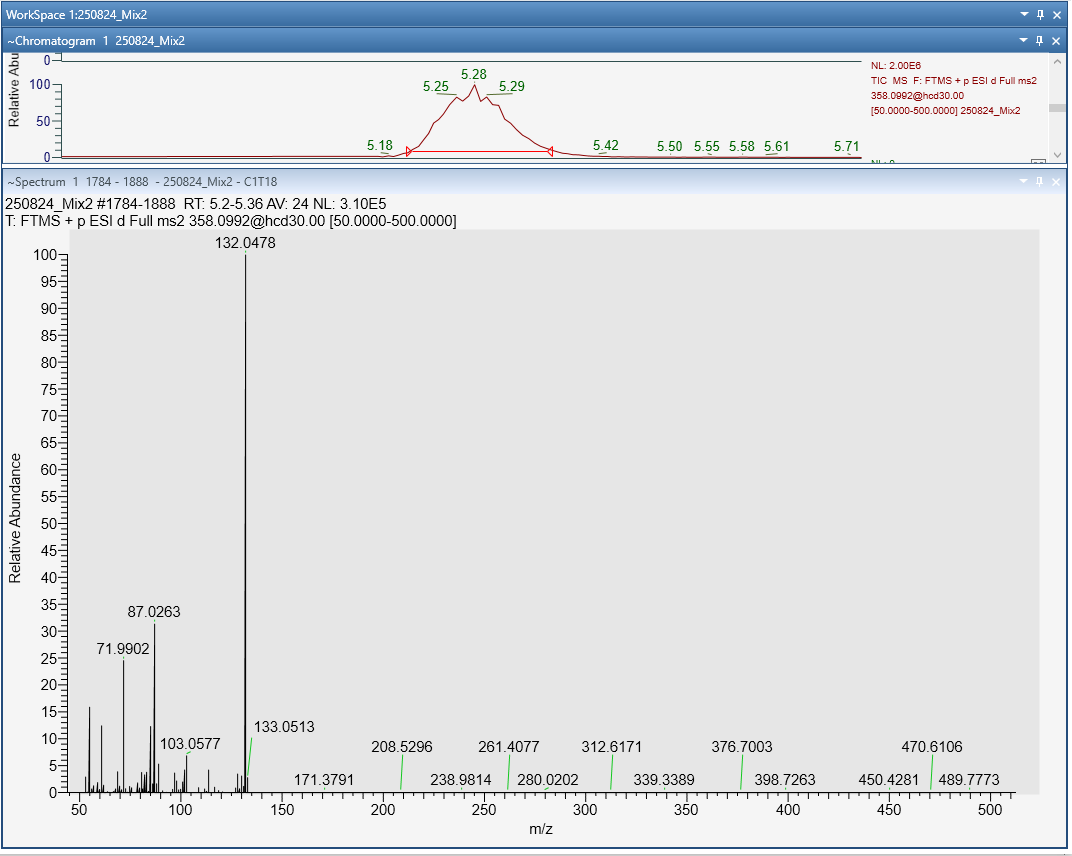


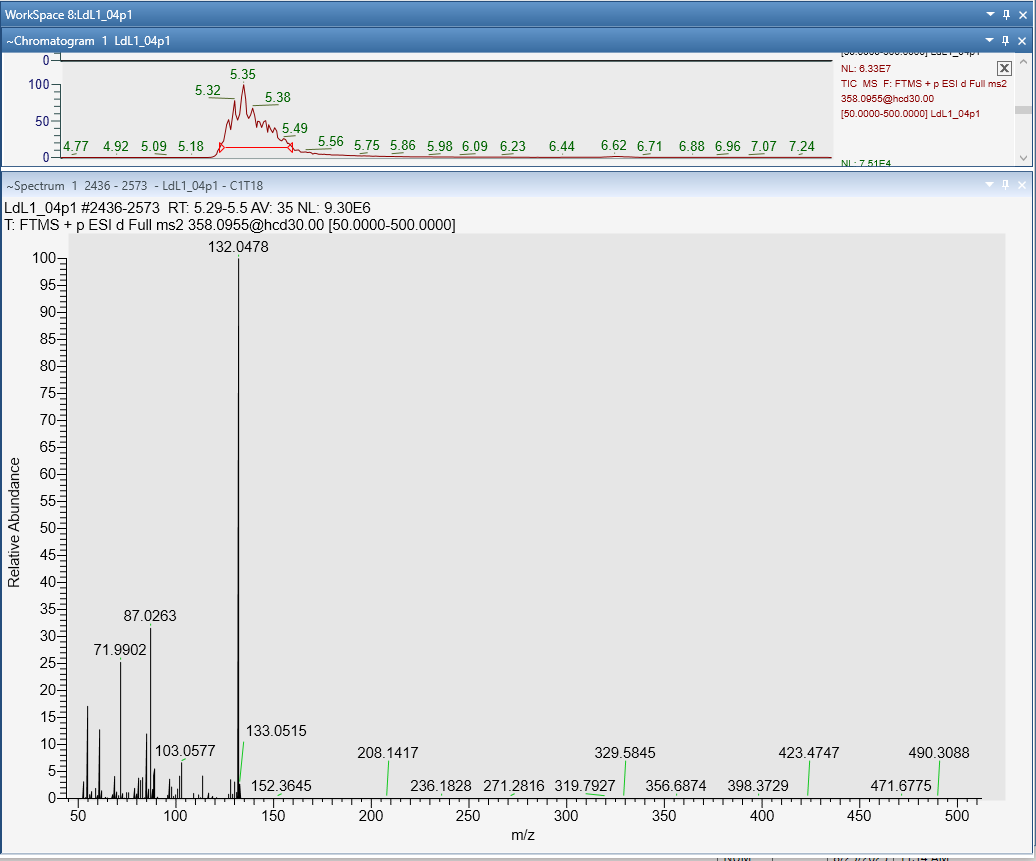


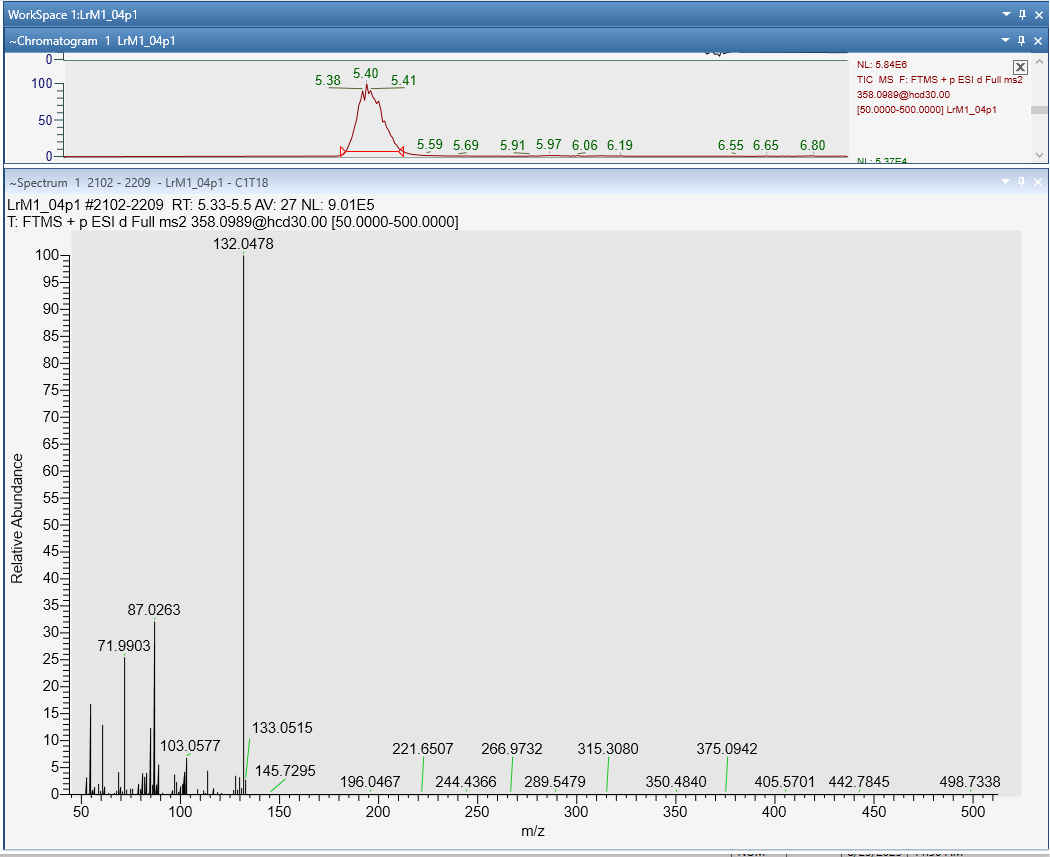


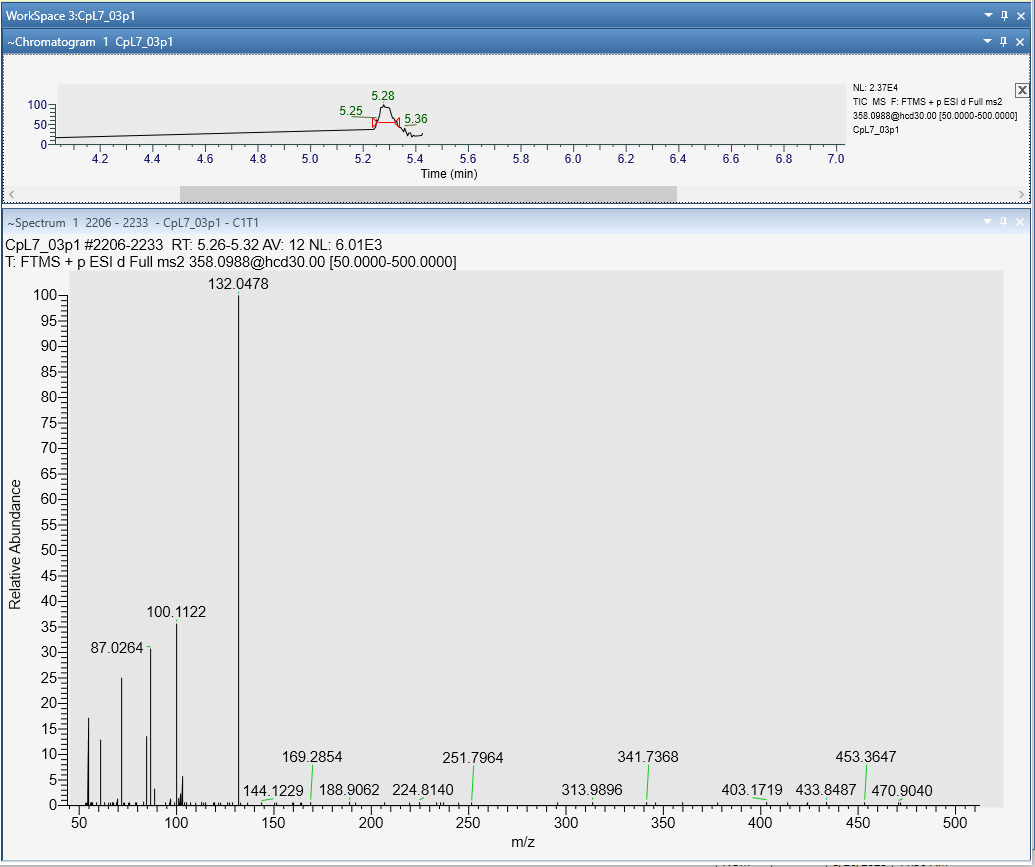


# ds 3,4-dihydroxybenzyl (#) 362.0905


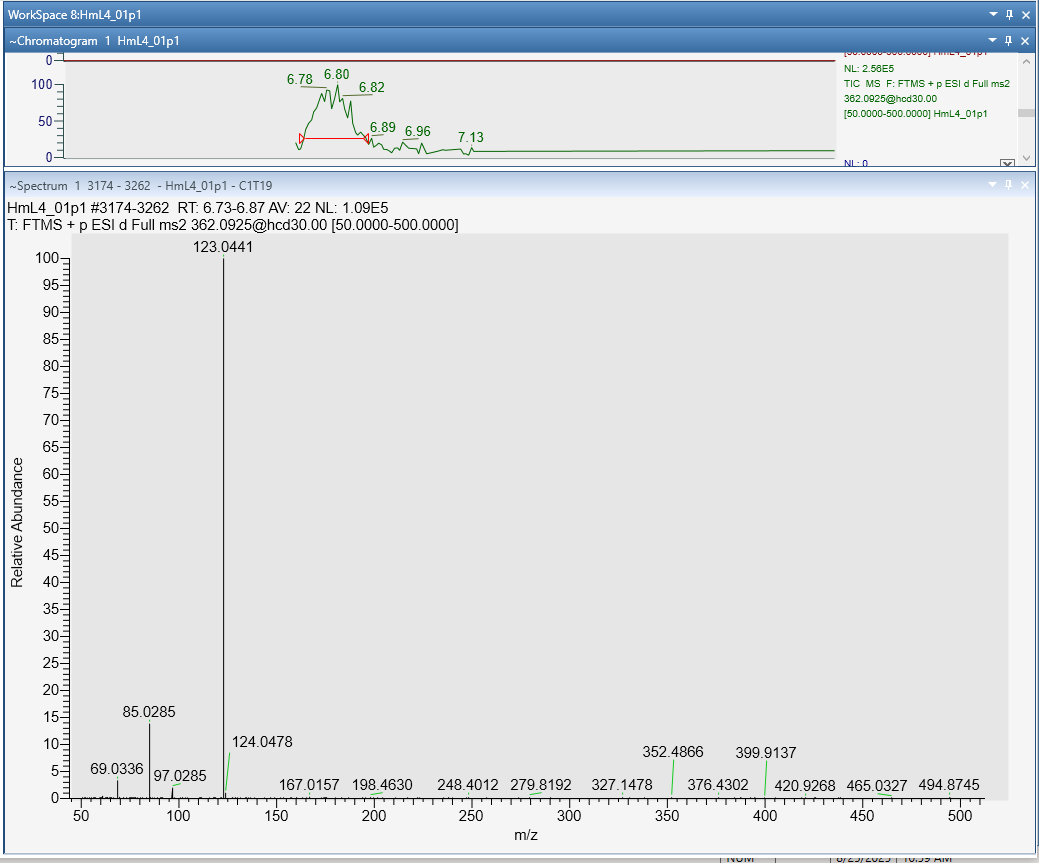


# ds indol-3-ylmethyl 369.1115


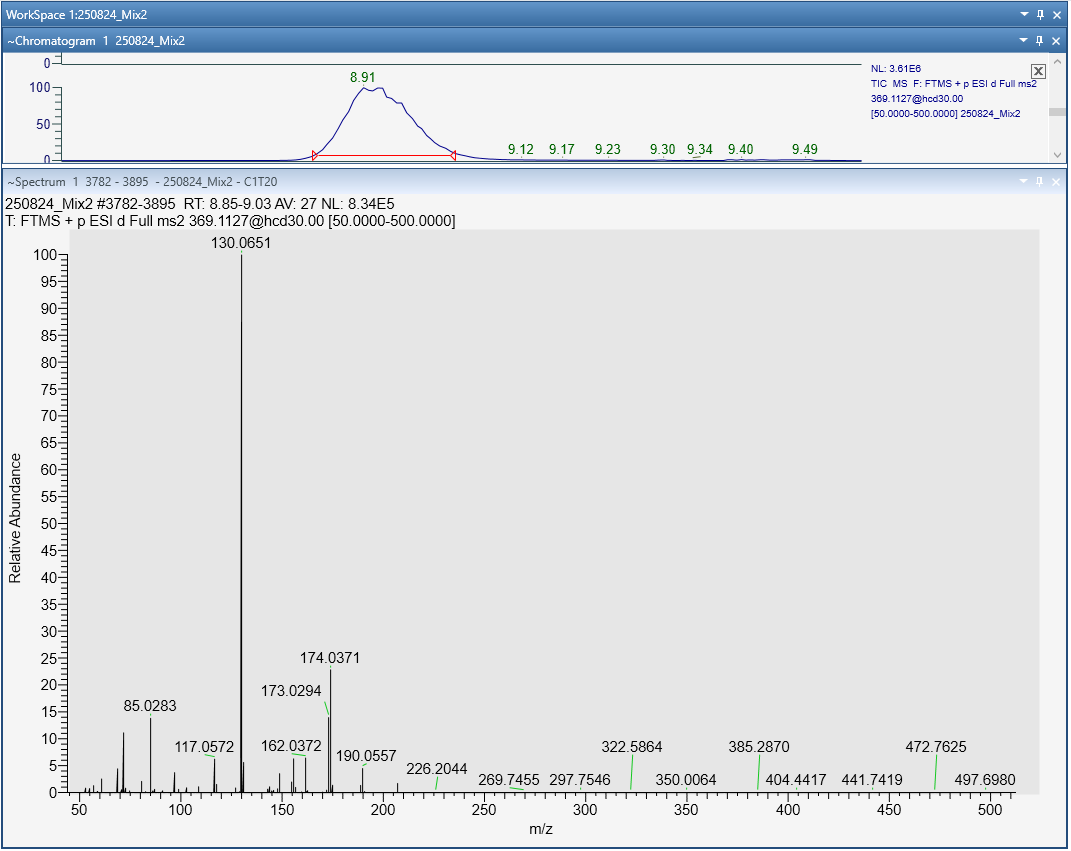


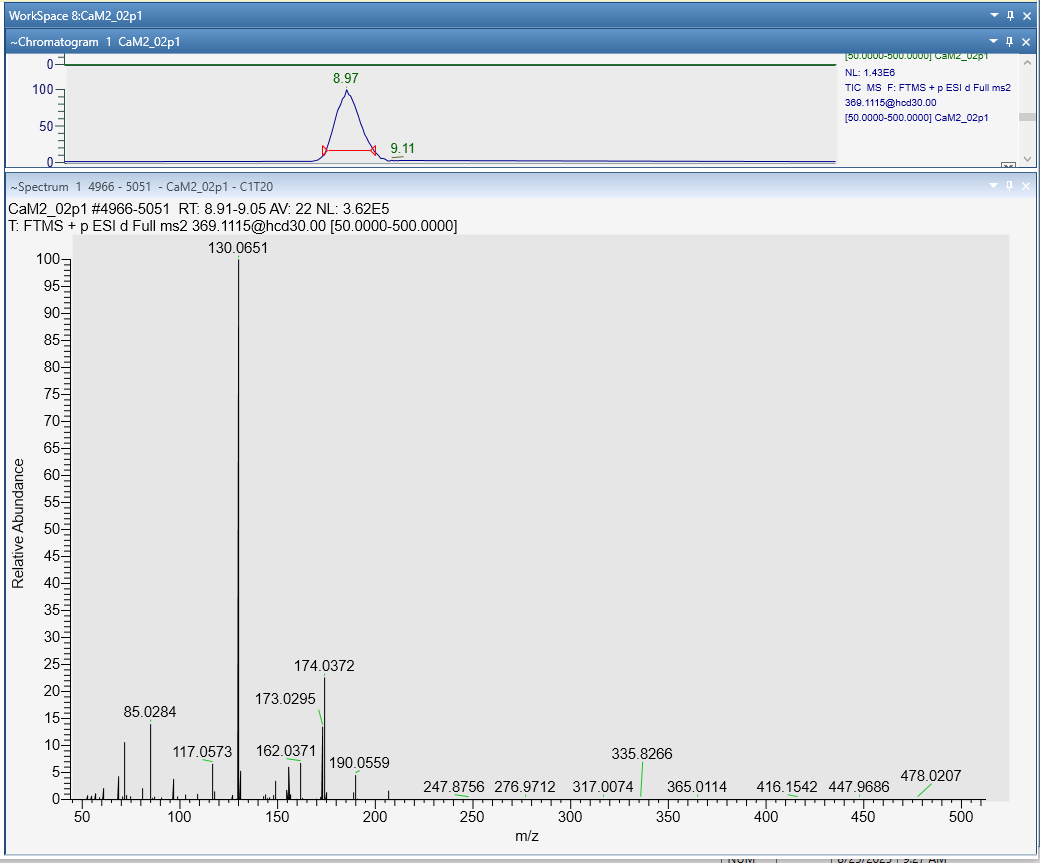


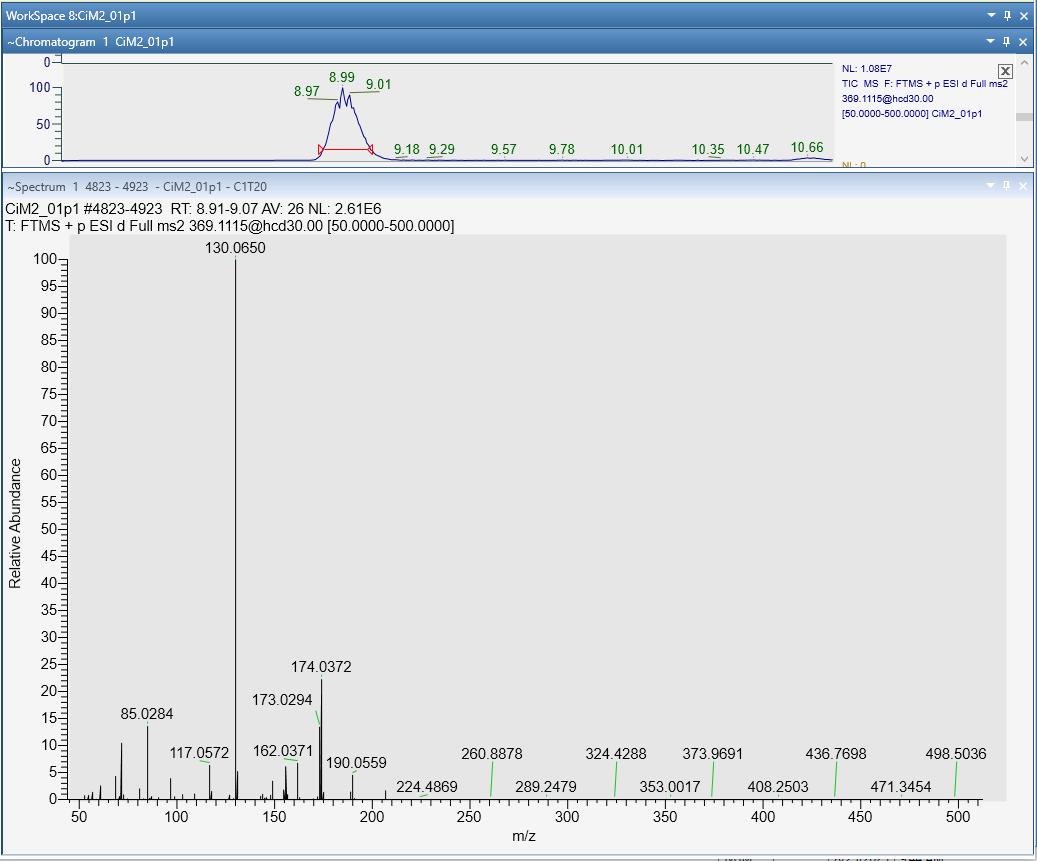


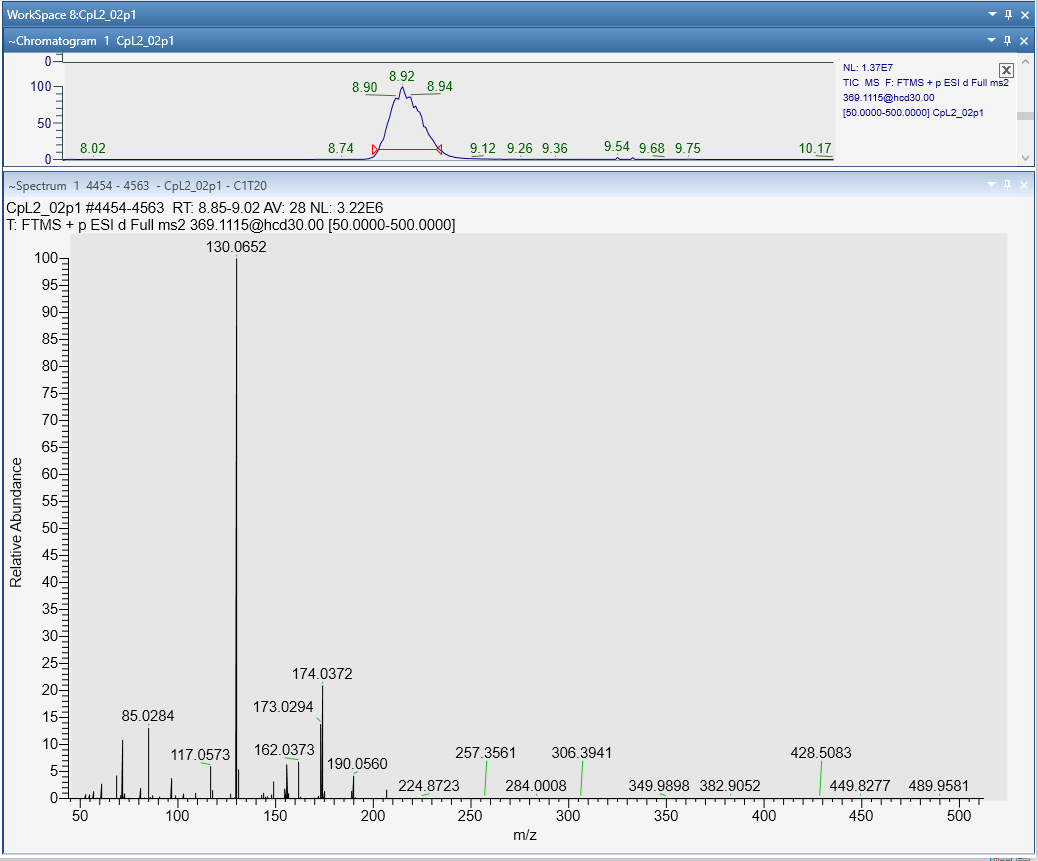


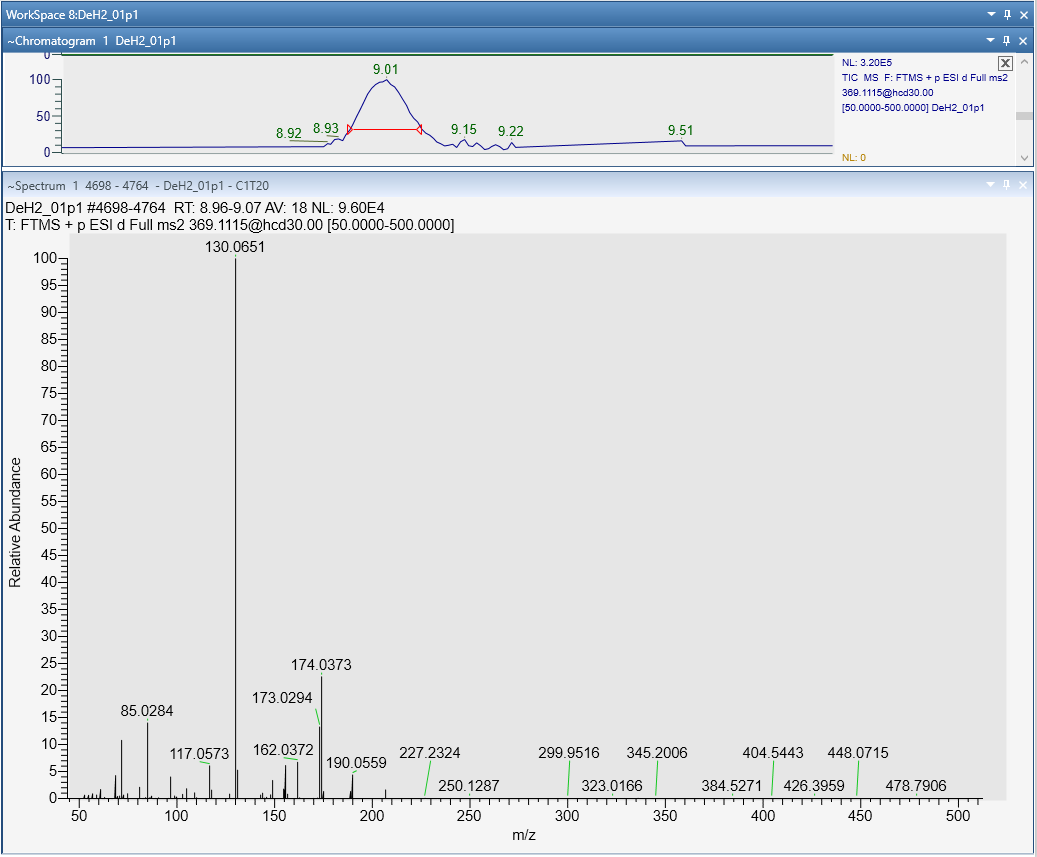


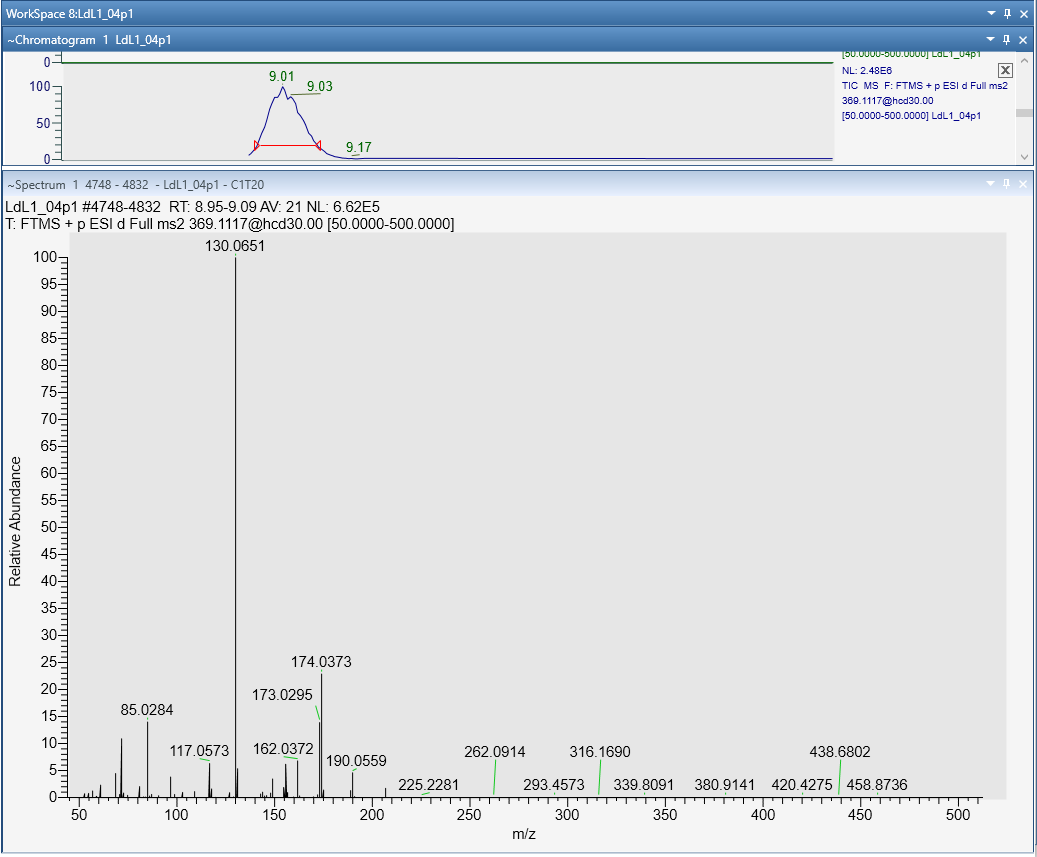


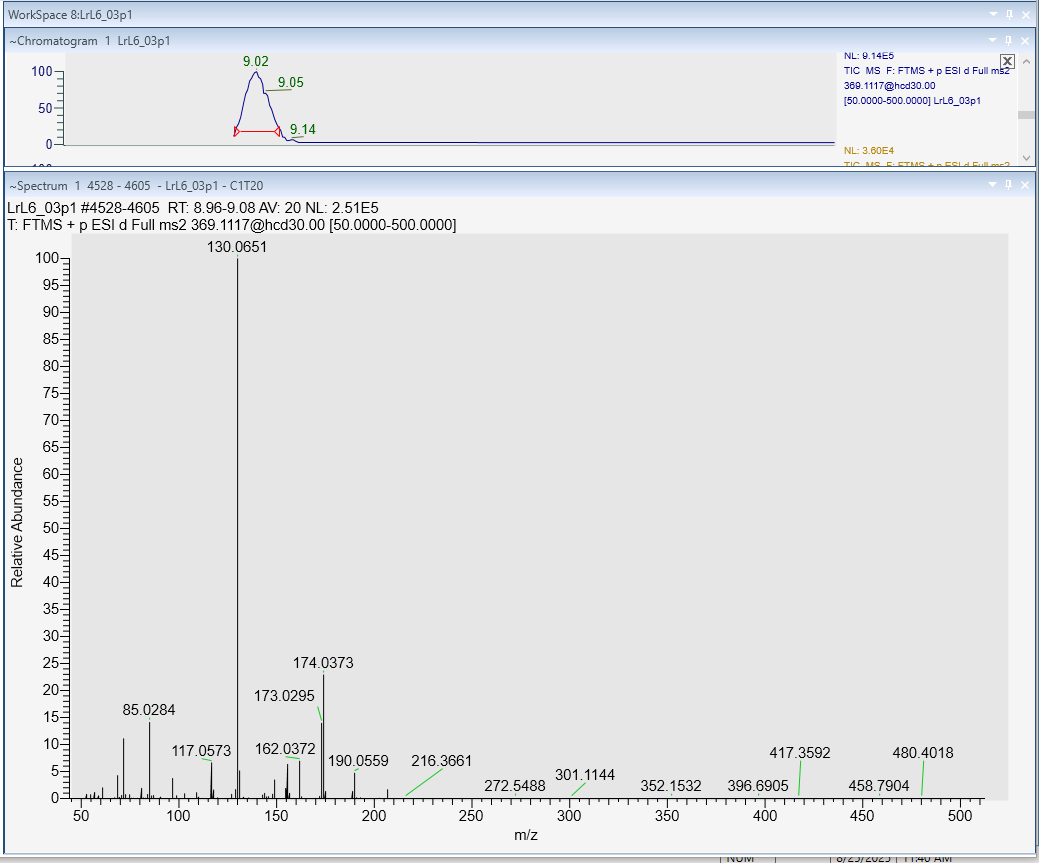


# ds 6-(methylthio)hexyl (#) 370.1353


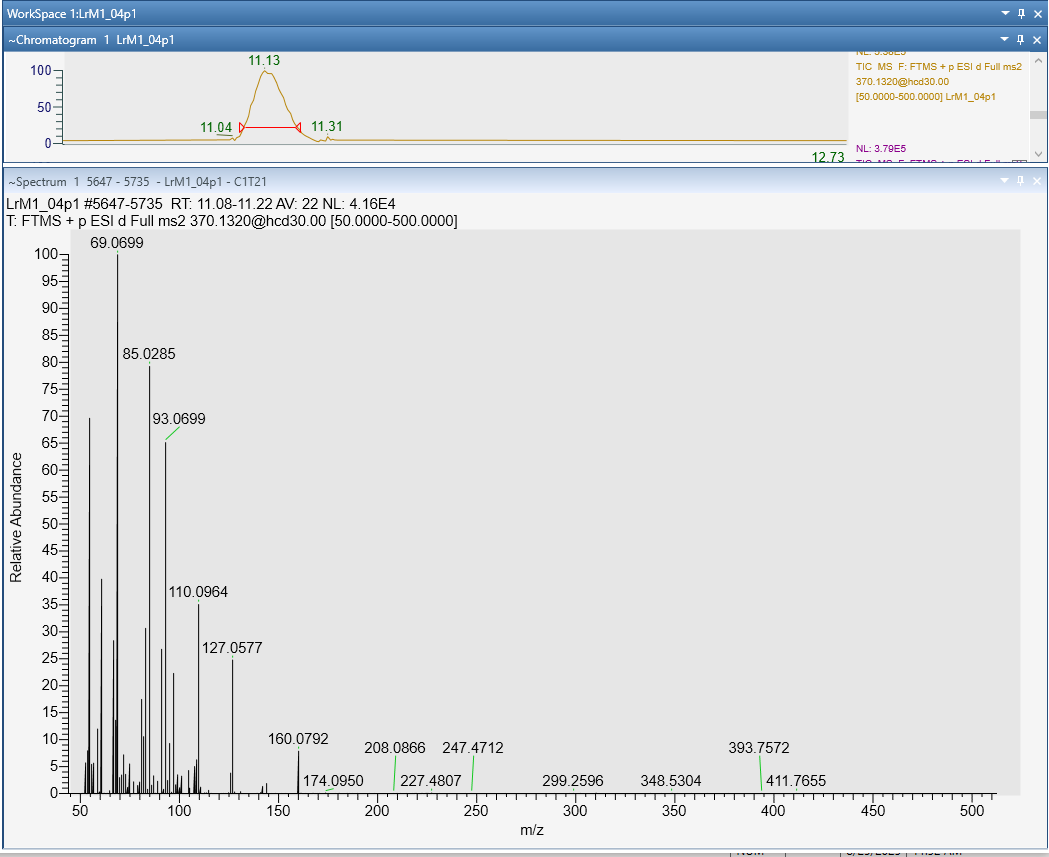


# ds 5-(methylsulfinyl)pentyl 372.1145


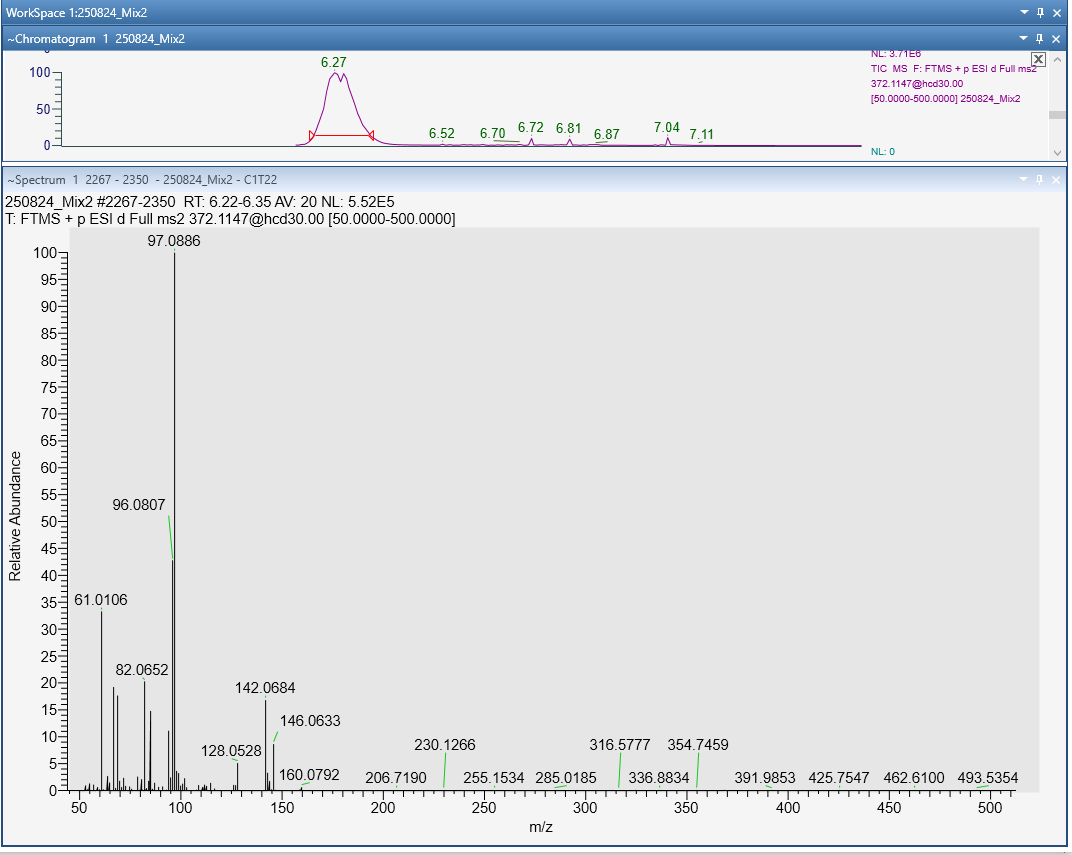


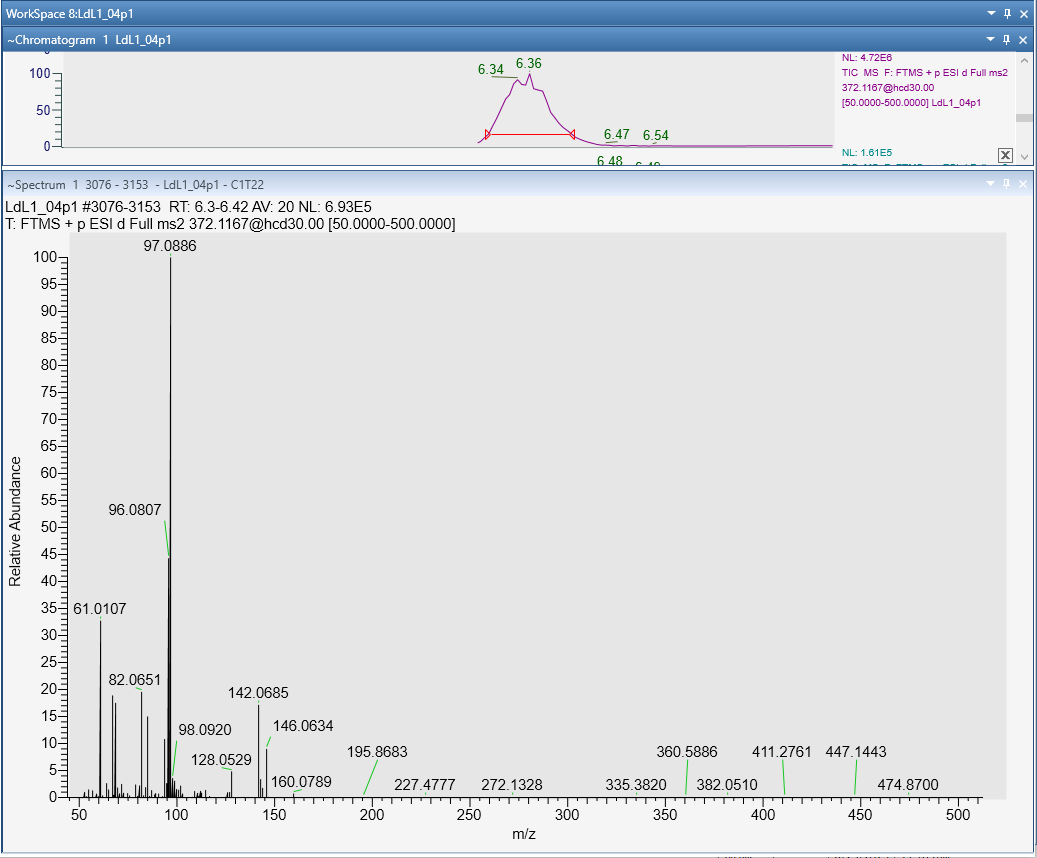


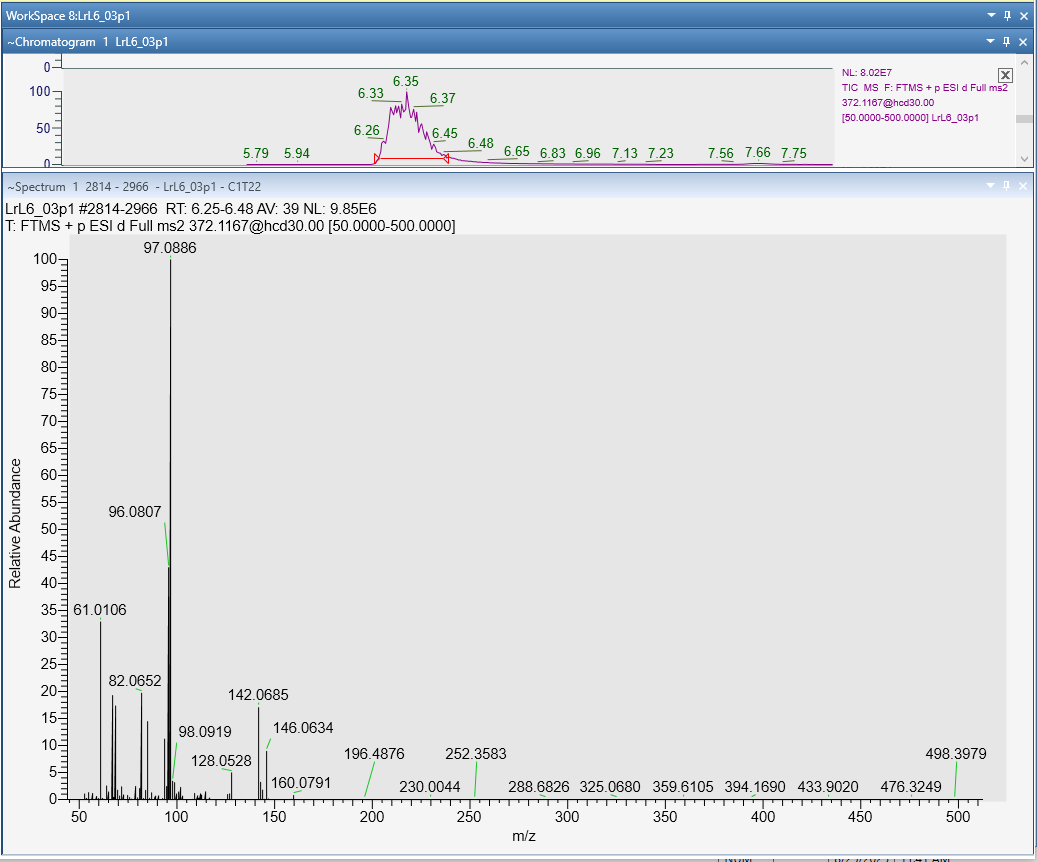


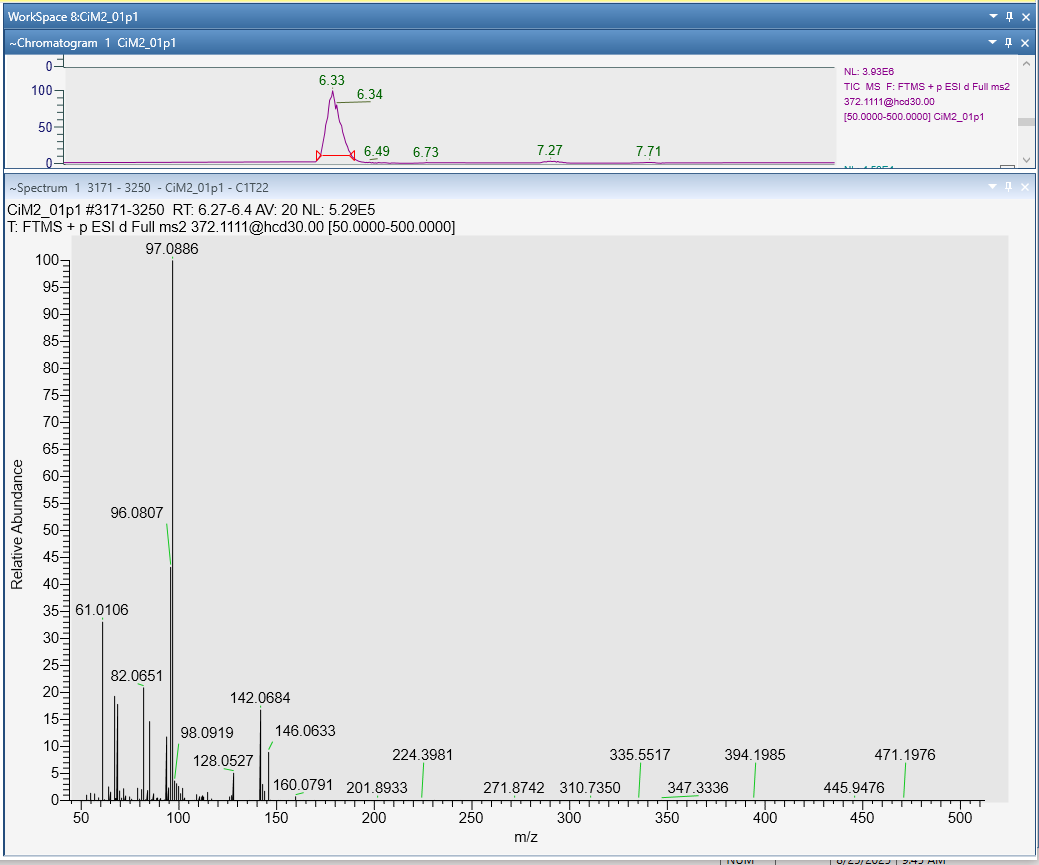


# ds 4-(methylsulfonyl)butyl (#) 374.0938


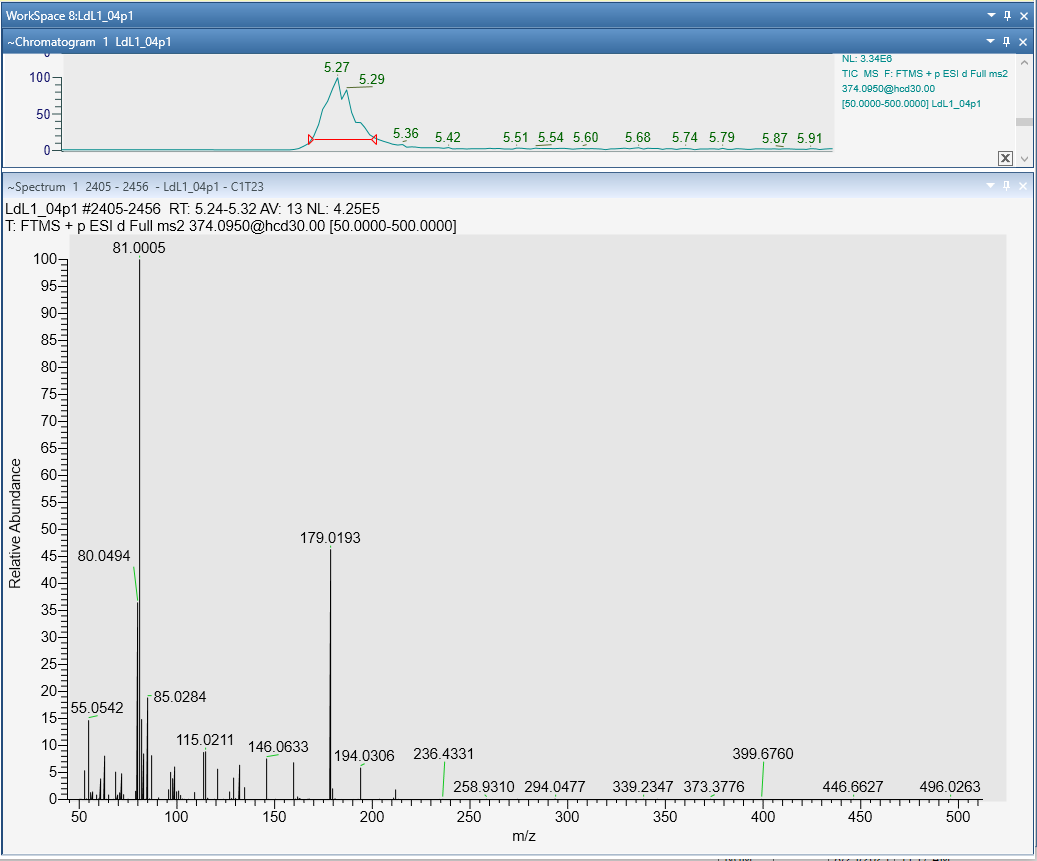


# ds 7-(methylthio)heptyl 384.151


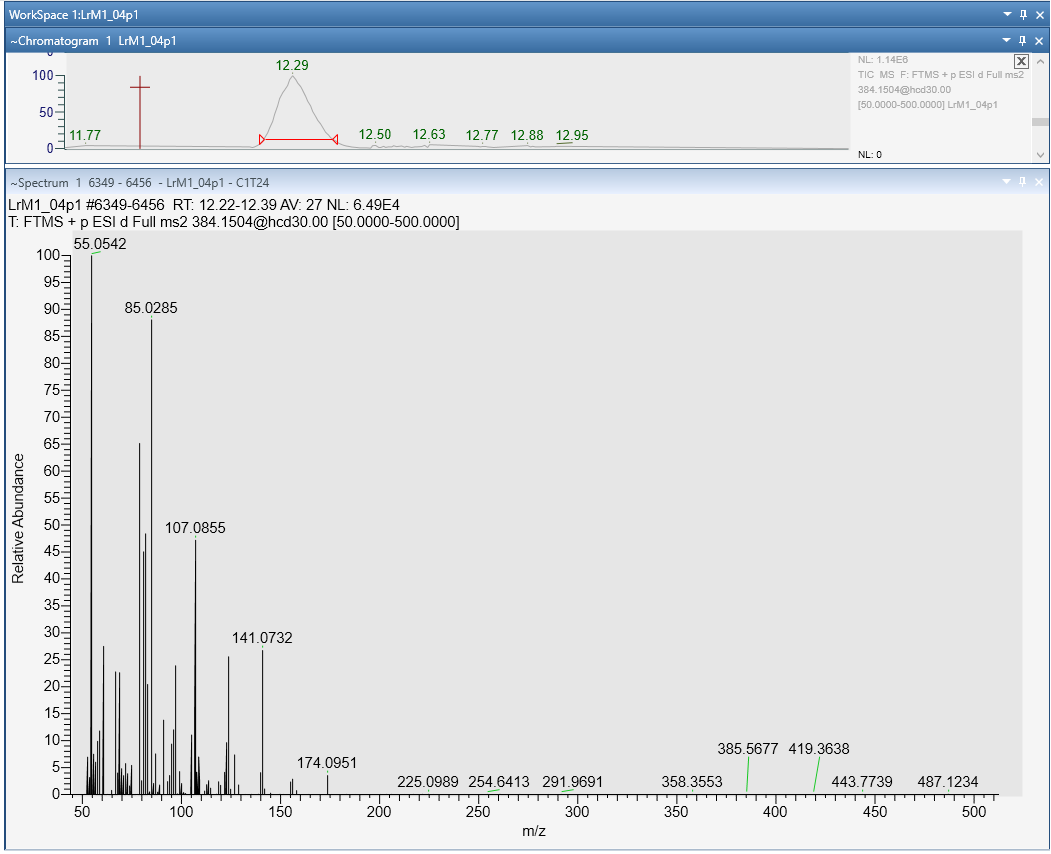


# ds 4-hydroxyindol-3-ylmethyl 385.1064


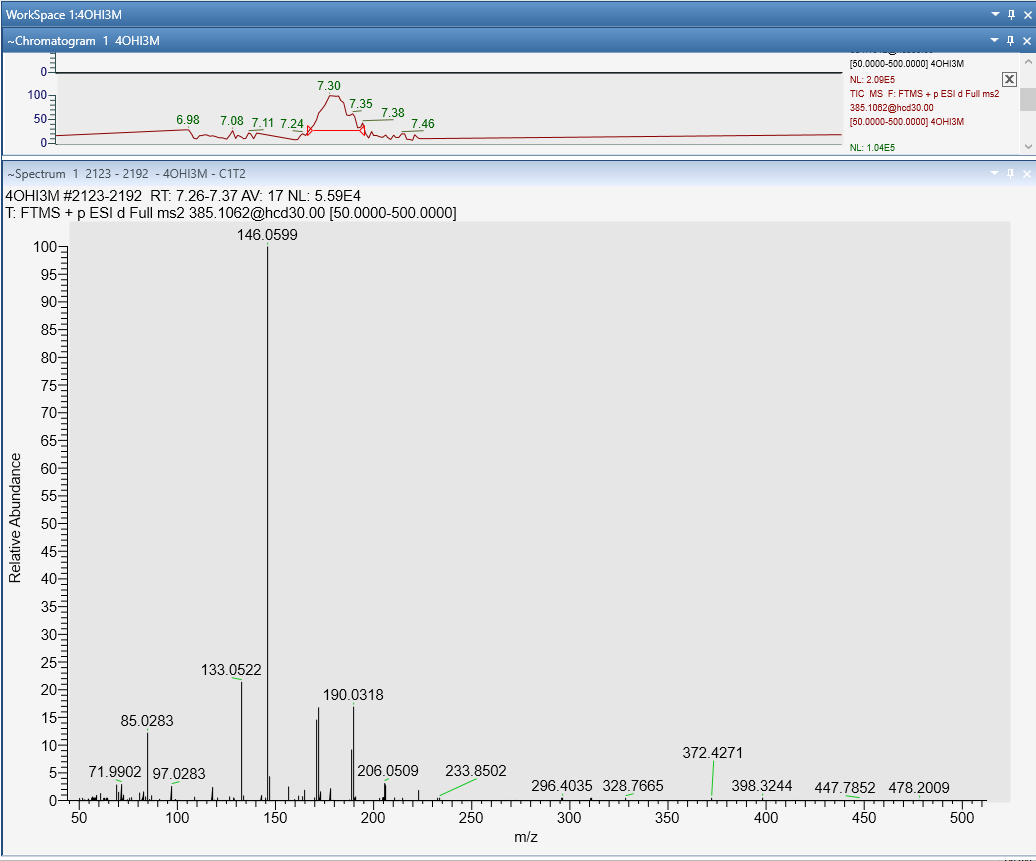


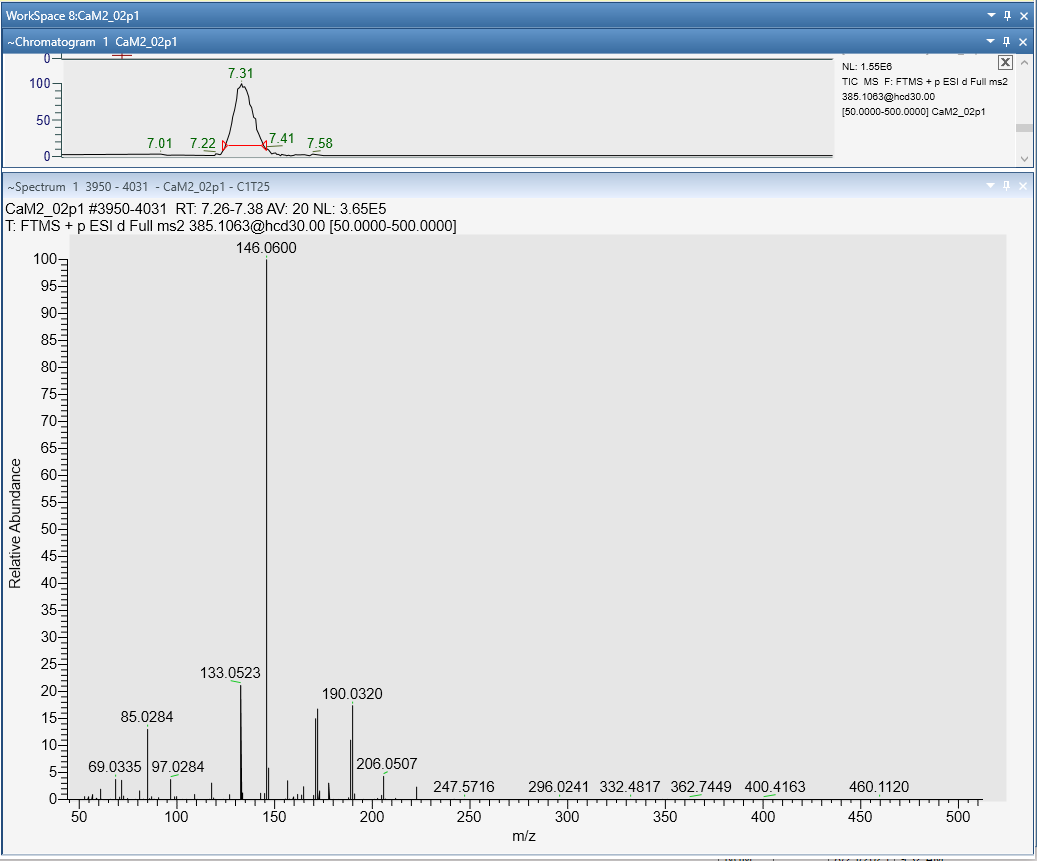


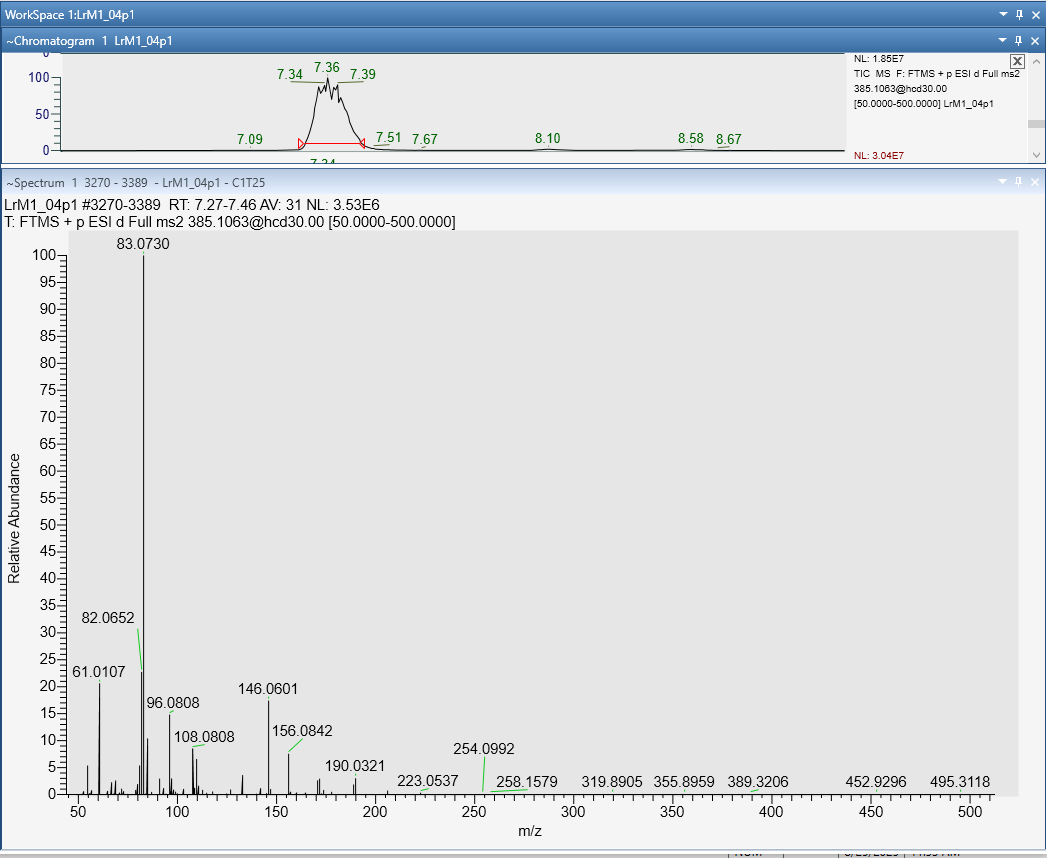


# ds 6-(methylsulfinyl)hexyl 386.1302


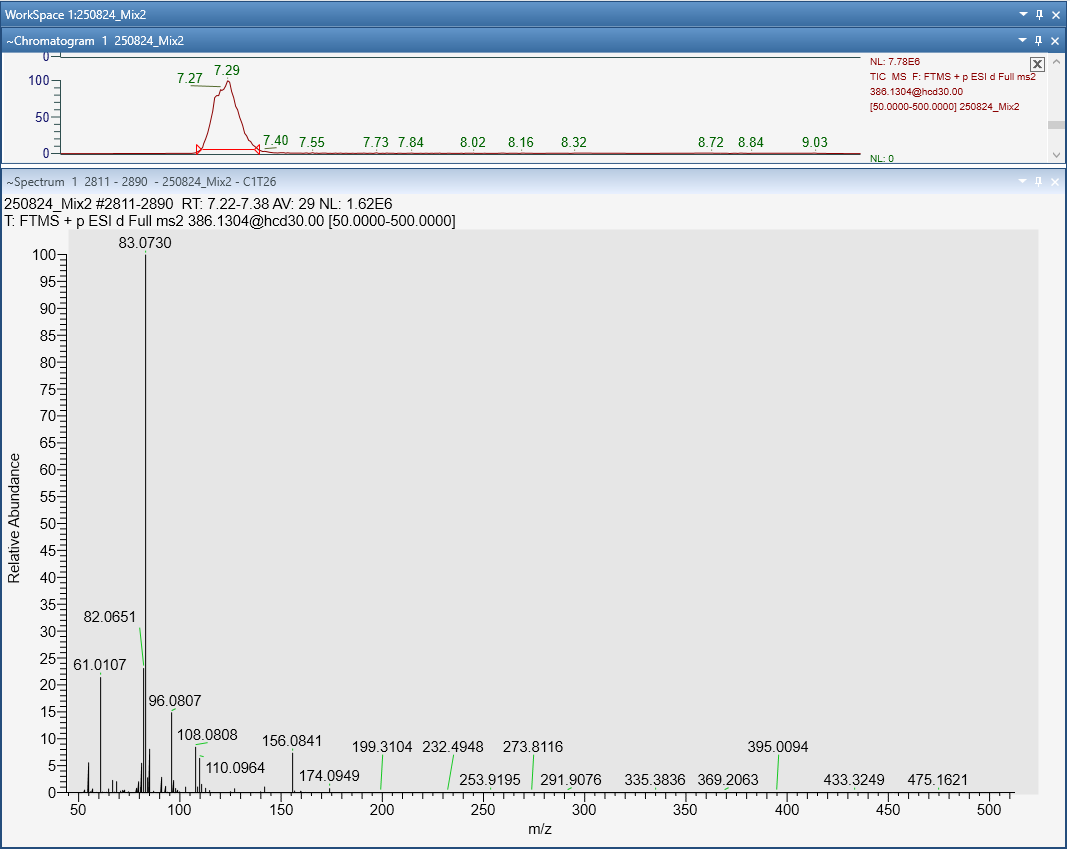


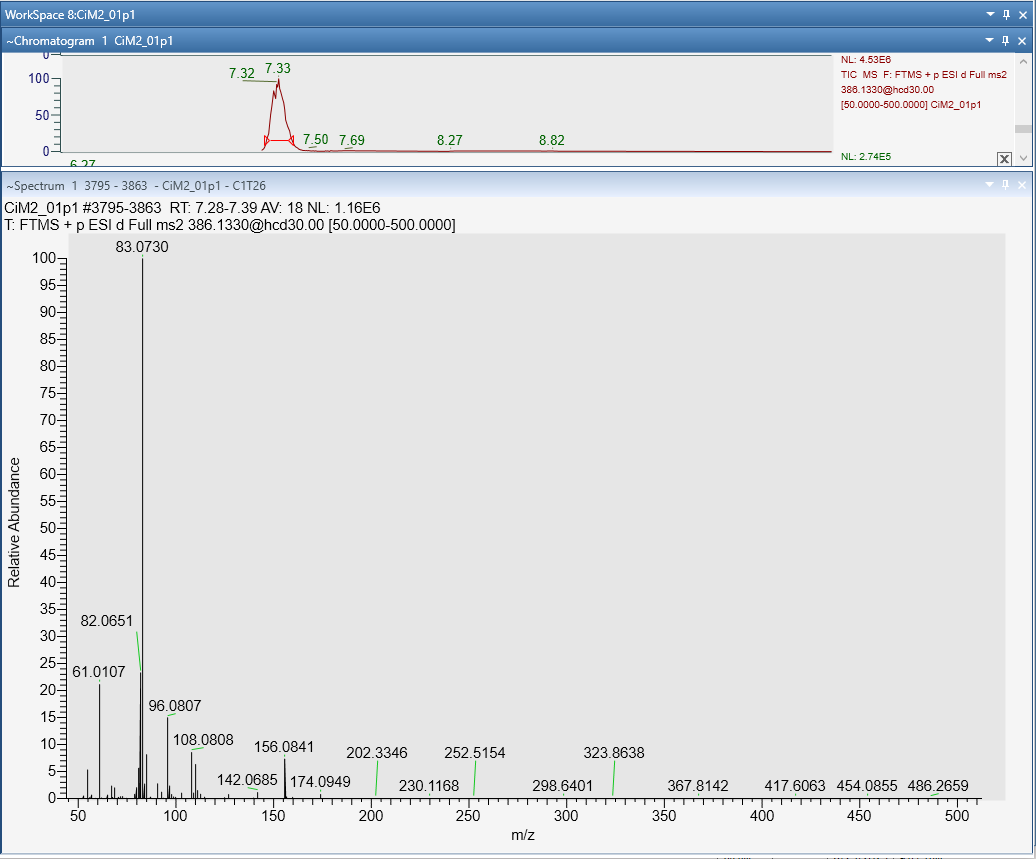


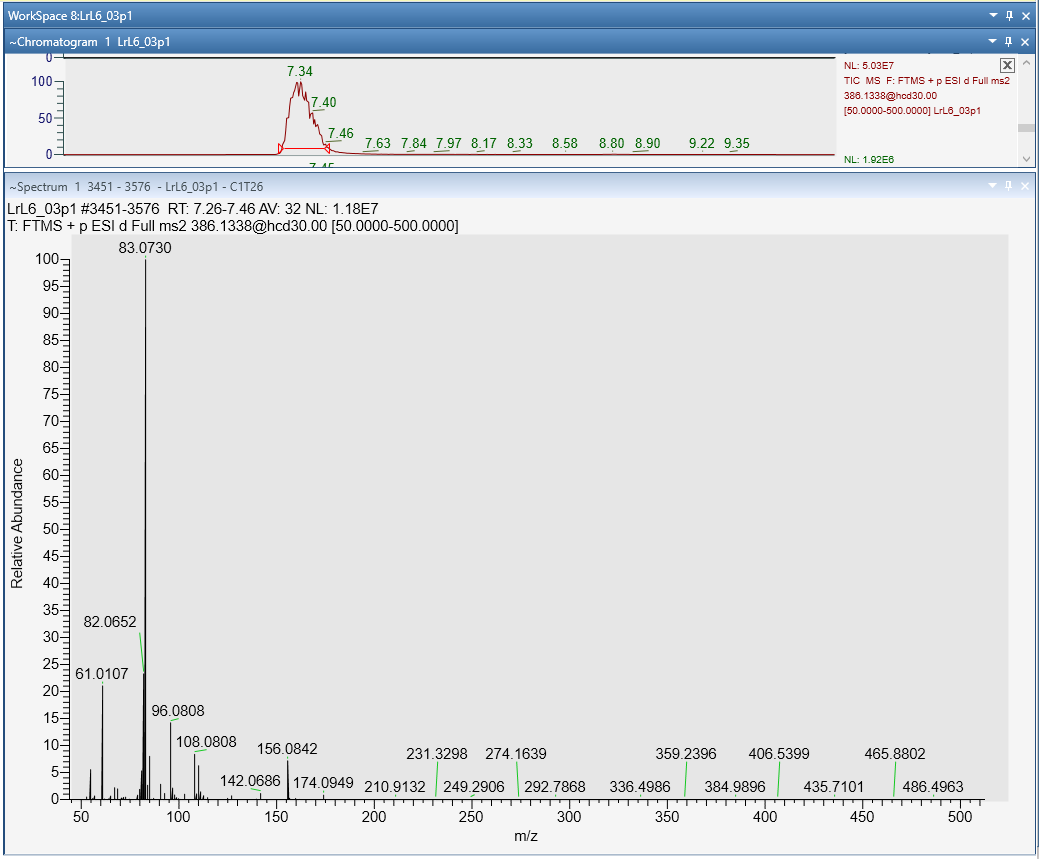


# ds 5-(methylsulfonyl)pentyl (#) 388.1095


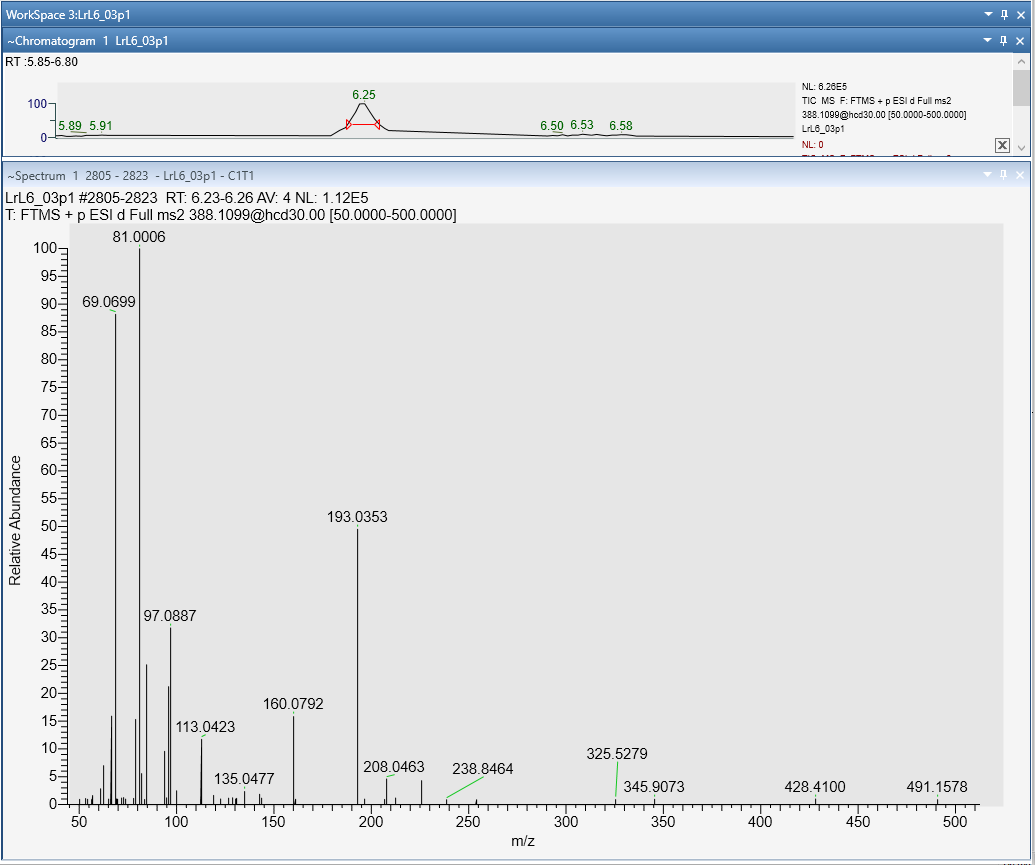


# ds 8-(methylthio)octyl (#) 398.1666


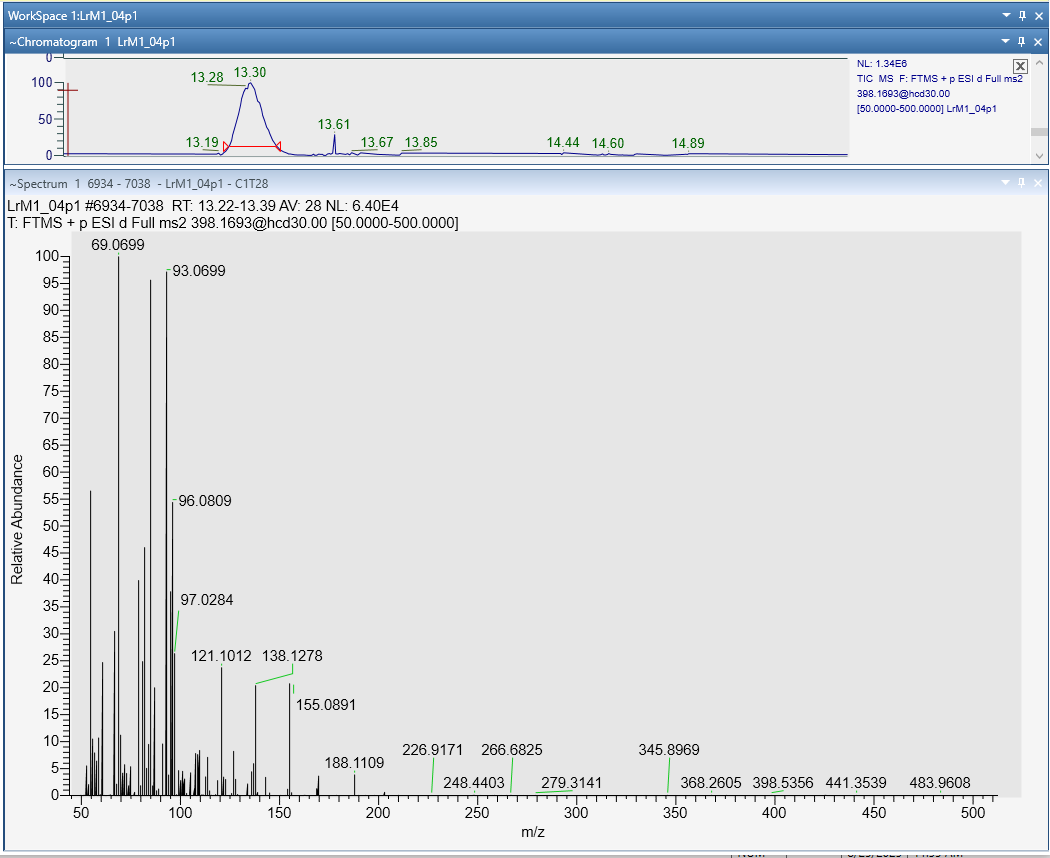


# ds 4-methoxyindol-3-ylmethyl 399.1221


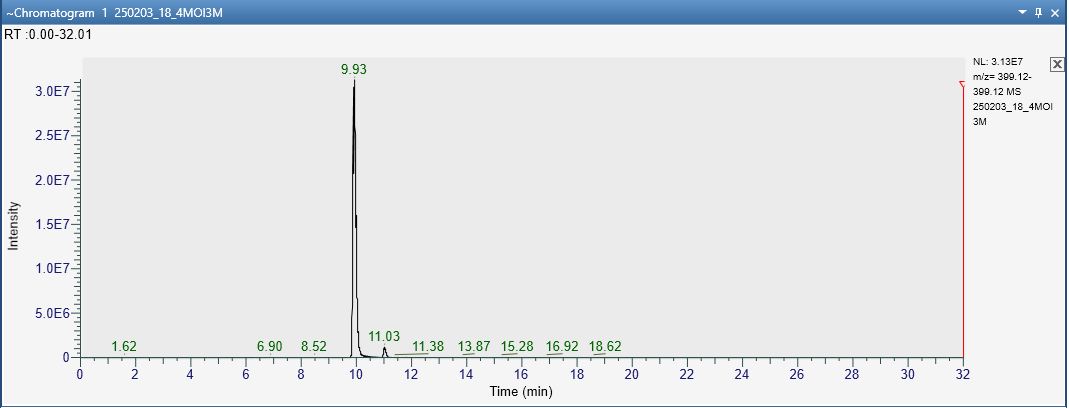


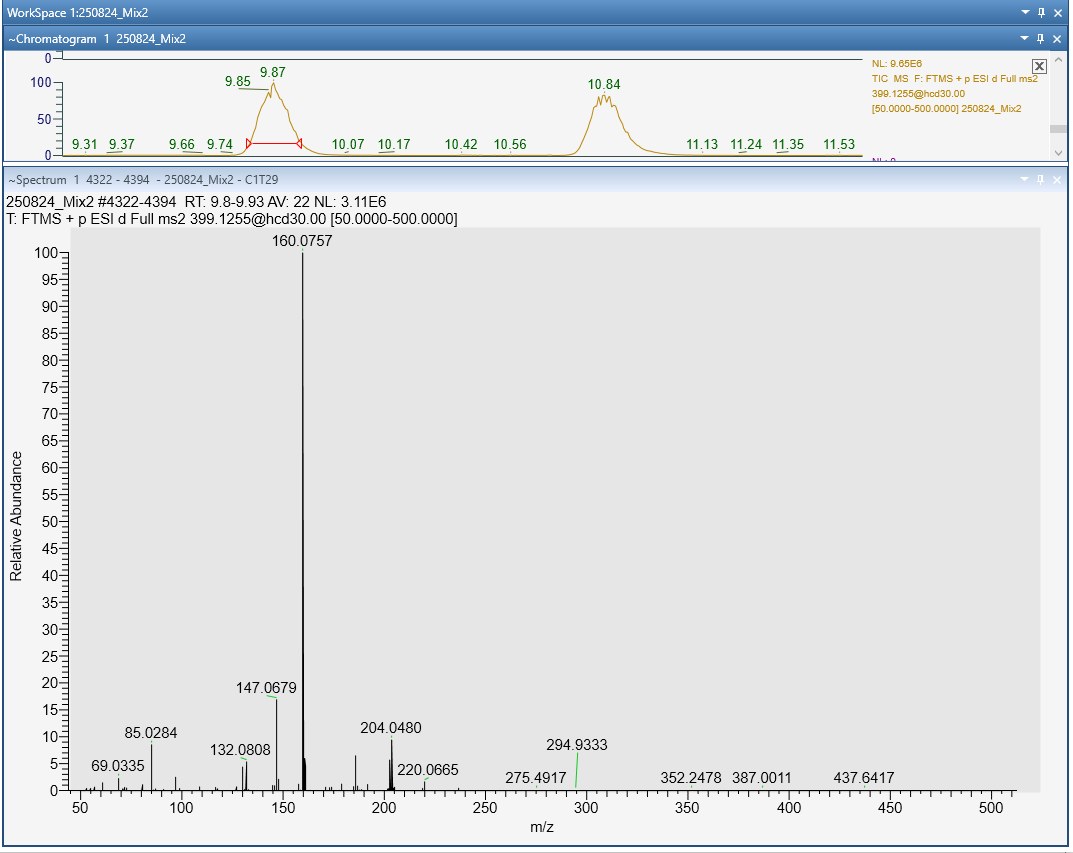


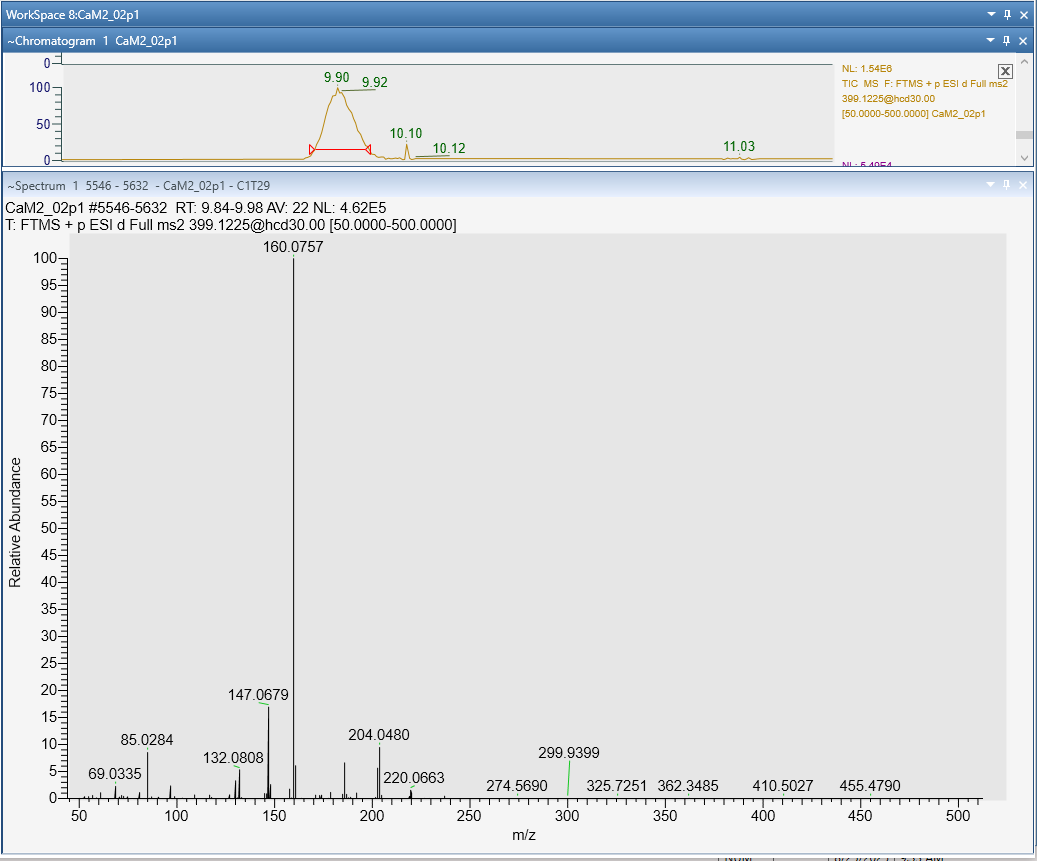


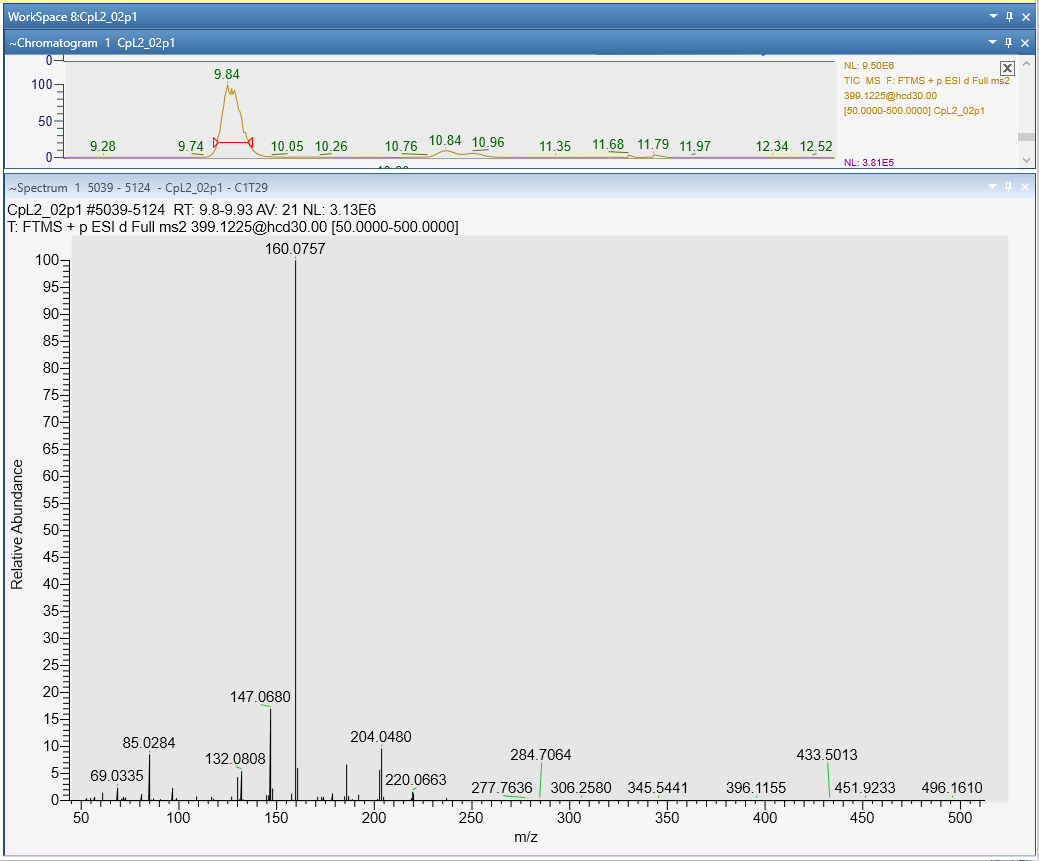


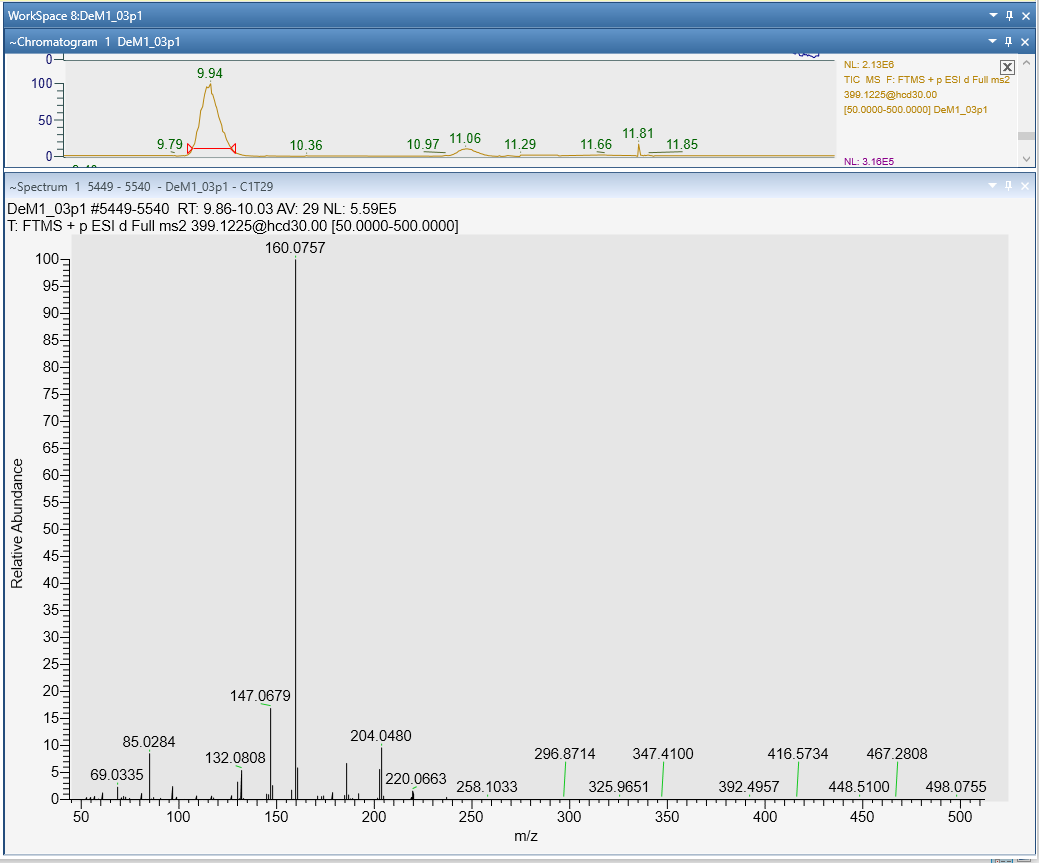


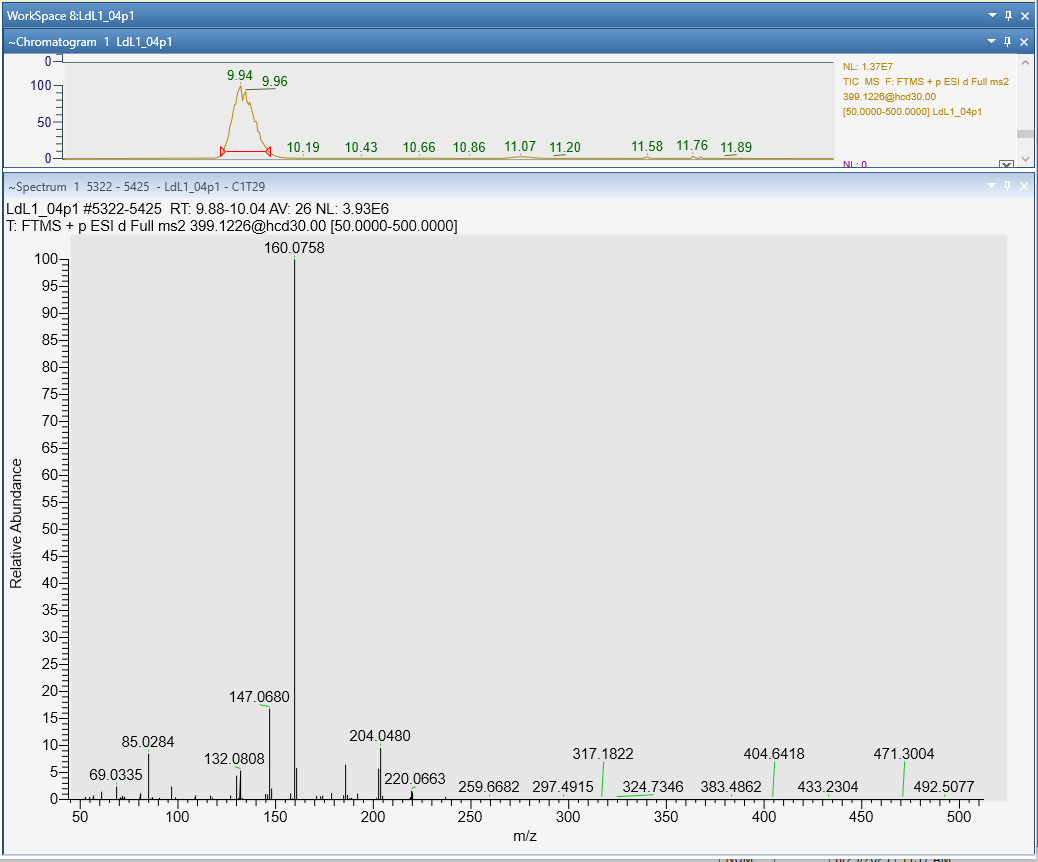


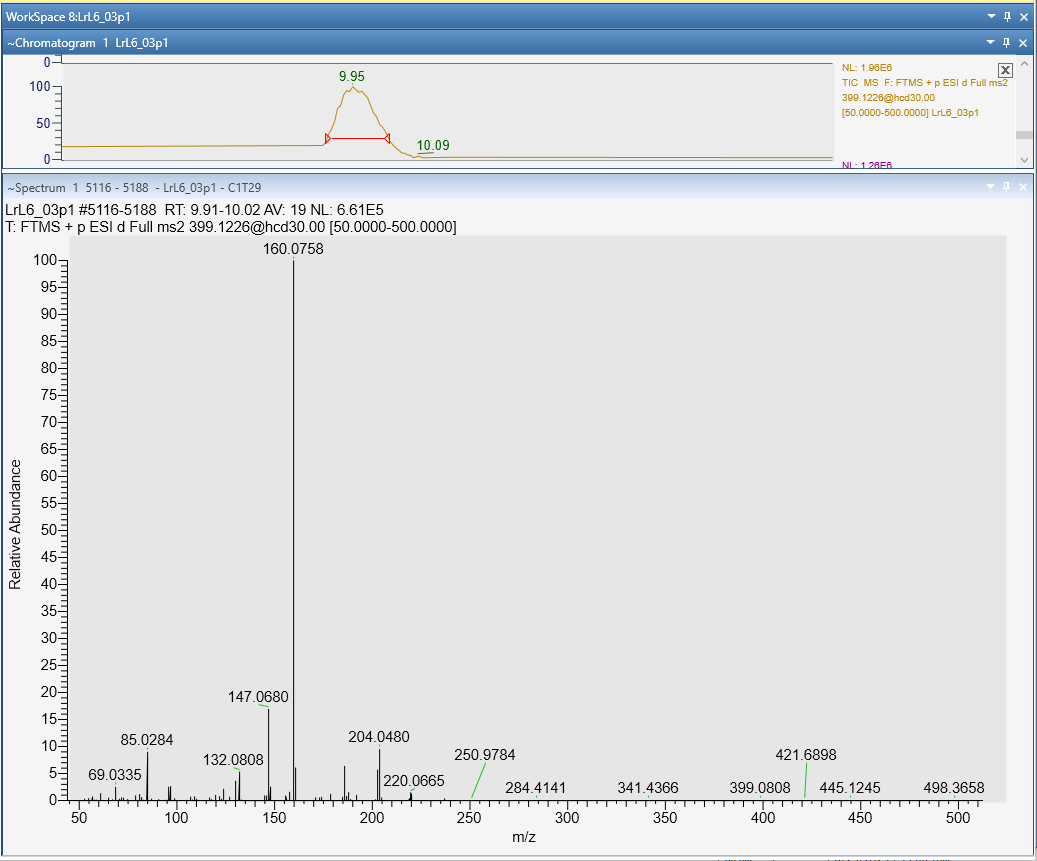


# ds 1-methoxyindol-3-ylmethyl 399.1221


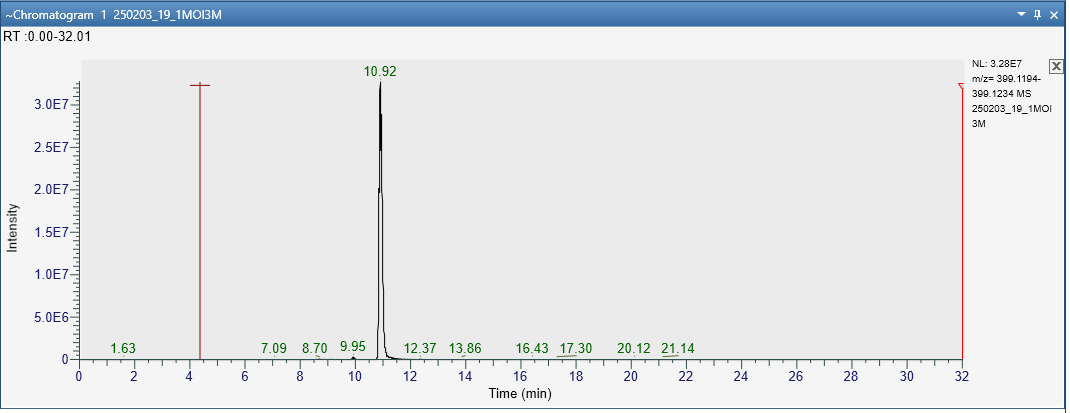


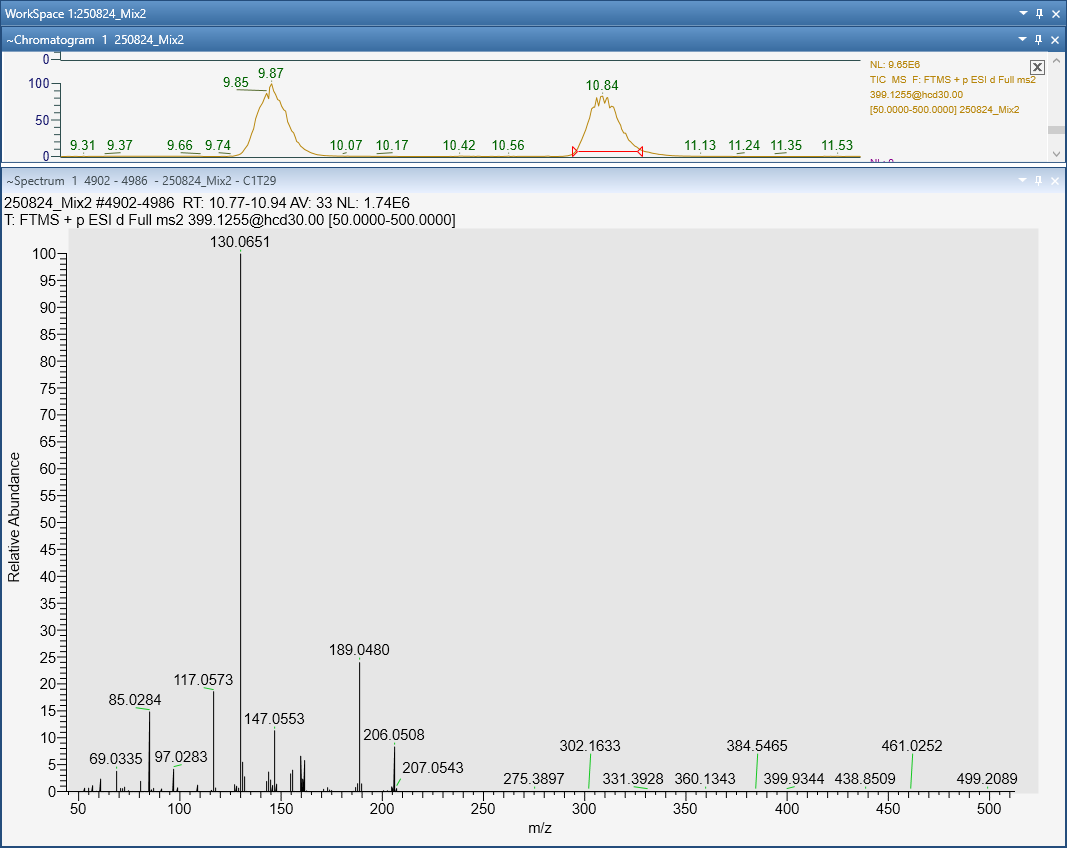


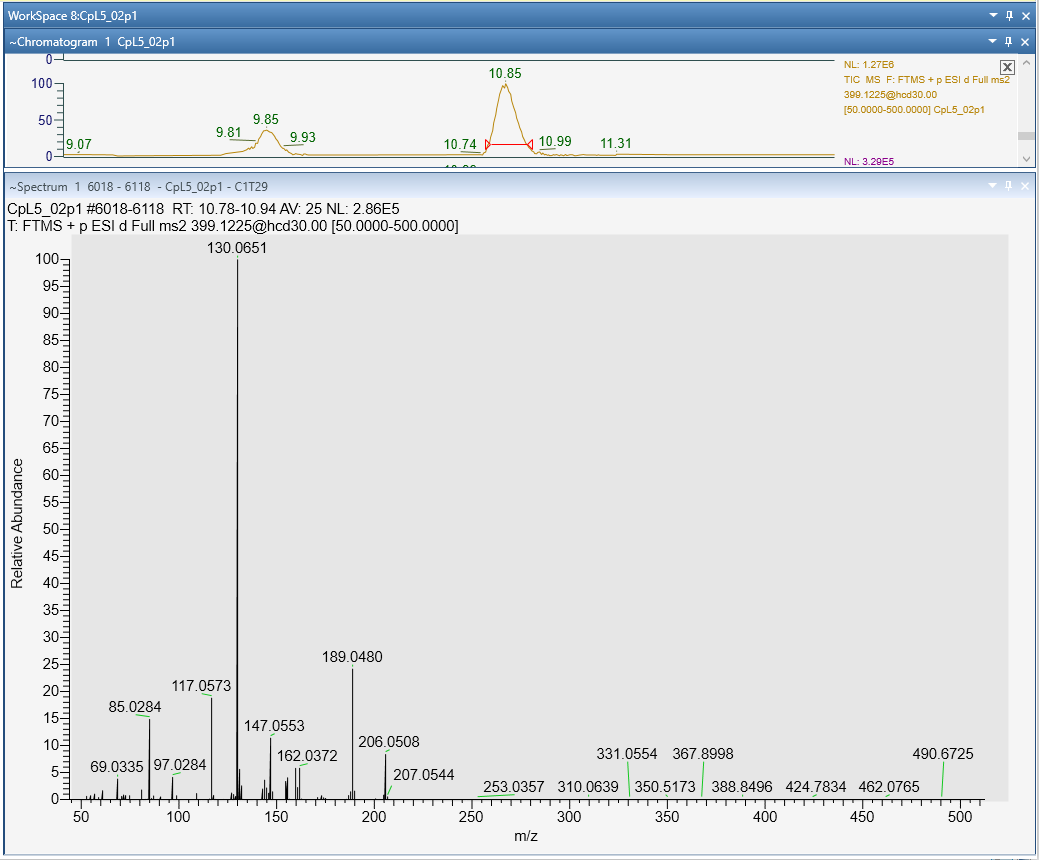


# ds 7-(methylsulfinyl)heptyl (#) 400.1459


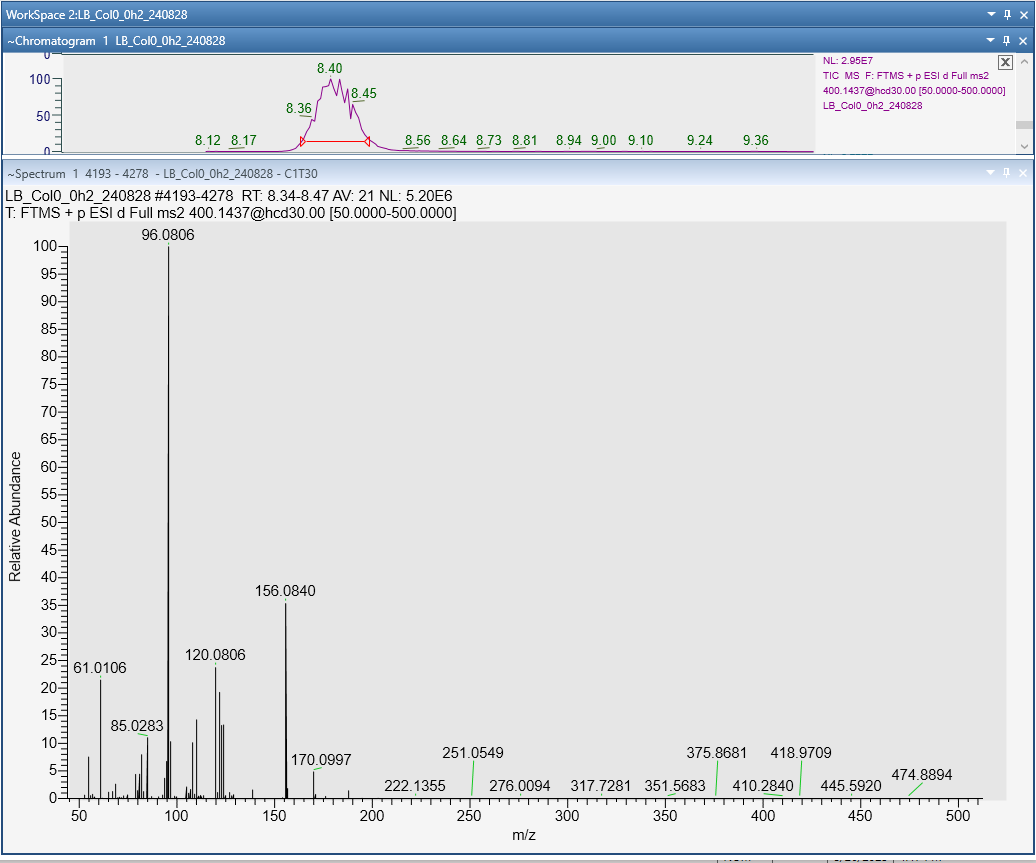


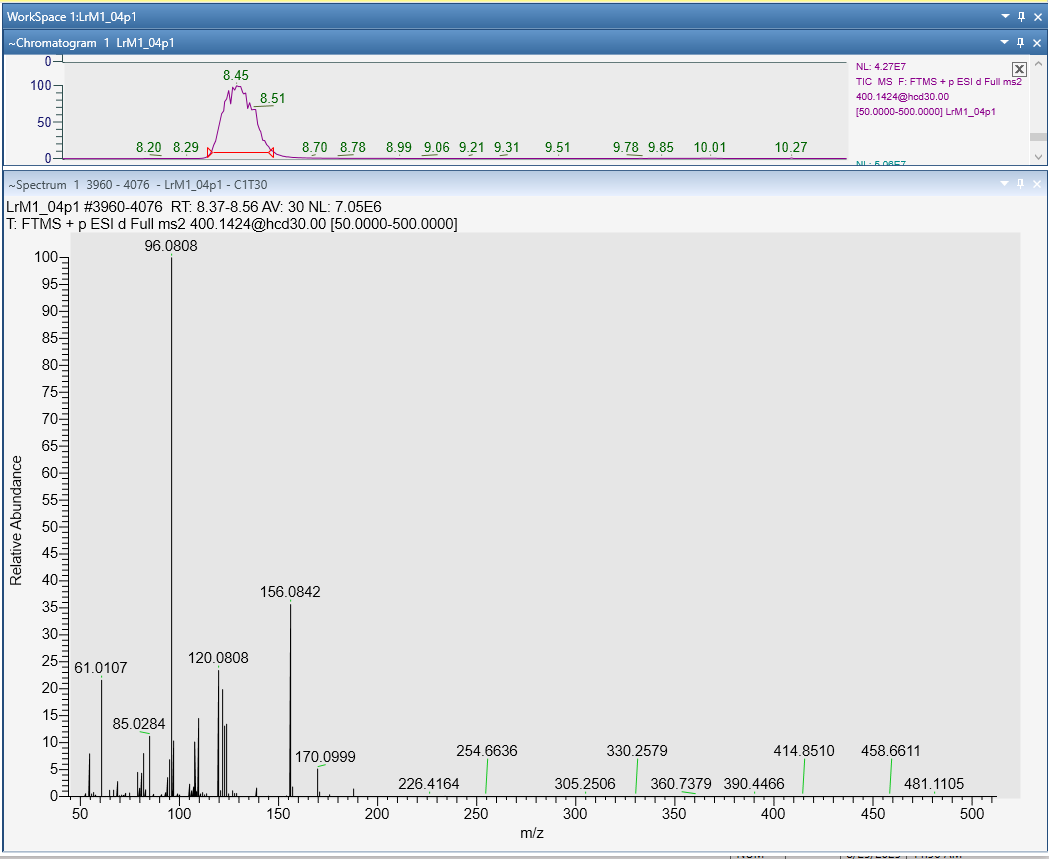


# ds 8-(methylsulfinyl)octyl 414.1615


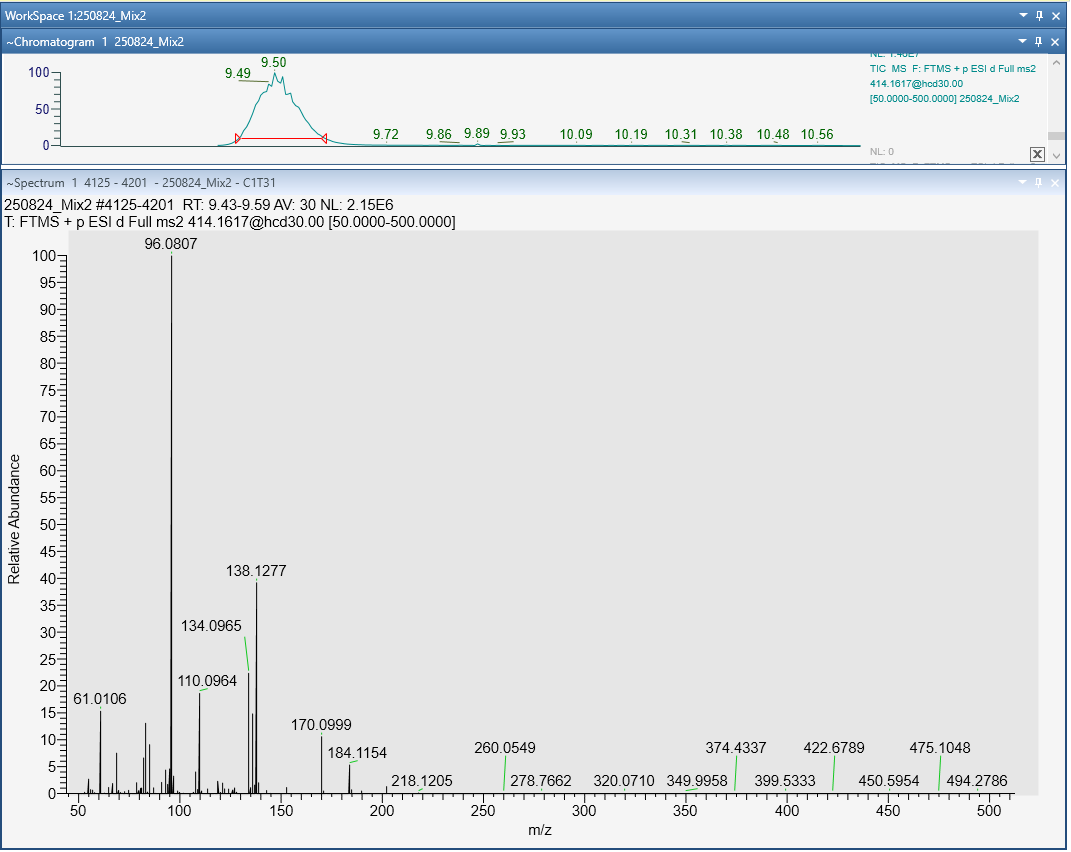


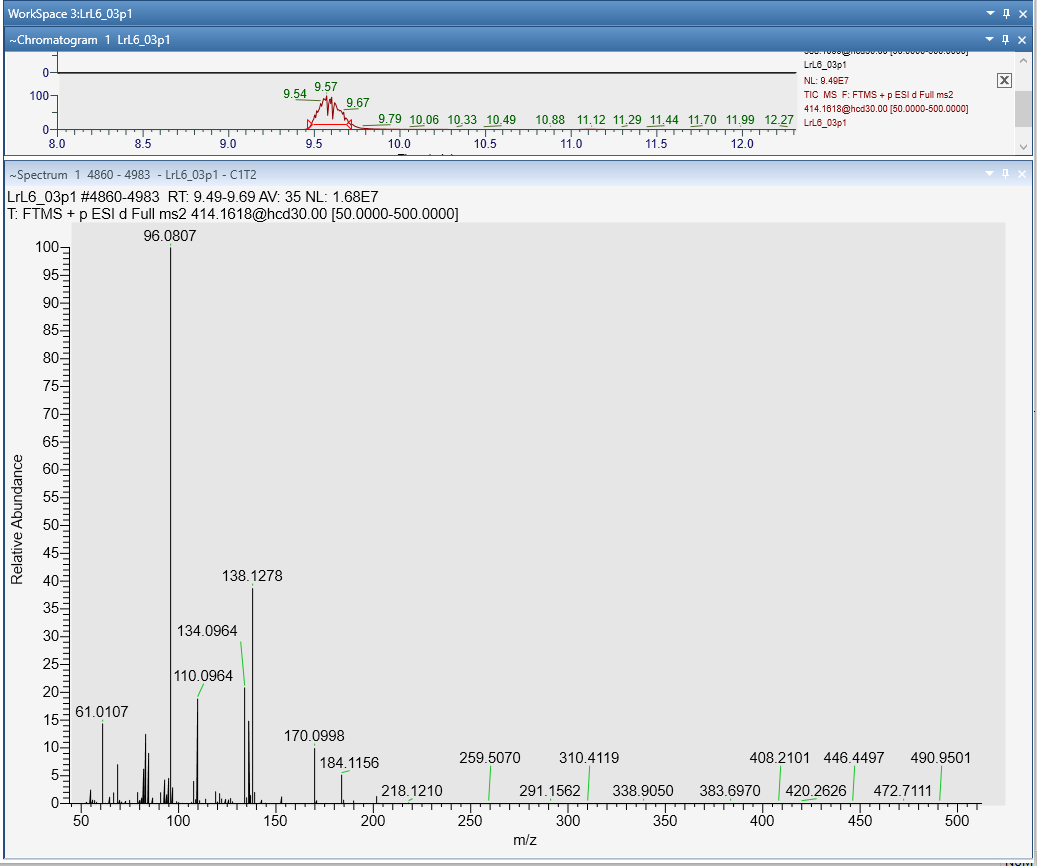


# ds 9-(methylsulfinyl)nonyl (#) 428.1772


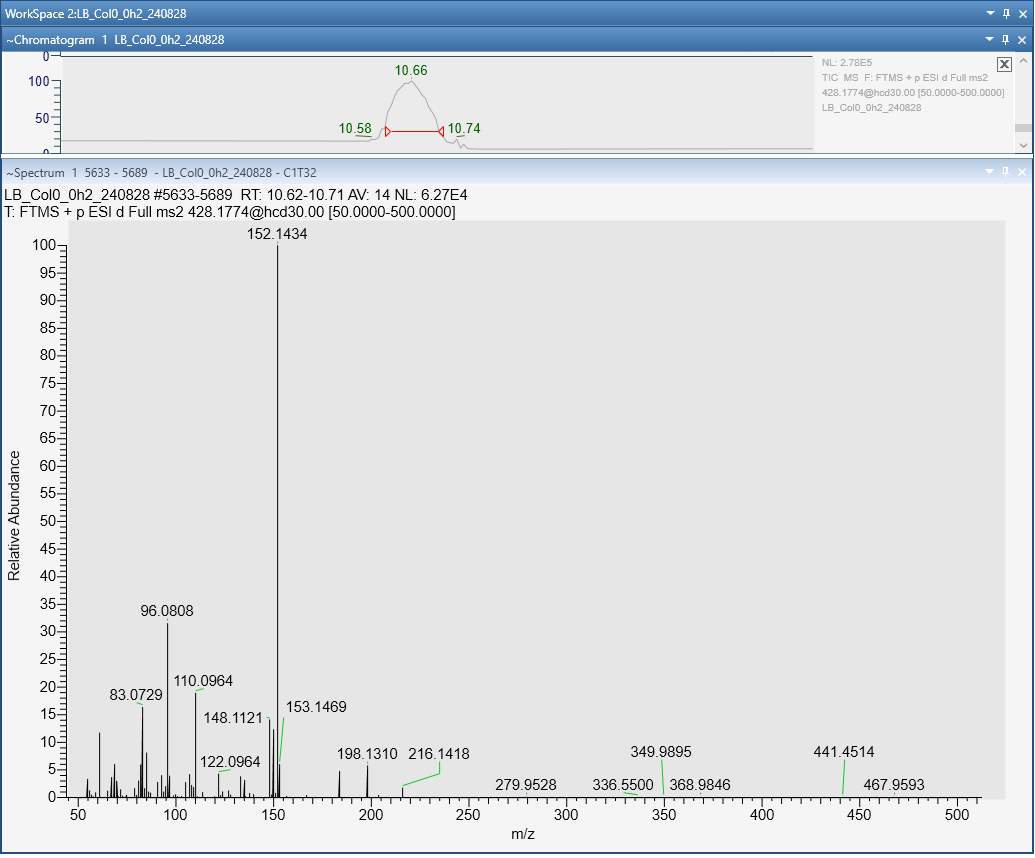


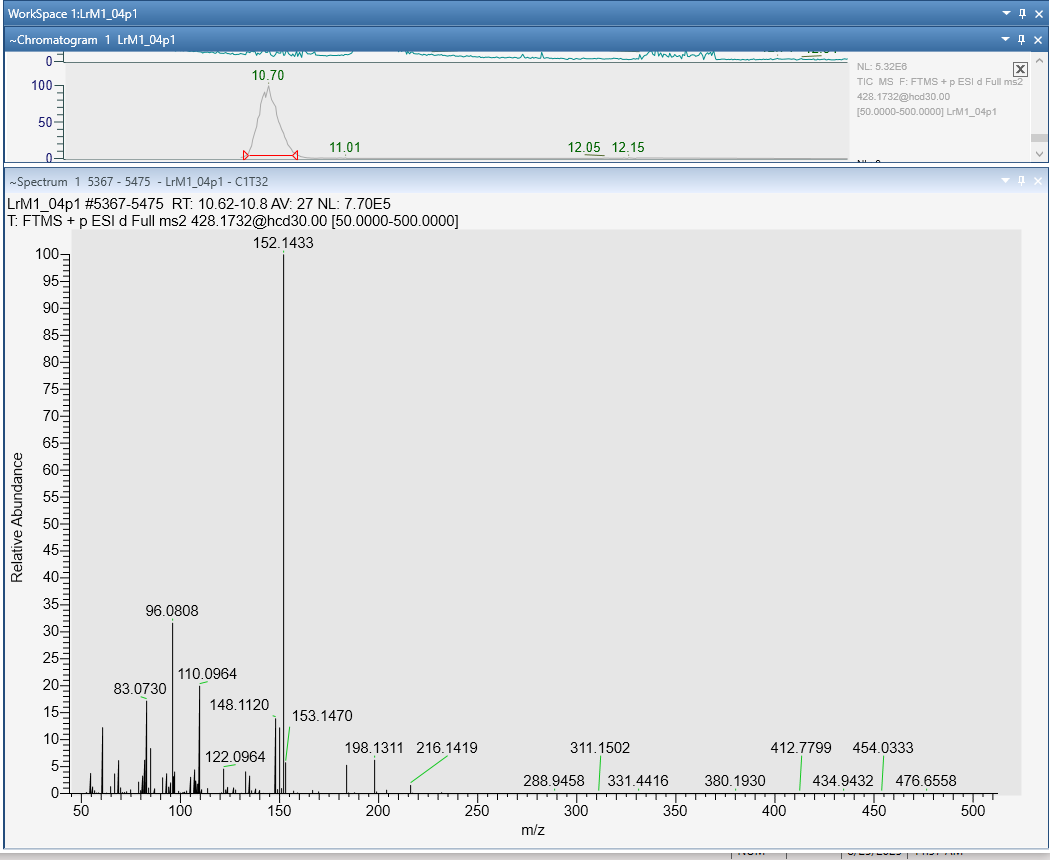


# tentative ds 4-apiosyloxybenzyl (#) 478.1378


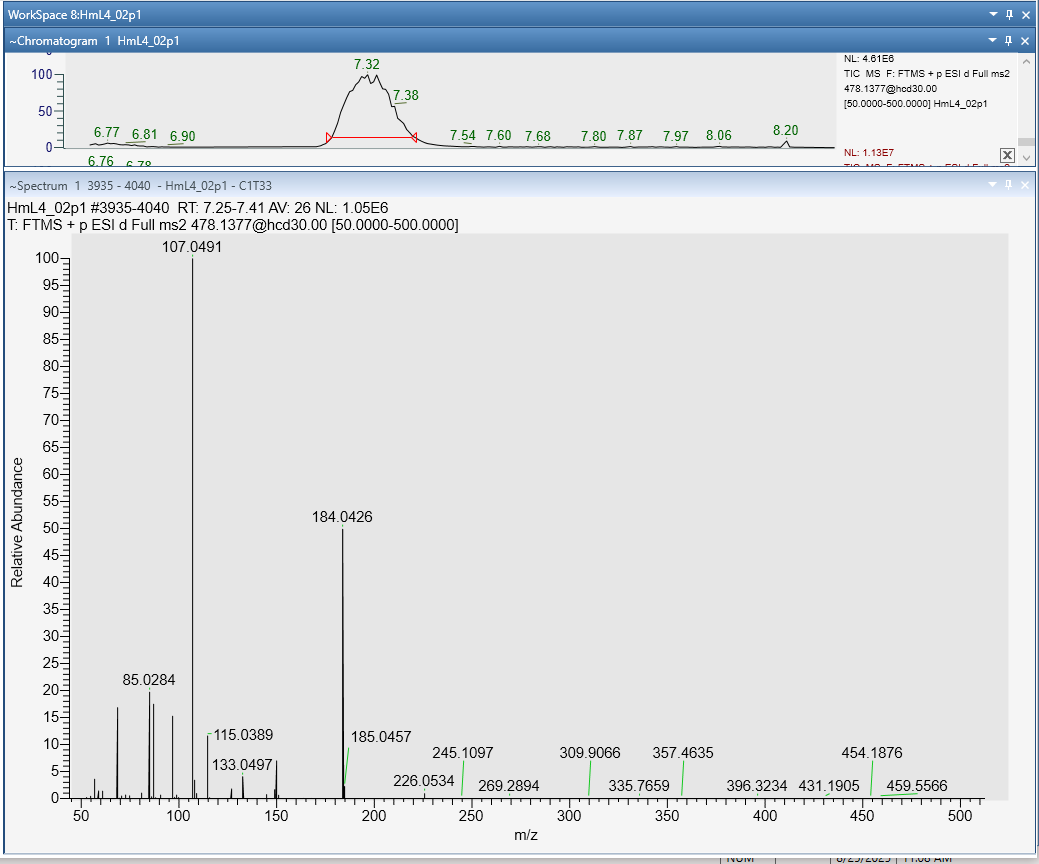


# ds 4-apiosyloxy-3-hydroxybenzyl 494.1327


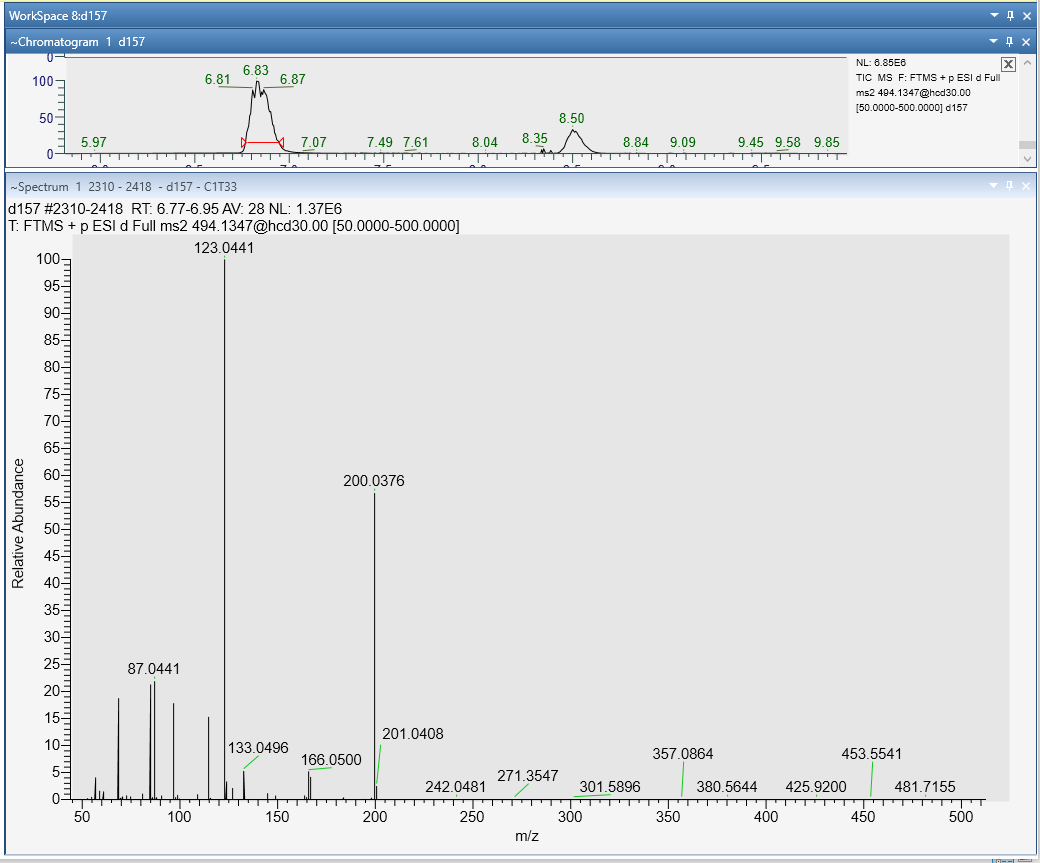


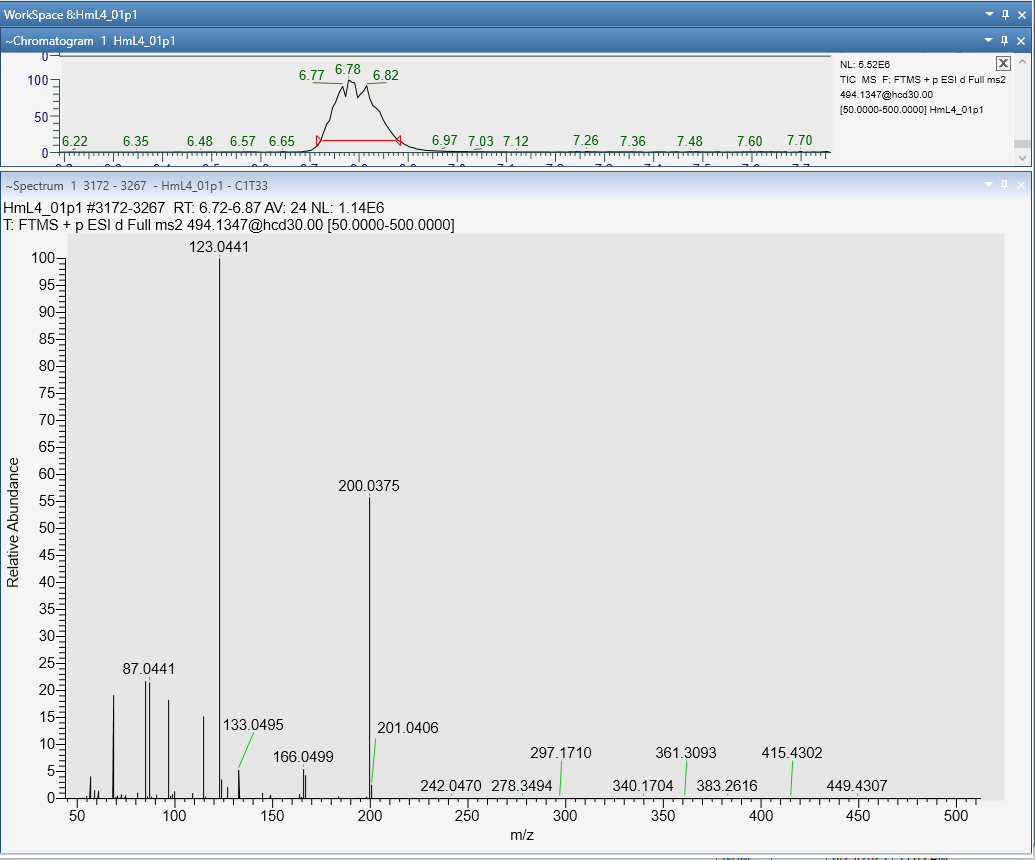

Supplement: S1 Appendix — Spectra were recorded with a collision energy of 30 V except those frame in yellow which were recorded with 10 V collision energy. For assignment of spectra to plant species, the reader is referred to the sample information line in each image. The first two letters indicate the plant species (Ca, Cardamine amara; Ci, C. impatiens; Ld, Lepidium draba; Lr, Lunaria rediviva; Hm, Hesperis matronalis; Cp, C. pratensis; Ds, Descurainia sophia). Primary, secondary, and tertiary alcohols could only be distinguished from one another with the aid of standards. Since theoretical differentiation options, such as dehydration peaks of varying intensities and C-C cleavage because of secondary or tertiary alcohols, did not apply to the standards, no conclusions were drawn about the structures without an existing standard. # indicates that standards were not available (tentative identification). ds, desulfo. (DOCX) [file pone.0336172.s016.docx]
