# Supplementary material for: Glucosinolate diversity in seven field-collected Brassicaceae species
Source: PLoS One. 2025 Nov 13;20(11):e0336172. doi: 10.1371/journal.pone.0336172 (PMC12614607; doi:10.1371/journal.pone.0336172)
Supplement: S2 Appendix — Representative examples. (PDF) [file pone.0336172.s017.pdf]

Cardamine amara

population: 1
sample event: A
(S2 Table)

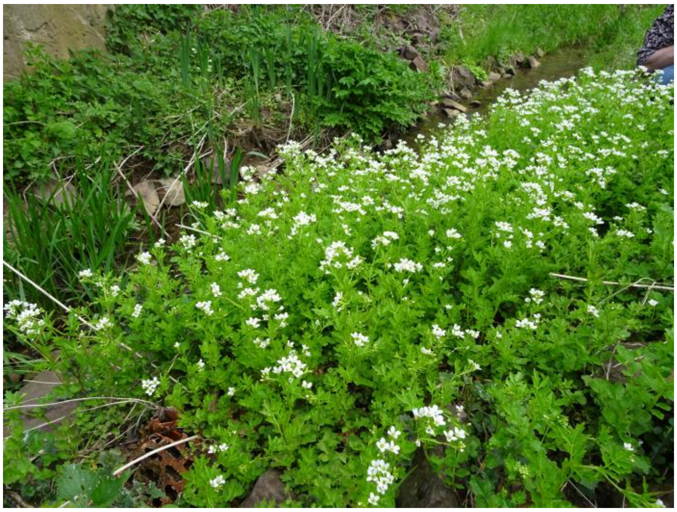

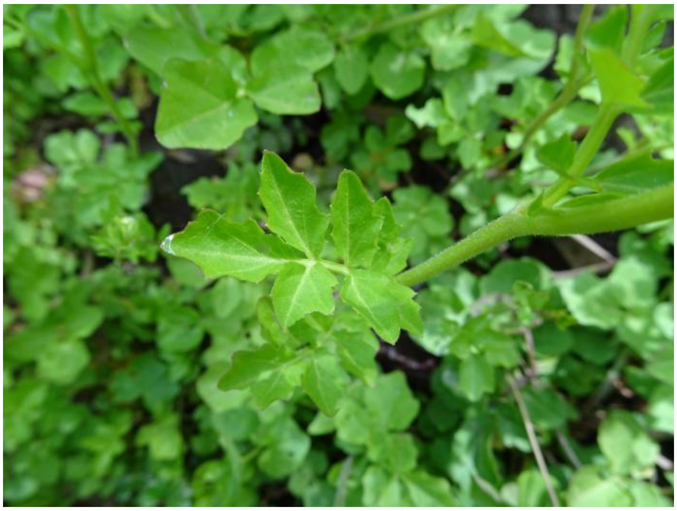

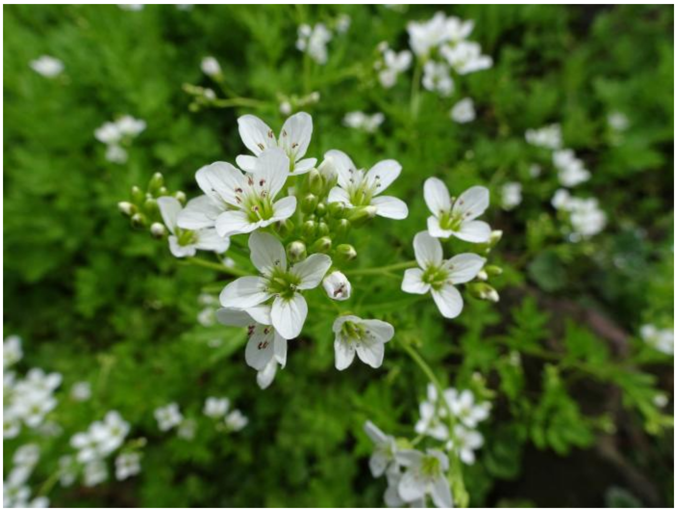

Cardamine amara

population: 1
sample event: C
(S2 Table)

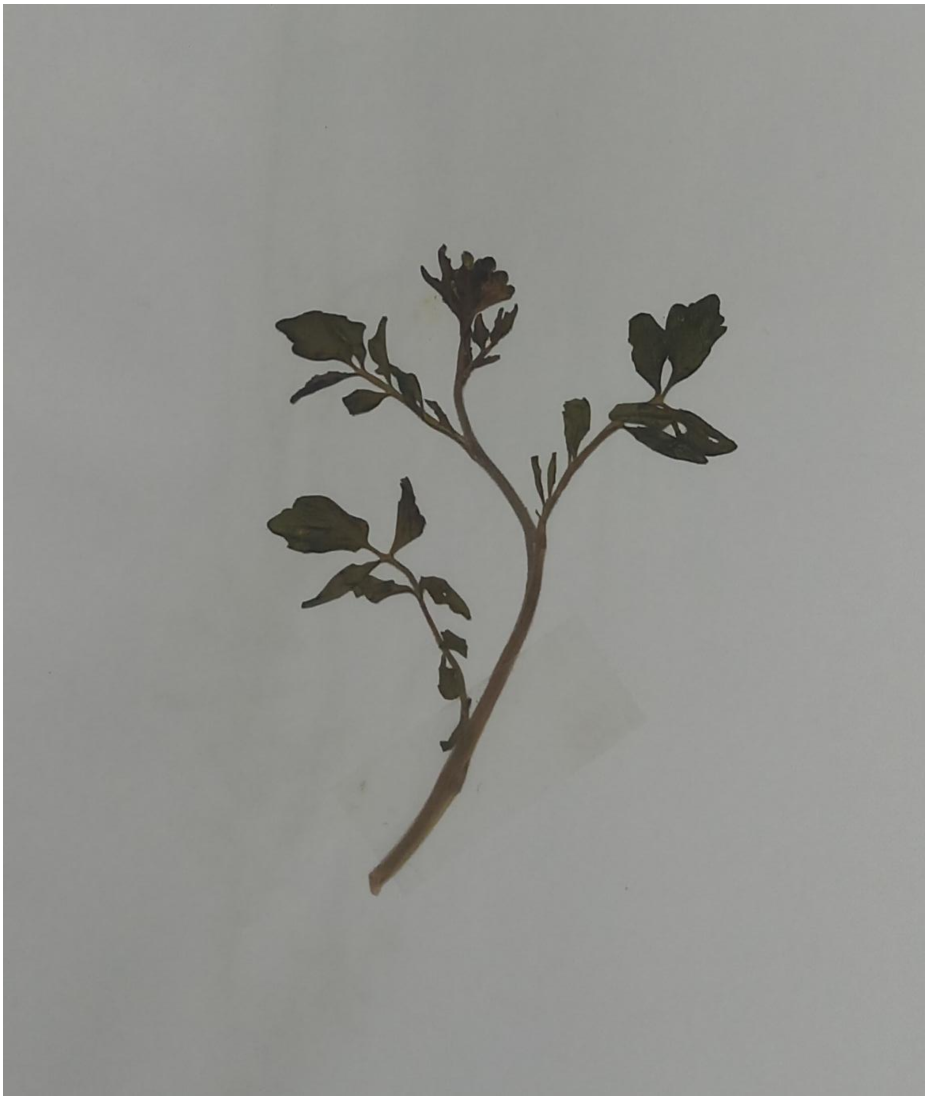

Cardamine impatiens

population: 1
sample event: A
(S2 Table)

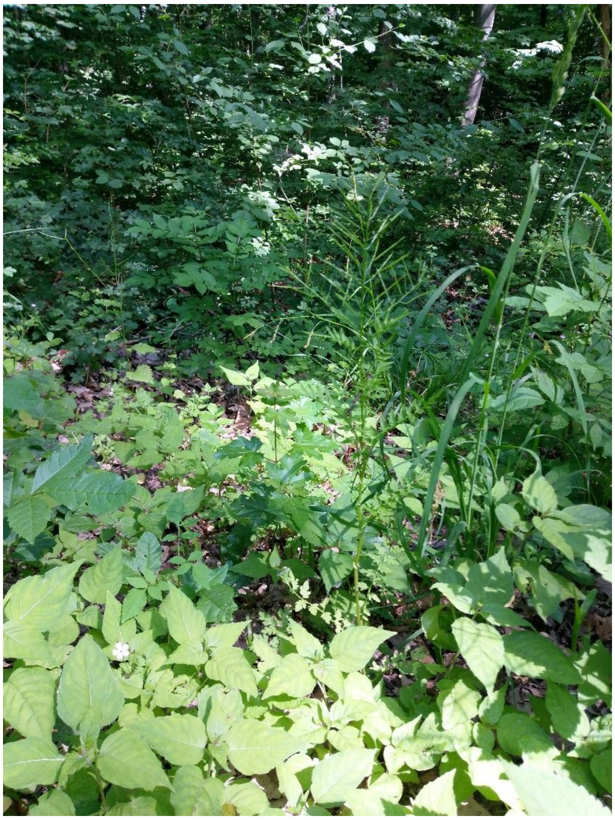

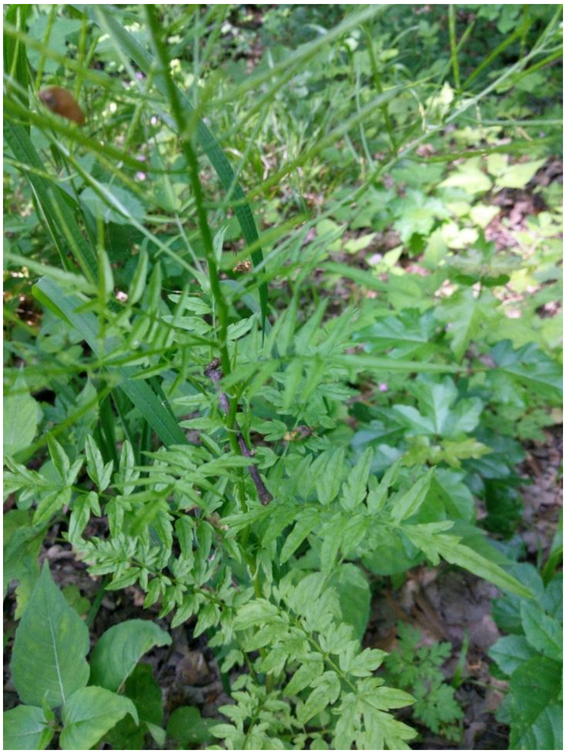

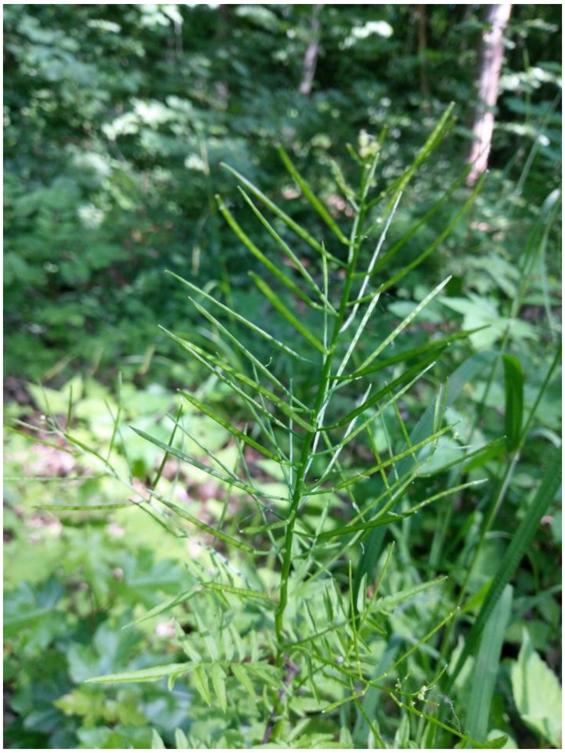

S2 Appendix: Photographical documentation of sampled populations, plants and herbarium specimen. Representative examples.

Cardamine impatiens

population: 2
sample event: B
(S2 Table)

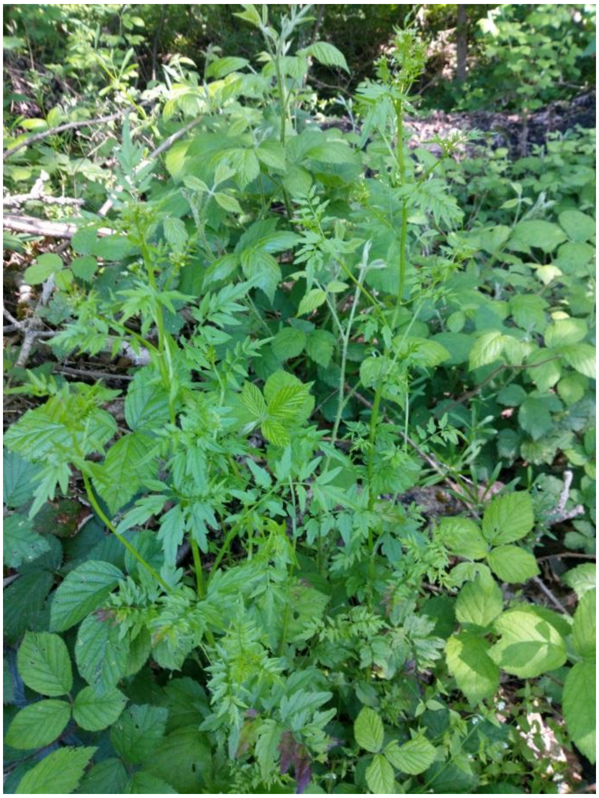

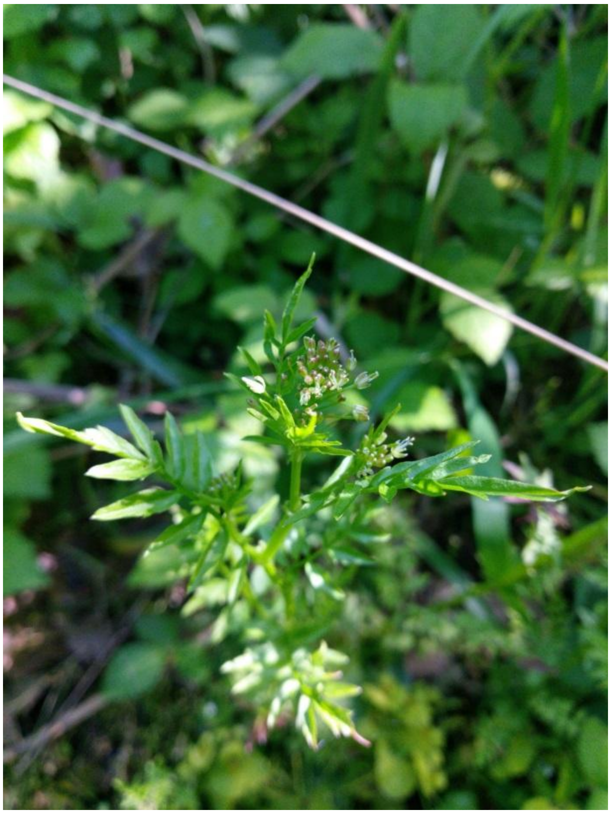

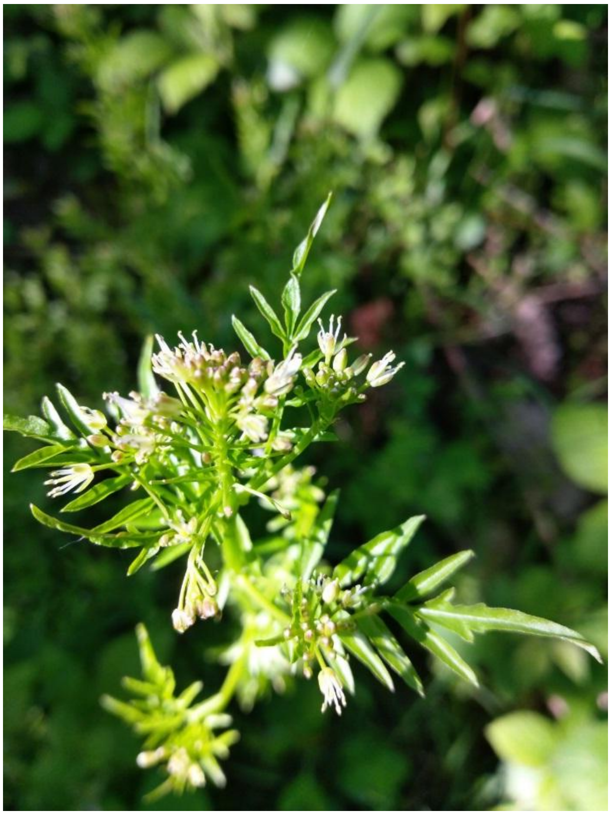

S2 Appendix: Photographical documentation of sampled populations, plants and herbarium specimen. Representative examples.

Cardamine pratensis

population: 4
sample event: G
(S2 Table)

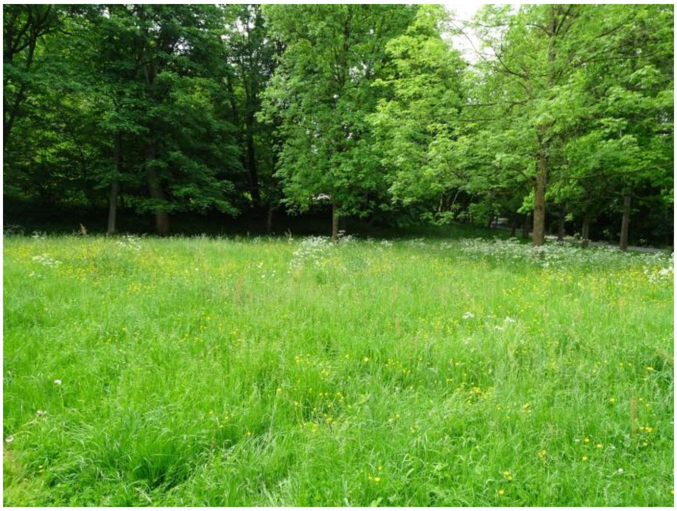

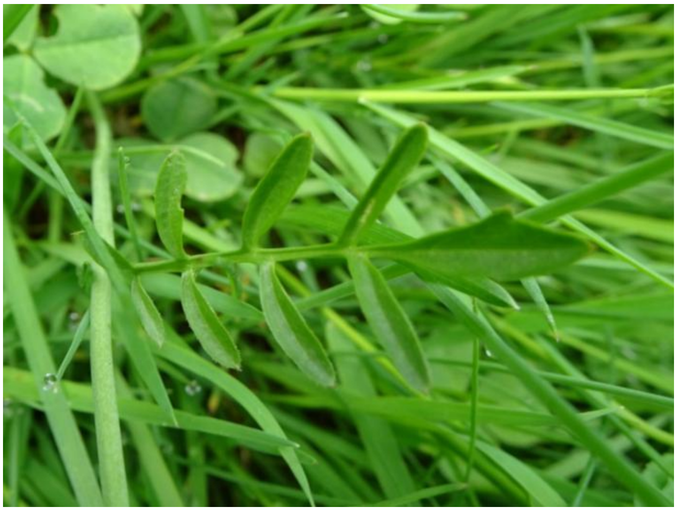

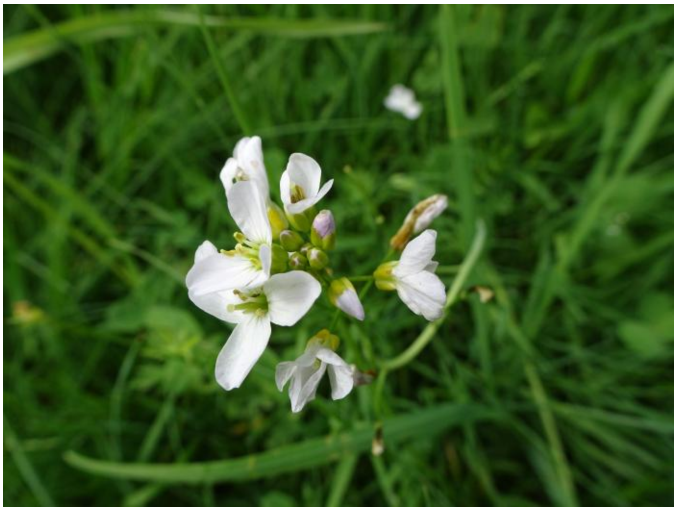

Cardamine pratensis

population: 1
sample event: A
(S2 Table)

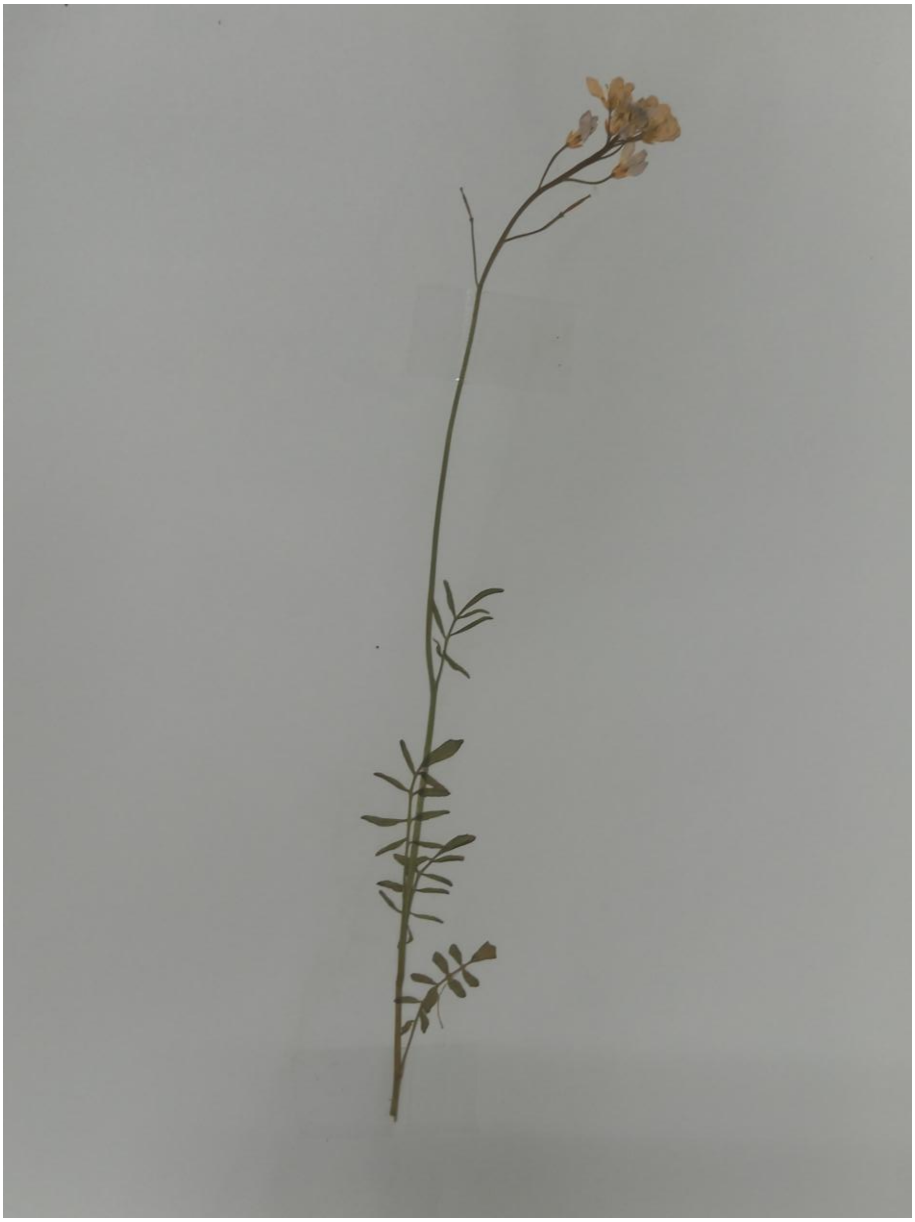

Descurainia sophia

population: 1
sample event: E
(S2 Table)

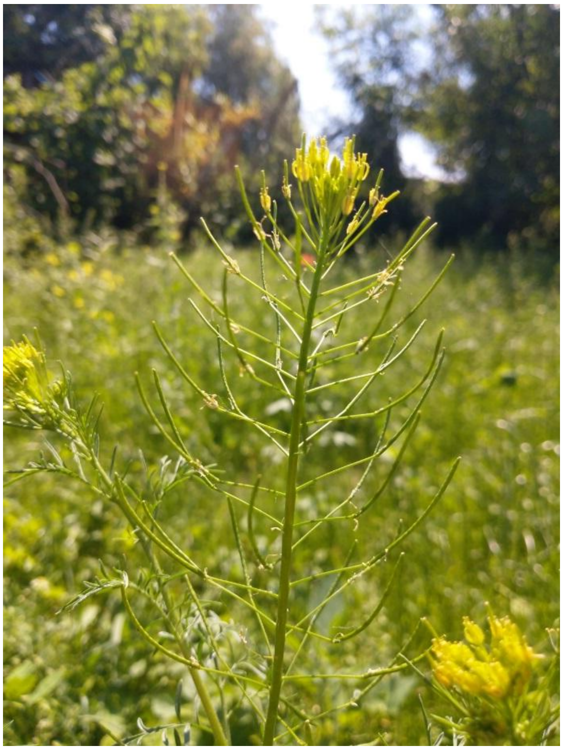

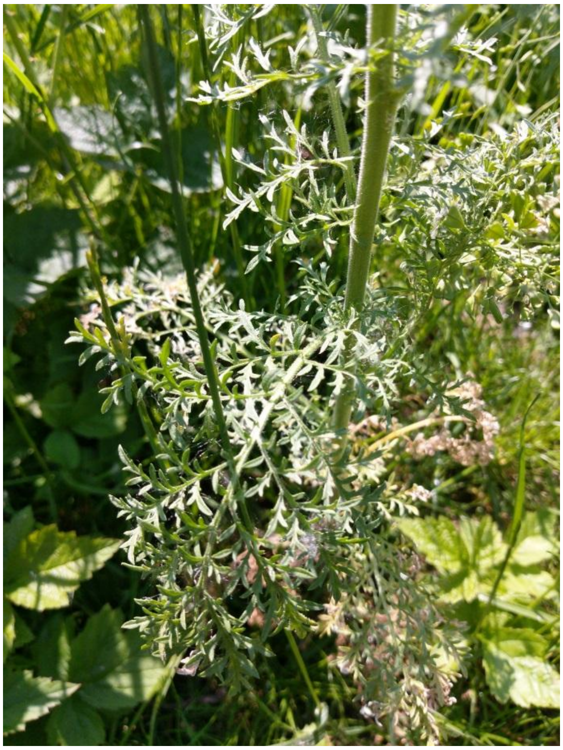

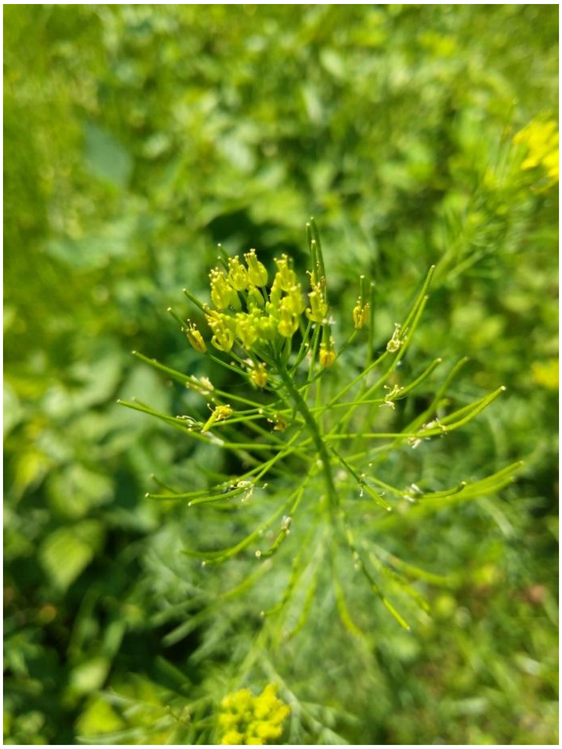

S2 Appendix: Photographical documentation of sampled populations, plants and herbarium specimen. Representative examples.

Hesperis matronalis

population: 1
sample event: A
(S2 Table)

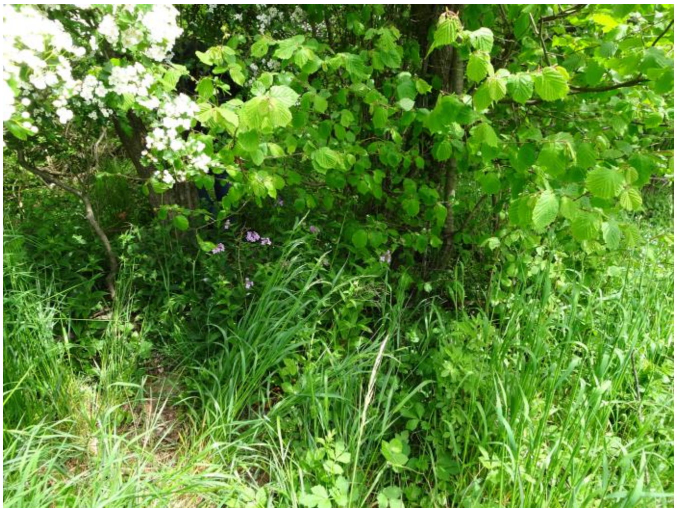

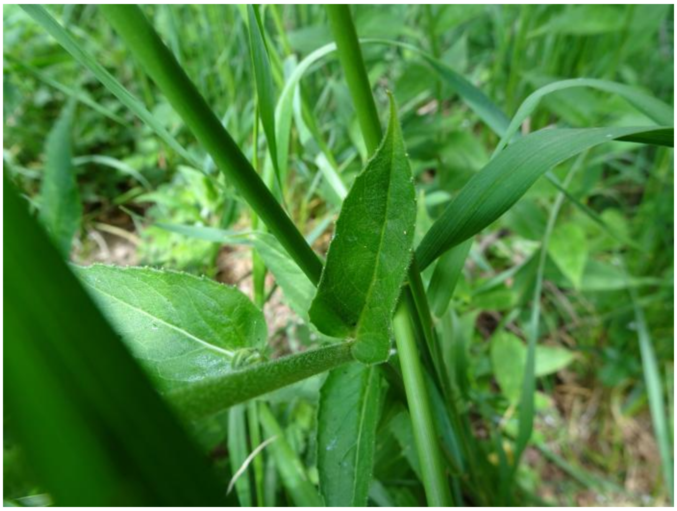

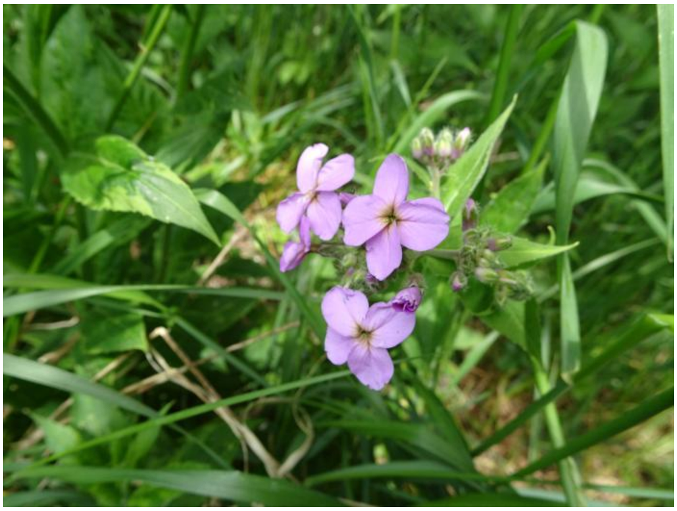

Hesperis matronalis

population: 1
sample event: B
(S2 Table)

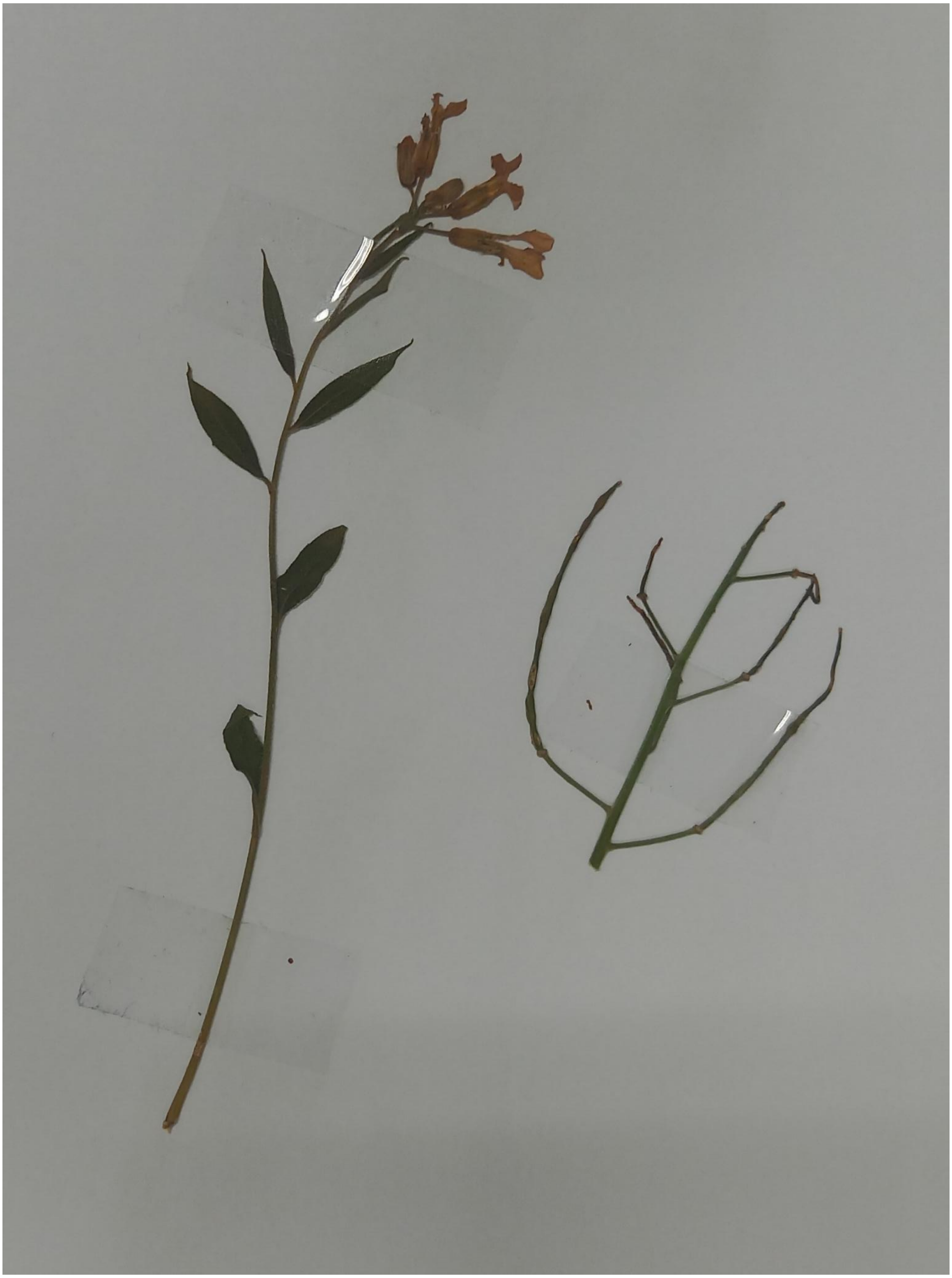

S2 Appendix: Photographical documentation of sampled populations, plants and herbarium specimen. Representative examples.

Lepidium draba

population: 3
sample event: D
(S2 Table)

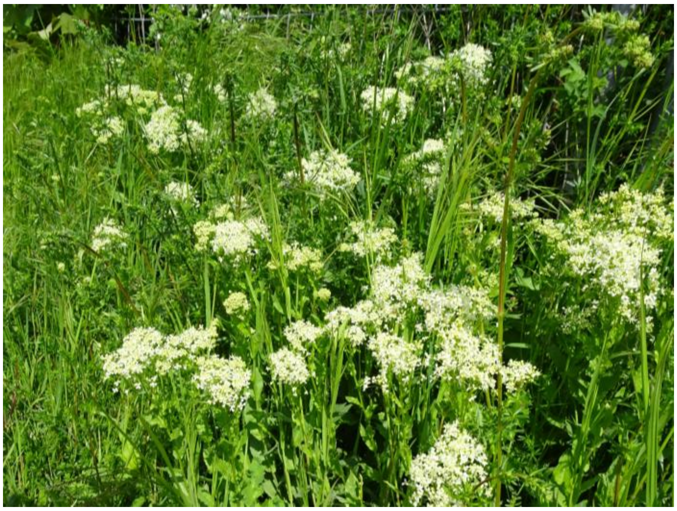

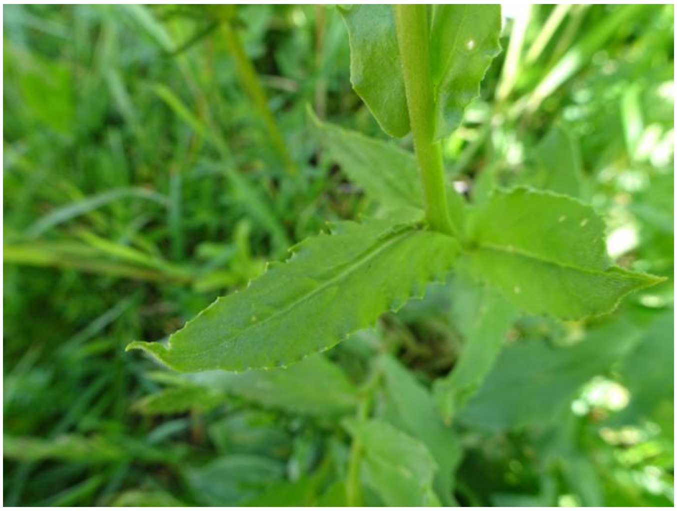

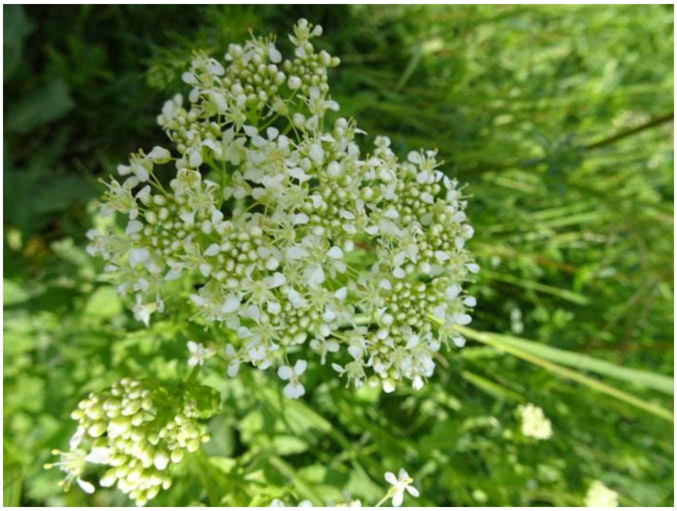

S2 Appendix: Photographical documentation of sampled populations, plants and herbarium specimen. Representative examples.

Lepidium draba

population: 2
sample event: B
(S2 Table)

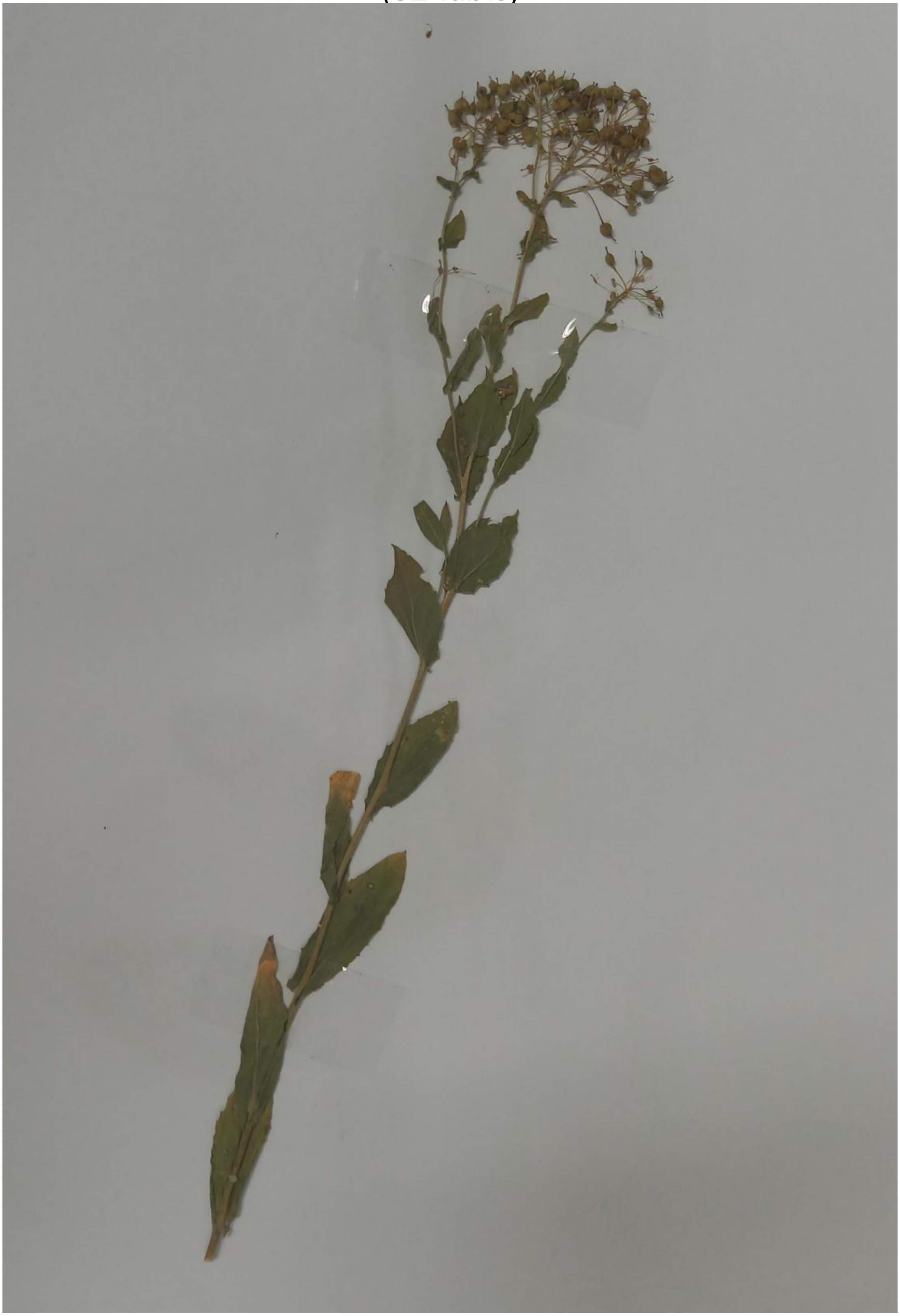

S2 Appendix: Photographical documentation of sampled populations, plants and herbarium specimen. Representative examples.

Lunaria rediviva

population: 1
sample event: A
(S2 Table)

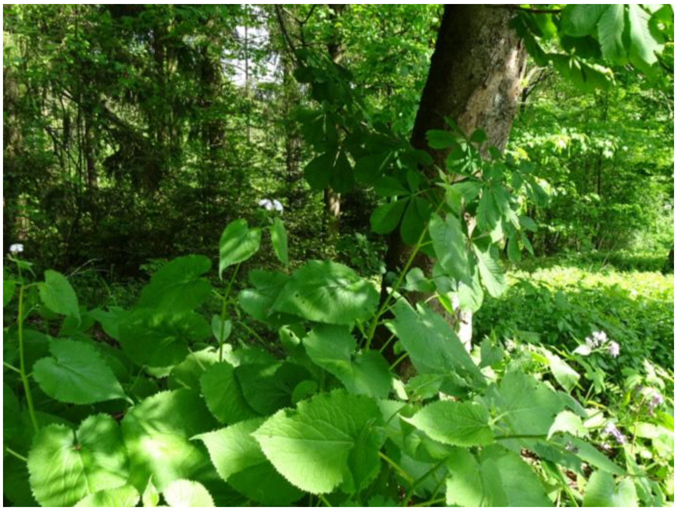

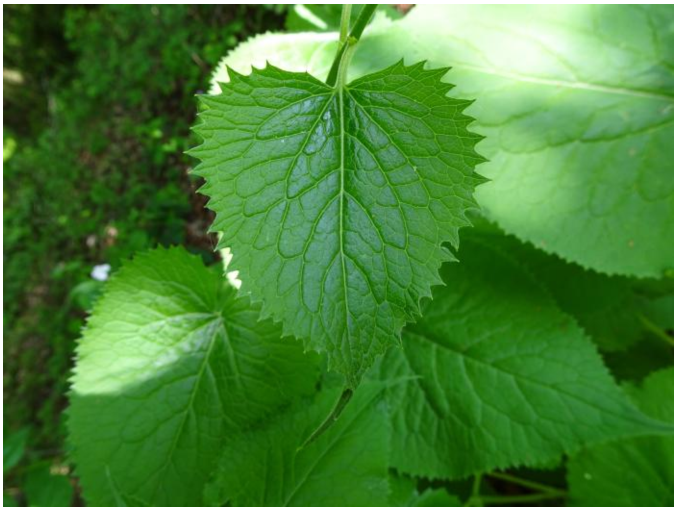

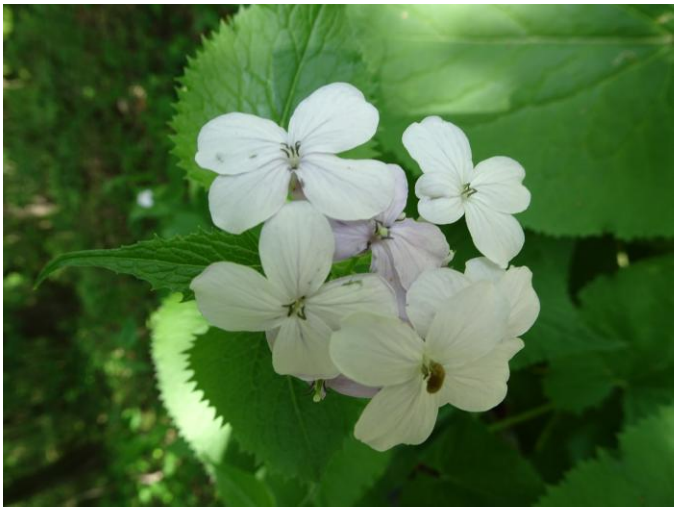

Lunaria rediviva

population: 2
sample event: C
(S2 Table)

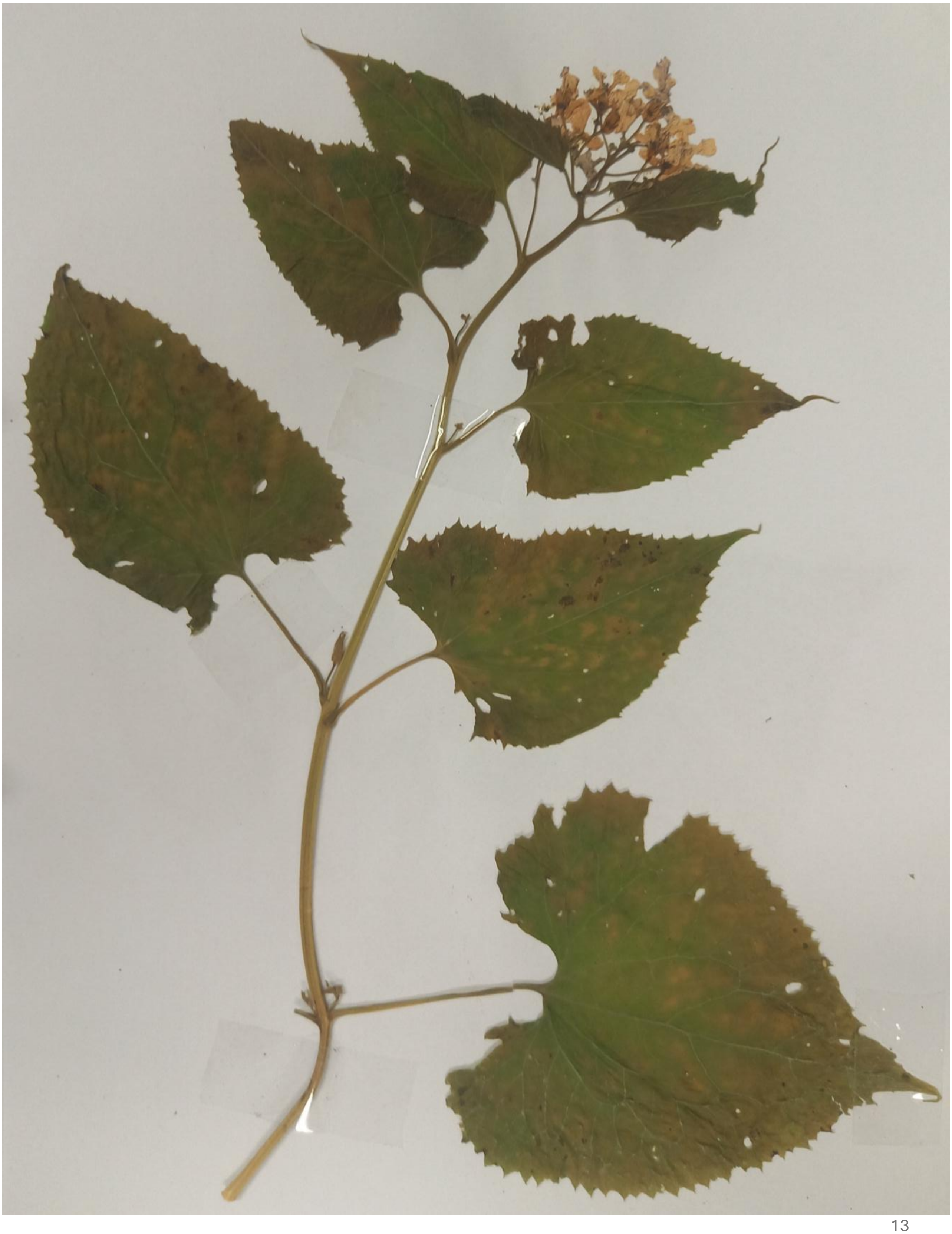

S2 Appendix: Photographical documentation of sampled populations, plants and herbarium specimen. Representative examples.
